# Supplementary material for: Selective peptide bond formation via side chain reactivity and self-assembly of abiotic phosphates
Source: Nat Commun. 2025 Feb 3;16:1306. doi: 10.1038/s41467-025-56432-6 (PMC11790832; doi:10.1038/s41467-025-56432-6)
Supplement: Supplementary file 1 — Supplementary Information [file 41467_2025_56432_MOESM1_ESM.pdf]

Supplementary Information

Selective Peptide Bond Formation via Side Chain Reactivity and Self-Assembly of Abiotic Phosphates

Arti Sharma,[1,2] Kun Dai[3], Mahesh D. Pol[2,3], Ralf Thomann[1,4], Yi Thomann[1], Subhra Kanti Roy[2], Charalampos G. Pappas[1,2,3*]

[1]FIT – Freiburg Center for Interactive Materials and Bioinspired Technologies, University of Freiburg, Georges-Köhler-Allee 105, 79110, Freiburg, Germany. [2]Institute of Organic Chemistry, University of Freiburg, Albert-Strasse 21, 79104, Freiburg, Germany. [3]DFG Cluster of Excellence *liv*MatS @FIT – Freiburg Center for Interactive Materials and Bioinspired Technologies, University of Freiburg, Georges-Köhler-Allee 105, 79110, Freiburg, Germany. [4]Freiburg Materials Research Center (FMF), University of Freiburg, Stefan-Meier-Strasse 21, 79104, Freiburg, Germany.

Table of Contents

1. Methods 3

2. Synthesis and characterization of Aminoacyl phosphate esters (1a, 1b, 1d, 1e, 1f, 1g, 2a, 2b, 2c, 2d 3) 5

2.1 General synthesis

2.1.1 General synthesis of phosphate salts 5

2.1.2 General synthesis of Boc protected aminoacyl phosphate esters

2.1.3 General synthesis of non protected aminoacyl phosphate esters

2.2 Characterization of compounds 7

2.2.1 Characterization of aminoacyl phosphate esters (APs) by NMR 7

2.2.2 Characterization of APs by UPLC-MS 23

3. Supplementary Figures 32

4. References 146

1. Methods

UPLC methods:

A)

Time (min)	Eluent-A (Water) %	Eluent-B (ACN) %
0	95	5
15	30	70
16	10	90
17	10	90
17.5	95	5
20	95	5

B)

Time (min)	Eluent-A (Water) %	Eluent-B (ACN) %
0	95	5
3	50	50
15	10	90
16	10	90
17	10	90
17.5	95	5
20	95	5

C)

Time (min)	Eluent-A (Water) %	Eluent-B (ACN) %
0	95	5
15	30	70
20	30	70
21	10	90
22	10	90
22.5	95	5
25	95	5

D)

Time (min)	Eluent-A (Water) %	Eluent-B (ACN) %
0	95	5
15	10	90
16	10	90
17	10	90
17.5	95	5
20	95	5

UPLC methods for the analysis of libraries.

Method A) was used for the libraries of **2a**, **2c and 2d** with all amino acids and amino acids mixtures.

Method B) was used for the libraries of **2b** and **3** with all natural amino acids, amino acids mixtures and dipeptides.

Method C) was used for the individual and mixed libraries of **1a, 1b, 1d, 1e, 1f, 1g**.

Method D) was used for the libraries of **2b** and **3** with amino acid mixture of R (arginine) and F(4-guanidine).

2. Synthesis and characterization of aminoacyl phosphate esters (Boc-FEP, Boc-VEP, Boc-AEP, Boc-F(4-NH-Fmoc)EP, Boc-F(4-NH-Fmoc)PP, F(4-NH-Fmoc)EP, FEP, VEP, F(4-COOH)EP, F(4-gua)EP, BPAEP

2.1 General synthesis

I. General procedure for the synthesis of Bis(tetraethylammonium salt) ethyl/phenyl phosphate:

Synthesis of aminoacyl phosphate esters was done according to the previously reported procedure.[1]

II. General procedure for the synthesis of Boc-aminoacyl phosphate esters:

N-Boc-L-amino acid (3.75 mmol, 1.0 eq) was dissolved in dry dichloromethane (DCM) (30 mL), followed by the addition of DCC (3.75 mmol, 1.0 eq), resulting in the formation of a white precipitate after stirring for 3-5 minutes. Subsequently, bis(tetraethylammonium salt) ethyl phosphate (3.75 mmol, 1.2 eq), previously dissolved in dry DCM (10 mL), was added and the reaction mixture was stirred for 1 hour at room temperature. The reaction progress was monitored via TLC analysis [using MeOH:DCM (10:90)]. Afterwards, the reaction mixture was filtered to remove the white precipitate of DCU. The filtrate was then evaporated under reduced pressure, and the resulting residue was purified using automated RP flash column chromatography (C18-AQ) with a gradient of H_2O and acetonitrile. Fractions containing the desired product were collected and lyophilized to yield the aminoacyl phosphate esters as tetraethylammonium salts.

III. General procedure for ion exchange:

The TEA salt of Boc aminoacyl phosphate esters was dissolved in a small amount of water and then passed through a column containing Dowex® 50WX8 ion exchange resin with Na^+ counter ions. The resulting solution was collected and frozen in liquid nitrogen before being subjected to lyophilization until complete drying. This process results in the formation of the sodium salt of Boc-aminoacyl phosphate esters, appearing as a white hygroscopic solid.

IV. General procedure for the Boc deprotection:

The TEA salt of N-t-Boc aminoacyl ethyl phosphate was dissolved in a small quantity of trifluoroacetic acid (TFA). After allowing 10-15 minutes for the reaction, the TFA was removed through rotary evaporation at 30°C under vacuum pumping. The resulting oil was then dissolved in a mixture of cold acetone and diethyl ether. The resulting precipitate was collected via vacuum filtration and subsequently washed multiple times with acetone and diethyl ether.

After drying under a stream of nitrogen for 10 minutes, the aminoacyl ethyl phosphate ester was obtained as a solid.

2.2 Characterization of compounds

2.2.1 Characterization of Boc-aminoacyl phosphate esters (Boc-APs) by NMR

[I] (S)-3-(4-((((9H-fluoren-9-yl)methoxy)carbonyl)amino)phenyl)-2-((tert-butoxycarbonyl)amino)propanoic (ethyl phosphoric) anhydride:

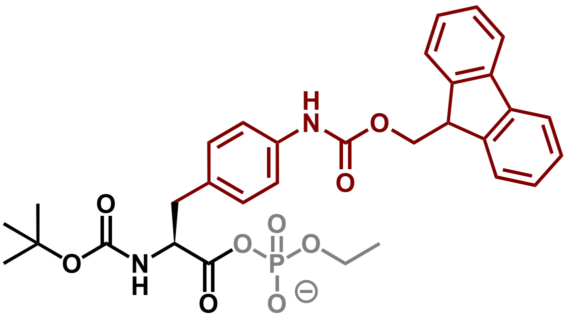

Boc-F(4-NH-Fmoc)EP (2b)

The title compound was prepared according to the general procedure [I &II] as white hygroscopic solid in 42% yield. **^{1}H NMR (400 MHz, Chloroform-*d*)** δ 7.77 (d, *J* = 7.5 Hz, 2H), 7.64 (d, *J* = 7.4 Hz, 2H), 7.40 (t, *J* = 7.5 Hz, 2H), 7.32 (t, *J* = 7.4 Hz, 4H), 7.19 (d, *J* = 8.3 Hz, 2H), 5.00 (d, *J* = 8.1 Hz, 1H), 4.48 (d, *J* = 6.9 Hz, 3H), 4.27 (t, *J* = 6.9 Hz, 1H), 4.04 (p, *J* = 7.1 Hz, 2H), 3.34 (q, *J* = 7.2 Hz, 8H), 3.12 (tt, *J* = 14.1, 6.9 Hz, 2H), 1.39 (s, 9H), 1.27 (dt, *J* = 17.1, 7.2 Hz, 15H); **^{31}P NMR (162 MHz, Chloroform-*d*)** *δ* (ppm) -7.61.

[ii] (S)-3-(4-((((9H-fluoren-9-yl)methoxy)carbonyl)amino)phenyl)-2-((tert-butoxycarbonyl)amino)propanoic (phenyl phosphoric) anhydride:

The title compound was prepared according to the general procedure [I &II] as white hygroscopic solid in 39% yield. **^{1}H NMR (400 MHz, Chloroform-*d*)** δ 7.77 (d, *J* = 7.5 Hz, 2H), 7.63 (d, *J* = 7.4 Hz, 2H), 7.40 (t, *J* = 7.5 Hz, 2H), 7.31 (ddd, *J* = 11.2, 6.1, 2.4 Hz, 5H), 7.23 (dd, *J* = 8.6, 7.1 Hz, 2H), 7.10 (d, *J* = 8.2 Hz, 2H), 7.00 (td, *J* = 7.2, 1.5 Hz, 1H), 4.93 (d, *J* = 8.2 Hz,

1H), 4.47 (d, *J* = 7.0 Hz, 3H), 4.26 (t, *J* = 6.8 Hz, 1H), 3.31 (q, *J* = 7.2 Hz, 8H), 3.14 – 2.95 (m, 2H), 1.38 (s, 9H), 1.27 (t, *J* = 7.5 Hz, 13H); **^{31}P NMR (162 MHz, Chloroform-*d*)** *δ* (ppm) -13.88.

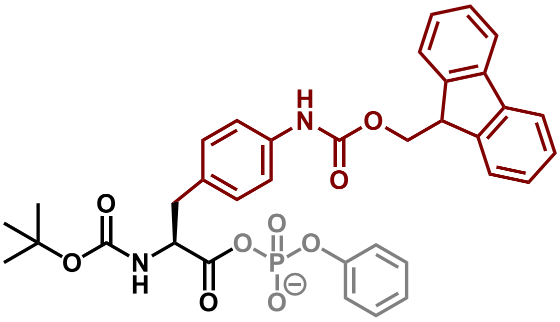

TEA salt

Boc-F(4-NH-Fmoc)PP (3)

[iii] (S)-2-((tert-butoxycarbonyl)amino)-3-phenylpropanoic (ethyl phosphoric) anhydride:

The title compound was prepared according to the general procedure [I, II & III] as white hygroscopic solid in 45% yield. **^{1}H NMR (300 MHz, Chloroform-*d*)** δ 7.20 (tt, *J* = 9.6, 5.2 Hz, 5H), 5.75 – 5.64 (m, 1H), 4.52 (td, *J* = 8.7, 4.6 Hz, 1H), 3.98 (p, *J* = 7.2 Hz, 2H), 3.24 (dd, *J* = 14.1, 4.6 Hz, 1H), 2.97 (dd, *J* = 14.1, 9.2 Hz, 1H), 1.43 – 1.01 (m, 13H); **^{31}P NMR (122 MHz, Chloroform-*d*)** *δ* (ppm) -6.29.

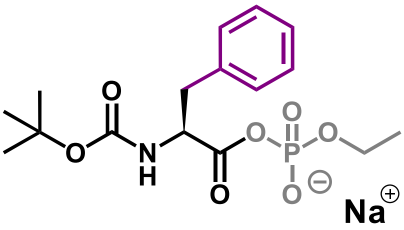

Boc-FEP (2a)

[iv] (S)-2-((tert-butoxycarbonyl)amino)propanoic (ethyl phosphoric) anhydride:

The title compound was prepared according to the general procedure [I, II & III] as white solid in 50% yield. **^{1}H NMR (400 MHz, D_2O)** δ 4.23 (d, *J* = 7.3 Hz, 1H), 4.13 – 4.00 (m, 2H), 1.52 – 1.39 (m, 13H), 1.37 – 1.24 (m, 3H); **^{31}P NMR (162 MHz, D_2O)** δ (ppm) -7.24.

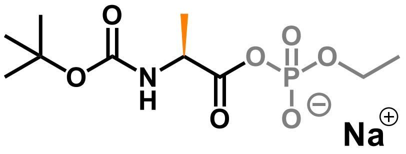

Boc-AEP (2c)

[v] (S)-2-((tert-butoxycarbonyl)amino)-3-methylbutanoic (ethyl phosphoric) anhydride:

The title compound was prepared according to the general procedure [I, II & III] as light yellow solid in 48% yield. **^{1}H NMR (400 MHz, D_2O)** δ 4.11 – 4.08 (m, 1H), 2.24 (dt, *J* = 13.2, 6.6 Hz, 1H), 1.47 (s, 9H), 1.31 (tt, *J* = 7.2, 0.8 Hz, 3H), 1.00 (dd, *J* = 17.3, 6.9 Hz, 6H); **^{31}P NMR (162 MHz, D_2O)** δ (ppm) -7.24.

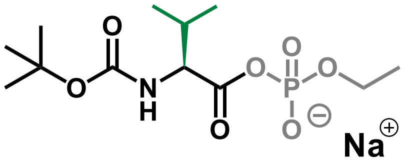

Boc-VEP (2d)

2.2.2 Characterization of aminoacyl phosphate esters (APs) by NMR

[i](*S*)-3-(4-((((9H-fluoren-9-yl)methoxy)carbonyl)amino)phenyl)-2-aminopropanoic (ethyl phosphoric) anhydride:

The title compound was prepared according to the general procedure [I & III] as white solid in 54% yield. **^{1}H NMR (300 MHz, DMSO-d_6)** δ (ppm) 9.71 (s, 1H), 8.33 (bs, 2H), 7.91 (d, *J* = 7.4 Hz, 2H), 7.78-7.69 (m, 2H), 7.74 (d, *J* = 7.3 Hz, 2H), 7.38 (dq, *J* = 14.7, 7.4 Hz, 6H), 7.20 (d, *J* = 8.0 Hz, 2H), 4.48 (d, *J* = 6.7 Hz, 2H), 4.30 (t, *J* = 6.5 Hz, 1H), 4.15 (t, *J* = 5.9 Hz, 1H), 3.81

– 3.60 (m, 2H), 3.05 (d, *J* = 5.6 Hz, 2H), 1.08 (t, *J* = 7.0 Hz, 3H); **^{31}P NMR (122 MHz, DMSO-*d$_6$*)** *δ* (ppm) -9.03.

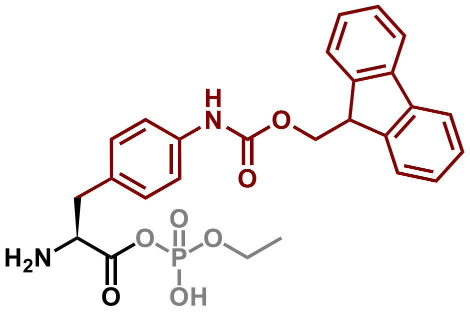

F(4-NH-Fmoc)EP (1b)

[ii] (*S*)-2-amino-3-phenylpropanoic (ethyl phosphoric) anhydride:

The title compound was prepared according to the general procedure [I & III] as white solid in 48% yield. **^{1}H NMR (400 MHz, D_2O)** *δ* (ppm) 7.51 – 7.36 (m, 5H), 4.54 (dd, *J* = 7.3, 6.1 Hz, 1H), 4.00 (dq, *J* = 8.1, 7.1 Hz, 2H), 3.43 (dd, *J* = 14.6, 6.2 Hz, 1H), 3.33 (dd, *J* = 14.6, 7.3 Hz, 1H), 1.31 – 1.26 (m, 3H); **^{31}P NMR (162 MHz, D_2O)** δ (ppm) -7.54.

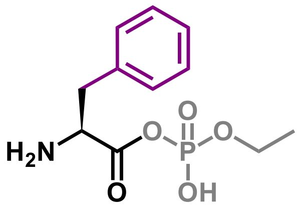

FEP (1a)

[iii] 4-((2S)-2-amino-3-((ethoxy(hydroxy)phosphoryl)oxy)-3-oxopropyl)benzoic acid:

The title compound was prepared according to the general procedure [I & III] as white solid in 51% yield. **^{1}H NMR (400 MHz, D_2O)** *δ* 8.15 – 7.92 (m, 2H), 7.56 – 7.41 (m, 2H), 4.57 (t, *J* = 6.9 Hz, 1H), 3.87 (p, *J* = 7.3 Hz, 2H), 3.42 (dd, *J* = 7.0, 4.6 Hz, 2H), 1.19 (t, *J* = 7.1 Hz, 3H); **^{31}P NMR (162 MHz, D_2O)** δ (ppm) -7.63.

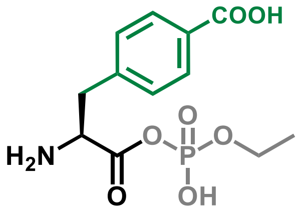

F(4-COOH)EP (1g)

[iv] (S)-2-amino-3-(4-benzoylphenyl)propanoic (ethyl phosphoric) anhydride:

The title compound was prepared according to the general procedure [I & III] as white solid in 55% yield. **^{1}H NMR (300 MHz, DMSO-*d*$_6$)** δ 8.58 (s, 2H), 7.85 – 7.60 (m, 5H), 7.60 – 7.41 (m, 4H), 4.29 (t, *J* = 6.5 Hz, 1H), 3.78 – 3.61 (m, 2H), 3.38 (q, *J* = 7.0 Hz, 1H), 3.19 (dd, *J* = 9.7, 4.8 Hz, 1H), 1.08 (q, *J* = 6.8 Hz, 4H); **^{31}P NMR (122 MHz, DMSO-*d*$_6$)** δ (ppm) -9.44.

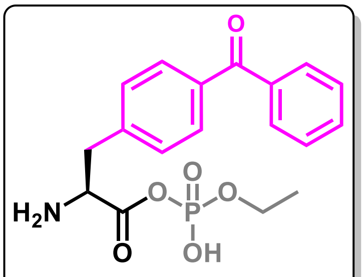

BPAEP (1f)

[v] (S)-2-amino-3-(4-guanidinophenyl)propanoic (ethyl phosphoric) anhydride:

The title compound was prepared according to the general procedure [I & III] as pale yellow solid in 45 % yield. **^{1}H NMR (400 MHz, DMSO-*d*$_6$)** δ 10.31 (s, 1H), 8.40 (s, 2H), 7.60 (s, 4H), 7.43 – 7.24 (m, 2H), 7.13 (d, J = 7.9 Hz, 2H), 4.23 (t, J = 6.6 Hz, 1H), 3.88 – 3.72 (m, 2H), 3.21 (ddt, J = 10.5, 7.2, 3.6 Hz, 1H), 3.13 – 3.00 (m, 1H), 1.12 (t, J = 7.1 Hz, 3H); **^{31}P NMR (162 MHz, DMSO-*d*$_6$)** δ (ppm) -8.96.

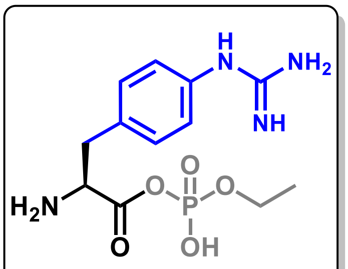

F(4-gua)EP (1c)

[vi] (S)-2-amino-3-methylbutanoic (ethyl phosphoric) anhydride:

The title compound was prepared according to the general procedure [I & III] as white solid in 60% yield. **^{1}H NMR (400 MHz, D_2O)** δ 4.15 (d, J = 4.3 Hz, 1H), 4.09 (dq, J = 8.2, 7.1 Hz, 2H), 2.45 (pd, J = 7.0, 4.3 Hz, 1H), 1.31 (td, J = 7.1, 1.0 Hz, 3H), 1.12 (dd, J = 7.0, 3.9 Hz, 6H); **^{31}P NMR (162 MHz, D_2O)** δ (ppm) -7.53.

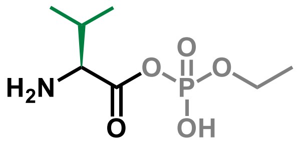

VEP (1d)

EP-Salt ^{1}H, 300 MHz, D_2O

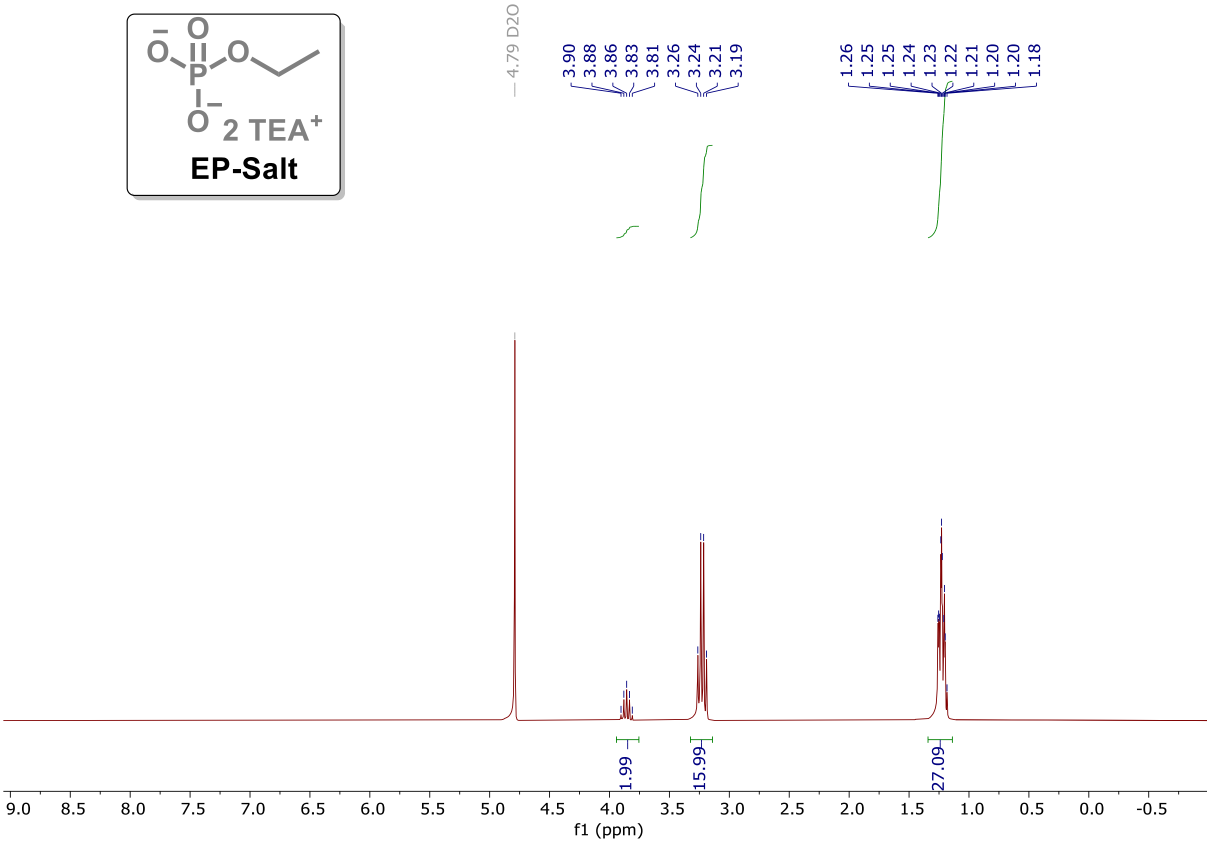

EP-Salt ^{31}P, 122 MHz, D_2O

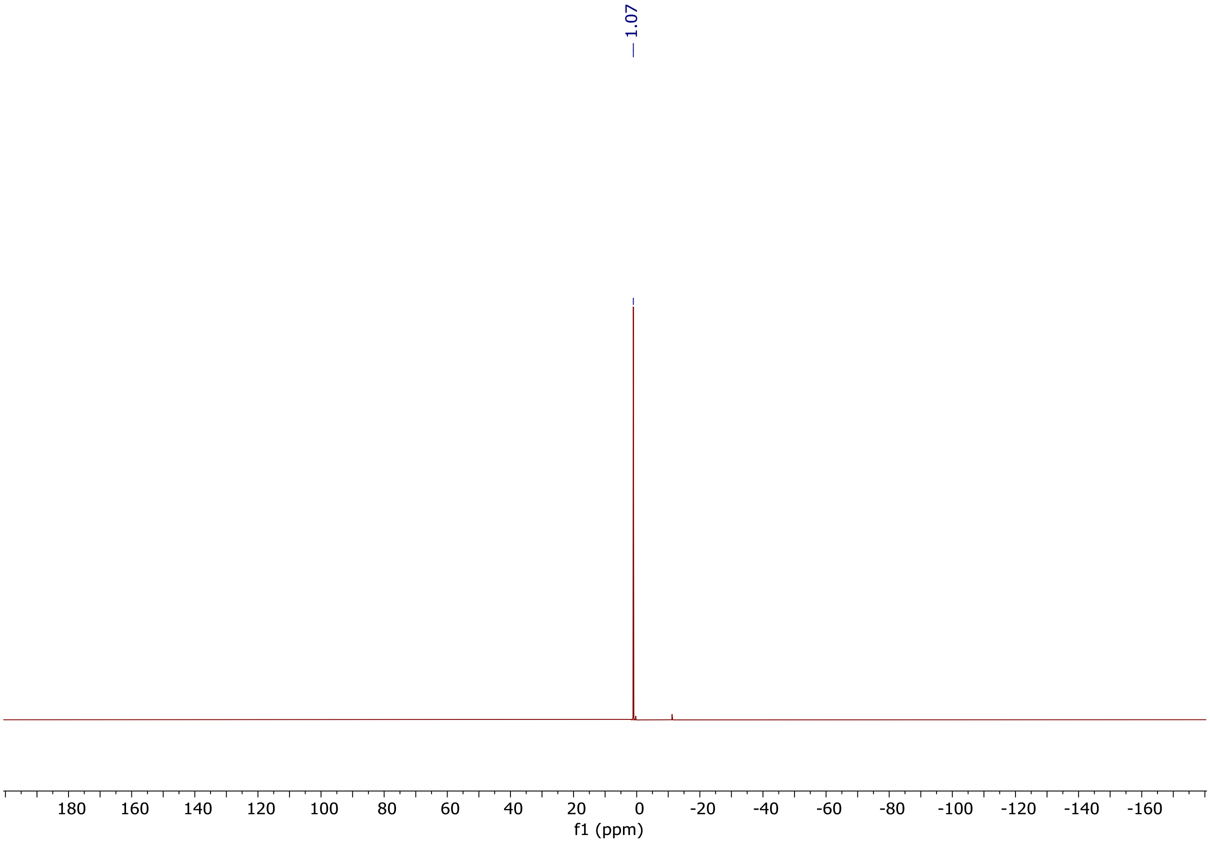

PP-Salt ^{1}H, 300 MHz, D_2O

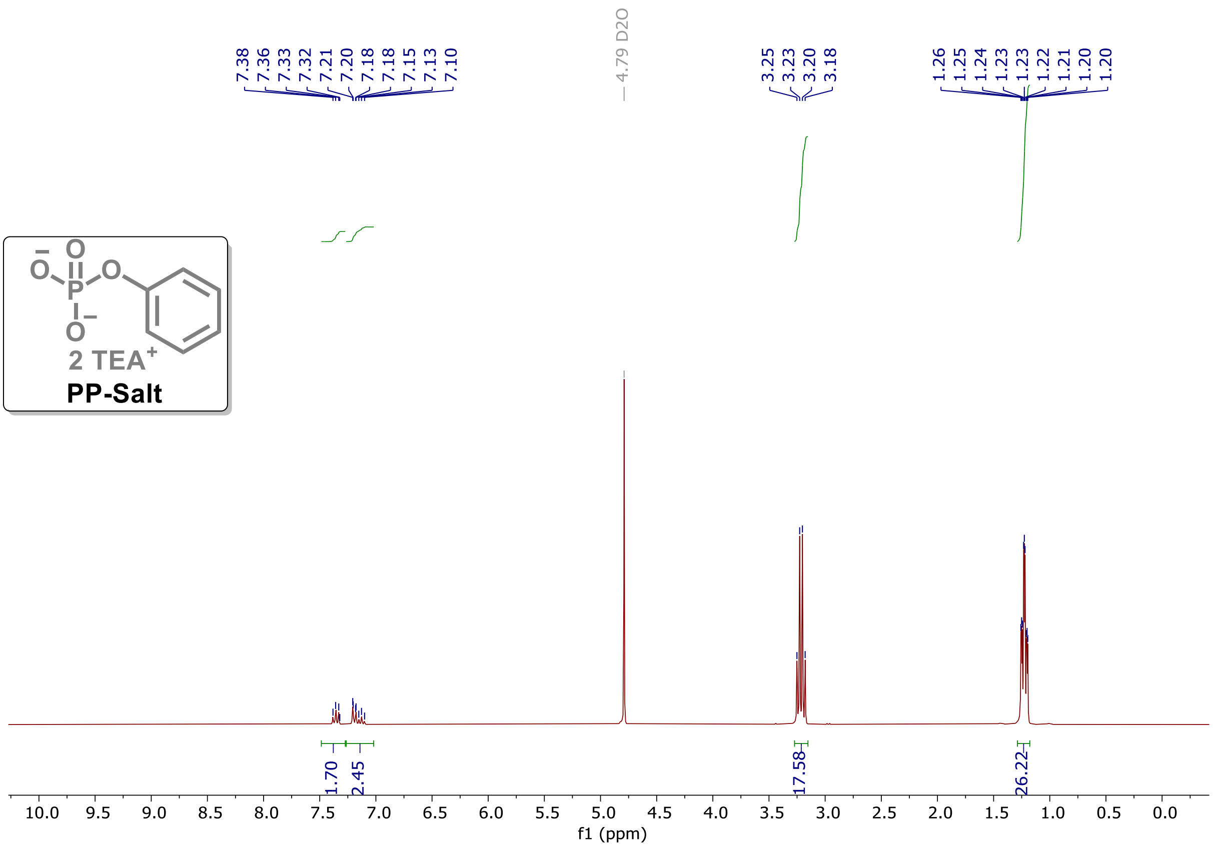

PP-Salt ^{31}P, 122 MHz, D_2O

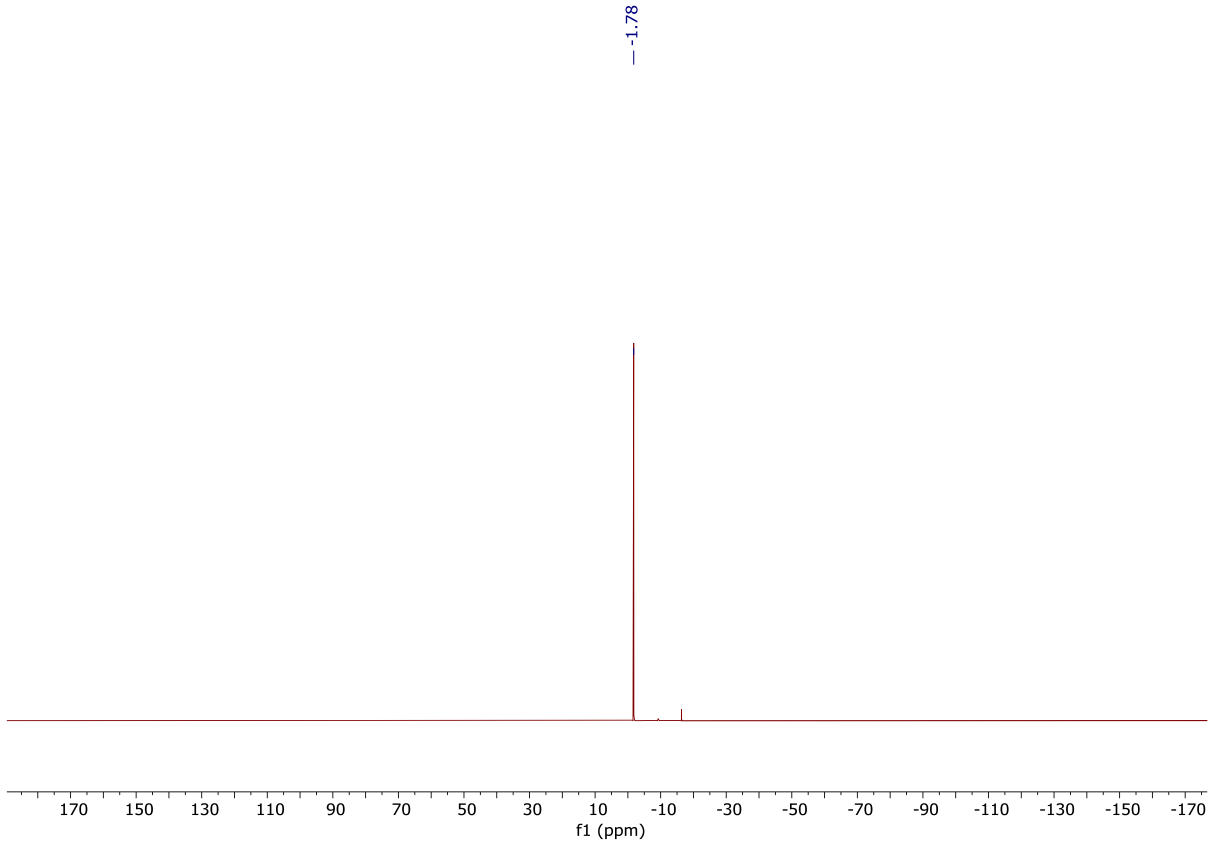

F(4-NH-Fmoc)EP ^{1}H, 300 MHz DMSO-d_6

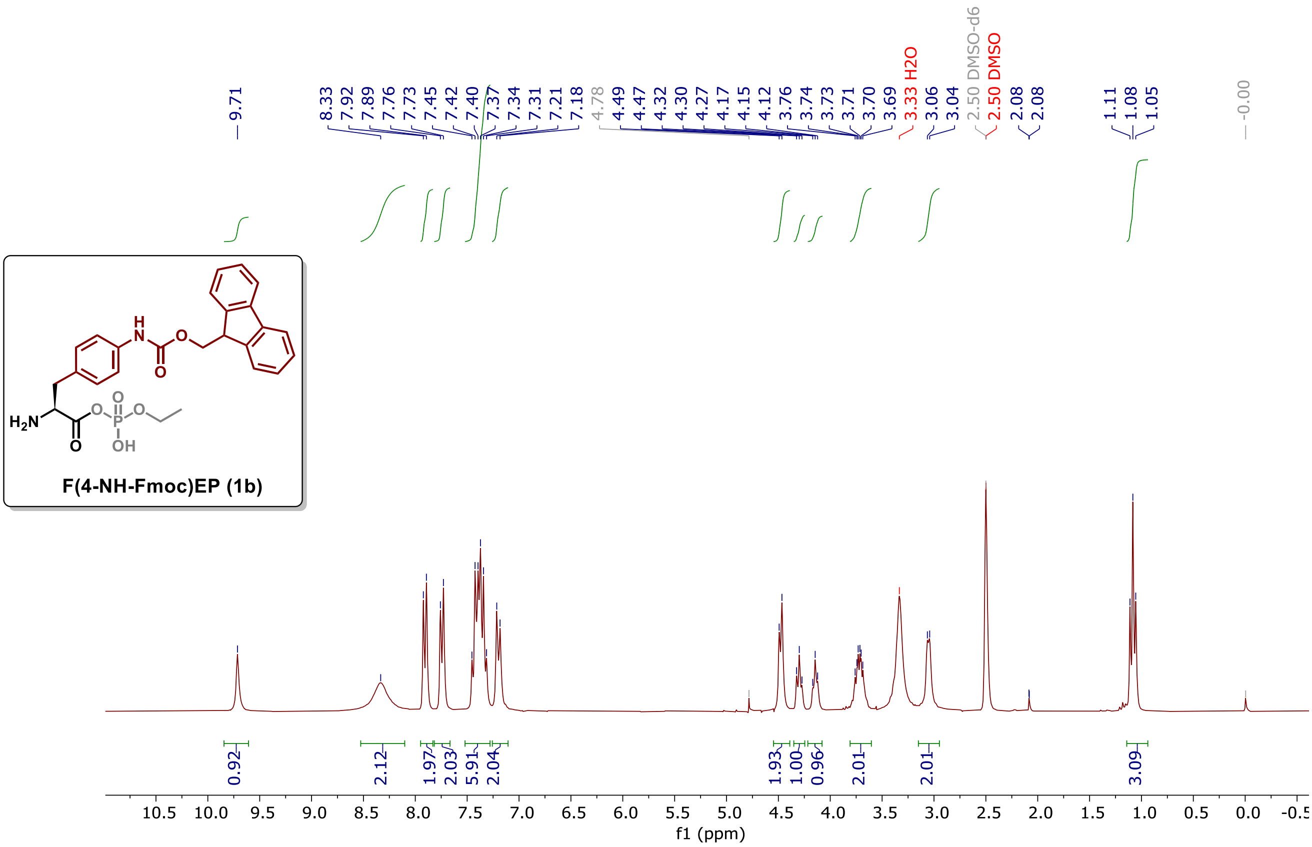

F(4-NH-Fmoc)EP ^{31}P, 122 MHz DMSO-d_6

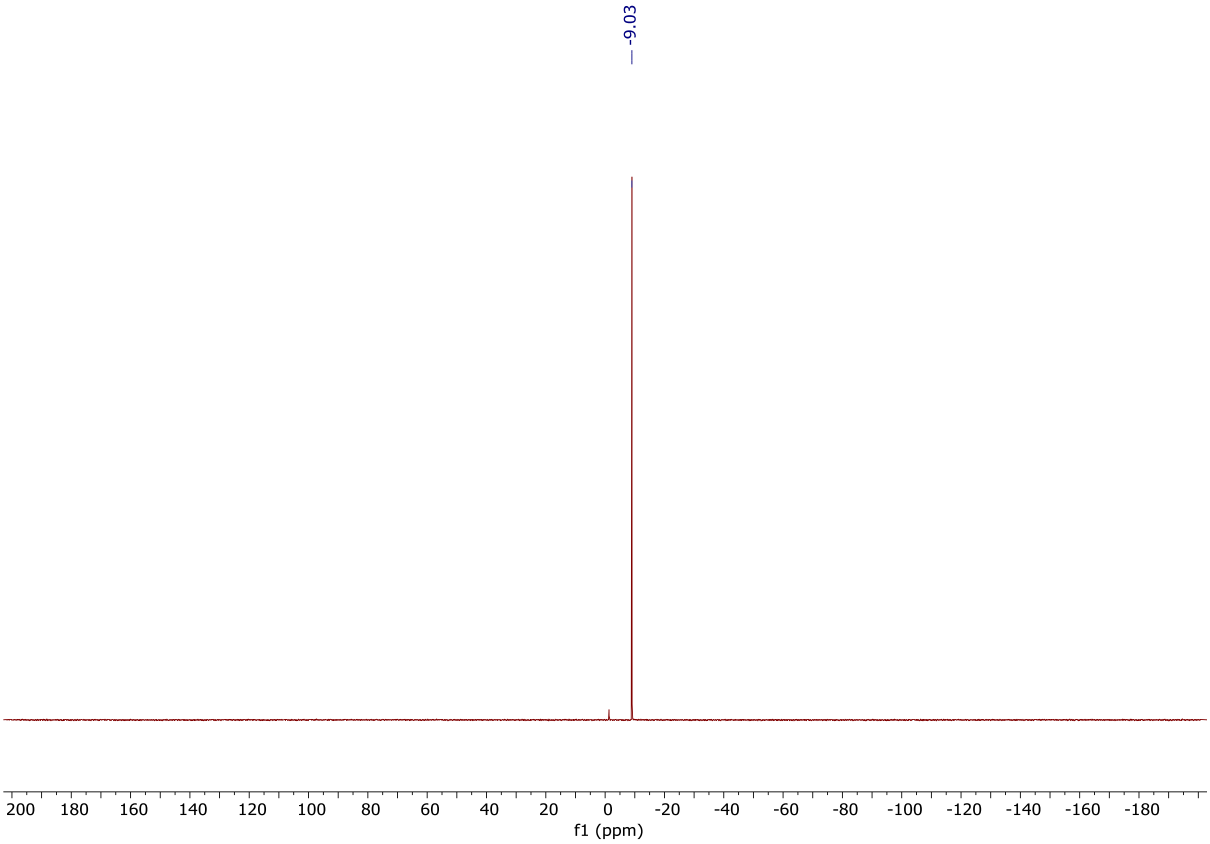

FEP ^{1}H, 400 MHz, D_2O

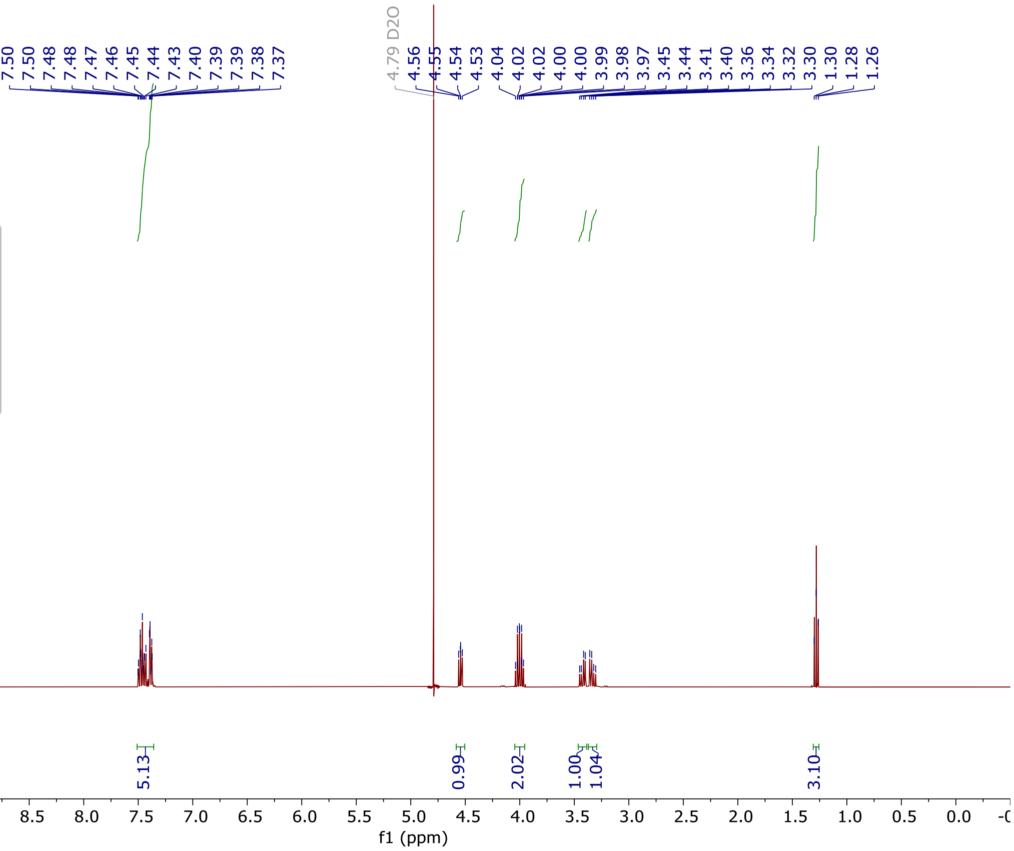

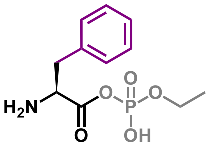

FEP (1a)

FEP ^{31}P, 162 MHz, D_2O

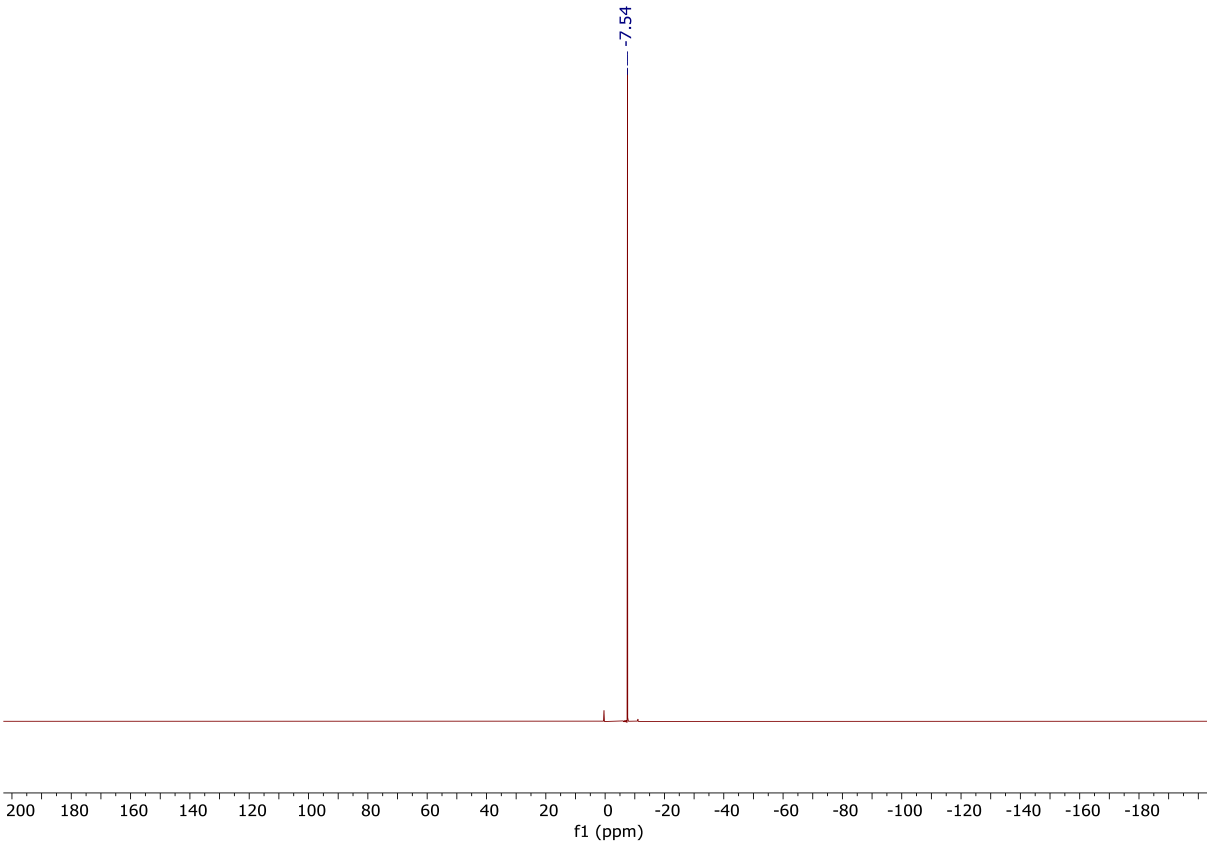

Boc-F(4-NH-Fmoc)EP ^{1}H, 400 MHz, $CDCl_3$

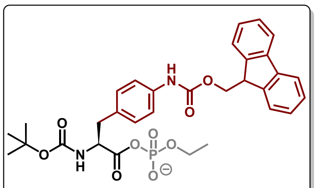

TEA salt

Boc-F(4-NH-Fmoc)EP (2b)

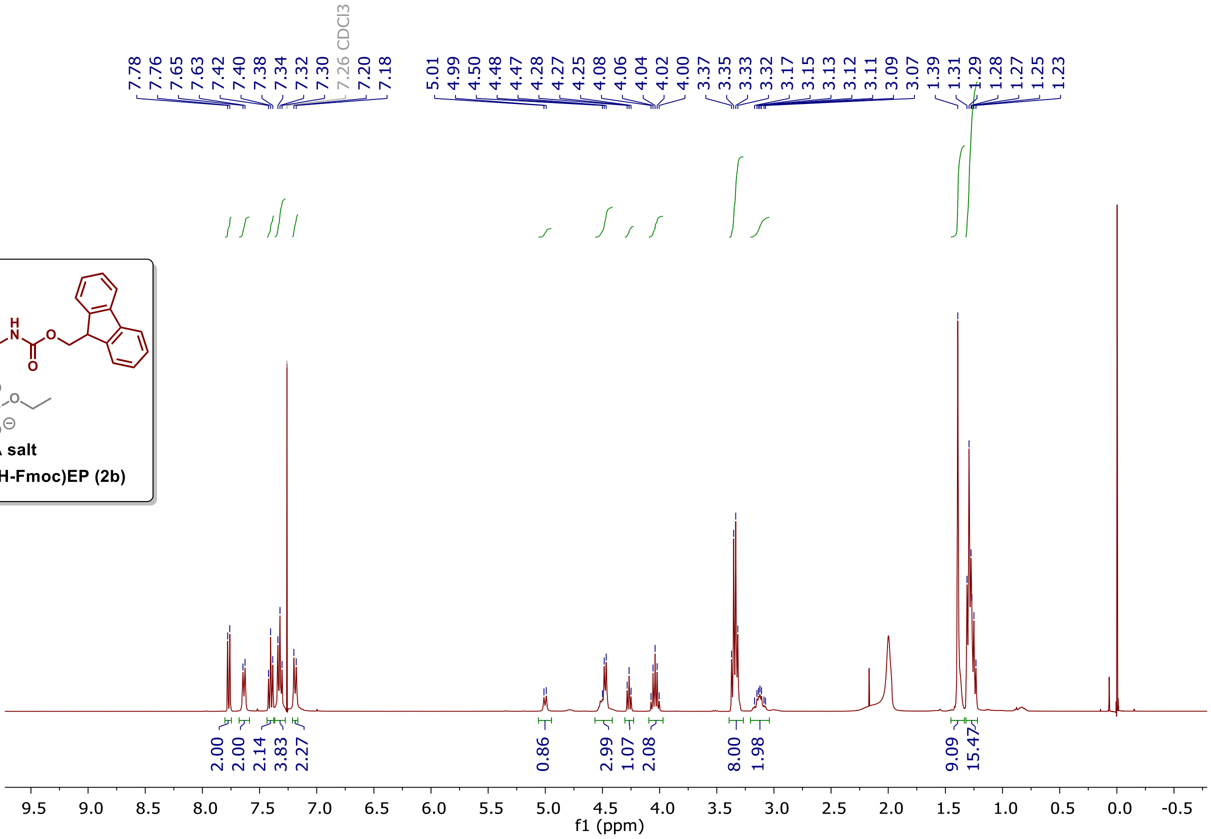

Boc-F(4-NH-Fmoc)EP ^{31}P, 162 MHz, $CDCl_3$

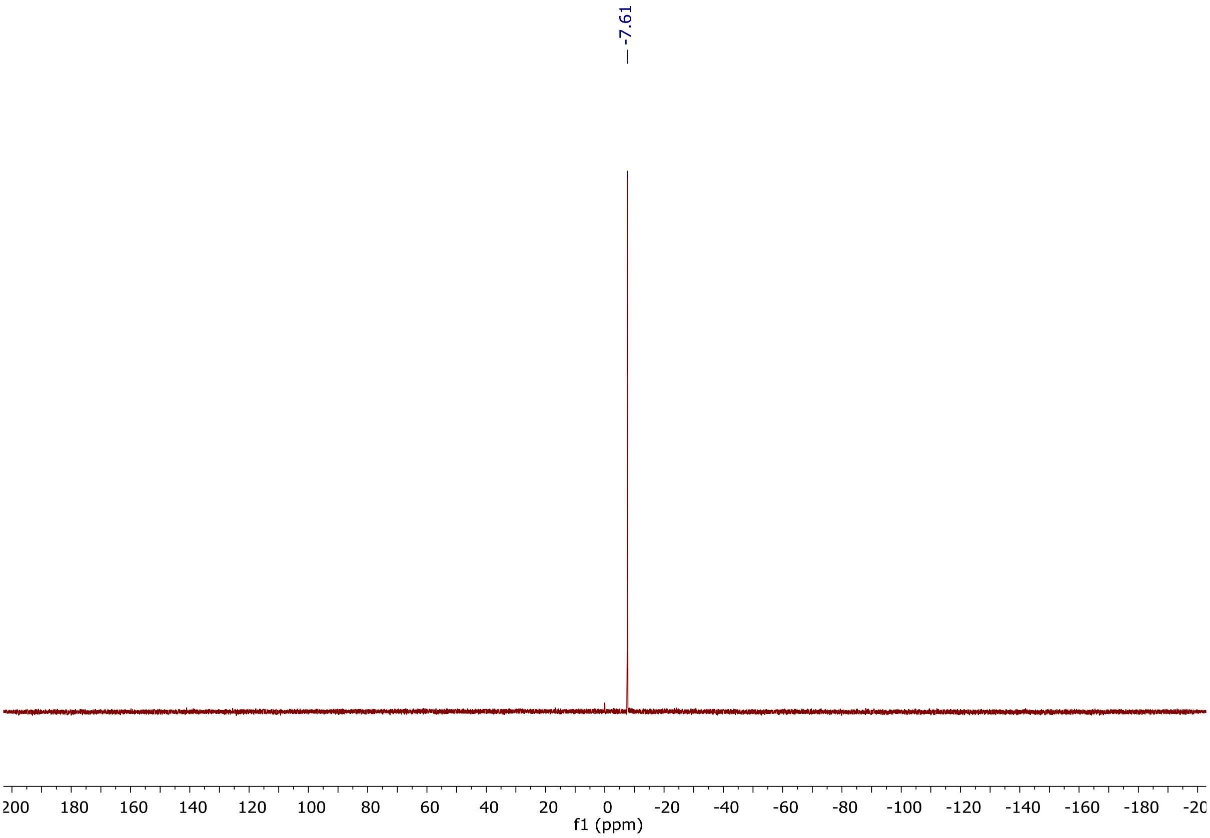

Boc-F(4-NH-Fmoc)PP ^{1}H, 400 MHz, $CDCl_3$

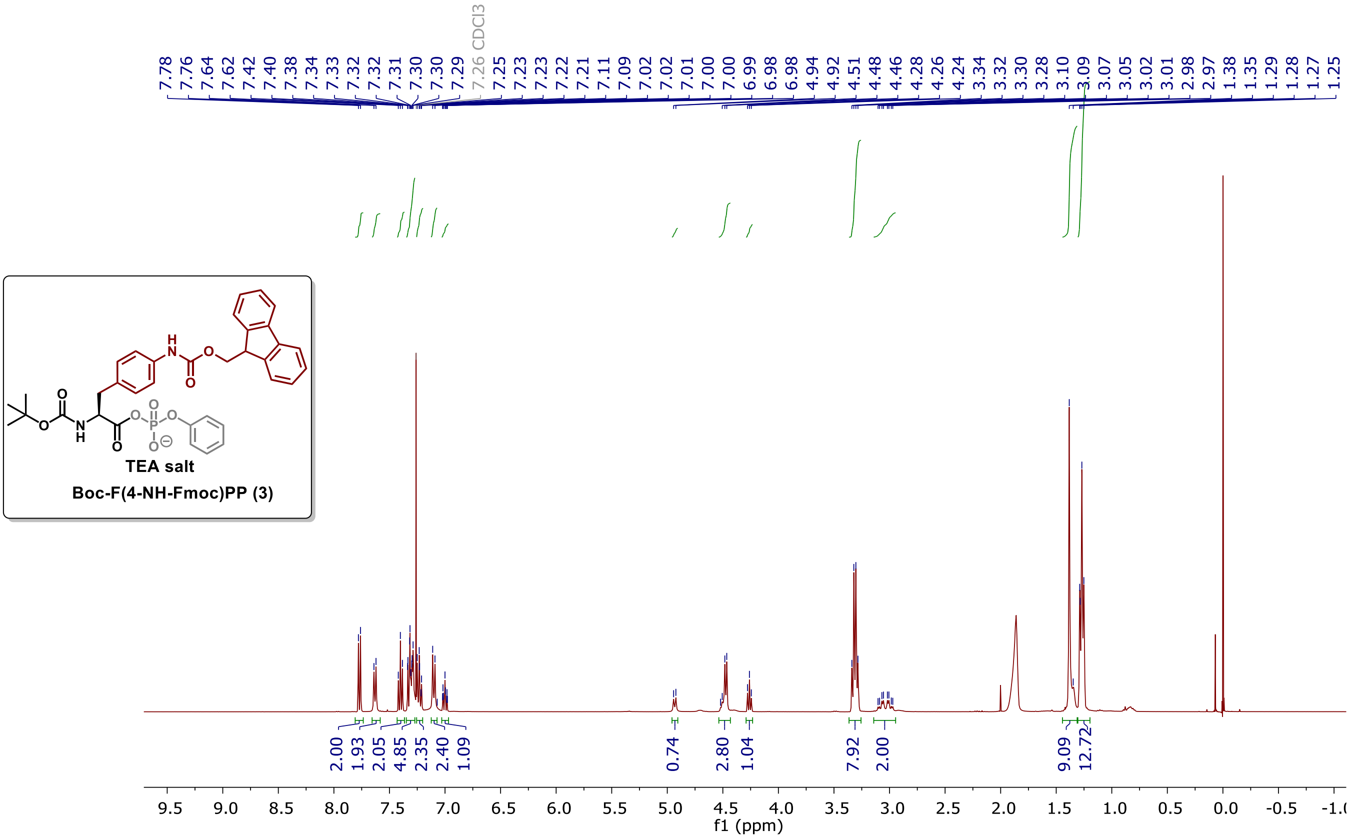

Boc-F(4-NH-Fmoc)PP ^{31}P, 162 MHz, $CDCl_3$

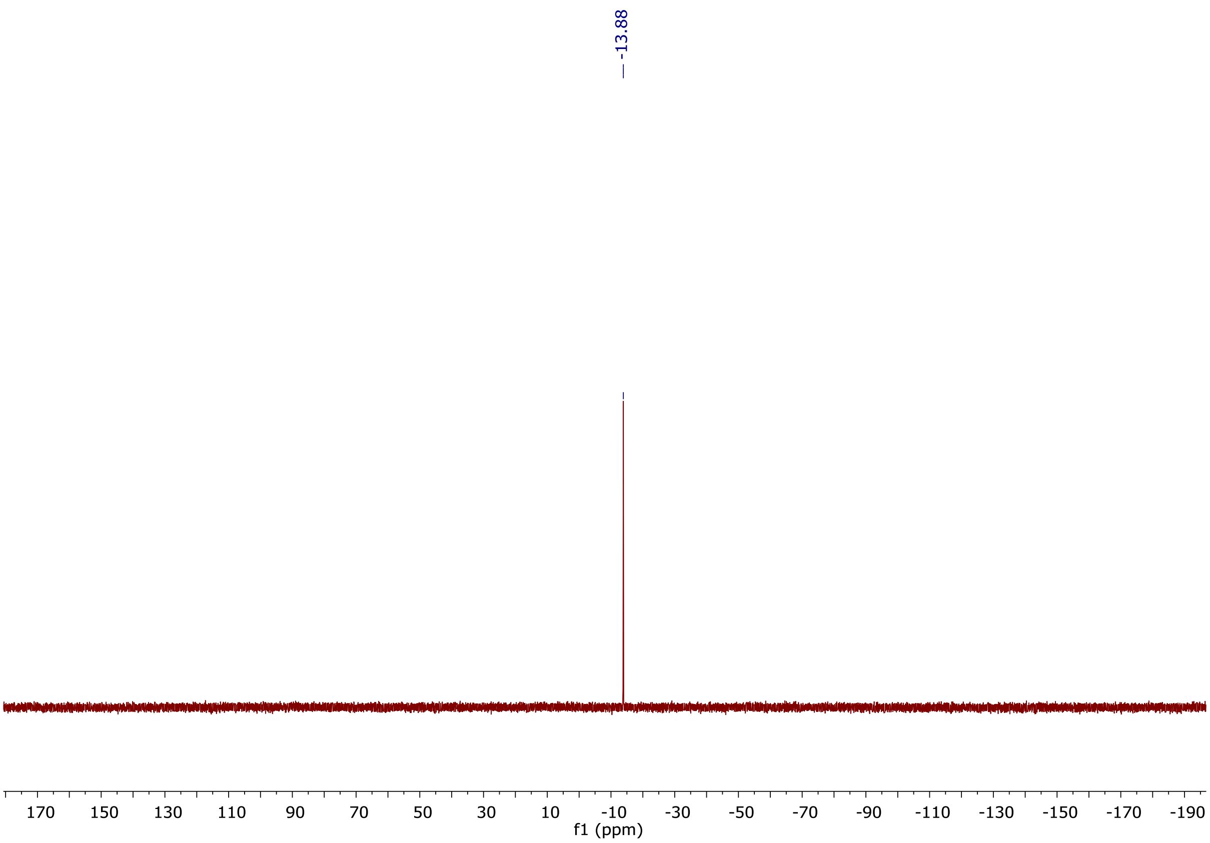

Boc-FEP ^{1}H, 300 MHz, $CDCl_3$

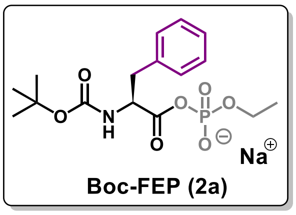

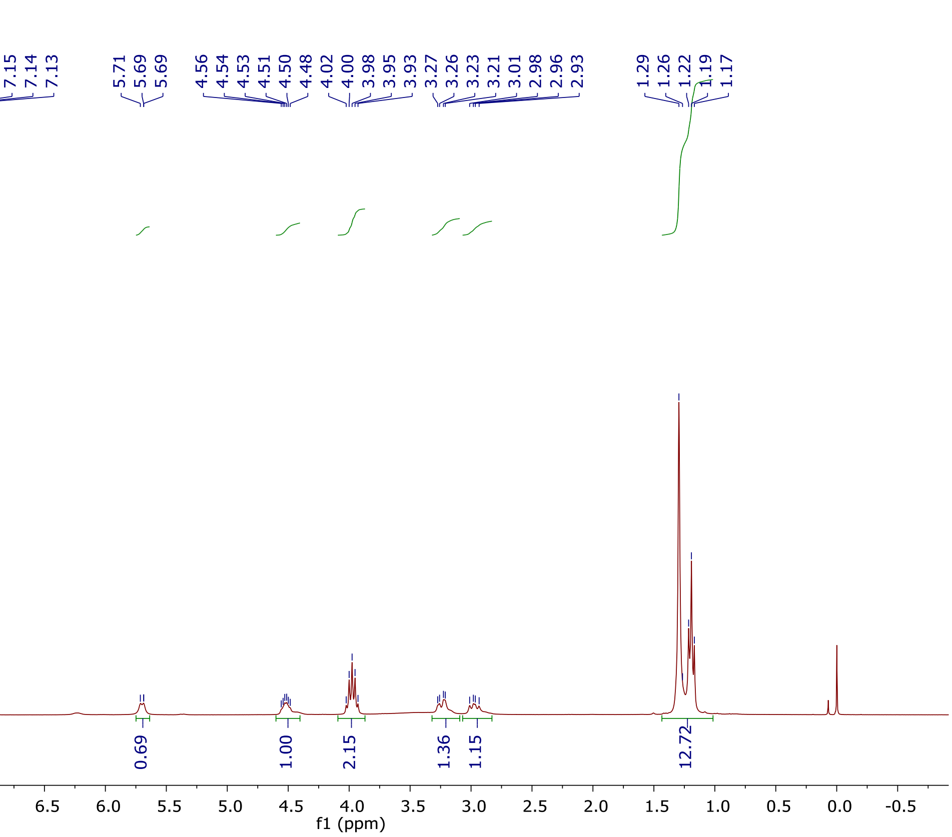

Boc-FEP ^{31}P, 122 MHz, $CDCl_3$

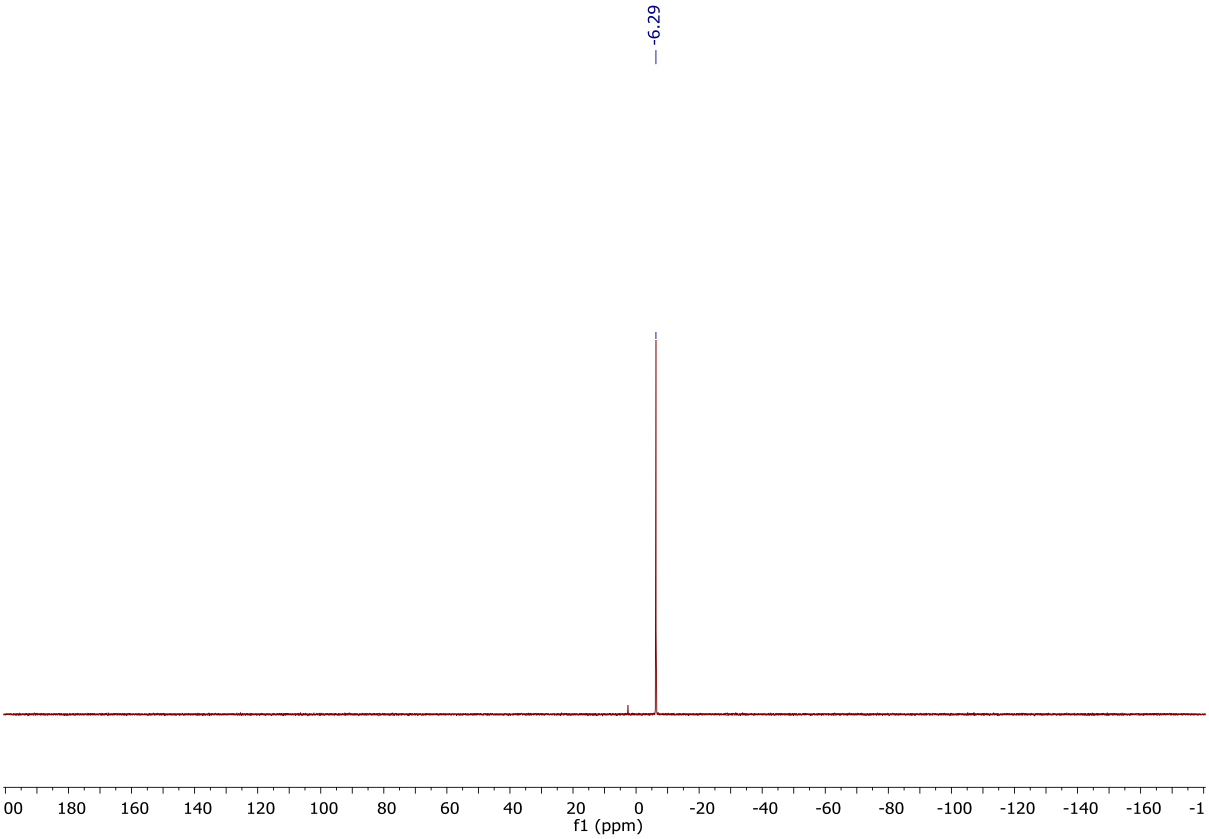

BPAEP ^{1}H, 300 MHz, DMSO-*d*$_6$

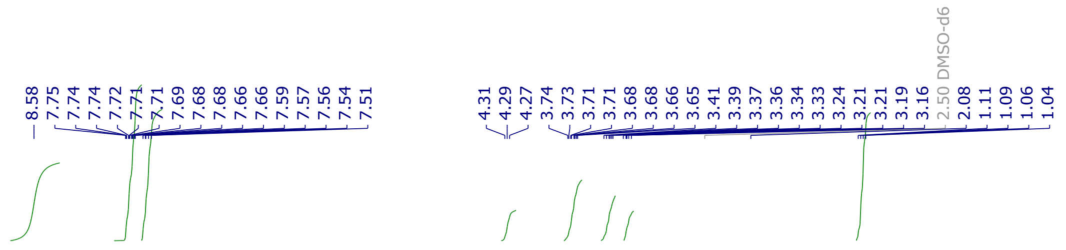

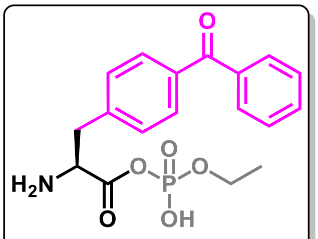

BPAEP (1f)

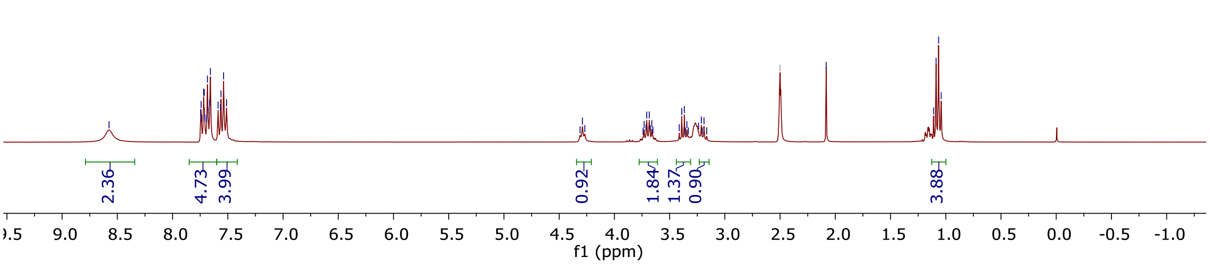

BPAEP ^{31}P, 122 MHz, DMSO-*d*$_6$

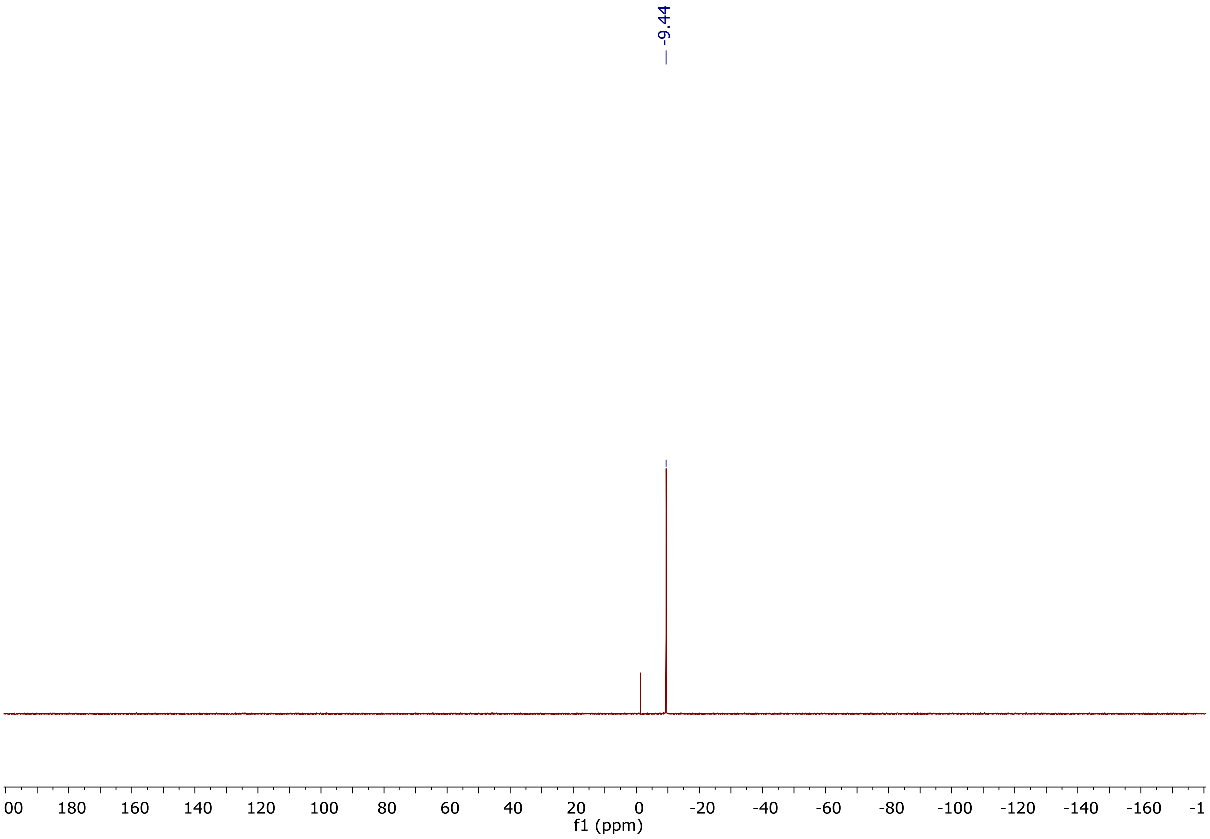

F(4-COOH)EP ^1H, 400 MHz, D_2O

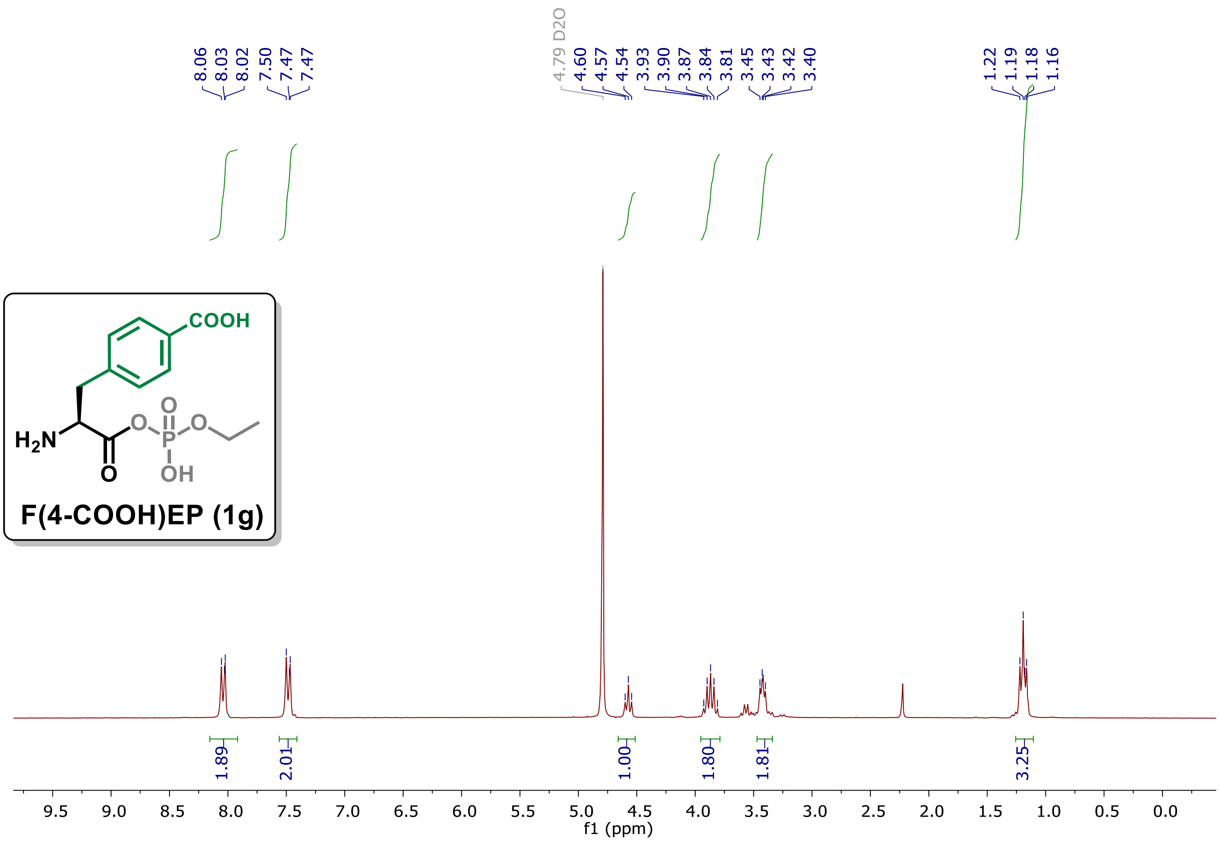

F(4-COOH)EP ^{31}P, 162 MHz, D_2O

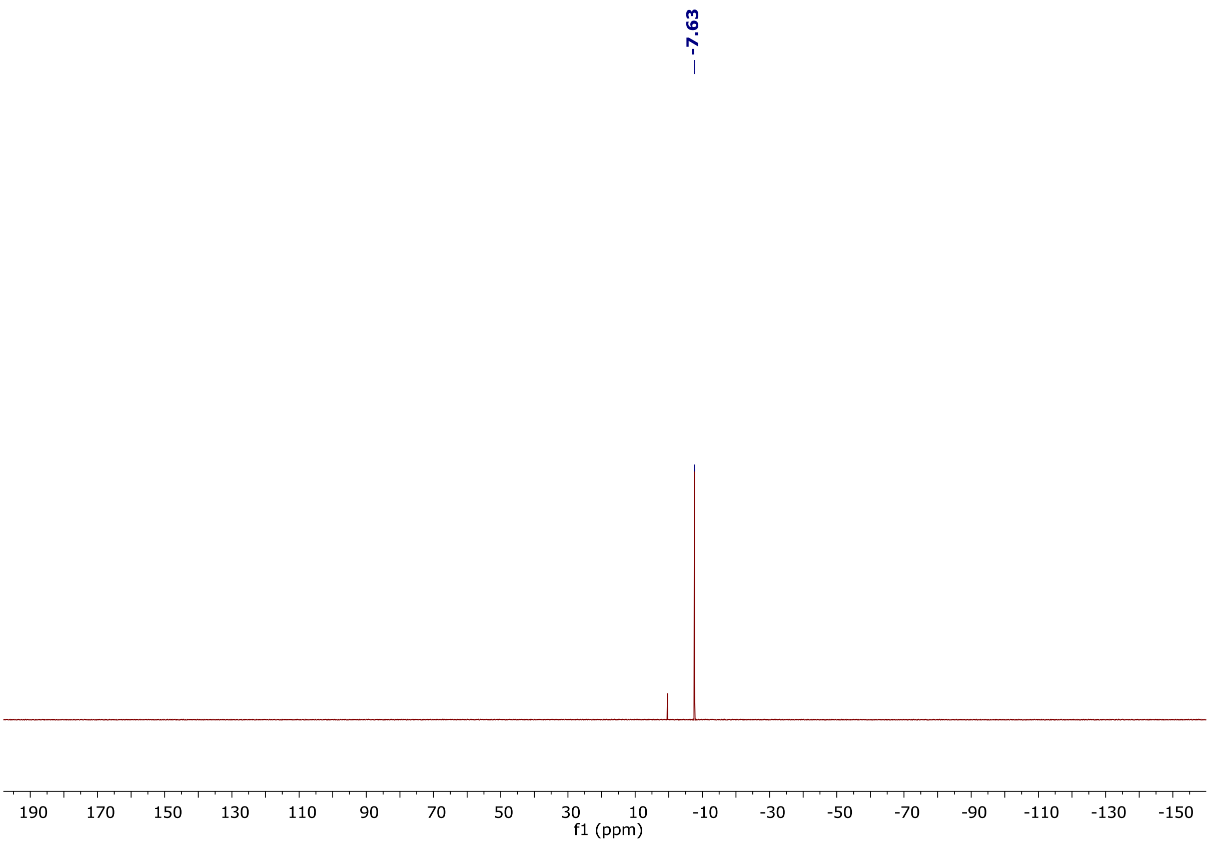

F-(4-gua)EP ^{1}H, 400 MHz, DMSO-d_6

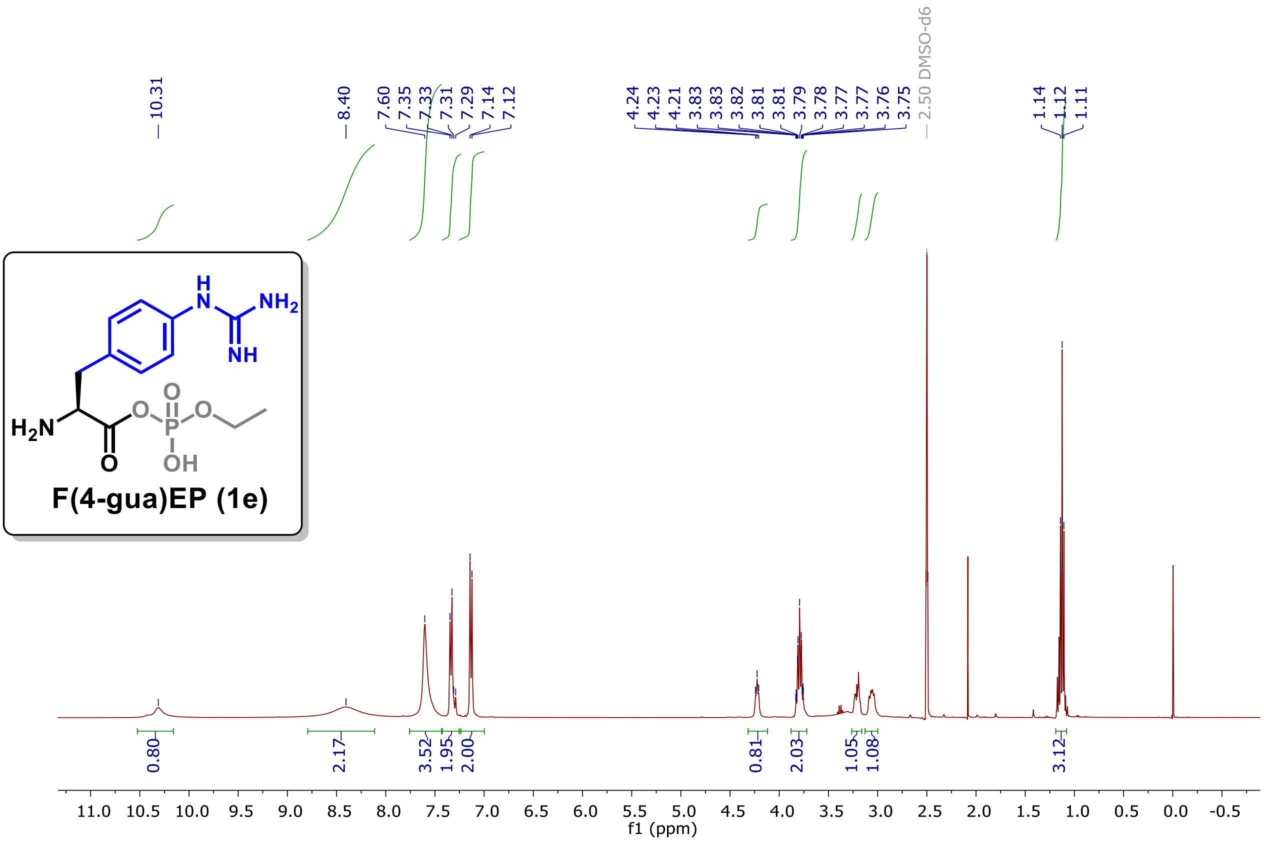

F-(4-gua)EP ^{31}P, 162 MHz, DMSO-d_6

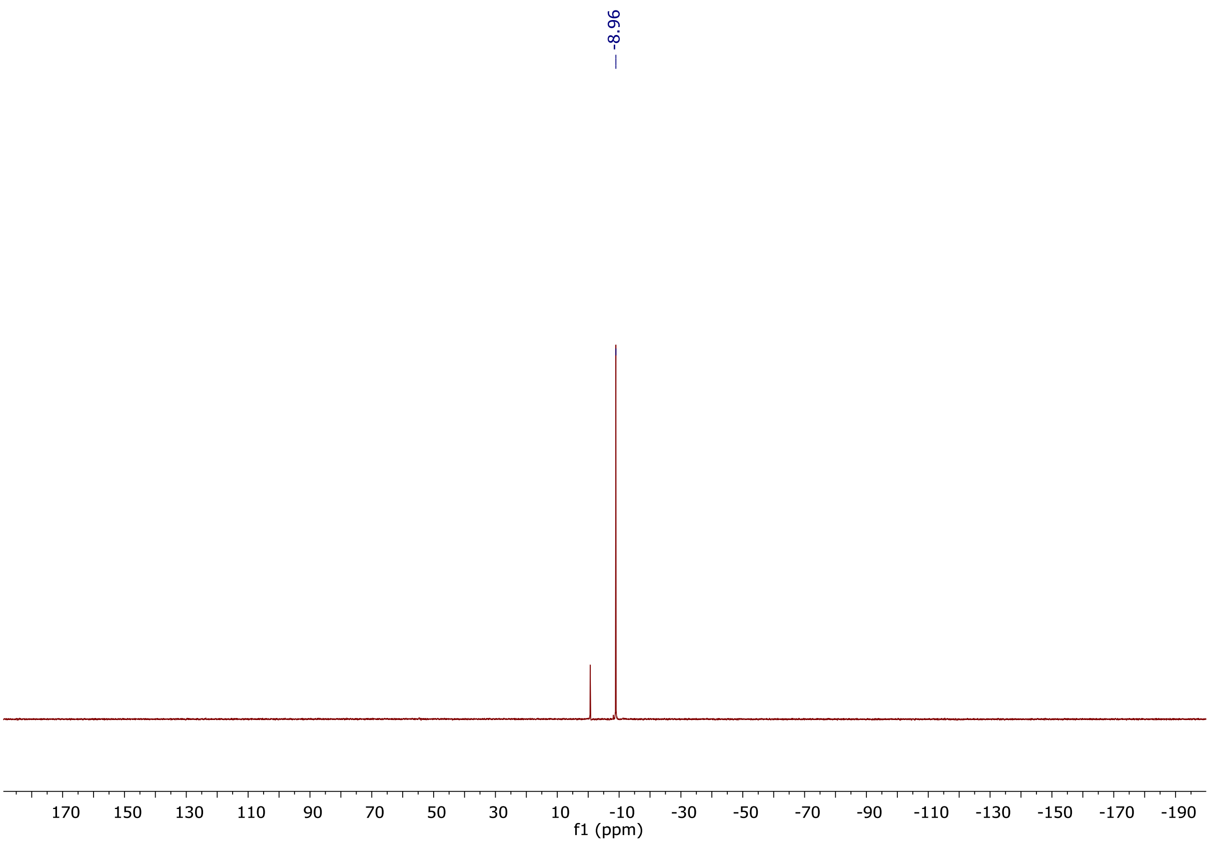

Boc-AEP ^{1}H, 400 MHz, D_2O

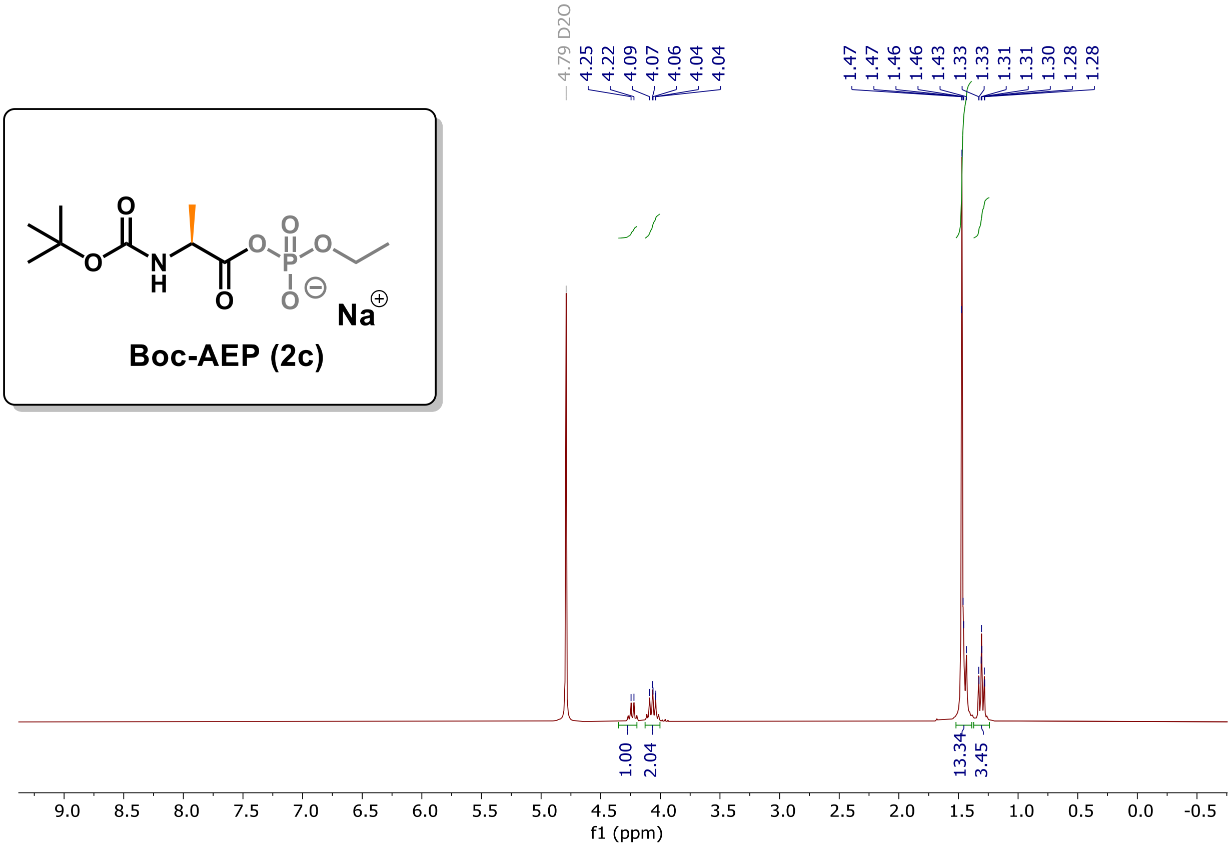

Boc-AEP (2c)

Boc-AEP ^{31}P, 162 MHz, D_2O

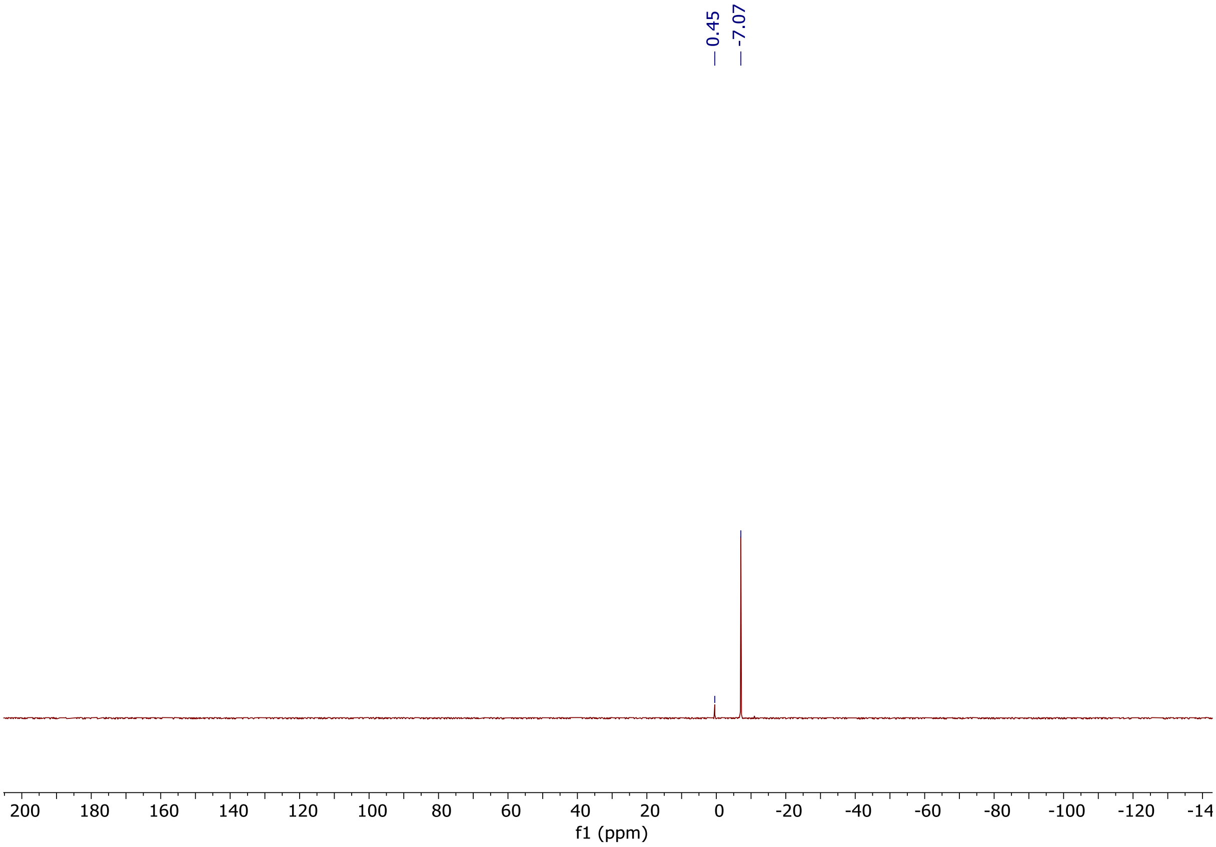

Boc-VEP ^{1}H, 400 MHz, D_2O

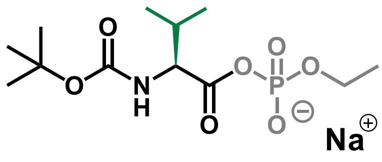

Boc-VEP (2d)

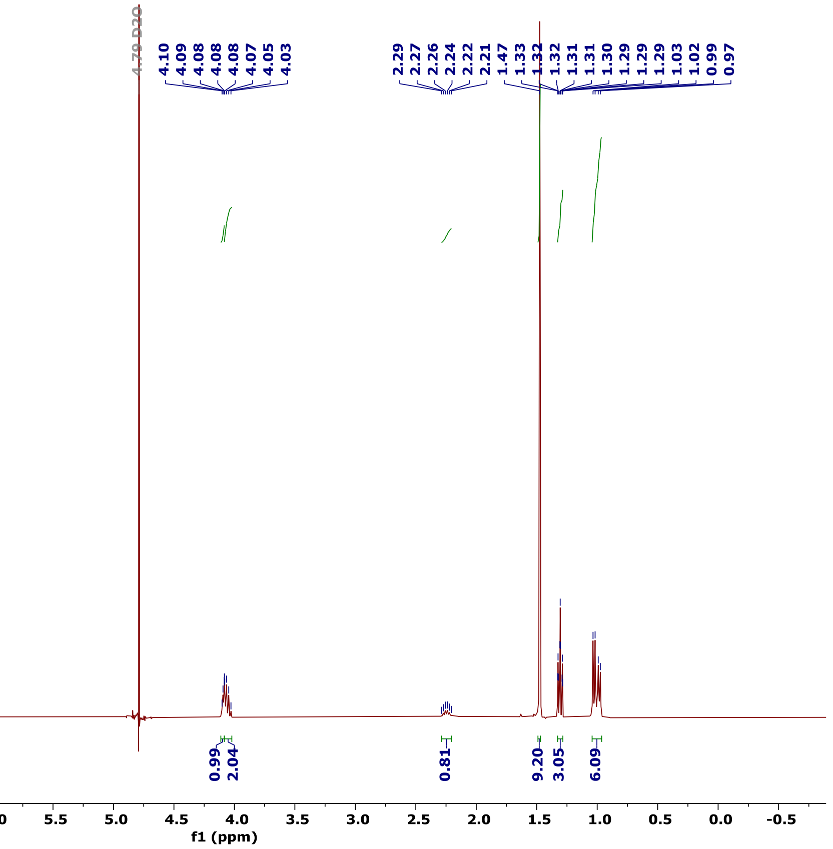

Boc-VEP ^{31}P, 162 MHz, D_2O

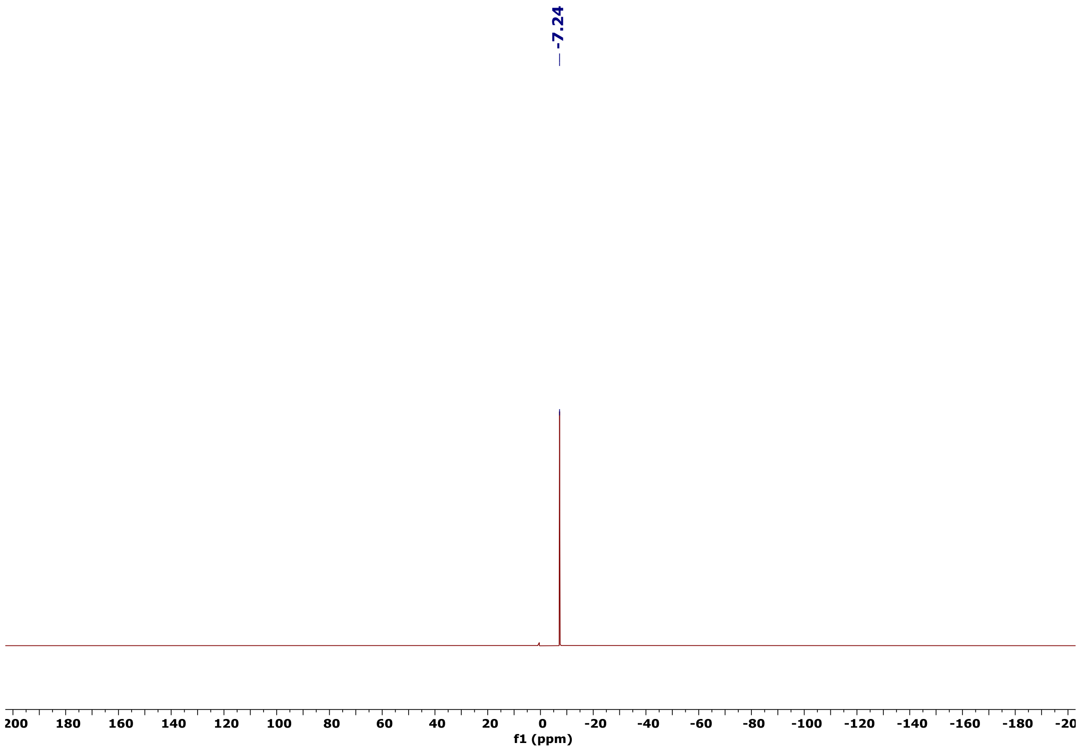

VEP ^1H, 400 MHz, D_2O

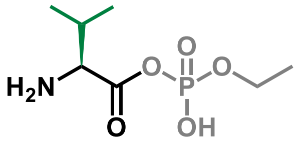

VEP (1d)

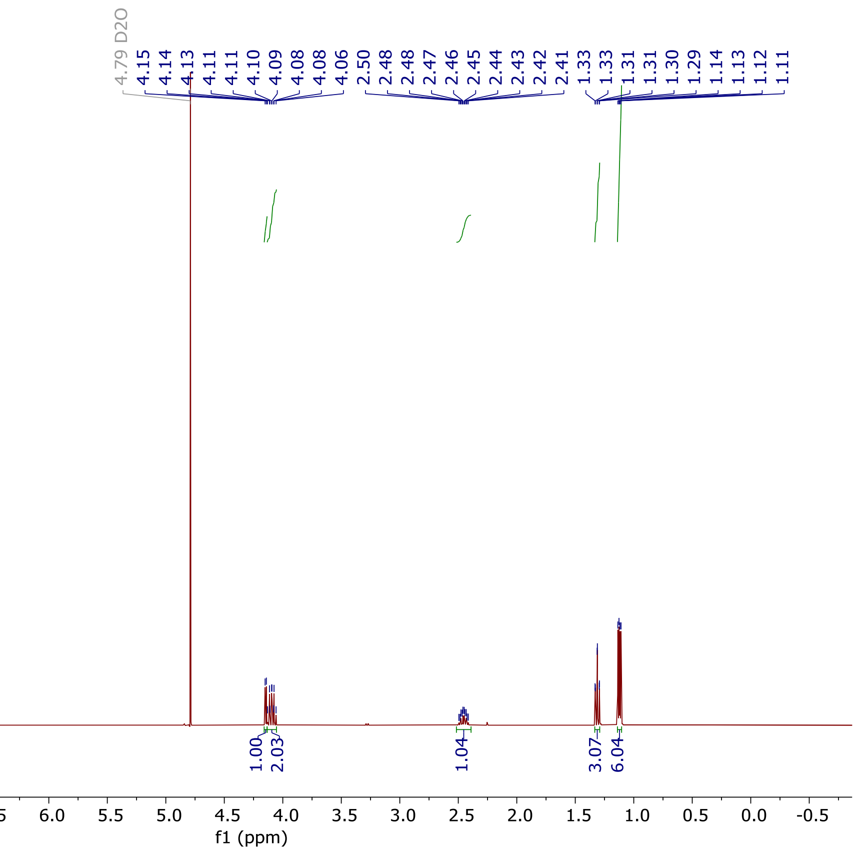

VEP ^{31}P, 162 MHz, D_2O

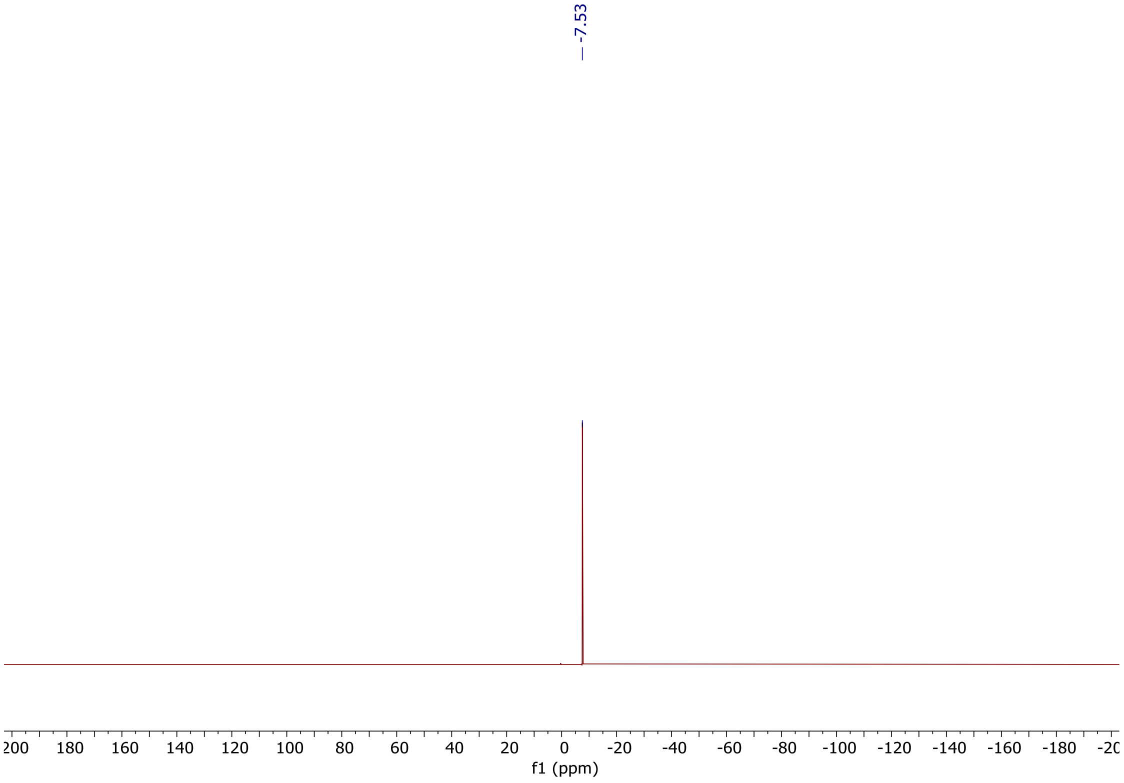

2.2.2 Characterization of Boc-APs by UPLC-MS

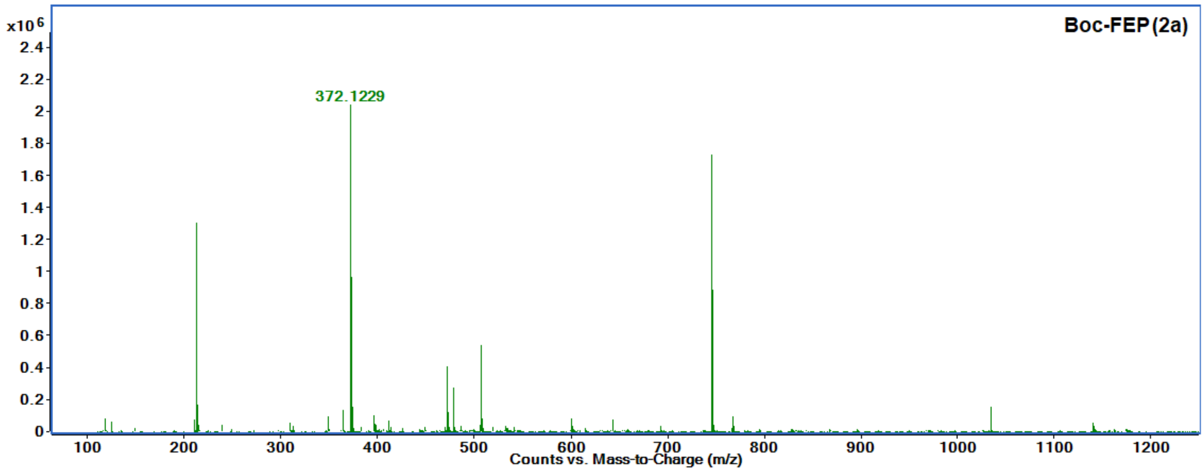

Mass spectra of **Boc-FEP (2a)**, obtained from the LC-MS analysis.

Calculated m/z [M-H]$^-$: 372.1218, Observed m/z [M-H]$^-$: 372.1229.

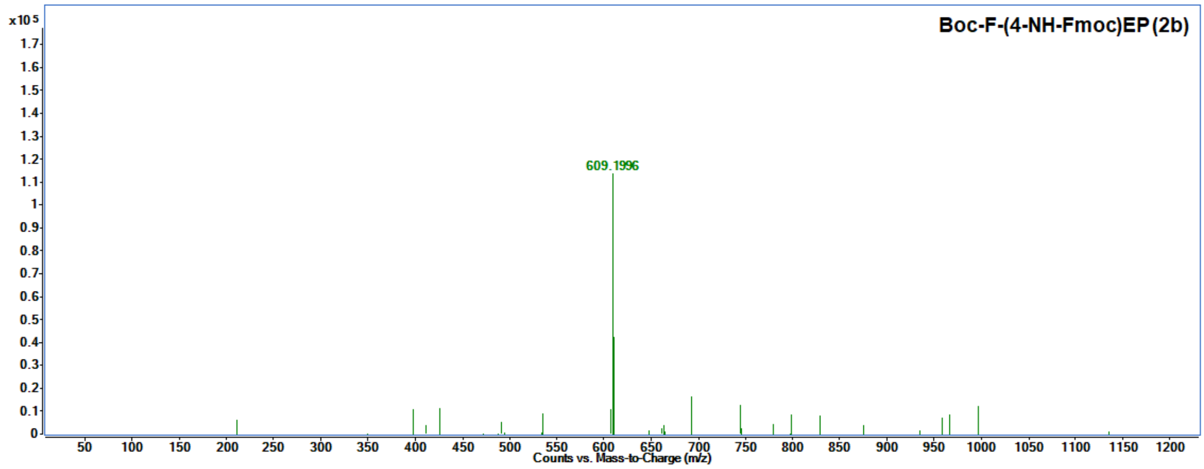

Mass spectra of **Boc-F(4-NH-Fmoc)EP (2b)**, obtained from the LC-MS analysis.

Calculated m/z [M-H]$^-$: 609.1996, Observed m/z [M-H]$^-$: 609.1996.

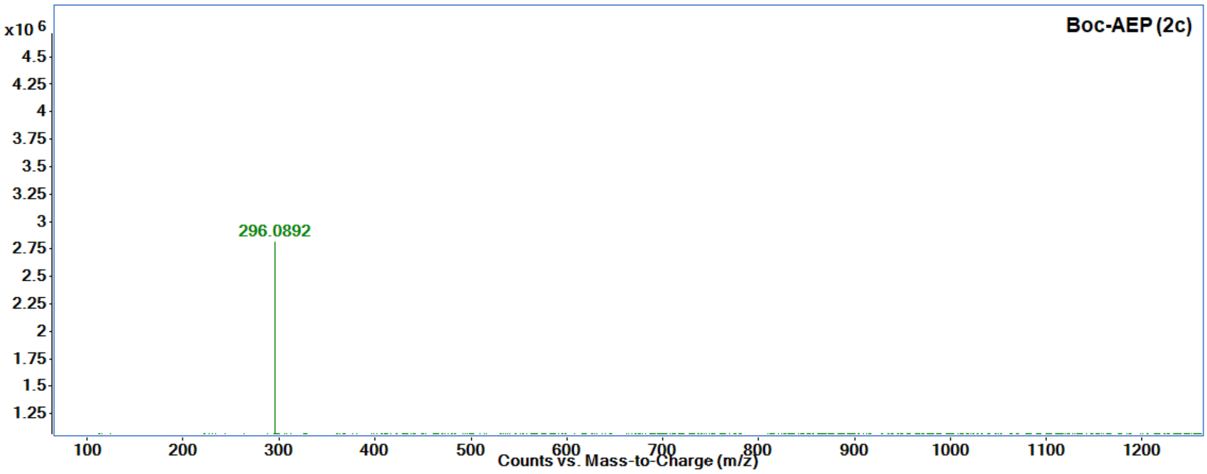

Mass spectra of **Boc-AEP (2c)**, obtained from the LC-MS analysis.

Calculated m/z $[M\text{-}H]^-$: 296.0905, Observed m/z $[M\text{-}H]^-$: 296.0892.

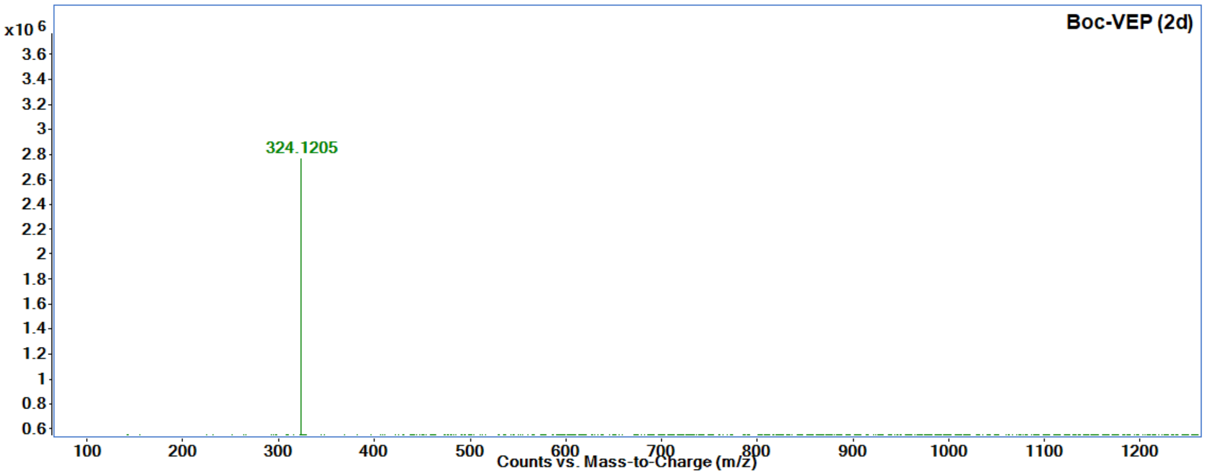

Mass spectra of **Boc-VEP (2d)**, obtained from the LC-MS analysis.

Calculated m/z $[M\text{-}H]^-$: 324.1218, Observed m/z $[M\text{-}H]^-$: 324.1205.

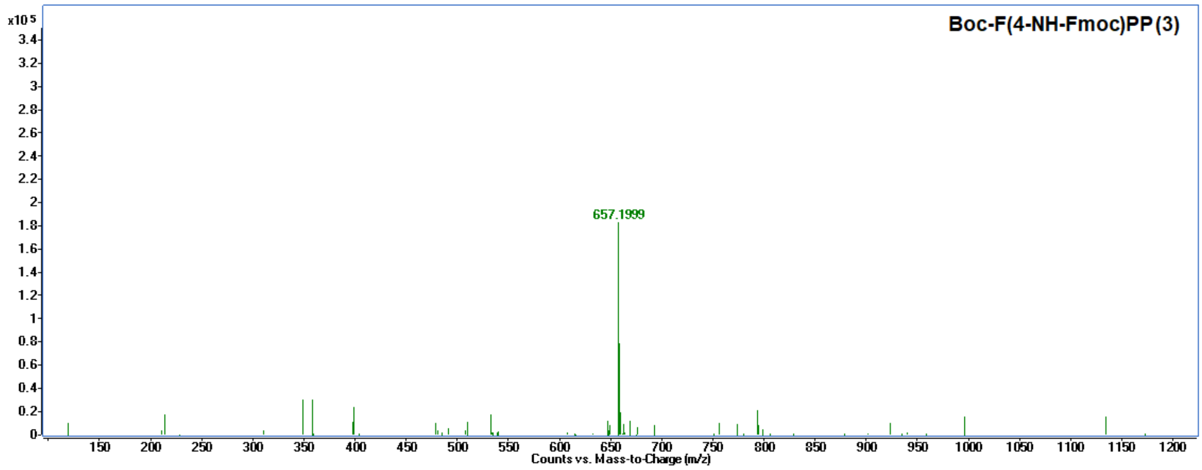

Mass spectra of **Boc-F(4-NH-Fmoc)PP (3)**, obtained from the LC-MS analysis.

Calculated m/z $[M-H]^-$: 657.2007, Observed m/z $[M-H]^-$: 657.1999.

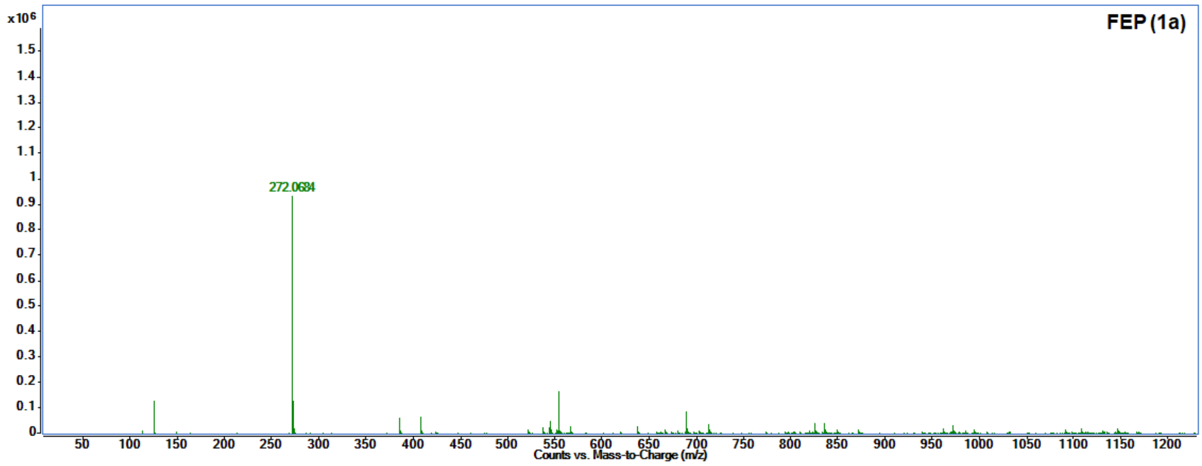

Mass spectra of **FEP (1a)**, obtained from the LC-MS analysis.

Calculated m/z $[M-H]^-$: 272.0682, Observed m/z $[M-H]^-$: 272.0684.

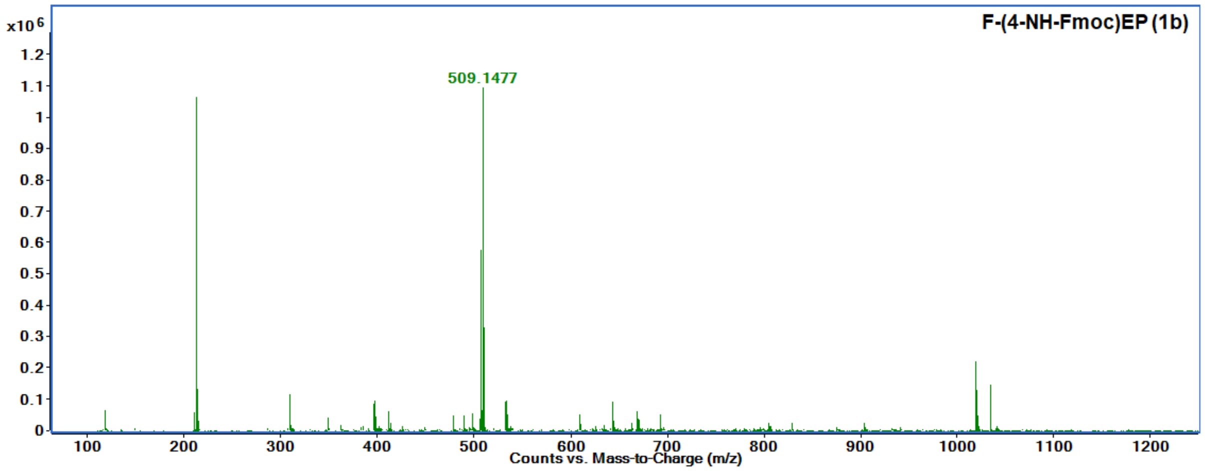

Mass spectra of **F(4-NH-Fmoc)EP (1b)**, obtained from the LC-MS analysis.

Calculated m/z $[M-H]^-$: 509.1483, Observed m/z $[M-H]^-$: 509.1477.

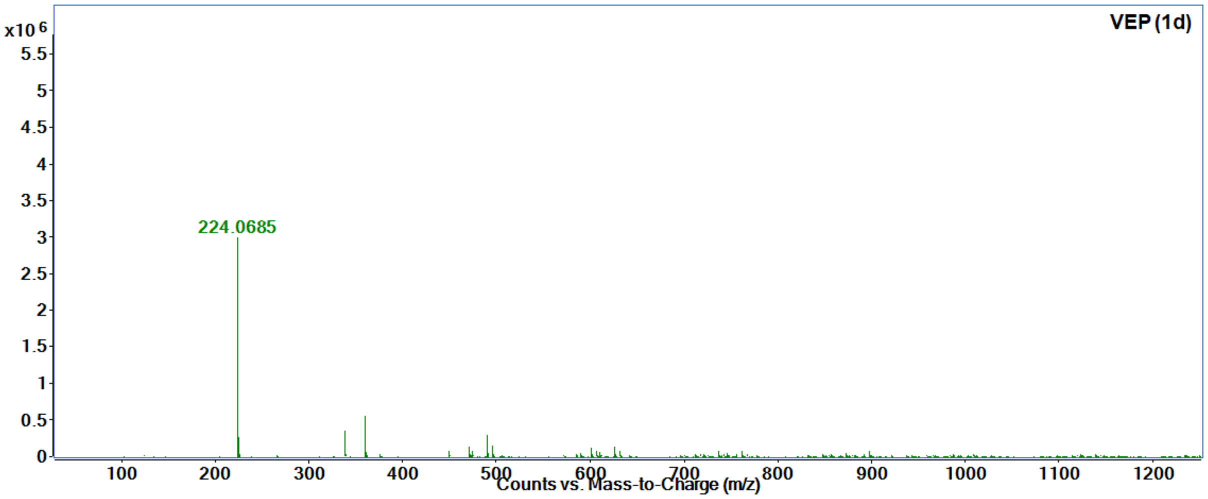

Mass spectra of **VEP (1d)**, obtained from the LC-MS analysis.

Calculated m/z $[M-H]^-$: 224.0693, Observed m/z $[M-H]^-$: 224.0685.

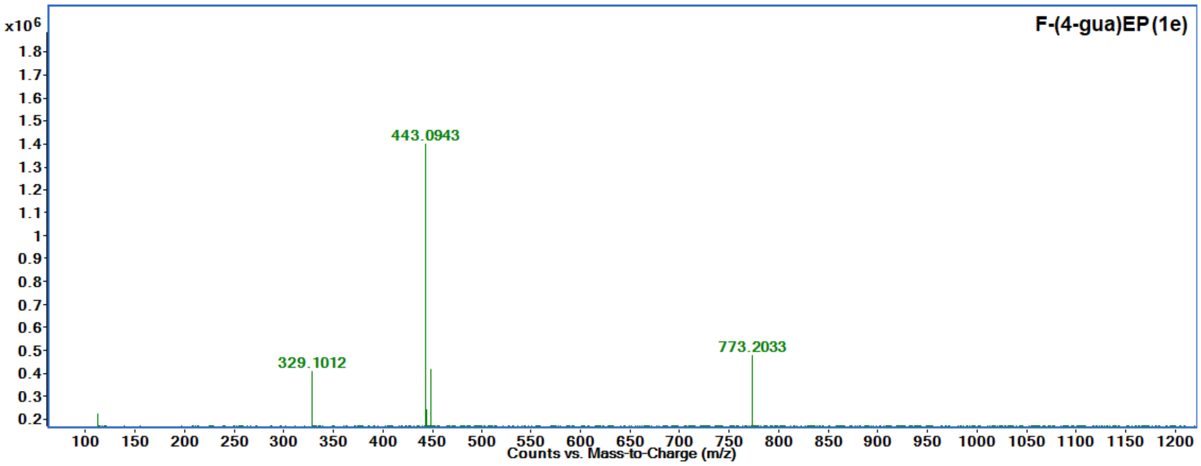

Mass spectra of **F(4-gua)EP (1e)**, obtained from the LC-MS analysis.

Calculated m/z $[M-H]^-$: 329.1012, Observed m/z $[M-H]^-$: 329.1012.

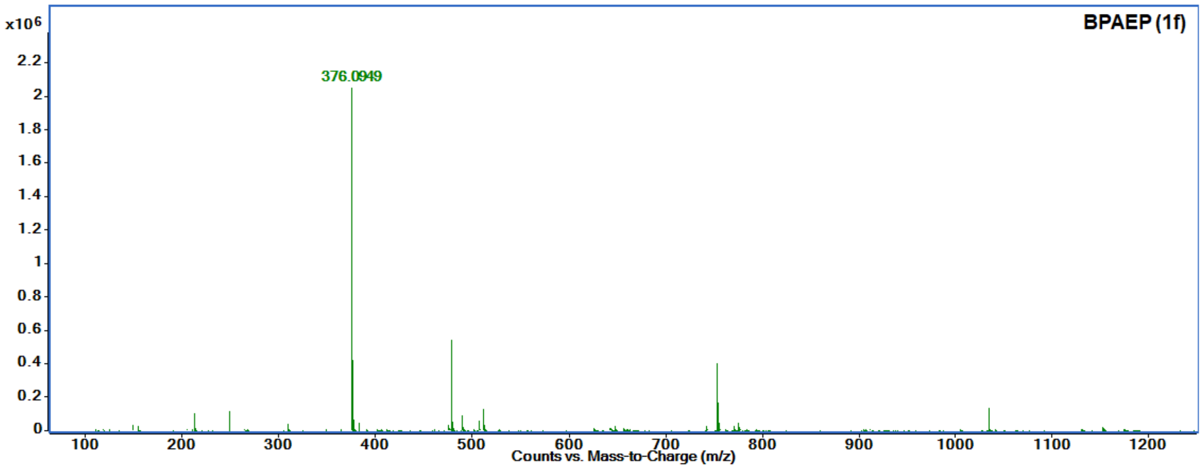

Mass spectra of **BPAEP (1f)**, obtained from the LC-MS analysis.

Calculated m/z $[M-H]^-$: 376.0955, Observed m/z $[M-H]^-$: 376.0949.

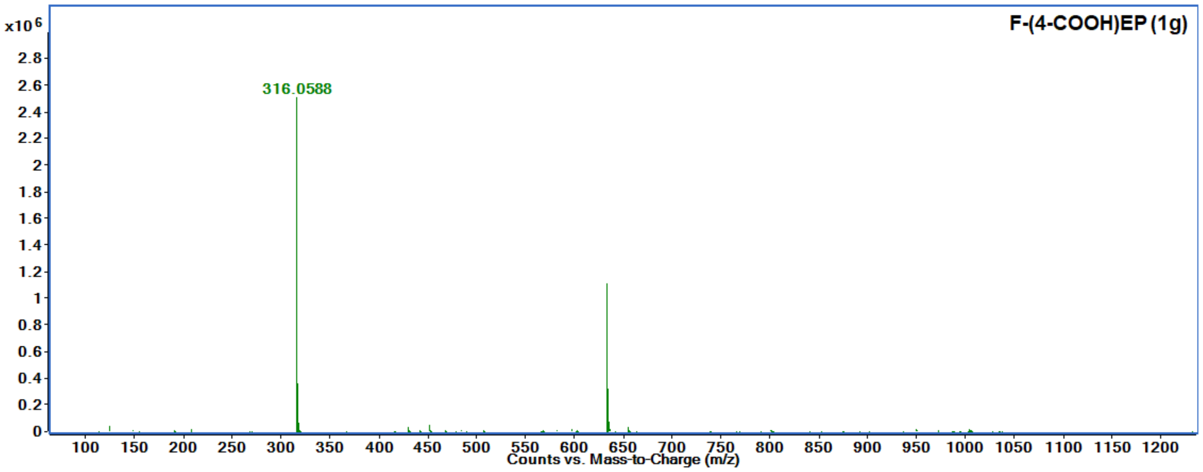

Mass spectra of **F-(4-COOH)EP (1g)**, obtained from the LC-MS analysis.

Calculated m/z $[M-H]^-$:316.0592, Observed m/z $[M-H]^-$: 316.0588.

3. Supplementary Figures:

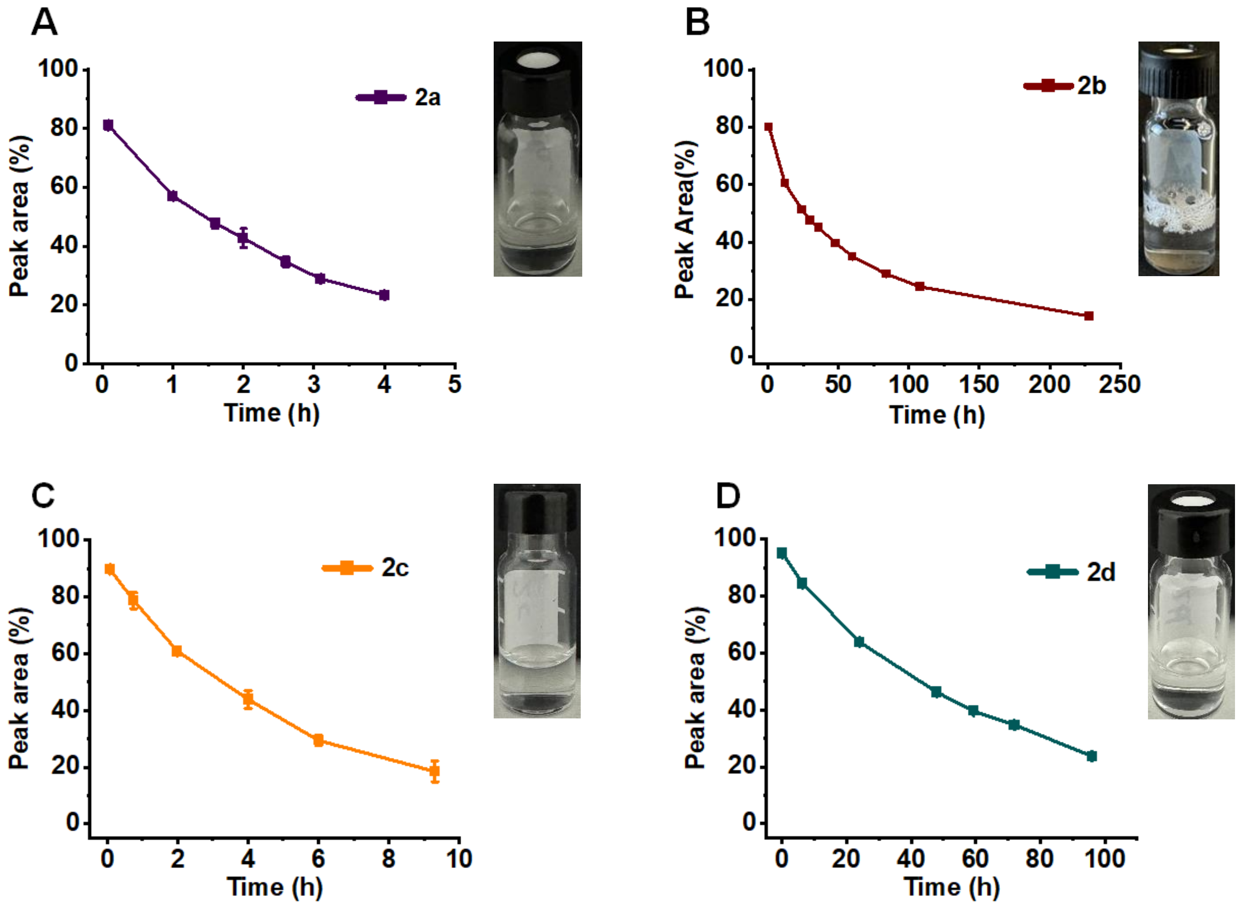

Supplementary Figure 1: Hydrolysis of 10 mM A) **2a**, B) **2b,** C) **2c** and D) **2d** in 0.6 M borate buffer, pH 9.1. The half-life of **2a, 2b, 2c** and **2d** is 2.17, 99, 4 and 49.5 hours respectively. The half-lives are calculated by using the equation: $t_{1/2} = 0.693/k$, assuming first-order kinetics. Error bars represent the standard deviation of three independent experiments. Inset photos represent the macroscopic behavior of the samples throughout the reactions.

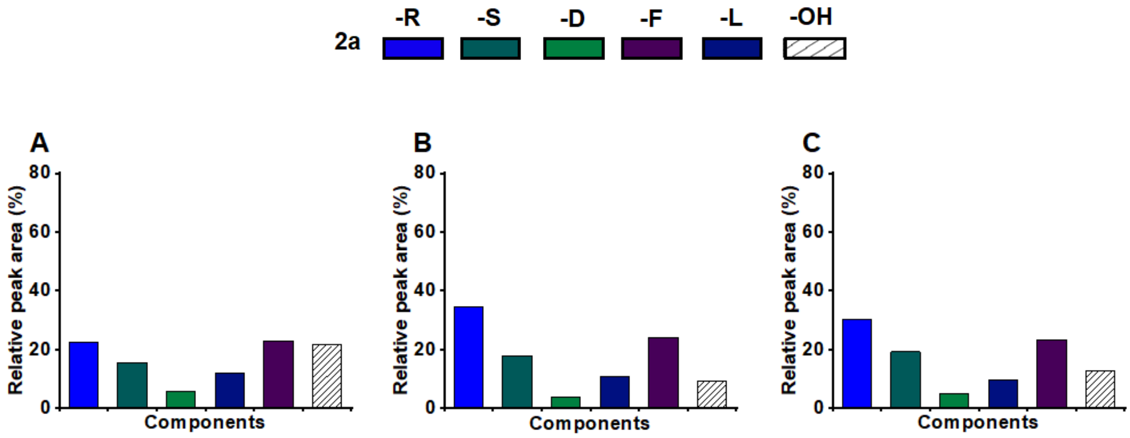

Supplementary Figure 2: Bar graphs showing peptide conversion between 10 mM **2a** and A) amino acid mixture I (10 mM D+S+L+R+F) at a 1:1 ratio in borate buffer (pH 9.1, 0.6 M), B) Amino acid mixture I (50 mM D+S+L+R+F) at a 1:5 ratio in PBS buffer (pH 8.1, 0.1 M) and C) Amino acid mixture I (50 mM D+S+L+R+F) at a 1:5 ratio in PBS buffer (pH 7.5, 0.1 M). In each bar graph, striped bars represent the hydrolysis product **2a-OH**. Peptide coupling yields are measured after 2 hours of reaction time.

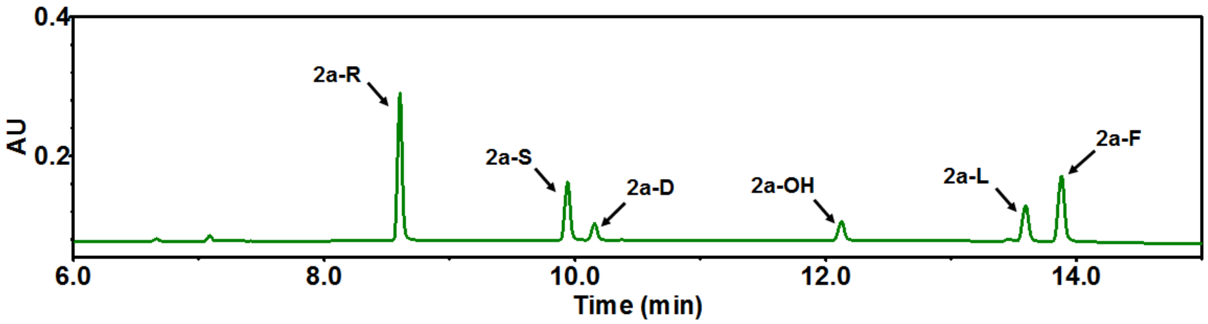

Supplementary Figure 3: UPLC chromatogram of reaction between 10 mM **2a** and 50 mM amino acid mixture I (D, S, L, R, and F, each at 10 mM) in 0.6 M borate buffer, pH 9.1. Measurements were taken after 30 minutes.

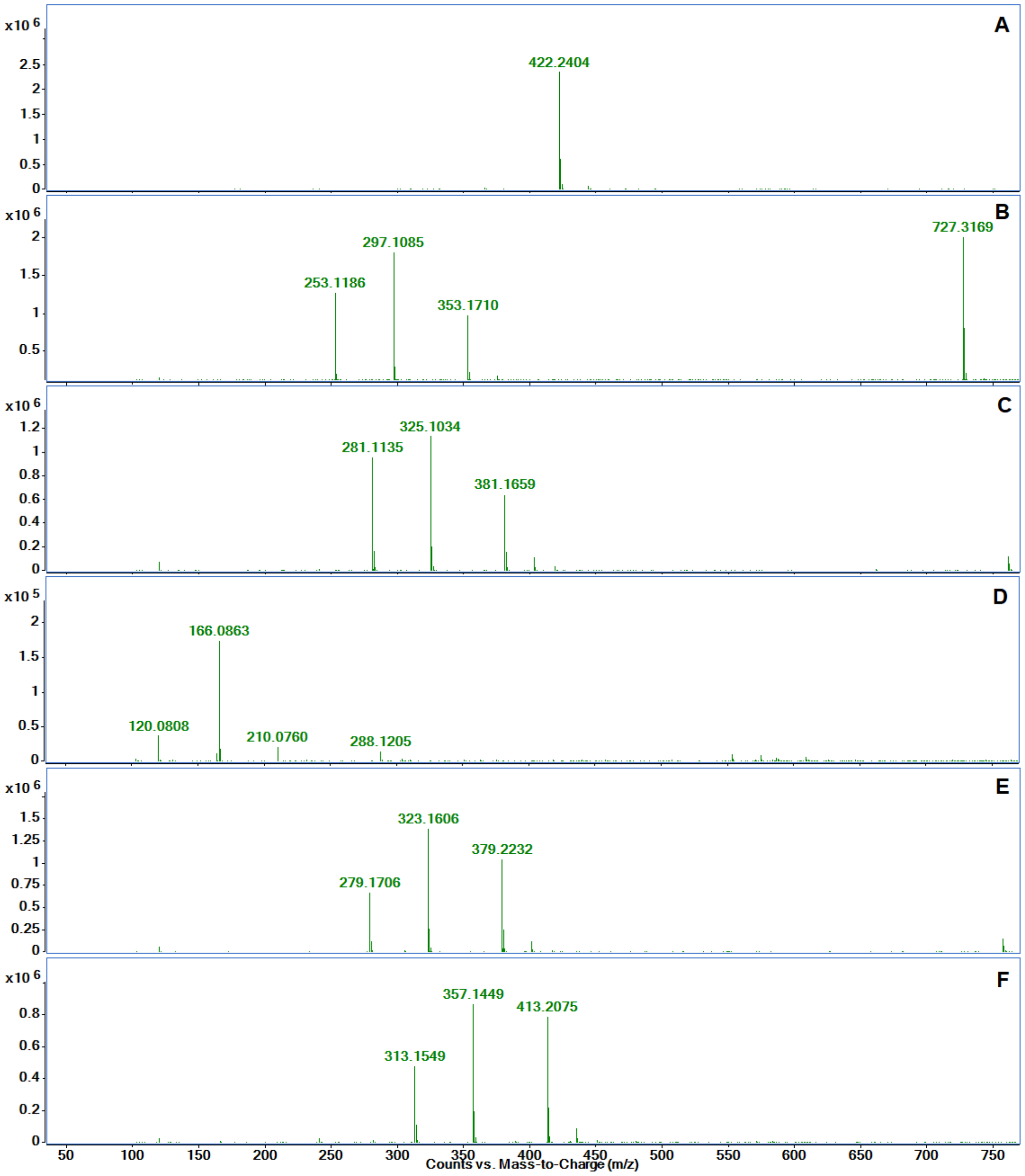

Supplementary Figure 4: Mass spectra of species formed between 10 mM **2a** and 50 mM amino acid mixture I (D, S, L, R, and F, each at 10 mM) shown for: A) **2a-R** (retention time 8.60 min), B) **2a-S** (retention time 9.94 min), C) **2a-D** (retention time 10.15 min), D) **2a-OH** (retention time 12.12 min), E) **2a-L** (retention time 13.59 min), F) **2a-F** (retention time13.88 min), in Supplementary Figure 3, obtained from the LC-MS analysis.

Calculated m/z $[M+H]^+$: **2a-R** 422.2398, **2a-S** 353.1707**, 2a-D** 381.1656**, 2a-OH** 266.1387, **2a-L** 379.2227, **2a-F** 413.2071.

Observed m/z $[M+H]^+$: **2a-R** 422.2404, **2a-S** 353.1710**, 2a-D** 381.1659**, 2a-L** 379.2232, **2a-F** 413.2075, $[M+Na]^+$: **2a-OH** 288.1205, $[M+H\text{-}Boc+H]^+$: **2a-OH** 166.0863. The mass for $[M+H\text{-}Boc+H]^+$ fragment has also been observed previously.[2]

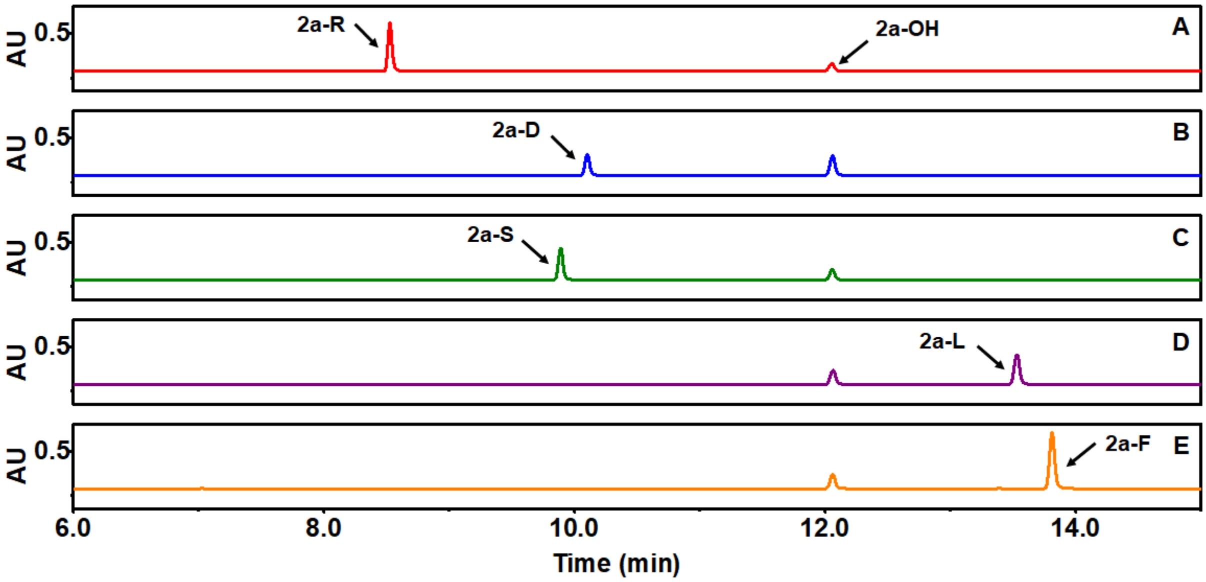

Supplementary Figure 5: UPLC chromatograms of reactions between 10 mM **2a** and A) 10 mM R, B) 10 mM D, C) 10 mM S, D) 10 mM L and E) 10 mM F in 0.6 M borate buffer, pH 9.1. We use the single letter code for amino acids. Measurements were taken after 60 minutes.

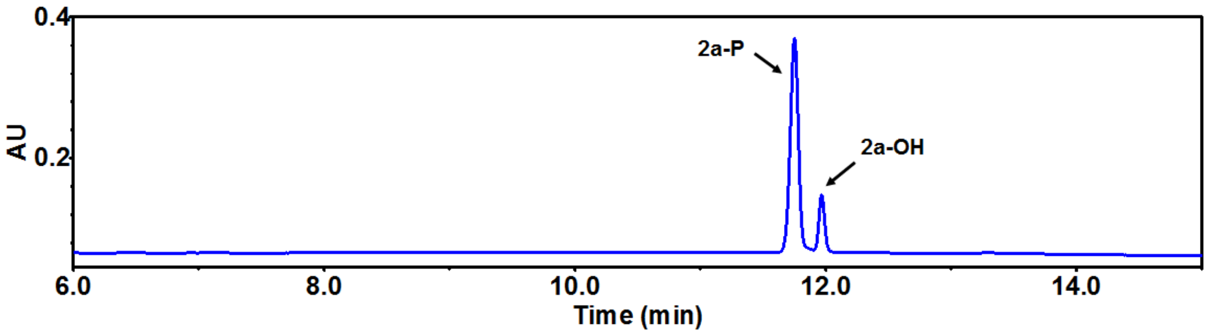

Supplementary Figure 6: UPLC chromatogram of reaction between 10 mM **2a** and 10 mM P in 0.6 M borate buffer, pH 9.1. Measurements were taken after 60 minutes.

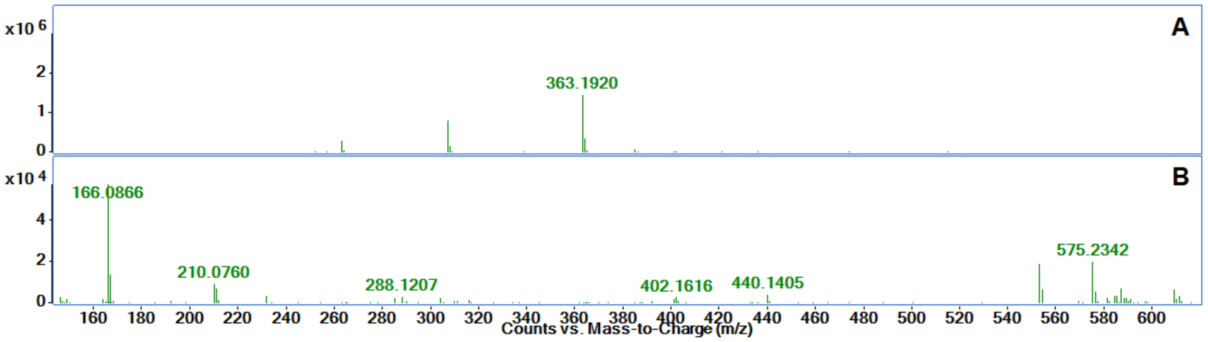

Supplementary Figure 7: Mass spectra of species formed between 10 mM **2a** and 10 mM P shown for: A) **2a-P** (retention time 11.75 min), B) **2a-OH** (retention time 11.96 min) in Supplementary Figure 6, obtained from the LC-MS analysis.

Calculated m/z $[M+H]^+$: **2a-P** 363.1914, **2a-OH** 266.1387.
Observed m/z $[M+H]^+$: **2a-P** 363.1920, $[M+Na]^+$: **2a-OH** 288.1207.

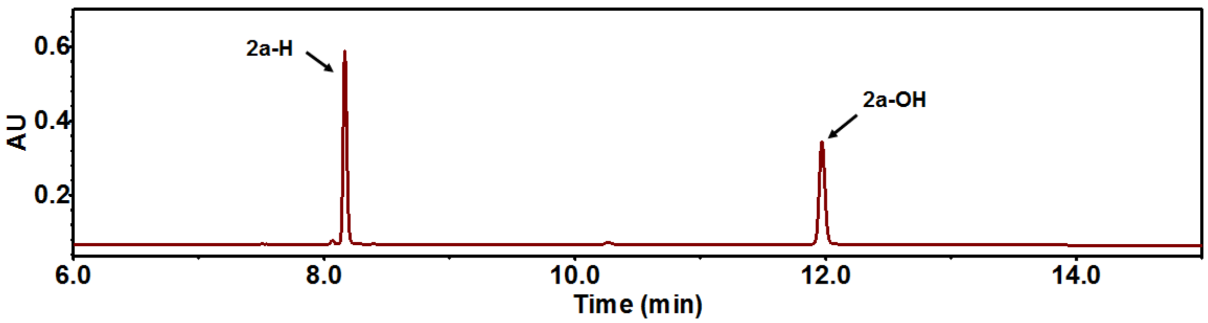

Supplementary Figure 8: UPLC chromatogram of reaction between 10 mM **2a** and 10 mM H in 0.6 M borate buffer, pH 9.1. Measurements were taken after 60 minutes.

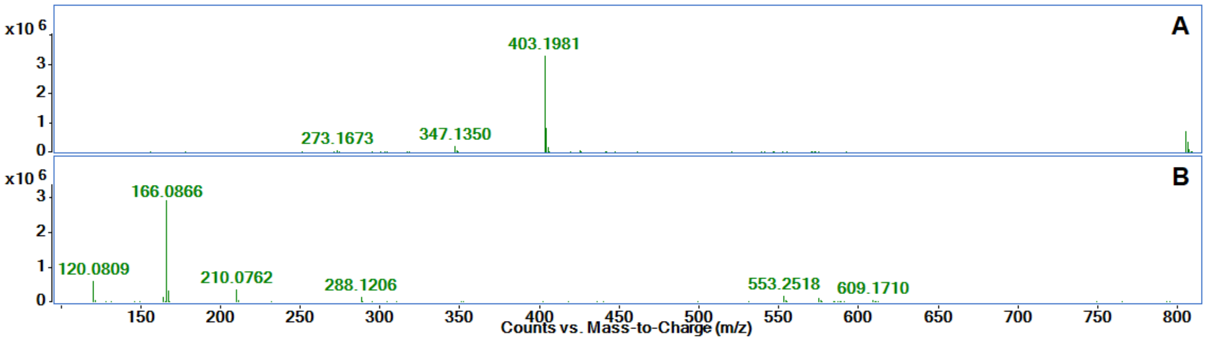

Supplementary Figure 9: Mass spectra of species formed between 10 mM **2a** and 10 mM H shown for: A) **2a-H** (retention time 8.16 min), B) **2a-OH** (retention time 11.96 min) in Supplementary Figure 8, obtained from the LC-MS analysis.

Calculated m/z $[M+H]^+$: **2a-H** 403.1976, **2a-OH** 266.1387.

Observed m/z $[M+H]^+$: **2a-H** 403.1981, $[M+Na]^+$: **2a-OH** 288.1206.

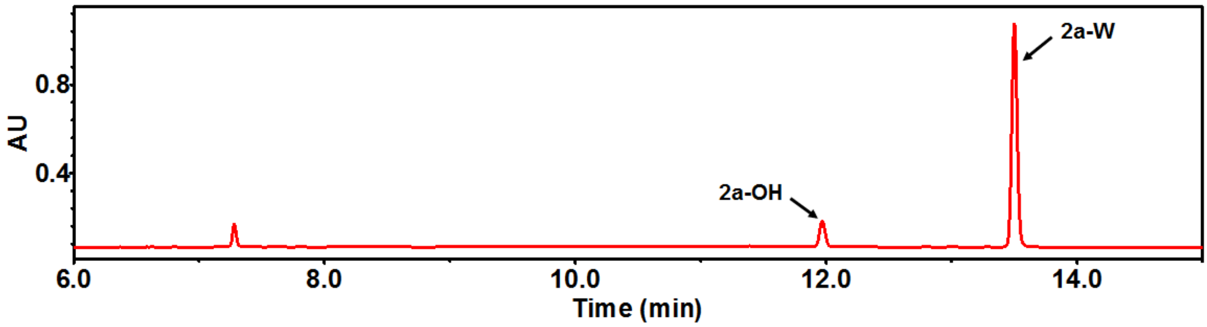

Supplementary Figure 10: UPLC chromatogram of reaction between 10 mM **2a** and 10 mM W in 0.6 M borate buffer, pH 9.1. Measurements were taken after 60 minutes.

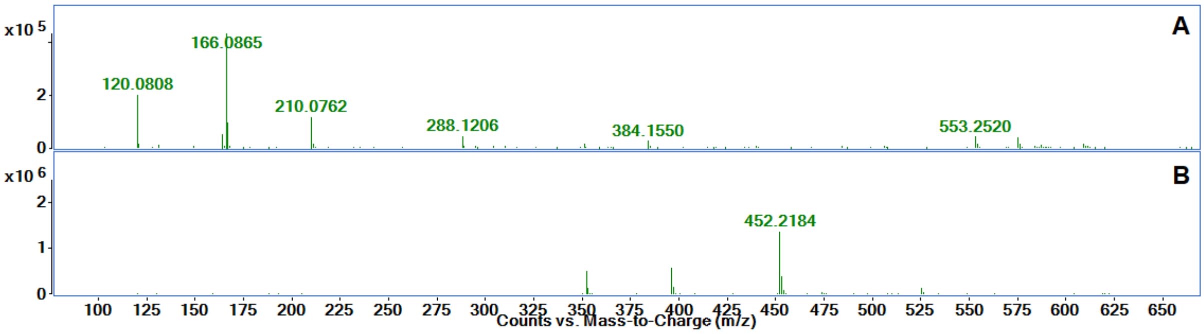

Supplementary Figure 11: Mass spectra of species formed between 10 mM **2a** and 10 mM W shown for: A) **2a-OH** (retention time 11.75 min), B) **2a-W** (retention time 13.50 min) in Supplementary Figure 10, obtained from the LC-MS analysis.

Calculated m/z $[M+H]^+$: **2a-OH** 266.1387, **2a-W** 452.2180.
Observed m/z $[M+H]^+$: **2a-W** 452.2184, $[M+Na]^+$: **2a-OH** 288.1206.

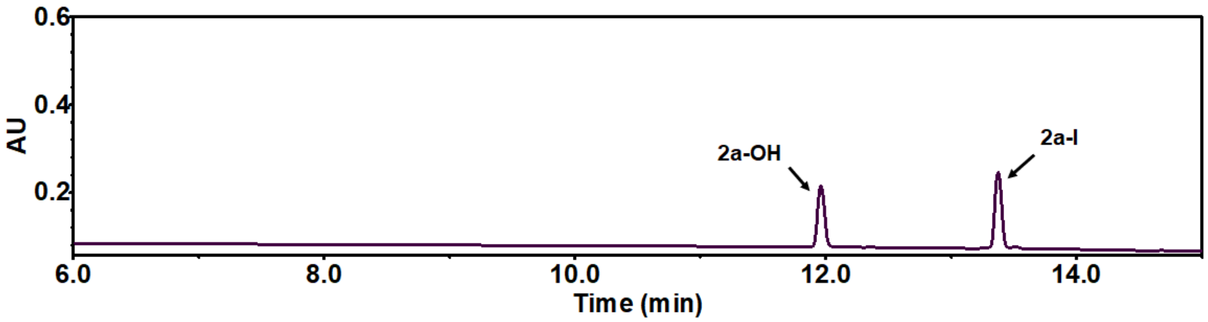

Supplementary Figure 12: UPLC chromatogram of reaction between 10 mM **2a** and 10 mM I in 0.6 M borate buffer, pH 9.1. Measurements were taken after 60 minutes.

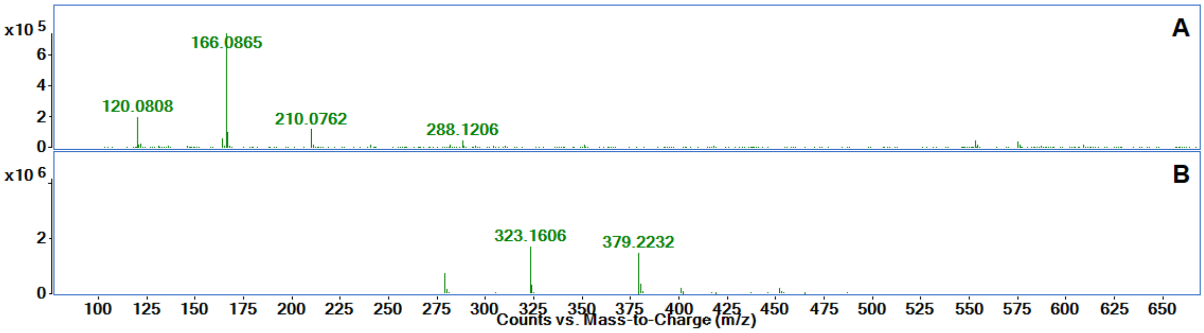

Supplementary Figure 13: Mass spectra of species formed between 10 mM **2a** and 10 mM I shown for: A) **2a-OH** (retention time 11.96 min), B) **2a-I** (retention time 13.37 min) in Supplementary Figure 12, obtained from the LC-MS analysis.

Calculated m/z $[M+H]^+$: **2a-OH** 266.1387, **2a-I** 379.2227.

Observed m/z $[M+H]^+$: **2a-I** 379.2232, $[M+Na]^+$: **2a-OH** 288.1206.

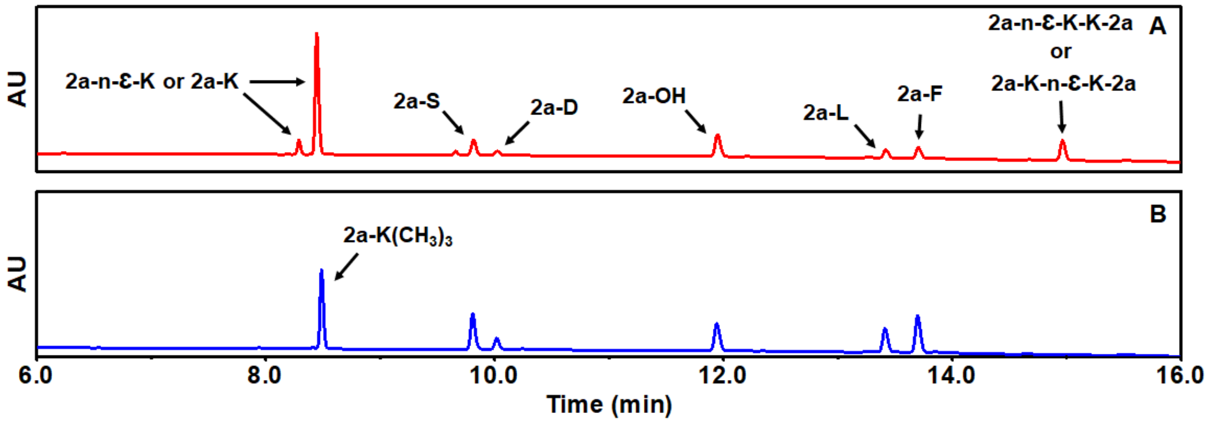

Supplementary Figure 14: UPLC chromatograms of reactions between 10 mM **2a** and A) 50 mM amino acid mixture (D+S+L+K+F**,** each amino acid at 10 mM), B) 50 mM amino acid mixture (D+S+L+K(CH_3)$_3$+F**,** each amino acid at 10 mM), in 0.6 M borate buffer, pH 9.1. Measurements were taken after 30 minutes.

Step 1:

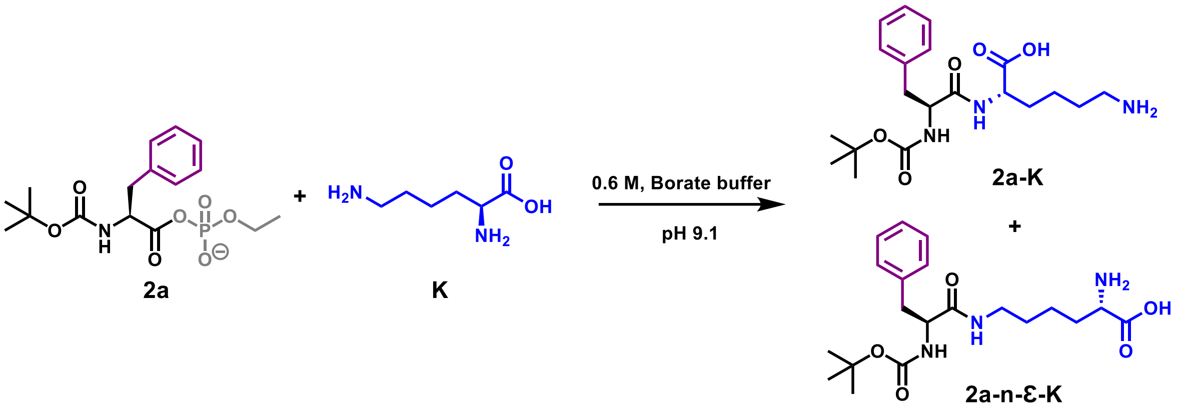

Step 2:

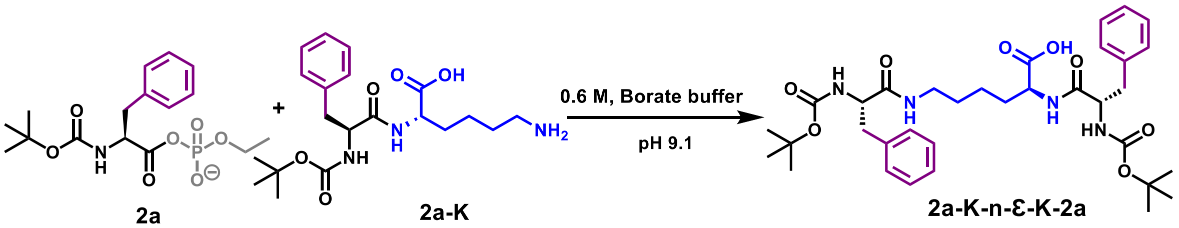

Or

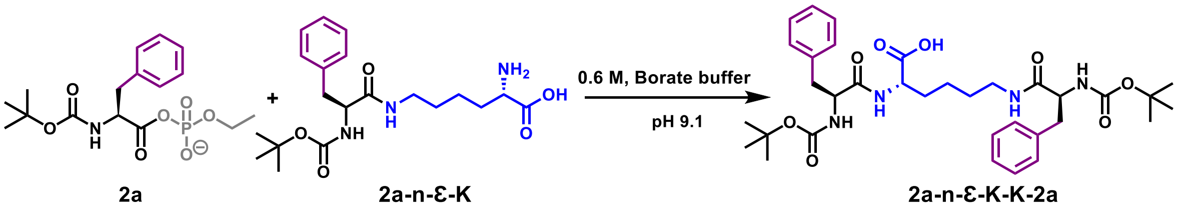

Supplementary Figure 15: Possible reaction scheme between **2a** and K. Firstly, primary amine of lysine (K) reacts with **2a**, forming the Ist amide **2a-K** and **2a-n-ε-K**, followed by the formation of the second amide **2a-K-n-ε-K-2a** or **2a-n-ε-K-K-2a** through the attack of the one of the free NH_2 group of the lysine on **2a** simultaneously.

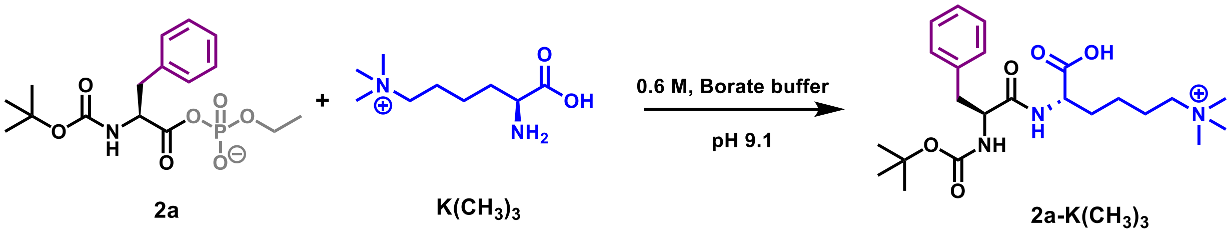

Supplementary Figure 16: Reaction scheme between **2a** and K$(CH_3)_3$.

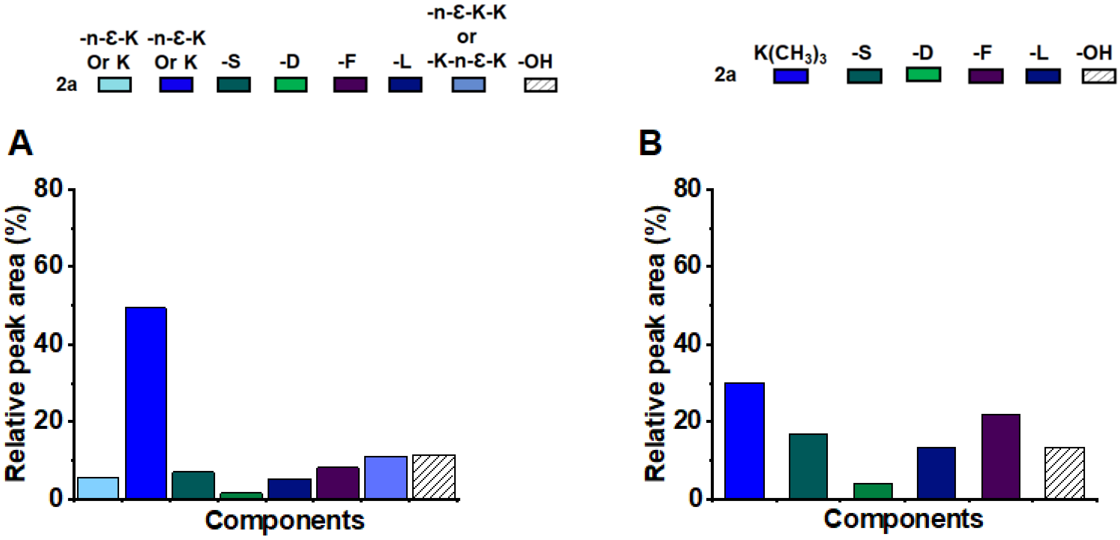

Supplementary Figure 17: Bar graphs showing peptide conversion between 10 mM **2a** and A) 50 mM amino acid mixture (D+S+L+K+F, each amino acid at 10 mM) and B) 50 mM amino acid mixture (D+S+L+K(CH_3)$_3$+F**,** each amino acid at 10 mM), in 0.6 M borate buffer, pH 9.1. In each bar graph, striped bars represent the hydrolysis product **2a-OH.** Peptide coupling yields are measured after 30 minutes of reaction.

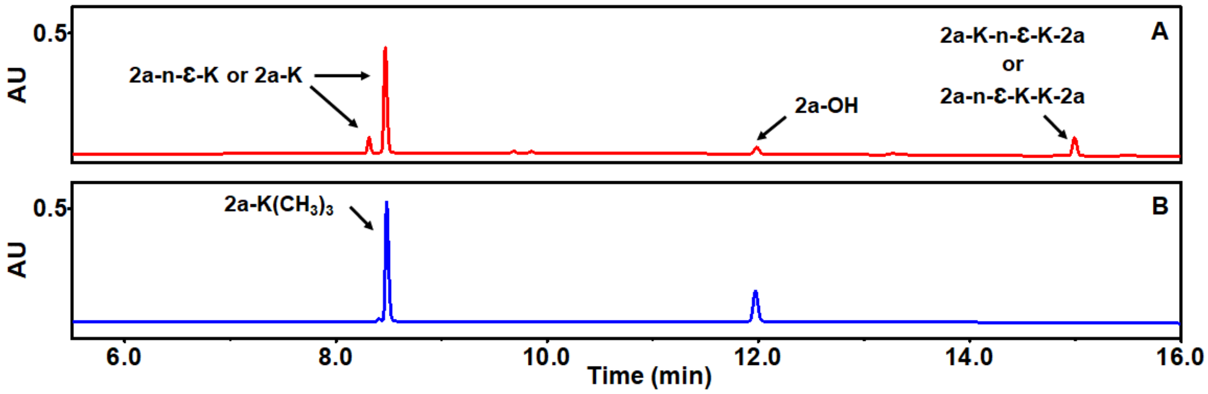

Supplementary Figure 18: UPLC chromatograms of reactions between 10 mM **2a** and A) 10 mM K, B) 10 mM $K(CH_3)_3$, in 0.6 M borate buffer, pH 9.1. Measurements were taken after 60 minutes.

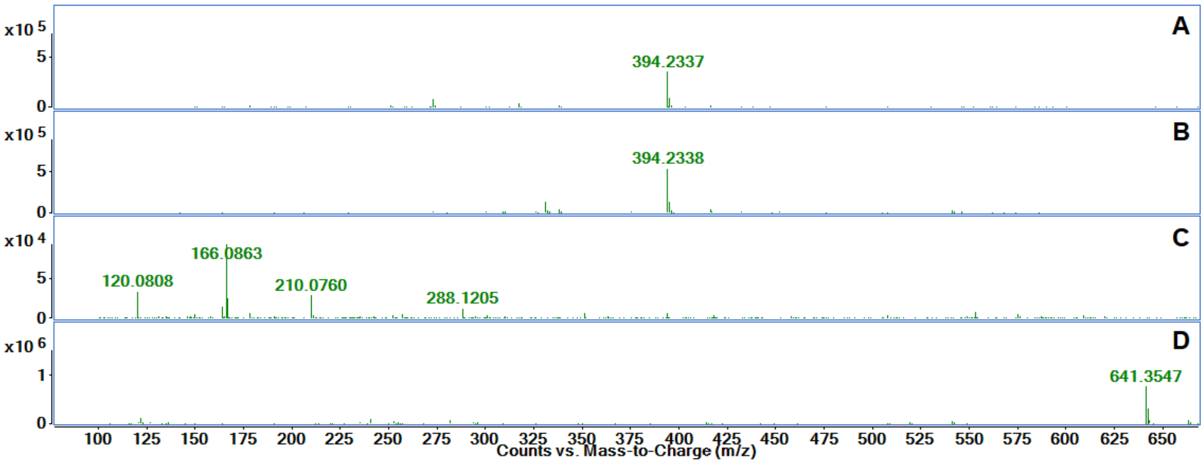

Supplementary Figure 19: Mass spectra of species formed between 10 mM **2a** and 10 mM K shown for**:** A) **2a-n-ε-K** or **2a-K** (retention time 8.28 min), B) **2a-K** or **2a-n-ε-K** (retention time 8.44min), C) **2a-OH** (retention time 11.94 min), D) **2a-K-n-ε-K-2a** or **2a- n-ε-K-K-2a** (retention time 14.99 min), in Supplementary Figure 18A, obtained from the LC-MS analysis.

Calculated m/z $[M+H]^+$: **2a-n-ε-K** 394.2336**, 2a-K** 394.2336, **2a-OH** 266.1387, **2a-K-n-ε-K-2a** 641.3545.

Observed m/z $[M+H]^+$: **2a-n-ε-K** 394.2337**, 2a-K** 394.2338, **2a-K-n-ε-K-2a** or **2a- n-ε-K-K-2a** 641.3547, $[M+Na]^+$: **2a-OH** 288.1205.

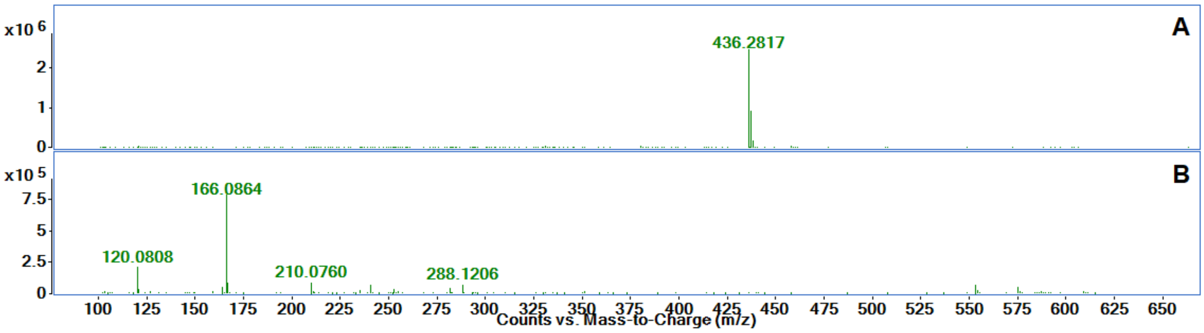

Supplementary Figure 20: Mass spectra of species formed between10 mM **2a** and 10 mM K(CH_3)$_3$ shown for: A) **2a-K(CH_3)$_3$** (retention time 8.49 min), B) **2a-OH** (retention time 11.94 min) in Supplementary Figure 18B, obtained from the LC-MS analysis.

Calculated m/z $[M]^+$: **2a- K(CH_3)$_3$** 436.2806, $[M+H]^+$: **2a-OH** 266.1387

Observed m/z $[M]^+$: **2a- K(CH_3)$_3$** 436.2817, $[M+Na]^+$: **2a-OH** 288.1206.

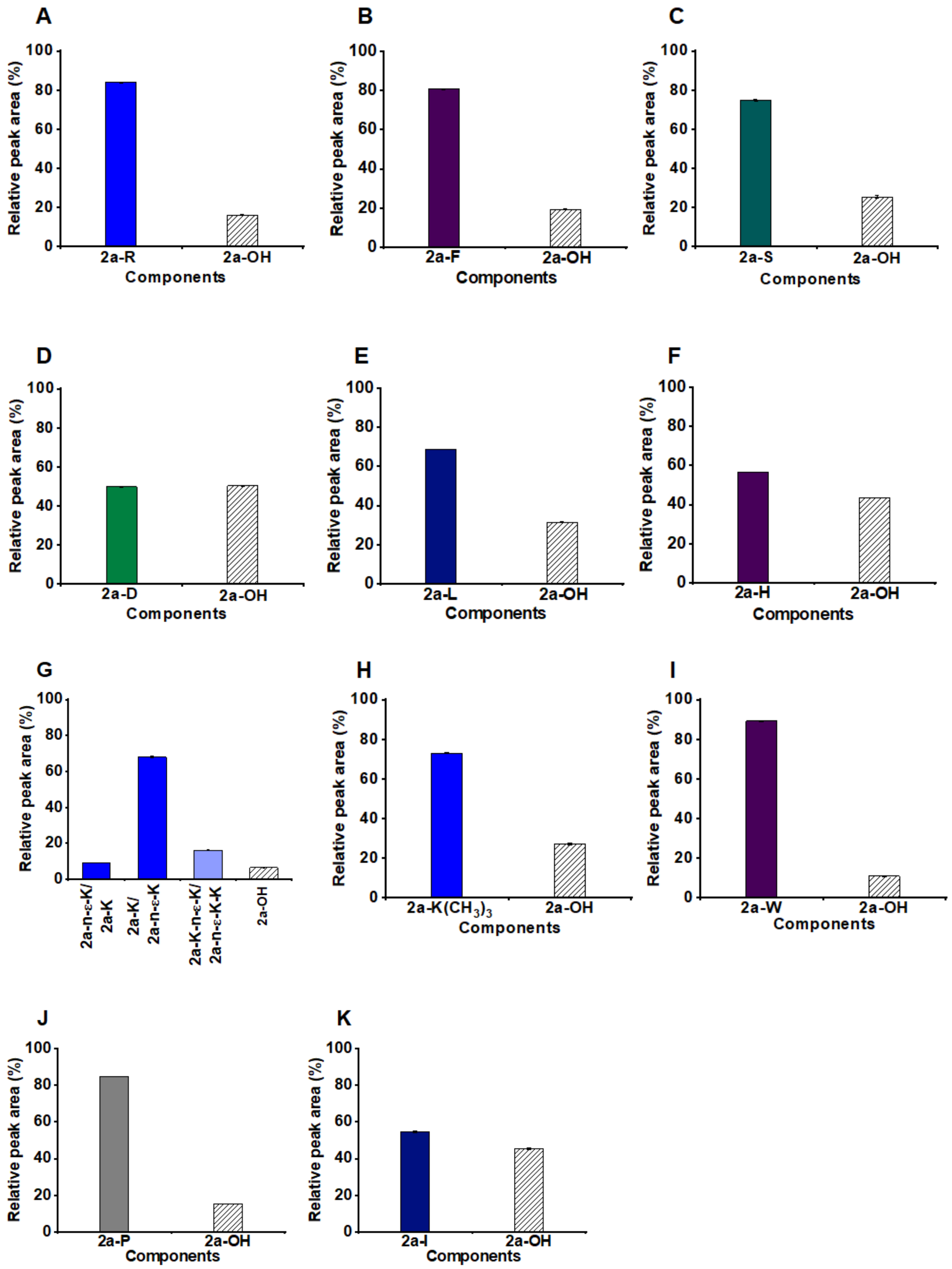

Supplementary Figure 21: Bar graphs showing peptide conversion between 10 mM **2a** and A) 10 mM R, B) 10 mM F, C) 10 mM S, D) 10 mM D, E) 10 mM L, F) 10 mM H, G) 10 mM K, H) 10 mM K(CH_3)$_3$, I) 10 mM W, J) 10 mM P, K), 10 mM I in 0.6 M borate buffer, pH 9.1. In each bar graph, striped bars represent the hydrolysis product **2a-OH**. Peptide coupling yields were measured after 60 minutes. Error bars represent standard deviation from three independent experiments.

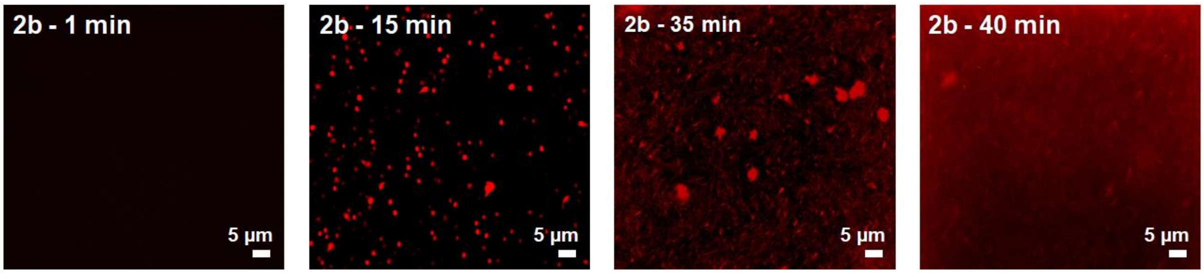

Supplementary Figure 22: Time-dependent confocal microscopy images of 10 mM **2b**, in 0.6 M borate buffer, pH 9.1.

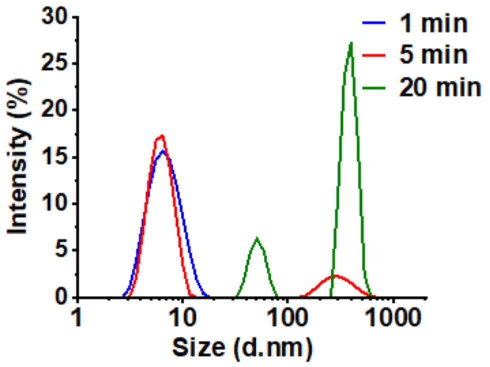

Supplementary Figure 23: Time-dependent size distribution measured by DLS of 10 mM **2b** in 0.6 M borate buffer, pH 9.1.

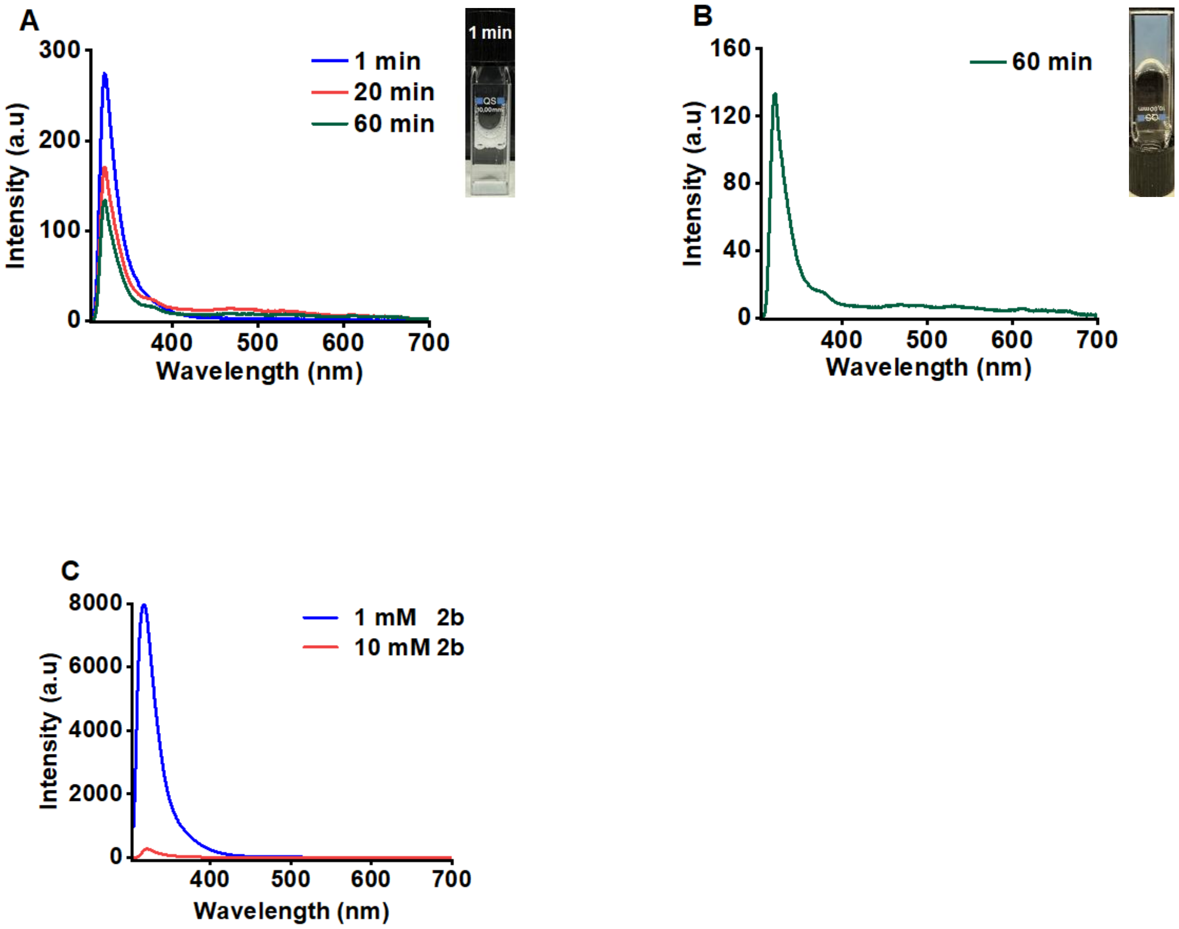

Supplementary Figure 24: Fluorescence emission spectra of A) 10 mM **2b** at different time points. B) Zoomed in spectra of 10 mM **2b** at 60 min. C) Concentration-dependent fluorescence emission spectra of **2b** in 0.6 M borate buffer, pH 9.1.Digital photos represent the macroscopic behavior of samples.

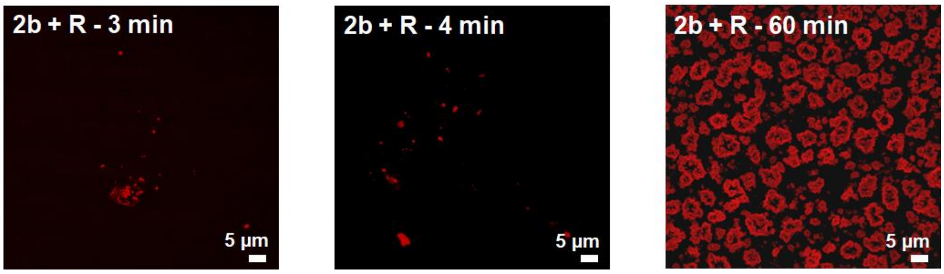

Supplementary Figure 25: Time-dependent confocal microscopy images of reaction between 10 mM **2b** and 10 mM R, in 0.6 M borate buffer, pH 9.1.

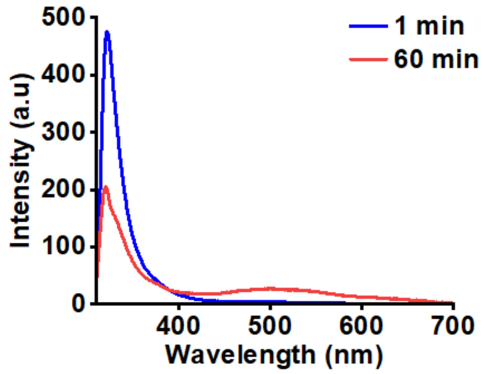

Supplementary Figure 26: Time-dependent Fluorescence emission spectra of 10 mM **2b** and 10 mM R, in 0.6 M borate buffer, pH 9.1.

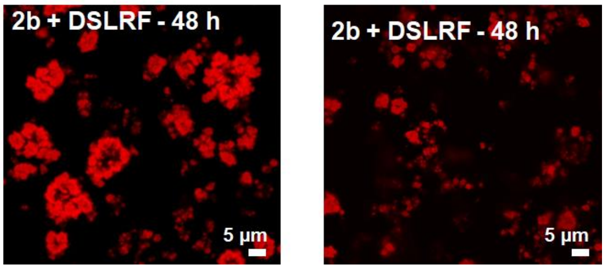

Supplementary Figure 27: Confocal microscopy images of reaction between 10 mM **2b** and 50 mM amino acid mixture 1 (D, S, L, R, F, each amino acid is at 10 mM concentration) measured after 48 hours, in 0.6 M borate buffer, pH 9.1.

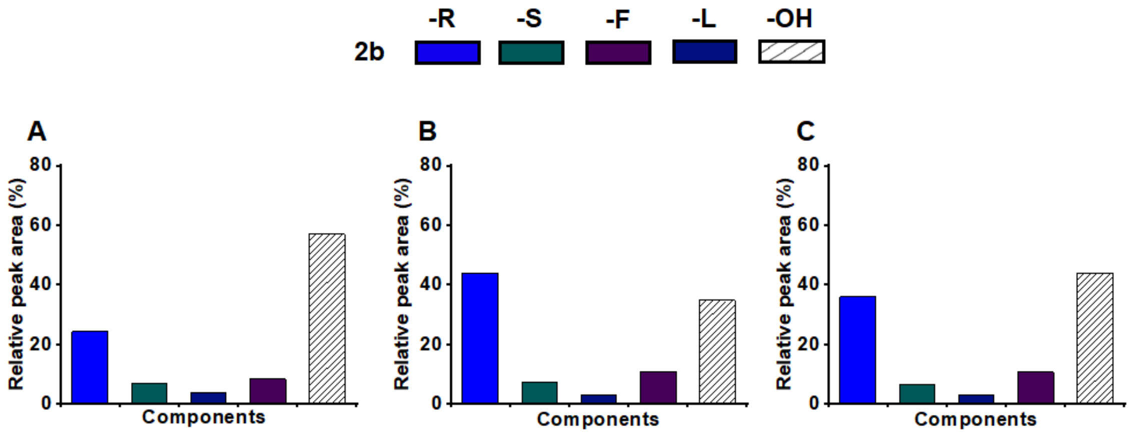

Supplementary Figure 28: Bar graphs showing peptide conversion between 10 mM **2b** and A) amino acid mixture I (10 mM D+S+L+R+F) at a 1:1 ratio in borate buffer (pH 9.1, 0.6 M), B) Amino acid mixture I (50 mM D+S+L+R+F) at a 1:5 ratio in PBS buffer (pH 8.1, 0.1 M) and C) Amino acid mixture I (50 mM D+S+L+R+F) at a 1:5 ratio in PBS buffer (pH 7.5, 0.1 M). In each bar graph, striped bars represent the hydrolysis product **2b-OH**. Peptide coupling yields were measured after 48 hours.

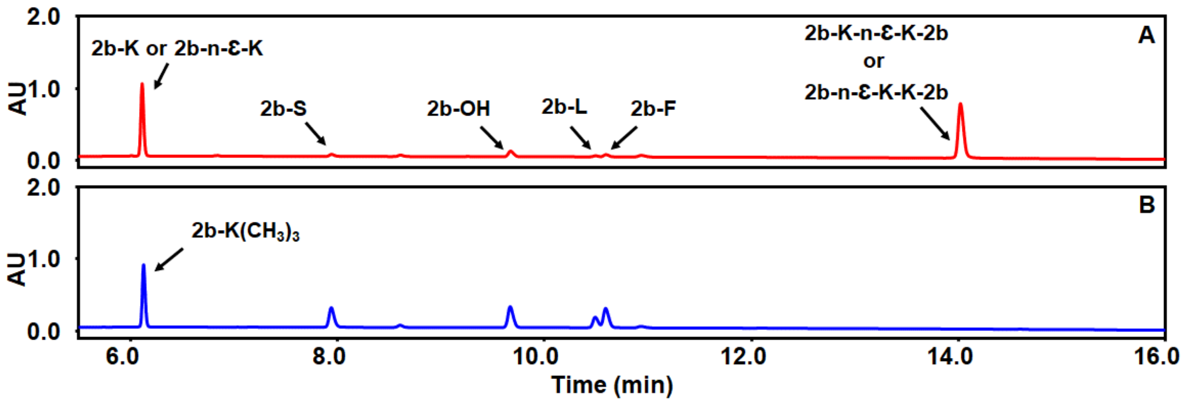

Supplementary Figure 29: UPLC chromatograms of reactions between 10 mM **2b** and A) 50 mM amino acid mixture (D+S+L+K+F**,** each amino acid at 10 mM) and B) 50 mM amino acid mixture (D+S+L+K$(CH_3)_3$+F**,** each amino acid at 10 mM), in 0.6 M borate buffer, pH 9.1. Measurements were taken after 48 hours.

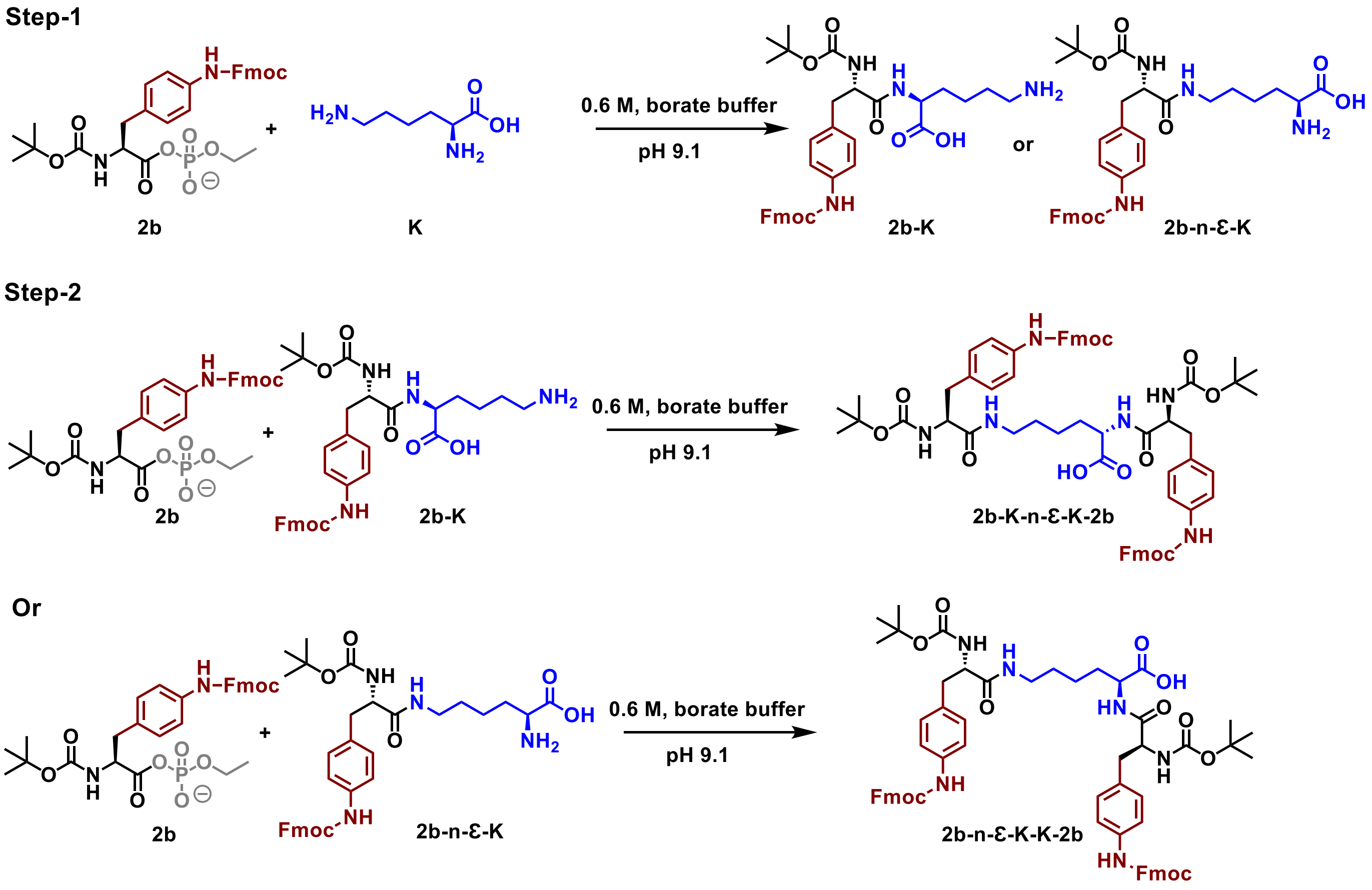

Supplementary Figure 30: Possible reaction scheme of **2b** and K. Firstly, primary amine of lysine (K) reacts with **2b**, forming the 1st amide **2b-K** or **2b-n-ε-K**, followed by the formation of the second amide **2b-K-n-ε-K-2b** or **2b-n-ε-K-K-2b** through the attack of the free NH_2 group on **2b** simultaneously. Similar abbreviations have also been used in case of Ornithine (O) and 2,3-Diaminopropionic acid (Dpr) to denote one-step and two-step couplings, respectively.

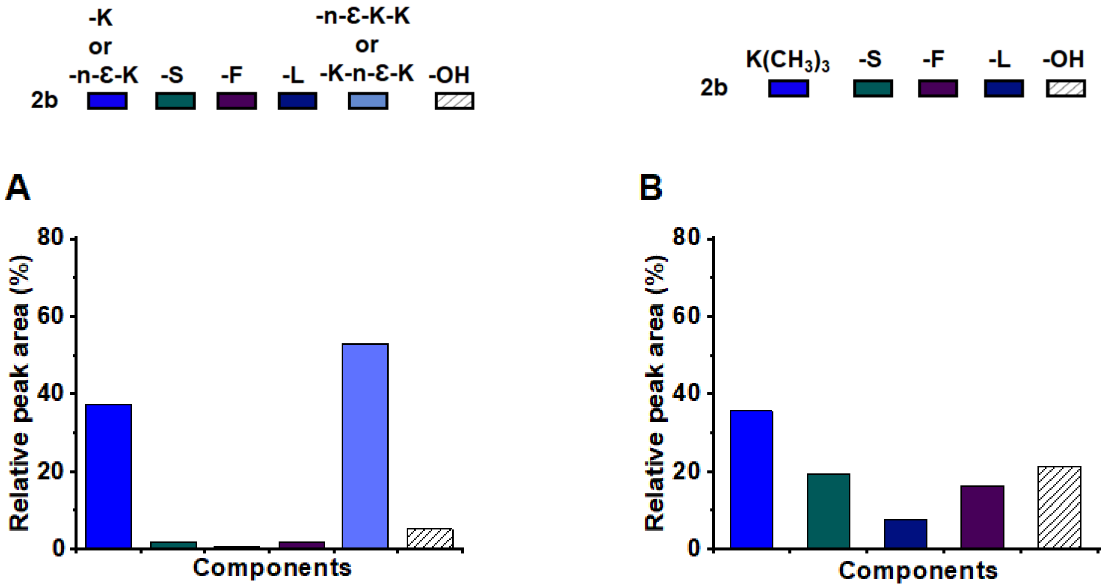

Supplementary Figure 31: Bar graphs showing peptide conversion between 10 mM **2b** and **A)** 50 mM amino acid mixture (D+S+L+K+F**,** each amino acid at 10 mM), B) 50 mM amino acid mixture (D+S+L+K(CH_3)$_3$+F**,** each amino acid at 10 mM), in 0.6 M borate buffer, pH 9.1. In each bar graph striped bar represent the hydrolysis product **2b-OH**. Peptide coupling yields were measured after 48 hours.

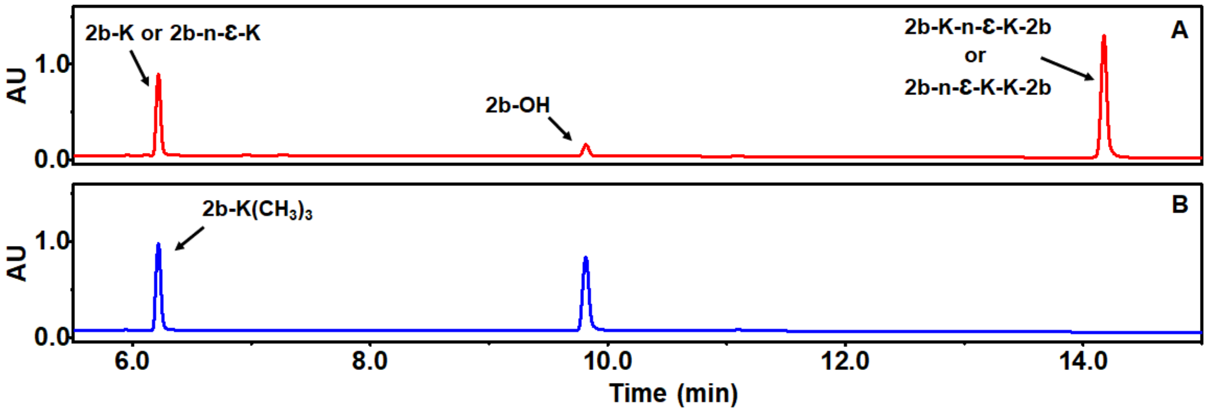

Supplementary Figure 32: UPLC chromatograms of reactions between 10 mM **2b** and A) 10 mM K, B) 10 mM $K(CH_3)_3$, in 0.6 M borate buffer, pH 9.1. Measurements were taken after 48 hours.

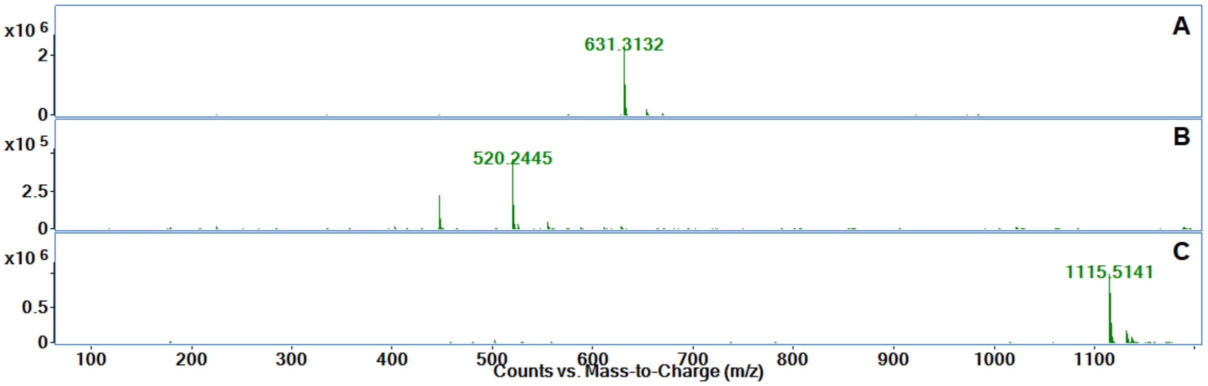

Supplementary Figure 33: Mass spectra of species formed between 10 mM **2b** and 10 mM K, shown for: A) **2b-K** or **2b-n-ε-K** (retention time 6.19 min), B) **2b-OH** (retention time 9.70 min), C) **2b-K-n-ε-K-2b** or **2b-n-ε-K-K-2b** (retention time 14.17 min), in supplementary Figure 32 A, obtained from the LC-MS analysis.

Calculated m/z $[M+H]^+$: **2b-K** or **2b-n-ε-K** 631.3126**, 2b-K-n-ε-K-2b** or **2b-n-ε-K-K-2b** 1115.5124 **2b-OH** 503.2177

Observed m/z $[M+H]^+$: **2b-K** or **2b-n-ε-K** 631.3132**, 2b-K-n-ε-K-2b** or **2b-n-ε-K-K-2b** 1115.5141, $[M+H_2O]$: **2b-OH** 520.2445.

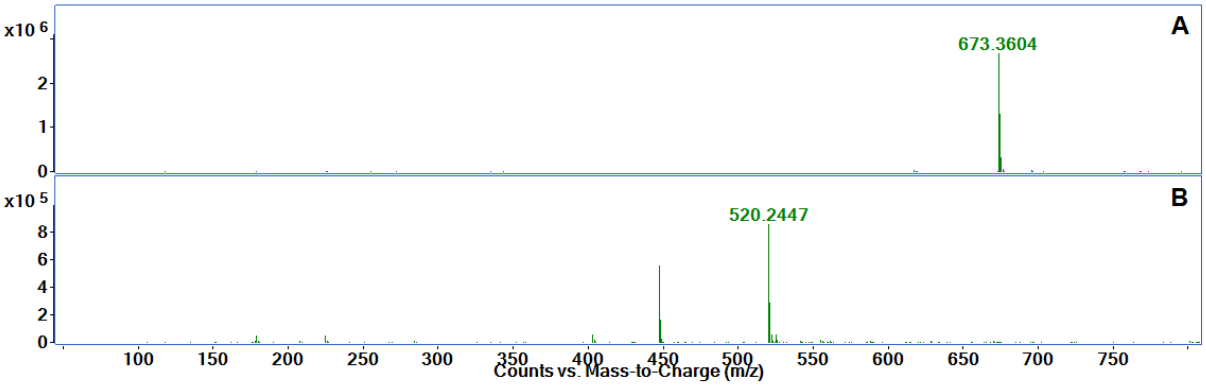

Supplementary Figure 34: Mass spectra of species formed between 10 mM **2b** and 10 mM $K(CH_3)_3$, shown for: A) **2b-K(CH$_3$)$_3$** (retention time 6.21 min), B) **2b-OH** (retention time 9.70 min) in supplementary Figure 32 B, obtained from the LC-MS analysis.

Calculated m/z $[M]^+$: **2b-K(CH$_3$)$_3$** 673.3596, $[M+H]^+$: **2b-OH** 503.2177.

Observed m/z $[M]^+$: **2b-K(CH$_3$)$_3$** 673.3604, $[M+H_2O]$: **2b-OH** 520.2447.

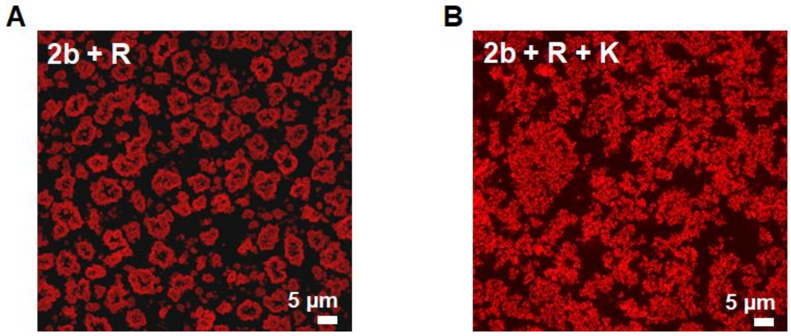

Supplementary Figure 35: Confocal microscopy images of reaction between A) 10 mM **2b** and 10 mM R, B) 10 mM **2b** and 20 mM amino acid mixture of R and K (10 mM each) in 0.6 M borate buffer, pH 9.1.

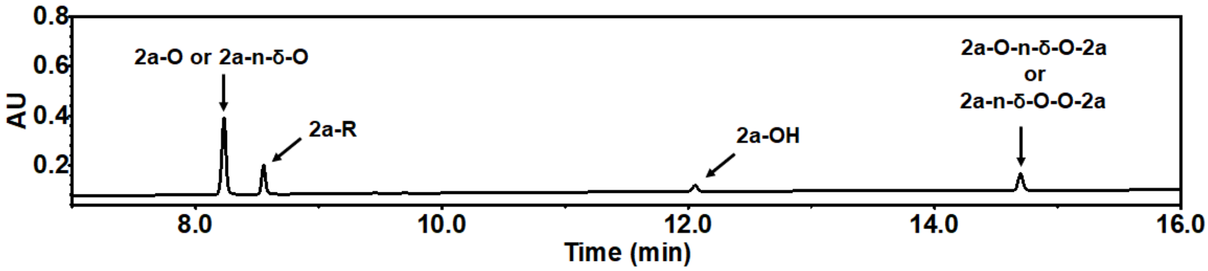

Supplementary Figure 36: UPLC chromatogram of reaction between 10 mM **2a** and 20 mM amino acid mixture containing R and O (each at 10 mM) in 0.6 M borate buffer, pH 9.1. Measurements were taken after 30 minutes.

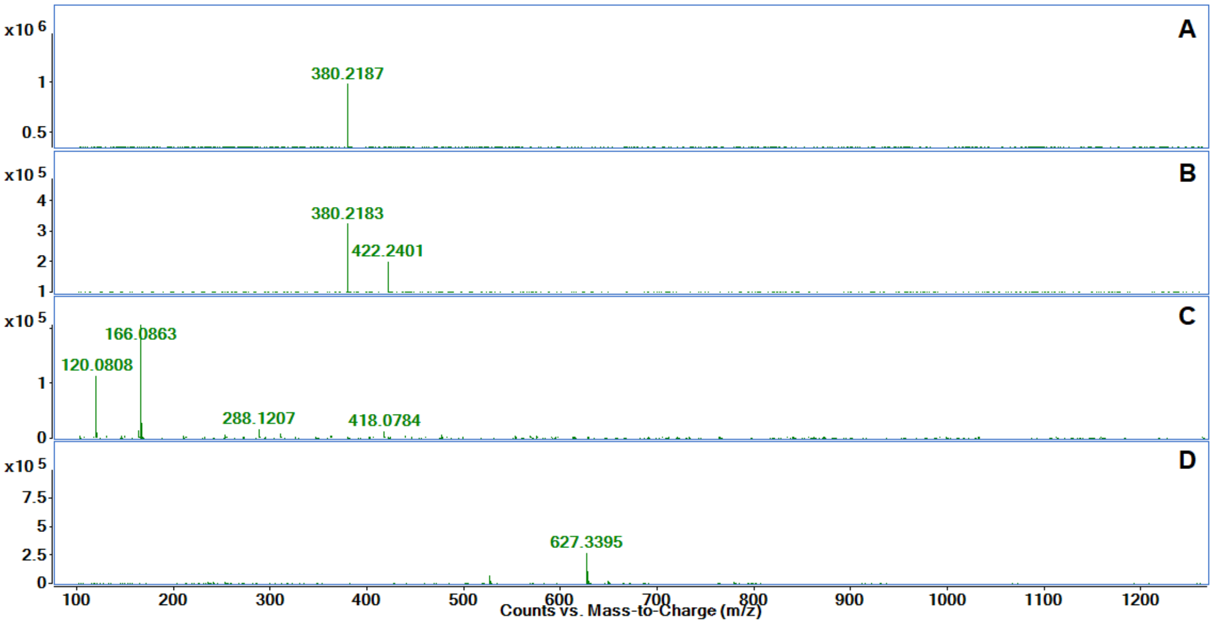

Supplementary Figure 37: Mass spectra of species formed between10 mM **2a** and 20 mM amino acid mixture containing R and O (each at 10 mM) shown for: A) **2a-O** or **2a-n-δ-O** (retention time 8.23 min), B) **2a-R** (retention time 8.55 min), C) **2a-OH** (retention time 12.05 min), D) **2a-O-n-δ-O-2a** or **2a-n-δ-O-O-2a** (retention time 14.69 min) in Supplementary Figure 36, obtained from the LC-MS analysis.

Calculated m/z $[M+H]^+$: **2a-O** or **2a-n-δ-O** 380.2120**, 2a-R** 422.2398**, 2a-OH** 266.1387, **2a-O-n-δ-O-2a** or **2a-n-δ-O-O-2a** 627.3388

Observed m/z $[M+H]^+$: **2a-O** or **2a-n-δ-O** 380.2187**, 2a-R** 422.2401**, 2a-O-n-δ-O** or **2a-n-δ-O-O-2a** 627.3395

$[M+Na]^+$: **2a-OH** 288.1207.

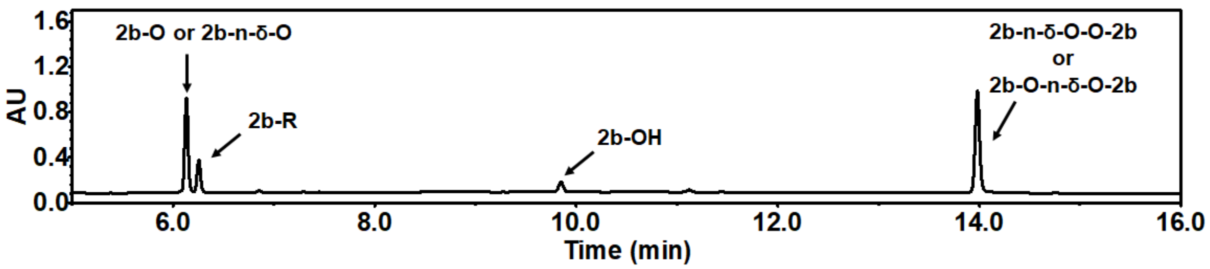

Supplementary Figure 38: UPLC chromatogram of reaction between 10 mM **2b** and 20 mM amino acid mixture containing R and O (each at 10 mM) in 0.6 M borate buffer, pH 9.1. Measurements were taken after 48 hours.

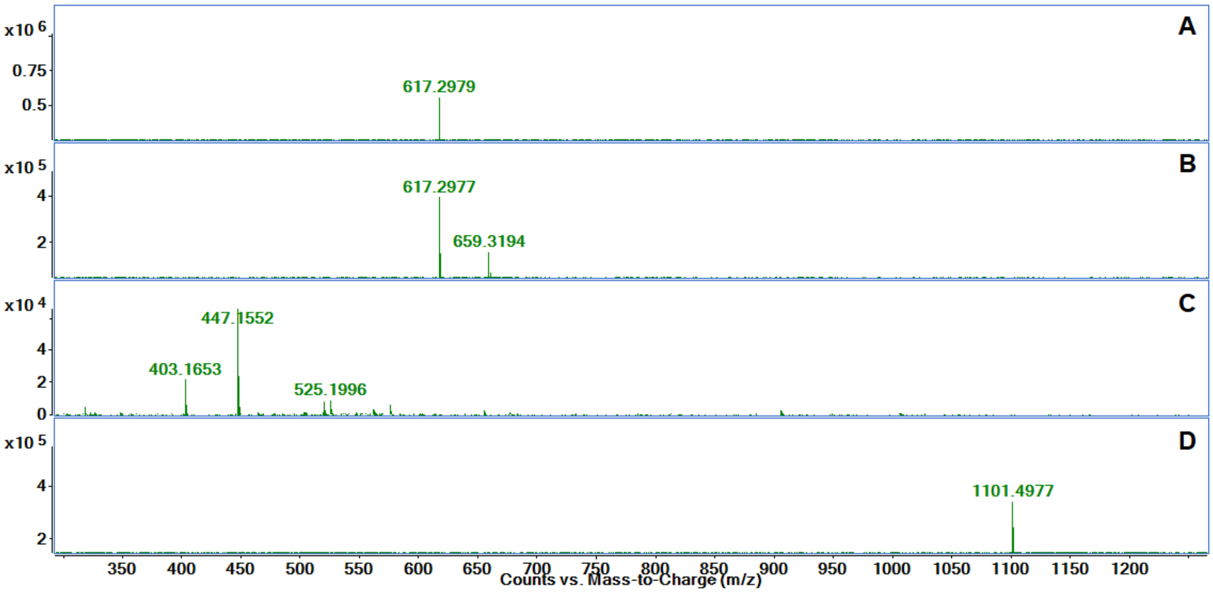

Supplementary Figure 39: Mass spectra of species formed between10 mM **2b** and 20 mM amino acid mixture containing R and O (each at 10 mM) shown for: A) **2b-O** or **2b-n-δ-O** (retention time 6.13 min), B) **2b-R** (retention time 6.25 min), C) **2b-OH** (retention time 9.85 min), D) **2b-n-δ-O-O-2b** or **2b-O-n-δ-O-2b** (retention time 13.98 min) in Supplementary Figure 38, obtained from the LC-MS analysis.

Calculated m/z $[M+H]^+$: **2b-O** or **2b-n-δ-O** 617.2970**, 2b-R** 659.3188**, 2b-OH** 503.2177, **2b-n-δ-O-O-2b** or **2b-O-n-δ-O-2b** 1101.4968

Observed m/z $[M+H]^+$: **2b-O** or **2b-n-δ-O** 617.2979**, 2b-R** 659.3194**, 2b-n-δ-O-O-2b** or **2b-O-n-δ-O-2b** 1101.4977

$[M+Na]^+$: **2b-OH** 525.1996.

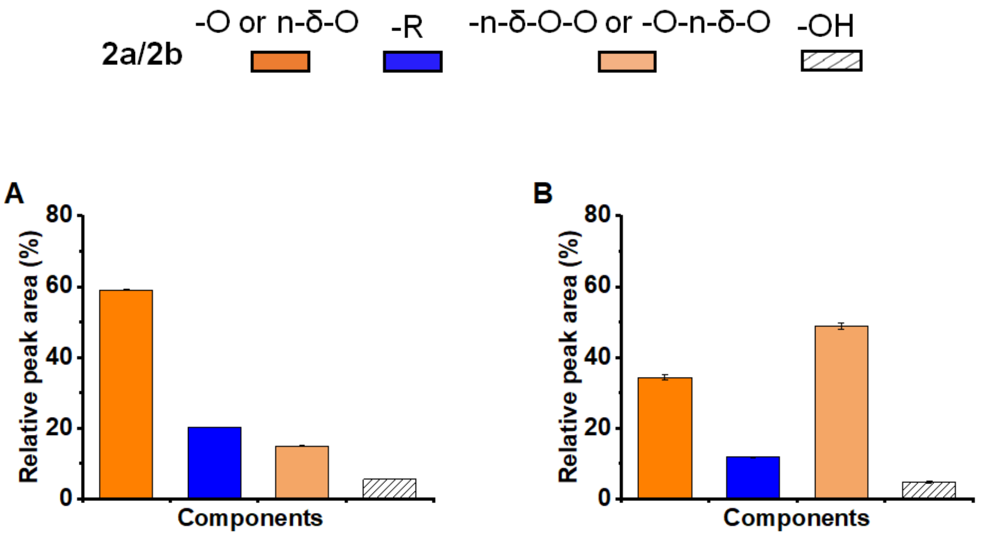

Supplementary Figure 40: Bar graphs showing peptide coupling between A) **2a**, B) **2b** with 20 mM amino acid mixture of R and O (10 mM each) in 0.6 M borate buffer, pH 9.1. In each bar graph striped bar represent the hydrolysis product **2a-OH**/**2b-OH**. Error bars represent standard deviation from three independent experiments. Peptide coupling yields were measured for **2a**, after 30 minutes, while for **2b**, after 48 hours.

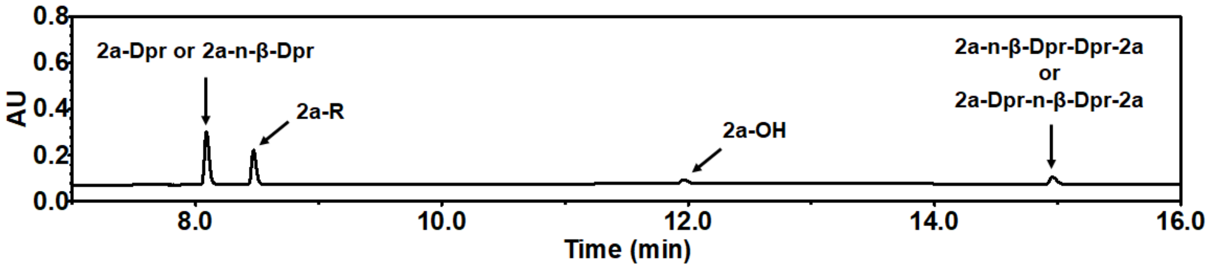

Supplementary Figure 41: UPLC chromatogram of competition reaction between 10 mM **2a** and 20 mM amino acid mixture of R and Dpr (each at 10 mM) in 0.6 M borate buffer, pH 9.1. Measurements were taken after 30 minutes.

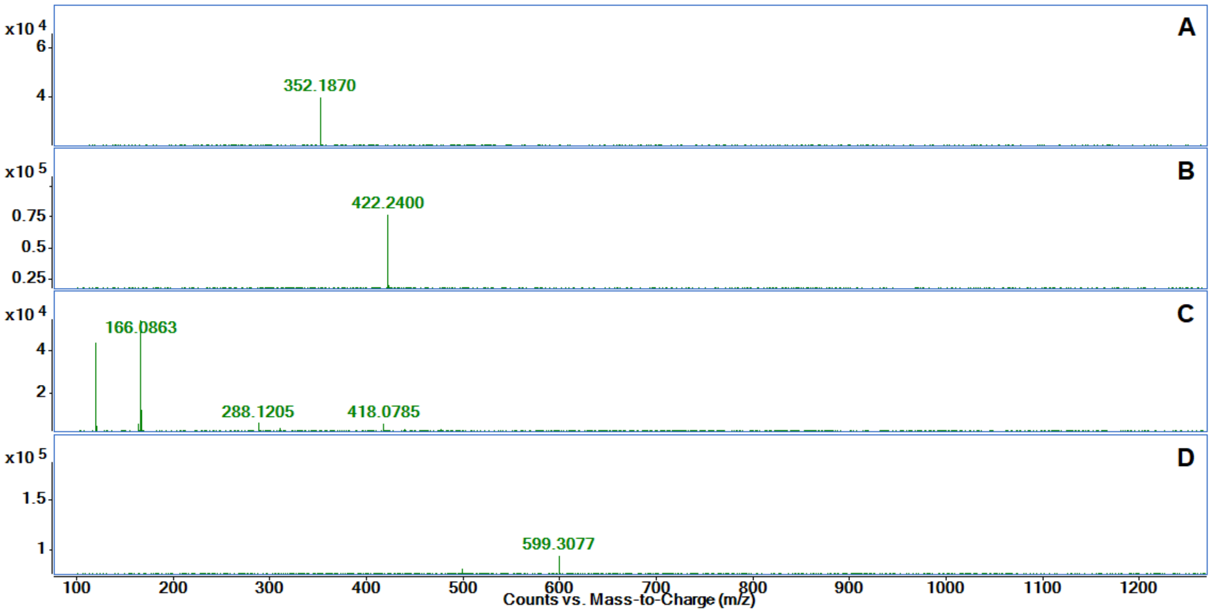

Supplementary Figure 42: Mass spectra of species formed between10 mM **2a** and 20 mM amino acid mixture of R and Dpr (each at 10 mM) shown for: A) **2a-Dpr** or **2a-n-β-Dpr** (retention time 8.11 min), B) **2a-R** (retention time 8.55 min), C) **2a-OH** (retention time 12.05 min), D) **2a-n-β-Dpr-Dpr-2a** or **2a-Dpr-n-β-Dpr-2a** (retention time 14.98 min) in Supplementary Figure 41, obtained from the LC-MS analysis.

Calculated m/z $[M+H]^+$: **2a-Dpr** or **2a-n-β-Dpr** 352.1867**, 2a-R** 422.2398**, 2a-OH** 266.1387, **2a-n-β-Dpr-Dpr-2a** or **2a-Dpr-n-β-Dpr-2a** 599.3075

Observed m/z $[M+H]^+$: **2a-Dpr** or **2a-n-β-Dpr** 352.1870**, 2a-R** 422.2400**, 2a-n-β-Dpr-Dpr-2a** or **2a-Dpr-n-β-Dpr-2a** 599.3077

$[M+Na]^+$: **2a-OH** 288.1205.

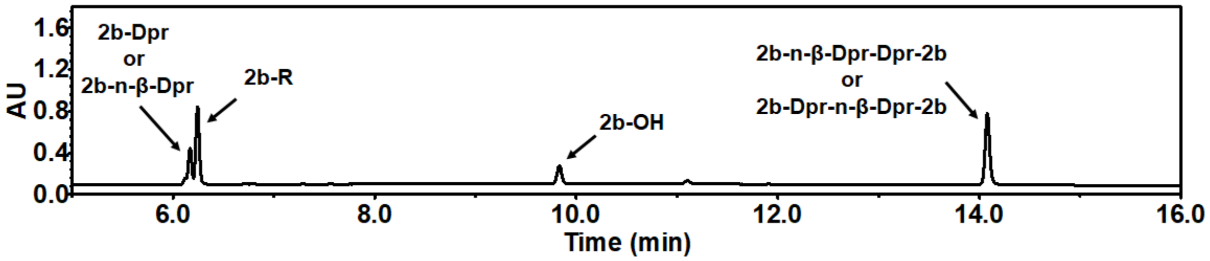

Supplementary Figure 43: UPLC chromatogram of reaction between 10 mM **2b** and 20 mM amino acid mixture containing R and Dpr (each at 10 mM) in 0.6 M borate buffer, pH 9.1. Measurements were taken after 48 hours.

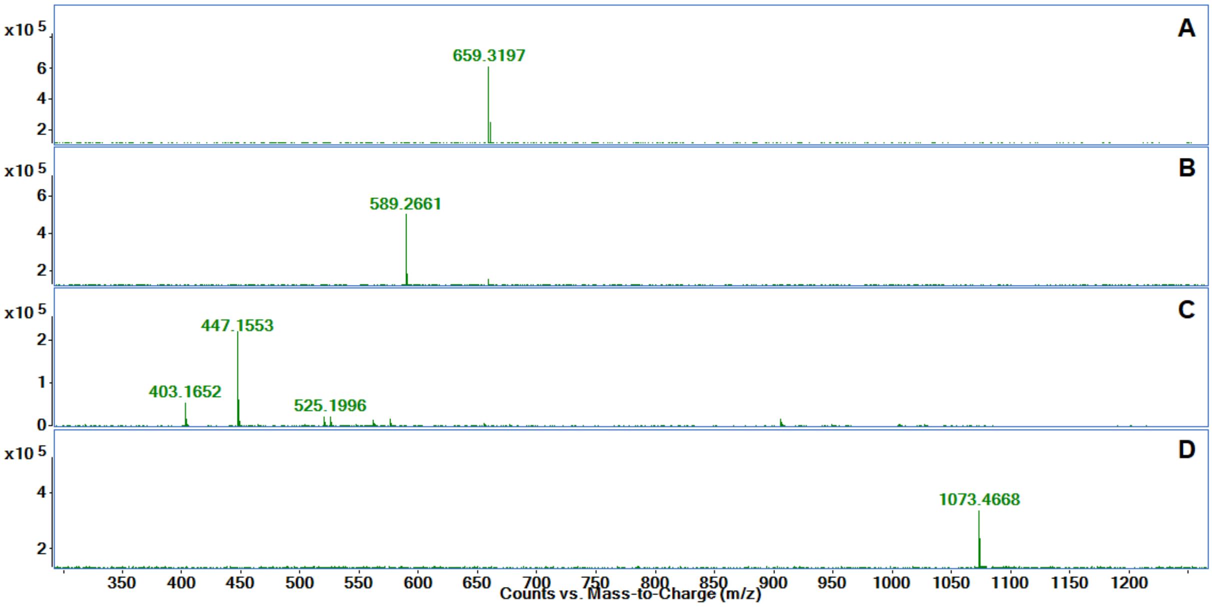

Supplementary Figure 44: Mass spectra of species formed between10 mM **2b** and 20 mM amino acid mixture of R and Dpr (each at 10 mM) shown for: A) **2b-Dpr** or **2b-n-β-Dpr** (retention time 6.16 min), B) **2b-R** (retention time 6.25 min), C) **2b-OH** (retention time 9.83 min), D) **2b-n-β-Dpr-Dpr-2b** or **2b-Dpr-n-β-Dpr-2b** (retention time 14.08 min) in Supplementary Figure 43, obtained from the LC-MS analysis.

Calculated m/z $[M+H]^+$: **2b-Dpr** or **2b-n-β-Dpr** 589.2657**, 2b-R** 659.3188**, 2b-OH** 503.2177, **2b-n-β-Dpr-Dpr-2b** or **2b-Dpr-n-β-Dpr-2b** 1073.4655

Observed m/z $[M+H]^+$: **2b-Dpr** or **2b-n-β-Dpr** 589.2661**, 2b-R** 659.3197**, 2b-n-β-Dpr-Dpr-2b** or **2b-Dpr-n-β-Dpr-2b** 1073.4668

$[M+Na]^+$: **2b-OH** 525.1996.

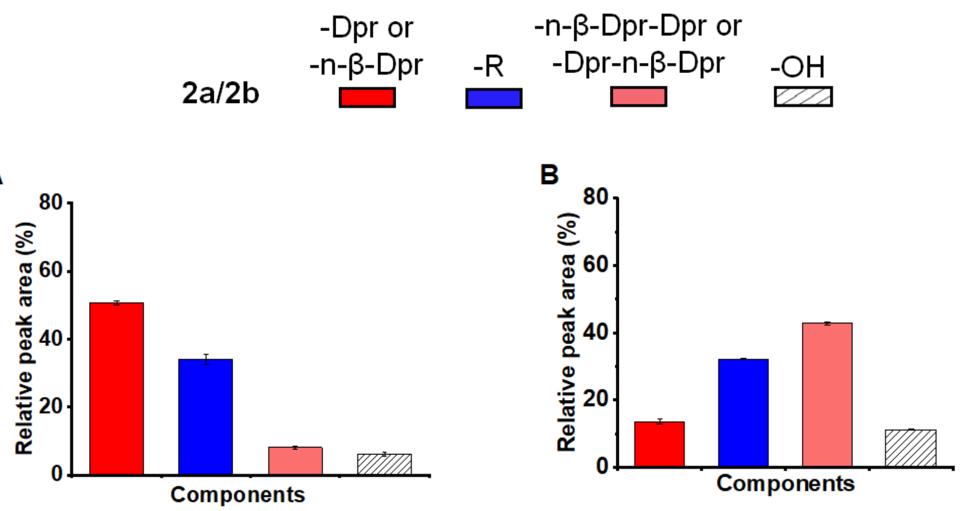

Supplementary Figure 45: Bar graphs showing peptide coupling from competition experiments between A) **2a**, B) **2b** with an amino acid mixture containing 10 mM R and 10 mM Dpr in 0.6 M borate buffer, pH 9.1. In each bar graph striped bar represent the hydrolysis product **2a-OH/2b-OH**. Error bars represent standard deviation from three independent experiments. Peptide coupling yields were measured for **2a**, after 30 minutes, while for **2b**, after 48 hours.

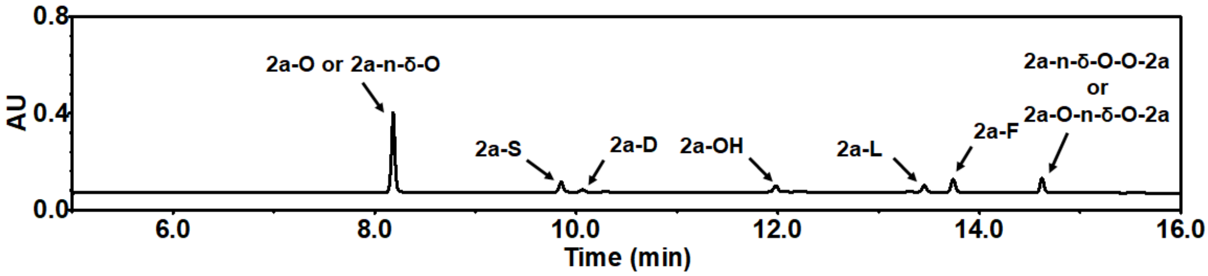

Supplementary Figure 46: UPLC chromatogram of reaction between 10 mM **2a** and 50 mM amino acid mixture (D, S, L, O, and F, each at 10 mM) in 0.6 M borate buffer, pH 9.1. Measurements were taken after 30 minutes.

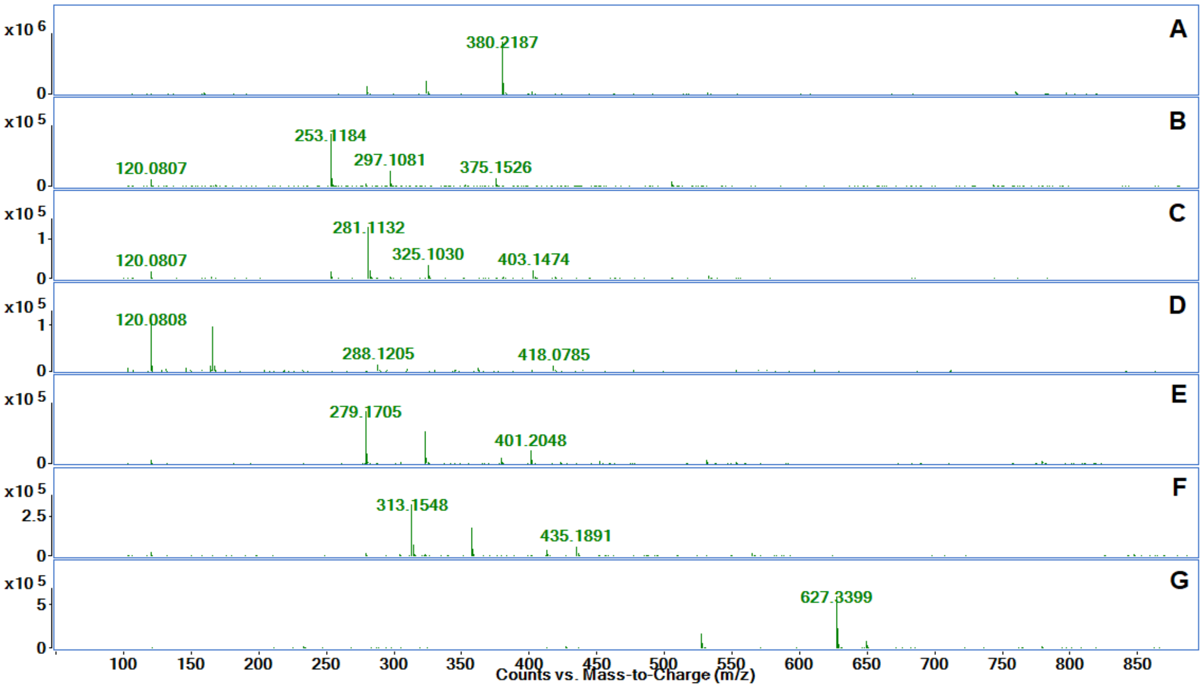

Supplementary Figure 47: Mass spectra of species formed between 10 mM **2a** and 50 mM amino acid mixture I (D, S, L, O, and F, each at 10 mM) shown for: A) **2a-O** or **2a-n-δ-O** (retention time 8.18 min), B) **2a-S** (retention time 9.85 min), C) **2a-D** (retention time 10.06 min), D) **2a-OH** (retention time 11.98 min), E) **2a-L** (retention time 13.45 min), F) **2a-F** (retention time13.74 min), G) **2a-n-δ-O-O-2a** or **2a-O-n-δ-O-2a** (retention time 14.62 min), in Supplementary Figure 46, obtained from the LC-MS analysis.

Calculated m/z $[M+H]^+$: **2a-O** or **2a-n-δ-O** 380.2120, **2a-S** 353.1707**, 2a-D** 381.1656**, 2a-OH** 266.1387, **2a-L** 379.2227, **2a-F** 413.2071, **2a-n-δ-O-O-2a** or **2a-O-n-δ-O-2a** 627.3388.
Observed m/z $[M+H]^+$: **2a-O** or **2a-n-δ-O** 380.2127, **2a-n-δ-O-O-2a** or **2a-O-n-δ-O-2a** 627.3399 $[M+Na]^+$: **2a-S** 375.1526**, 2a-D** 403.1474**, 2a-L** 401.2048, **2a-F** 435.1891, **2a-OH** 288.1205.

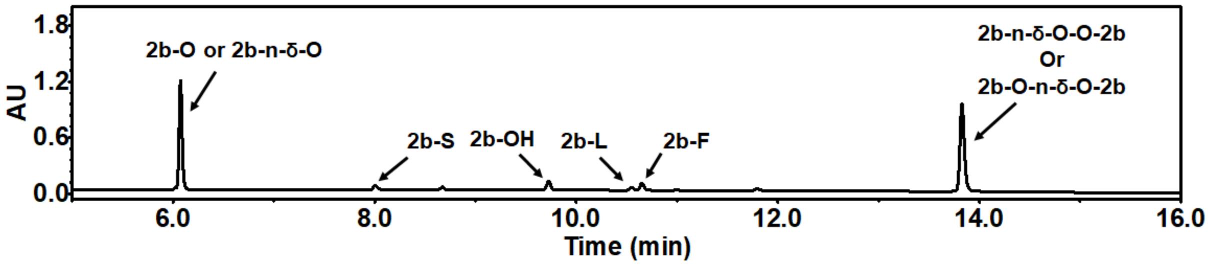

Supplementary Figure 48: UPLC chromatogram of reaction between 10 mM **2b** and 50 mM amino acid mixture (D, S, L, O, and F, each at 10 mM) in 0.6 M borate buffer, pH 9.1. Measurements were taken after 48 hours.

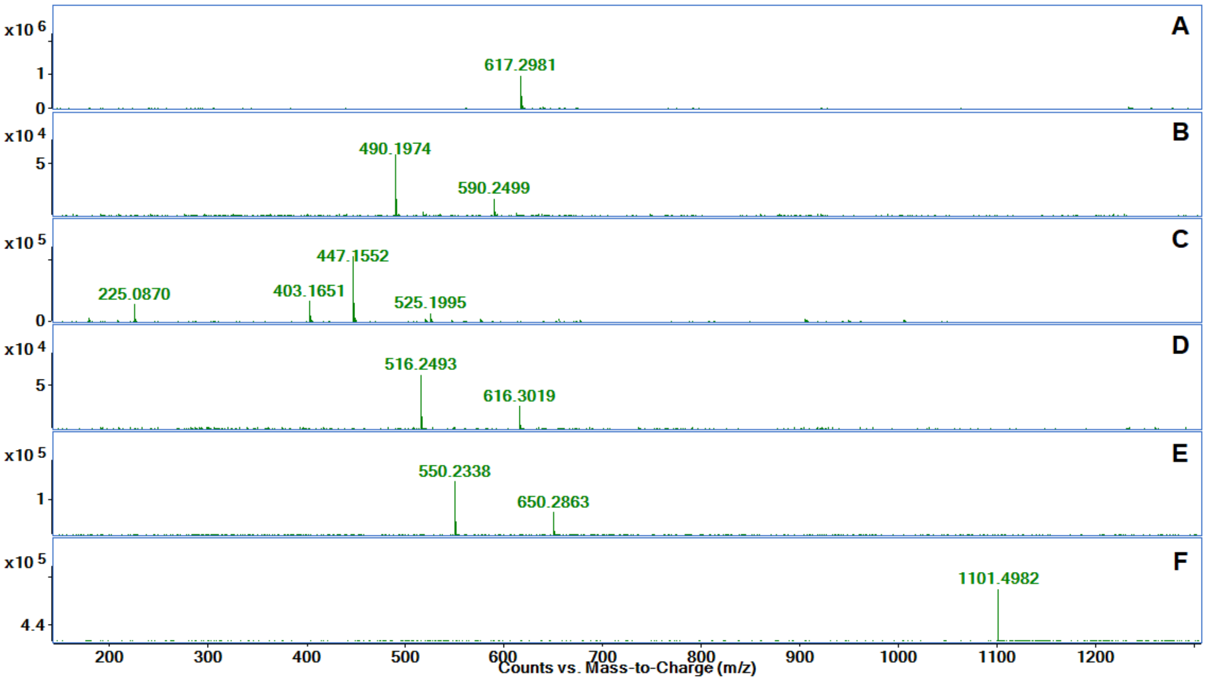

Supplementary Figure 49: Mass spectra of species formed between **2b** and amino acid mixture (D, S, L, O, and F, each at 10 mM), shown for: A) **2b-O** or **2b-n-δ-O** (retention time 6.07 min), B) **2b-S** (retention time 8.00 min), C) **2b-OH** (retention time 9.72 min), D) **2b-L** (retention time 10.55 min), E) **2b-F** (retention time 10.65 min), F) **2b-n-δ-O-O-2b** or **2b-O-n-δ-O-2b** (retention time 13.82 min) in Supplementary Figure 48, obtained from the LC-MS analysis.

Calculated m/z $[M+H]^+$: **2b-O** or **2b-n-δ-O** 617.2970, **2b-S** 590.2497**,** **2b-L** 616.3017**,** **2b-F** 650.2861, **2b-OH** 503.2177, **2b-n-δ-O-O-2b** or **2b-O-n-δ-O-2b** 1101.4968

Observed m/z $[M+H]^+$: **2b-O** or **2b-n-δ-O** 617.2981, **2b-S** 590.2499, **2b-L** 616.3019, **2b-F** 650.2863, **2b-n-δ-O-O-2b** or **2b-O-n-δ-O-2b** 1101.4962 $[M+Na]^+$: **2b-OH** 525.1995.

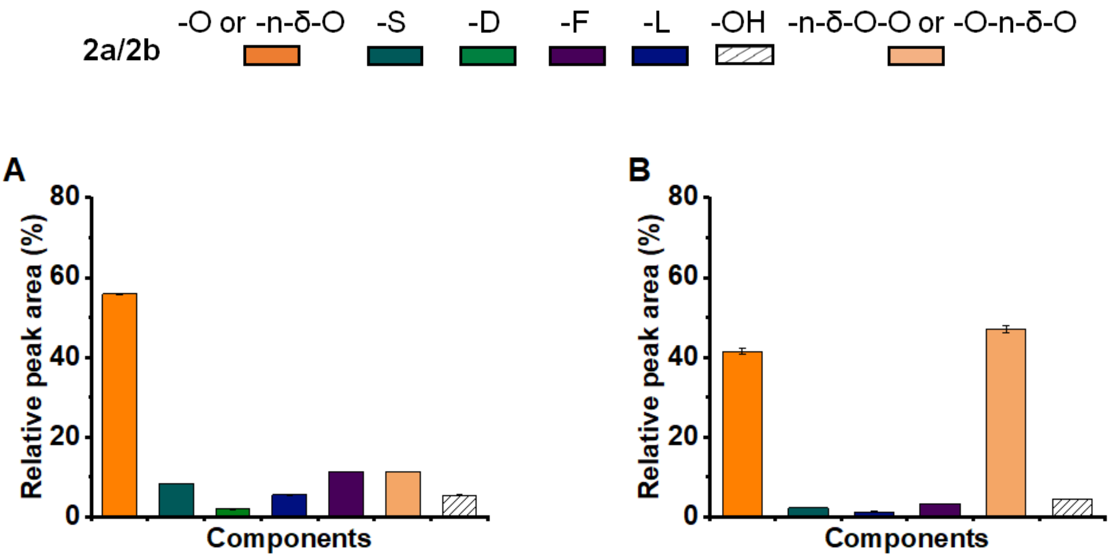

Supplementary Figure 50: Bar graphs showing peptide coupling between A) 10 mM **2a** or B) 10 mM **2b** with 50 mM amino acid mixture (D, S, L, O, F, each amino acid is 10 mM), in 0.6 M borate buffer, pH 9.1. In each bar graph striped bar represent the hydrolysis product **2a-OH**/**2b-OH**. Error bars represent standard deviation from three independent experiments. Peptide coupling yields were measured for **2a** after 30 minutes, while for **2b** after 48 hours.

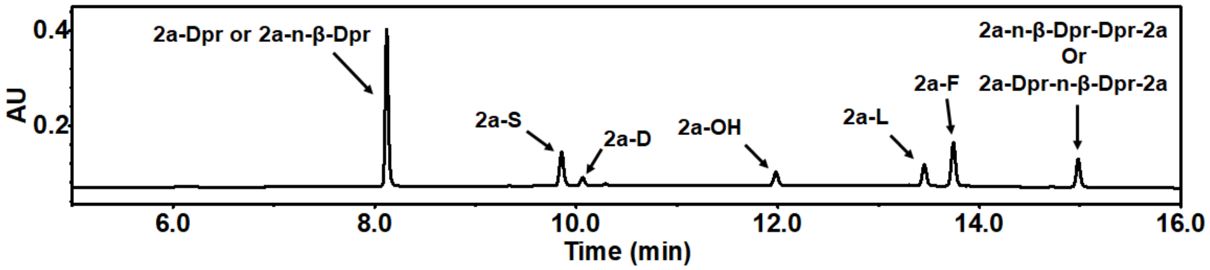

Supplementary Figure 51: UPLC chromatogram of reaction between 10 mM **2a** and 50 mM amino acid mixture (D, S, L, Dpr, and F, each at 10 mM) in 0.6 M borate buffer, pH 9.1 Measurements were taken after 60 minutes.

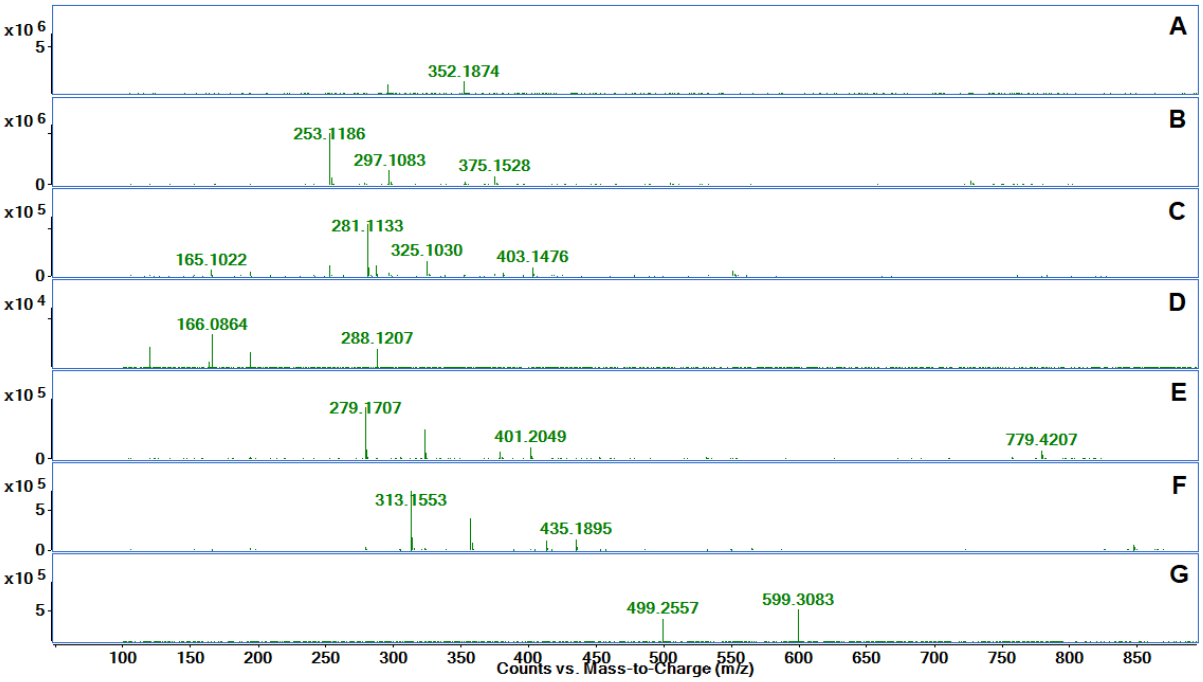

Supplementary Figure 52: Mass spectra of species formed between 10 mM **2a** and 50 mM amino acid mixture I (D, S, L, Dpr, and F, each at 10 mM) shown for: A) **2a-Dpr** or **2a-n-β-Dpr** (retention time 8.11 min), B) **2a-S** (retention time 9.85 min), C) **2a-D** (retention time 10.06 min), D) **2a-OH** (retention time 11.98 min), E) **2a-L** (retention time 13.45 min), F) **2a-F** (retention time13.74 min), G) **2a-n-β-Dpr-Dpr-2a** or **2a-Dpr-n-β-Dpr-2a** (retention time 14.98 min) in Supplementary Figure 51, obtained from the LC-MS analysis.

Calculated m/z $[M+H]^+$: **2a-Dpr** or **2a-n-β-Dpr** 352.1867, **2a-S** 353.1707**, 2a-D** 381.1656**, 2a-OH** 266.1387, **2a-L** 379.2227, **2a-F** 413.2071, **2a-n-β-Dpr-Dpr-2a** or **2a-Dpr-n-β-Dpr-2a** 599.3075.

Observed m/z $[M+H]^+$: **2a-Dpr** or **2a-n-β-Dpr** 352.1874**, 2a-n-β-Dpr-Dpr-2a** or **2a-Dpr-n-β-Dpr-2a** 599.3083 $[M+Na]^+$: **2a-S** 375.1528**, 2a-D** 403.1476**, 2a-L** 401.2049, **2a-F** 435.1895, **2a-OH** 288.1207.

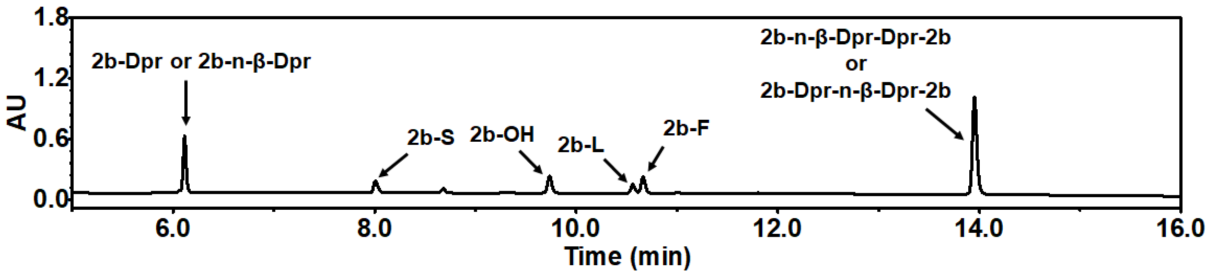

Supplementary Figure 53: UPLC chromatogram of reaction between 10 mM **2b** and 50 mM amino acid mixture (D, S, L, Dpr, and F, each at 10 mM) in 0.6 M borate buffer, pH 9.1. Measurements were taken after 48 hours.

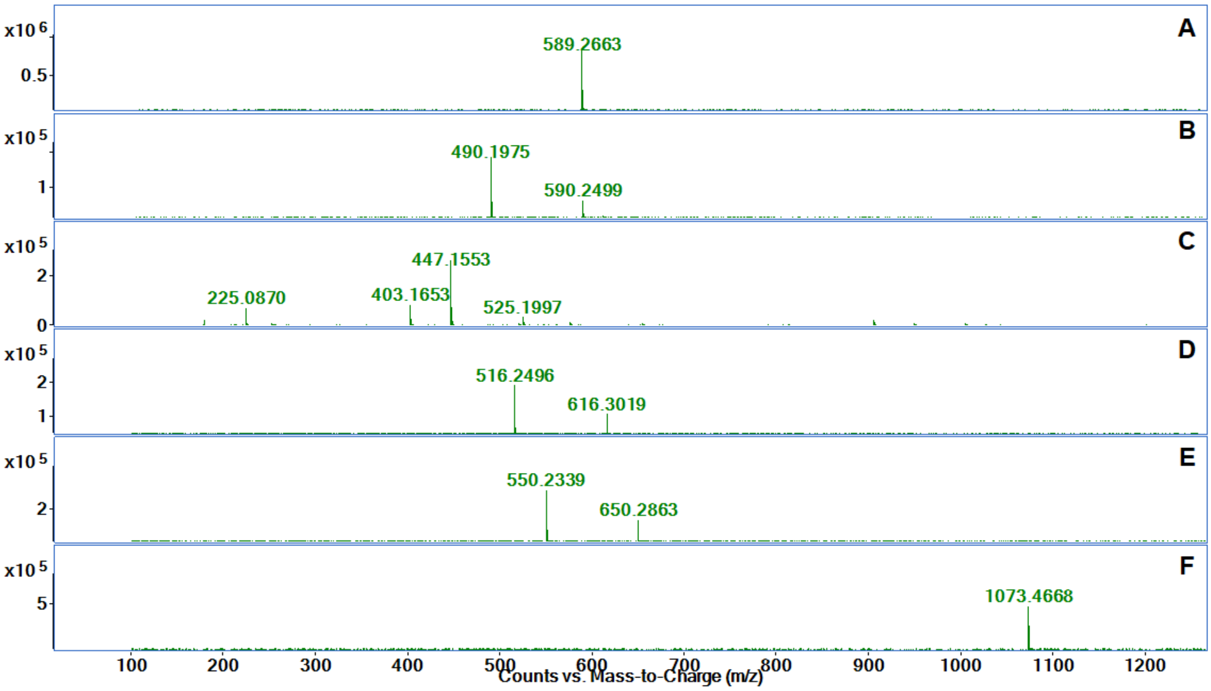

Supplementary Figure 54: Mass spectra of species formed between **2b** and amino acid mixture (D, S, L, Dpr, and F, each at 10 mM), shown for: A) **2b-Dpr** or **2b-n-β-Dpr** (retention time 6.11 min), B) **2b-S** (retention time 8.00 min), C) **2b-OH** (retention time 9.73 min), D) **2b-L** (retention time 10.56 min), E) **2b-F** (retention time 10.66 min), F) **2b-n-β-Dpr-Dpr-2b** or **2b-Dpr-n-β-Dpr-2b** (retention time 13.95 min) in Supplementary Figure 53, obtained from the LC-MS analysis.

Calculated m/z $[M+H]^+$: **2b-Dpr** or **2b-n-β-Dpr** 589.2657, **2b-S** 590.2497**,** **2b-L** 616.3017**,** **2b-F** 650.2861, **2b-OH** 503.2177, **2b-n-β-Dpr-Dpr-2b** or **2b-Dpr-n-β-Dpr-2b** 1073.4655

Observed m/z $[M+H]^+$: **2b-Dpr** or **2b-n-β-Dpr** 589.2663, **2b-S** 590.2499, **2b-L** 616.3019, **2b-F** 650.2863, **2b-n-β-Dpr-Dpr-2b** or **2b-Dpr-n-β-Dpr-2b** 1073.4668

$[M+Na]^+$: **2b-OH** 525.1997.

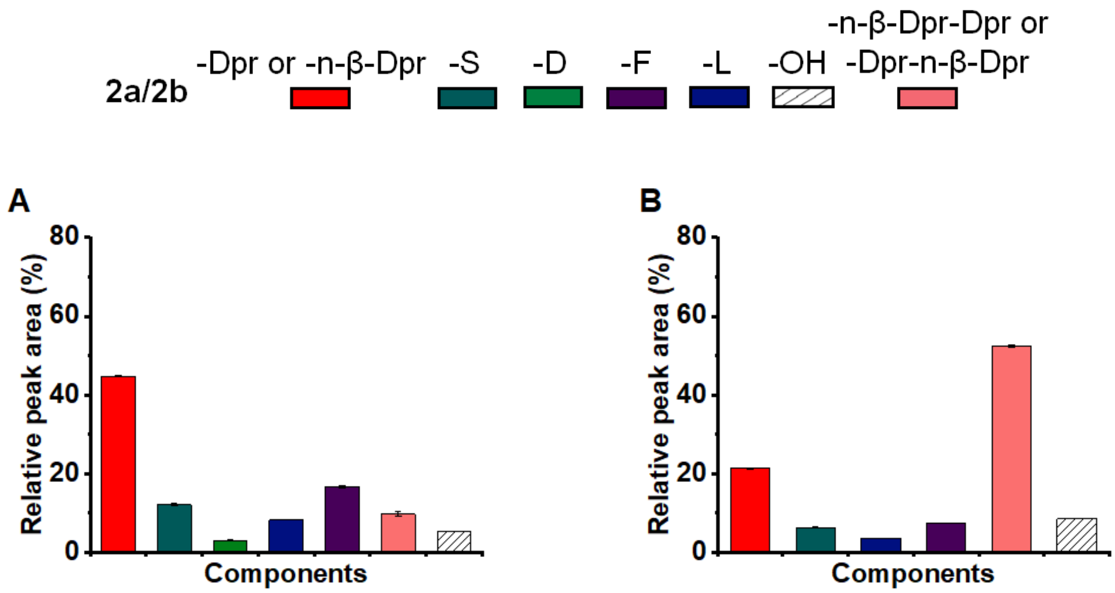

Supplementary Figure 55: Bar graphs showing peptide coupling between A) 10 mM **2a** and B) 10 mM **2b** with 50 mM amino acid mixture (D, S, L, Dpr, and F, each amino acid is 10 mM), in 0.6 M borate buffer, pH 9.1. In each bar graph striped bar represent the hydrolysis product **2a-OH/2b-OH**. Error bars represent standard deviation from three independent experiments. Peptide coupling yields were measured for **2a** after 30 minutes, while for **2b** after 48 hours.

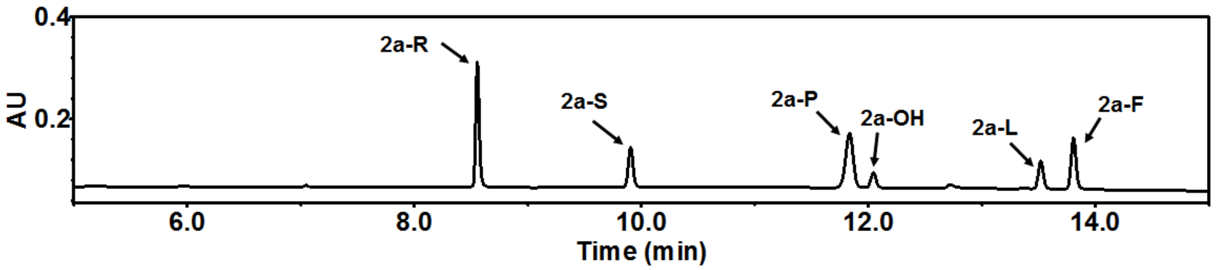

Supplementary Figure 56: UPLC chromatogram of reaction between 10 mM **2a** and 50 mM amino acid mixture (P, S, L, R, and F, each at 10 mM) in 0.6 M borate buffer, pH 9.1. Measurements were taken after 30 minutes.

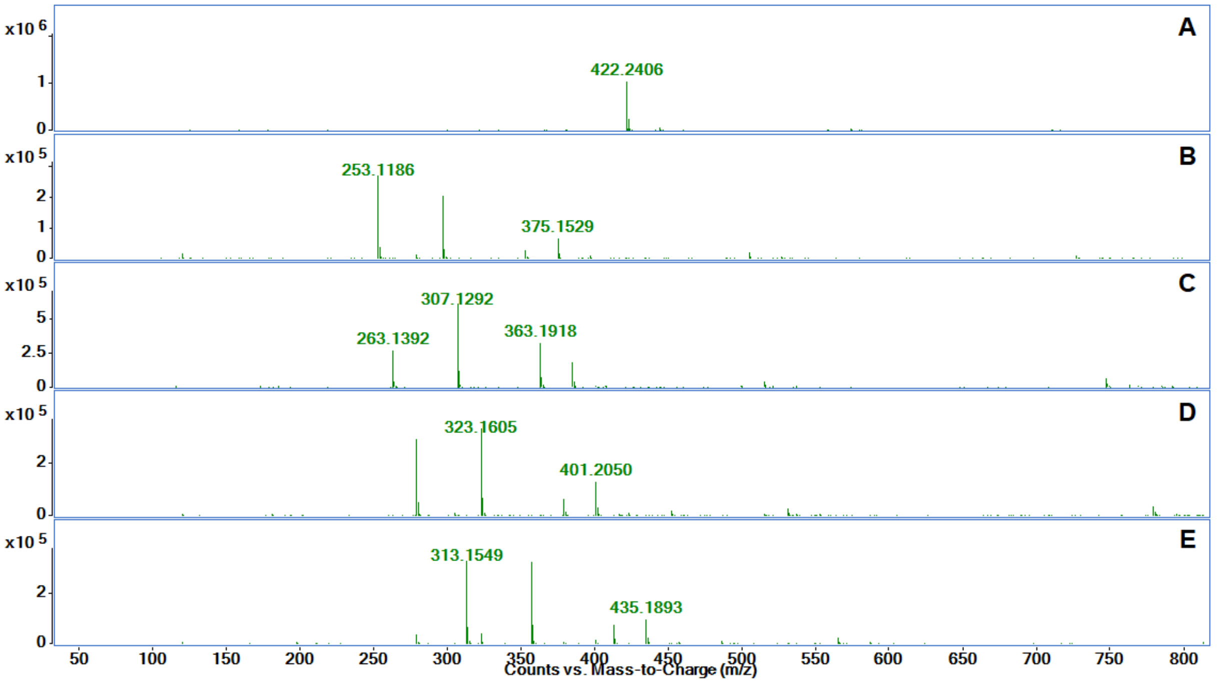

Supplementary Figure 57: Mass spectra of species formed between 10 mM **2a** and 50 mM amino acid mixture I (P, S, L, R, and F, each at 10 mM) shown for: A) **2a-R** (retention time 8.55 min), B) **2a-S** (retention time 9.90 min), C) **2a-P** (retention time 11.83 min), D) **2a-L** (retention time 13.51 min), E) **2a-F** (retention time13.80 min), in Supplementary Figure 56, obtained from the LC-MS analysis.

Calculated m/z $[M+H]^+$: **2a-R** 422.2398, **2a-S** 353.1707**, 2a-P** 363.1914**, 2a-L** 379.2227, **2a-F** 413.2071.

Observed m/z $[M+H]^+$: **2a-R** 422.2406, **2a-P** 363.1918**,** $[M+Na]^+$: **2a-S** 375.1529**, 2a-L** 401.2050, **2a-F** 435.1893.

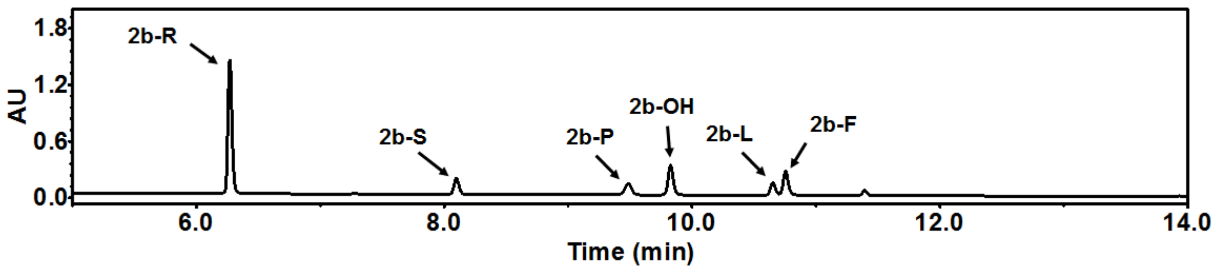

Supplementary Figure 58: UPLC chromatogram of reaction between 10 mM **2b** and 50 mM amino acid mixture (P, S, L, R, and F, each at 10 mM) in 0.6 M borate buffer, pH 9.1. Measurements were taken after 48 hours.

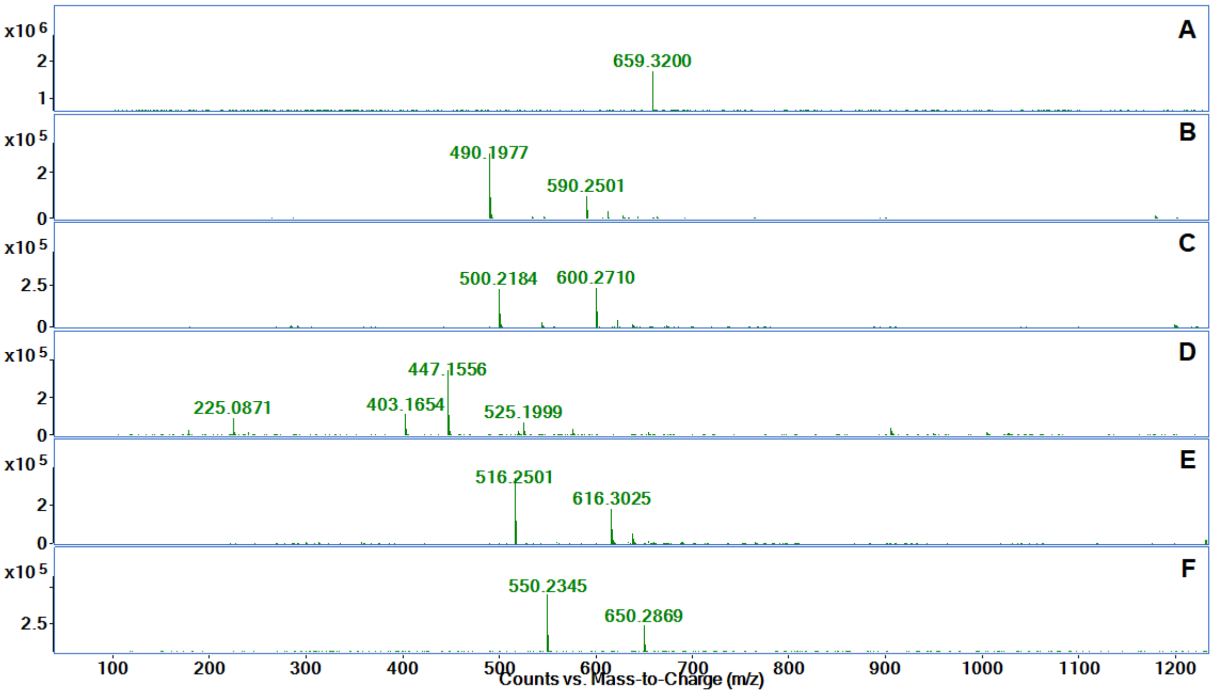

Supplementary Figure 59: Mass spectra of species formed between **2b** and amino acid mixture (P, S, L, R, and F, each at 10 mM), shown for: A) **2b-R** (retention time 6.27 min), B) **2b-S** (retention time 8.09 min), C) **2b-P** (retention time 9.48 min) D) **2b-OH** (retention time 9.82 min), E) **2b-L** (retention time 10.65 min), F) **2b-F** (retention time 10.75 min) in Supplementary Figure 58, obtained from the LC-MS analysis.

Calculated m/z $[M+H]^+$: **2b-R** 659.3188, **2b-S** 590.2497, **2b-P** 600.2704, **2b-L** 616.3017, **2b-F** 650.2861, **2b-OH** 503.2177

Observed m/z $[M+H]^+$: **2b-R** 659.3200, **2b-S** 590.2501, **2b-P** 600.2710, **2b-L** 616.3025, **2b-F** 650.2869, $[M+Na]^+$: **2b-OH** 525.1999.

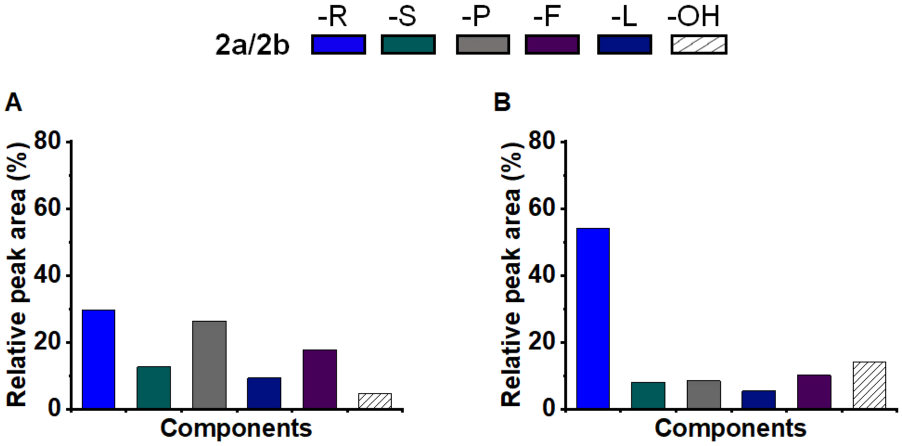

Supplementary Figure 60: Bar graphs showing peptide coupling between A) 10 mM **2a** and B) 10 mM **2b** with 50 mM amino acid mixture (P, S, R, F, L, each amino acid is 10 mM), in 0.6 M borate buffer, pH 9.1. In each bar graph striped bar represent the hydrolysis product **2a-OH**/**2b-OH**. Peptide coupling yields were measured for **2a** after 30 minutes, while for **2b** after 48 hours.

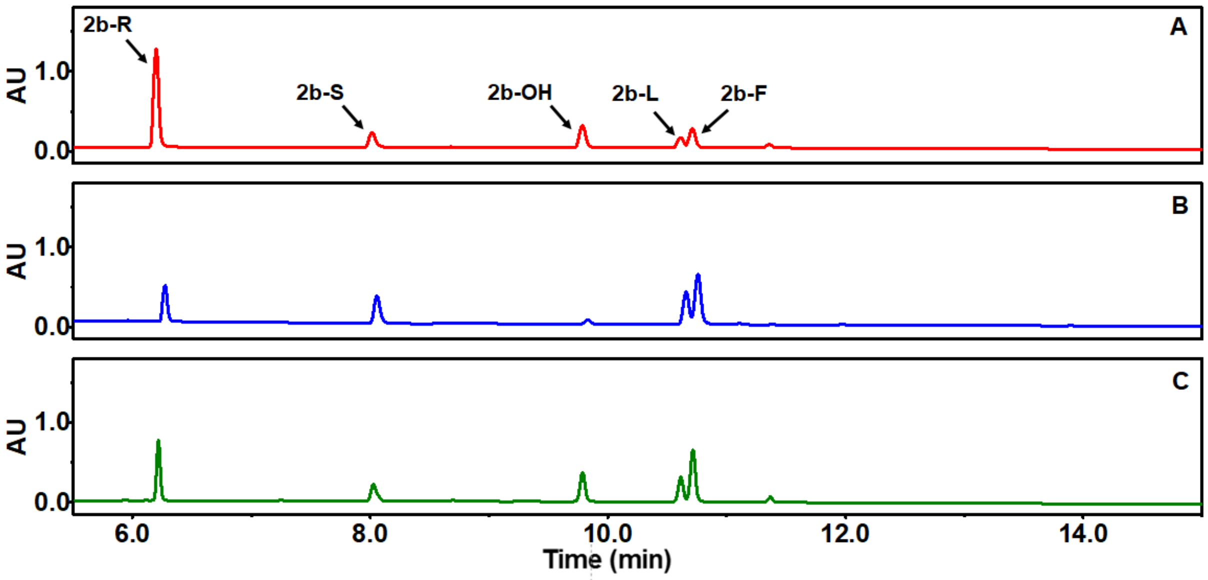

Supplementary Figure 61: UPLC chromatograms of reactions between 10 **2b** and 50 mM amino acid mixture I (D+S+L+R+F**,** each amino acid at 10 mM), A) in 0.6 M borate buffer, pH 9.1, B) in 80% ACN and C) with 1M Na_2SO_4 in 0.6 M borate buffer, pH 9.1. Measurements were taken after 48 hours.

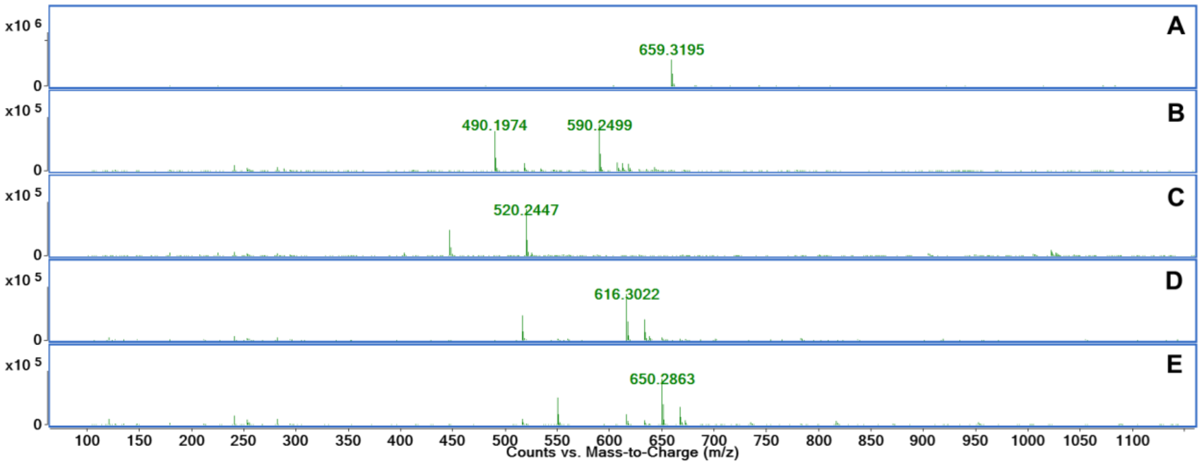

Supplementary Figure 62: Mass spectra of species formed between **2b** and amino acid mixture I, shown for: A) **2b-R** (retention time 6.19 min), B) **2b-S** (retention time 8.01 min), C) **2b-OH** (retention time 9.78 min), D) **2b-L** (retention time 10.61 min), E) **2b-F** (retention time 10.71min) in Supplementary Figure 61 A, obtained from the LC-MS analysis.

Calculated m/z $[M+H]^+$: **2b-R** 659.3188, **2b-S** 590.2497**, 2b-L** 616.3017**, 2b-F** 650.2861 **2b-OH** 503.2177

Observed m/z $[M+H]^+$: **2b-R** 659.3195, **2b-S** 590.2499, **2b-L** 616.3023, **2b-F** 650.2865, $[M+H_2O]$: **2b-OH** 520.2447.

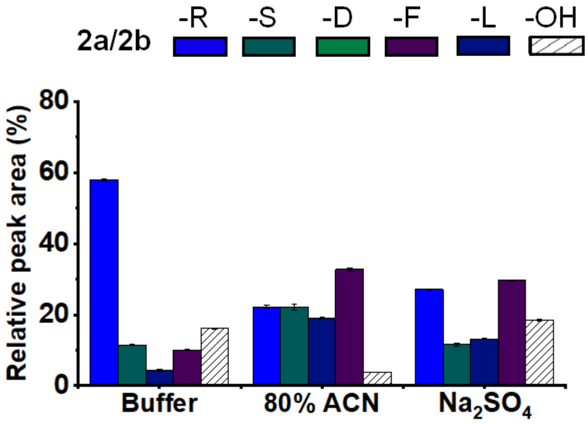

Supplementary Figure 63: Bar graph showing comparison of peptide coupling between 10 **2b** and 50 mM amino acid mixture I (D+S+L+R+F**,** each amino acid at 10 mM), in 0.6 M borate buffer, pH 9.1, in 80% ACN and with 1M Na_2SO_4 in 0.6 M borate buffer, pH 9.1. Peptide coupling yields were measured after 48 hours.

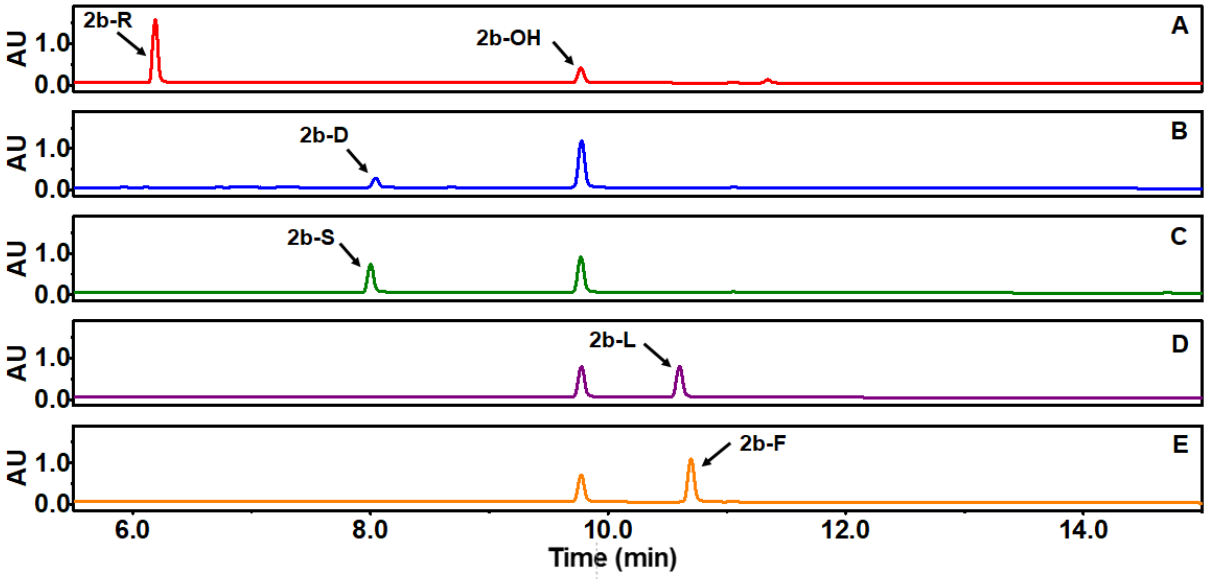

Supplementary Figure 64: UPLC chromatograms of reactions between 10 mM **2b** and A) 10 mM R, B) 10 mM D, C) 10 mM S, D) 10 mM L and E) 10 mM F in 0.6 M borate buffer, pH 9.1. Measurements were taken after 48 hours.

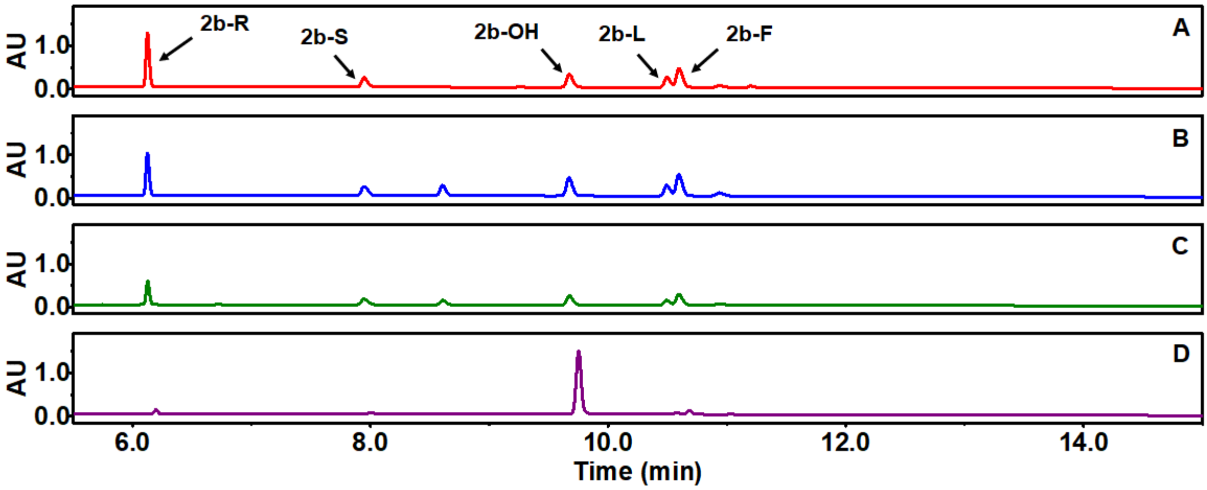

Supplementary Figure 65: UPLC chromatograms of reactions between 10 mM **2b** and 50 mM amino acid mixture-I (D, S, L, R, F**,** each amino acid at 10 mM) in the presence of different salts: A) 1M NaCl B) 1M Guanidinium chloride, C) 1M Guanidinium thiocynate and D) 1M $MgCl_2$ in 0.6 M borate buffer, pH 9.1. Measurements were taken after 48 hours.

Supplementary Table 1: Effect of different salts on peptide coupling between 10 mM **2b** and 50 mM amino acid mixture 1 (D, S, L, R, F**,** each amino acid at 10 mM), in 0.6 M borate buffer, pH 9.1. Peptide coupling is a measure of relative peak area %.

**Salt**	**Peptide coupling (%)**				
	**2b-R**	**2b-S**	**2b-L**	**2b-F**	**2b-OH**
1M NaCl	40.19	12.61	9.16	20.4	17.64
1M Guanidine.HCl	27.05	11.74	8.47	20.57	20.98
1M Guanidine.SCN	28.62	14.12	7.05	19.81	20.92
1M $MgCl_2$	4.03	2.12	2.23	4.68	86.94
1M Na_2SO_4	26.94	12.25	12.87	29.78	18.16

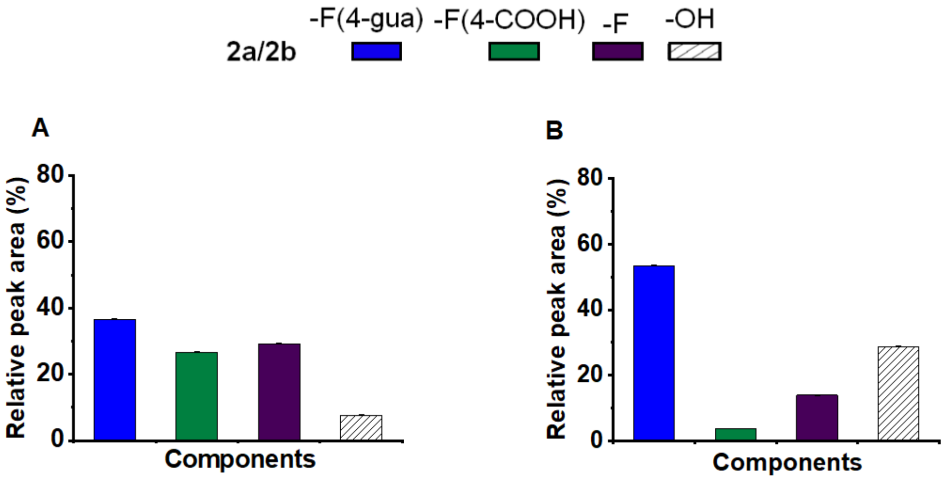

Supplementary Figure 66: Bar graph showing peptide coupling between A) 10 mM **2a** or B) 10 mM **2b** with 30 mM amino mixture II (F(4-Gua), F, F(4-COOH)**,** each amino acid at 10 mM), in 0.6 M borate buffer, pH 9.1. Peptide coupling yields were measured for **2a** after 30 minutes, while for **2b** after 48 hours.

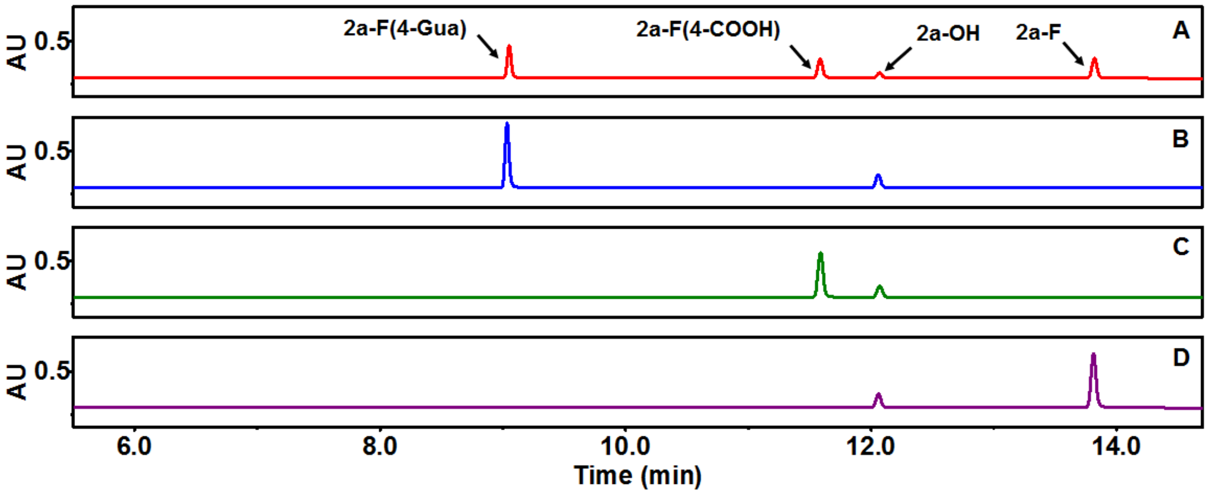

Supplementary Figure 67: UPLC chromatograms of reactions between 10 mM **2a** and A) and 30 mM amino acid mixture II ((F(4-Gua), F, F(4-COOH), each amino acid at 10 mM), B) 10 Mm F(4-Gua), C) 10 mM F(4-COOH), D) 10 mM F, in 0.6 M borate buffer, pH 9.1. Measurements were taken after 30 minutes.

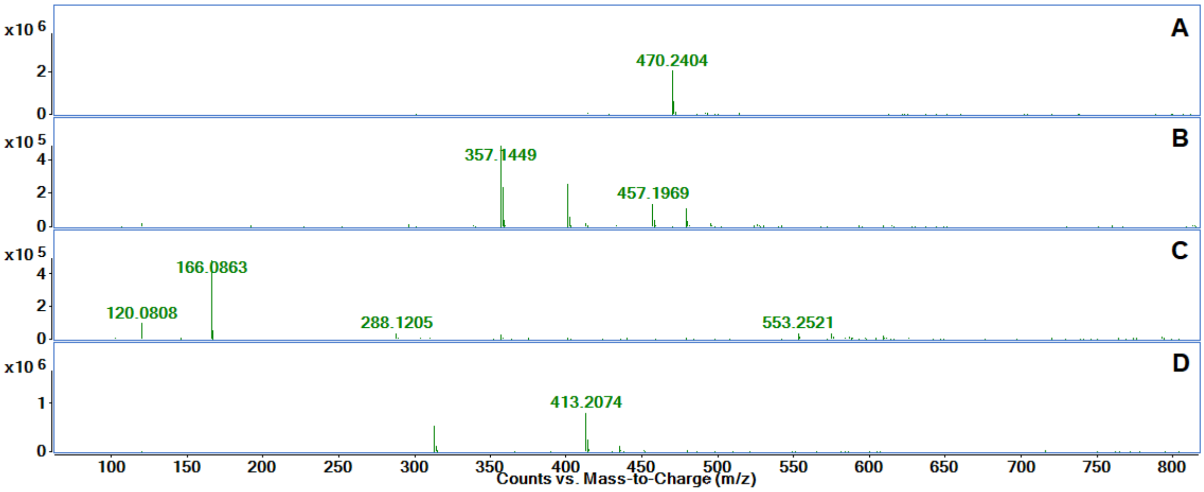

Supplementary Figure 68: Mass spectra of species formed between 10 mM **2a** and 30 mM amino acid mixture II amino acid mixture II (F(4-Gua), F, F(4-COOH)), each amino acid at 10 mM), shown for: A) **2a-F(4-Gua)** (retention time 9.05 min), B) **2a-F(4-COOH)** (retention time 11.58 min), C) **2a-OH** (retention time 12.06 min) and D) **2a-F** (retention time 13.82 min) in Supplementary Figure 67A, obtained from the LC-MS analysis.

Calculated m/z $[M+H]^+$: **2a-F(4-Gua)** 470.2398 **2a-F(4-COOH)** 457.1969 **2a-OH** 266.1387, **2a-F** 413.2071.

Observed m/z $[M+H]^+$: **2a-F(4-Gua)** 470.2404 **2a-F(4-COOH)** 457.1969, **2a-F** 413.2074, $[M+Na]^+$: **2a-OH** 288.1205

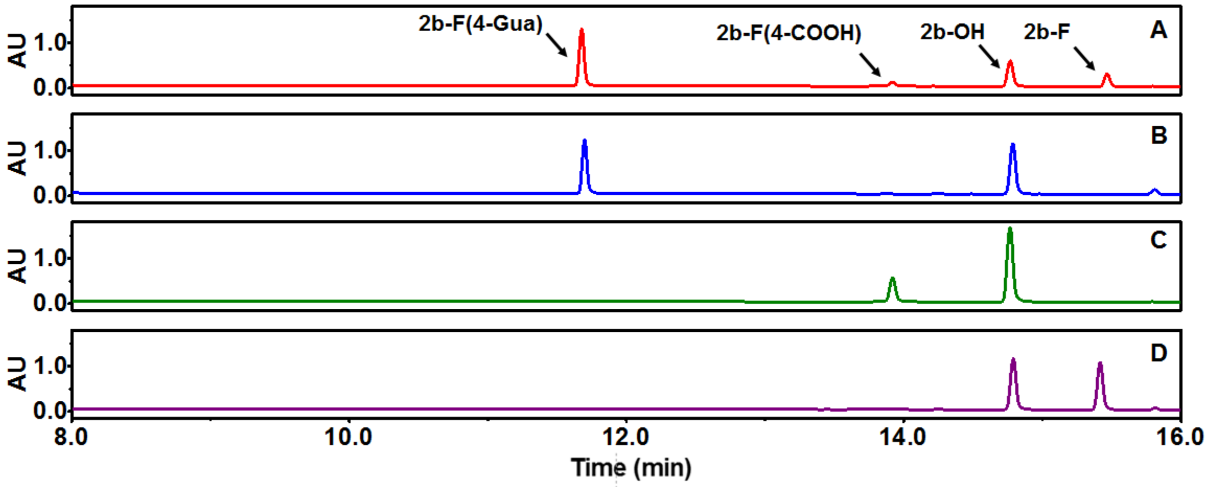

Supplementary Figure 69: UPLC chromatograms of reactions between 10 mM **2b** and A) 30 mM amino mixture II amino acid mixture II ((F(4-Gua), F, F(4-COOH))**,** each amino acid at 10 mM)**,** B) 10 Mm F(4-Gua), C) 10 mM F(4-COOH), and D) 10 mM F, in 0.6 M borate buffer, pH 9.1. Measurements were taken after 48 hours.

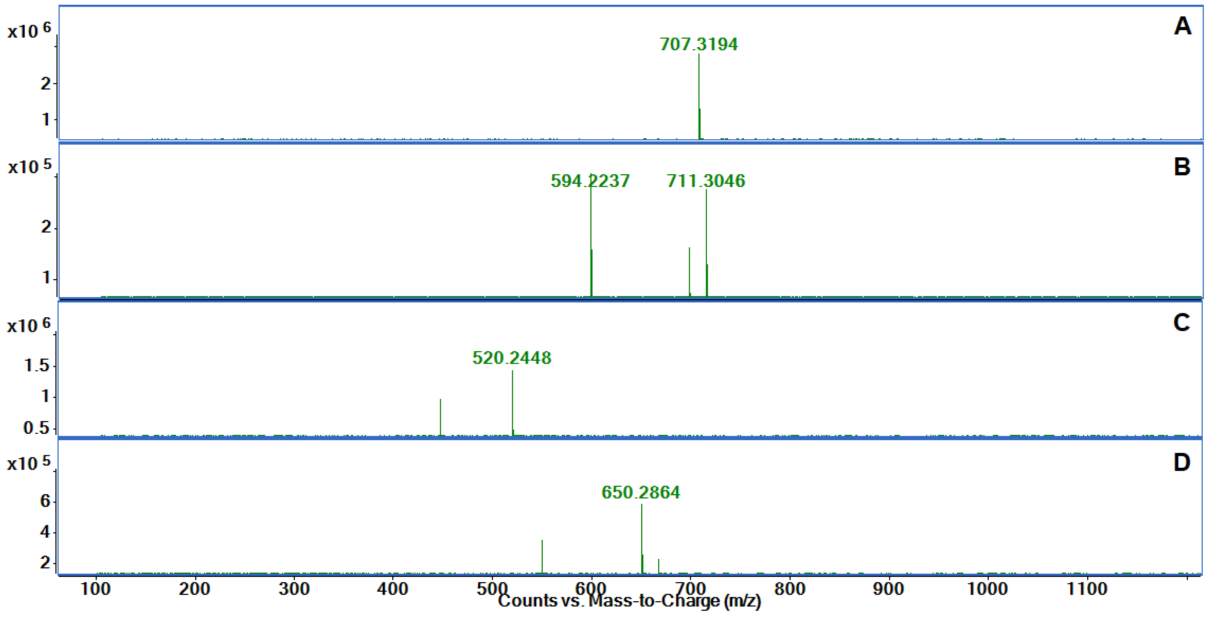

Supplementary Figure 70: Mass spectra of species formed between 10 mM **2b** and 30 mM amino acid mixture II amino acid mixture II (F(4-Gua), F, F(4-COOH), each amino acid is 10 mM) A) **2b-F(4-Gua)** (retention time 11.67 min), B) **2b-F(4-COOH)** (retention time 13.92 min), C) **2b-OH** (retention time 13.99 min), D) **2b-F** (retention time 14.76 min) in Supplementary Figure 69A, obtained from the LC-MS analysis.

Calculated m/z $[M+H]^+$: **2b-F(4-Gua)** 707.3188**, 2b-F(4-COOH)** 694.2759, **2b-F** 650.2861, **2b-OH** 503.2177.

Observed m/z $[M+H]^+$: **2b-F(4-Gua)** 707.3194**, 2b-F** 650.2864, **2b-OH** 503.2177, $[M+H_2O]$: **2b-F(4-COOH)** 711.3046, **2b-OH** 520.2448.

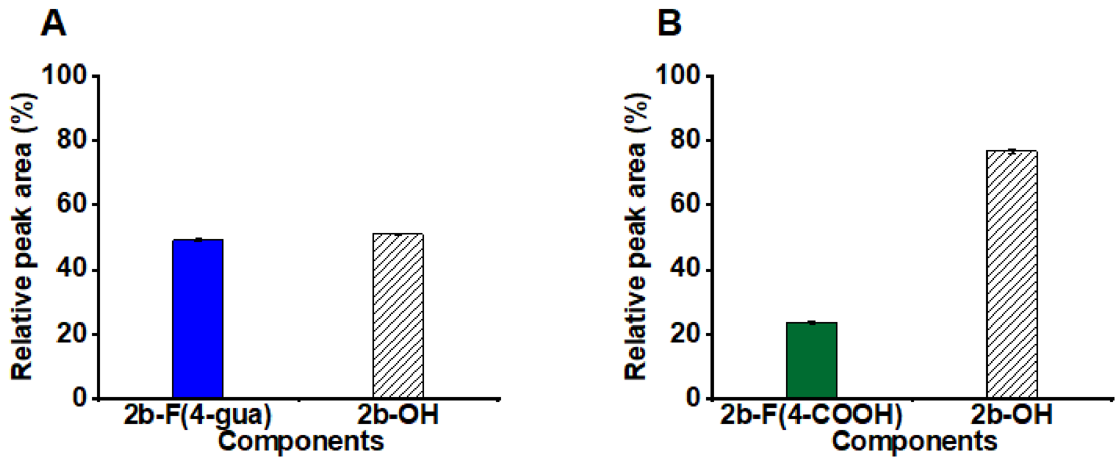

Supplementary Figure 71: Bar graph showing peptide conversion between 10 mM **2b** and A) 10 Mm F(4-Gua), B) 10 mM F(4-COOH) in 0.6 M borate buffer, pH 9.1. In bar graphs striped bar represent the hydrolysis product **2b-OH**. Error bars represent standard deviation from three independent experiments. Peptide coupling yields were measured after 48 hours.

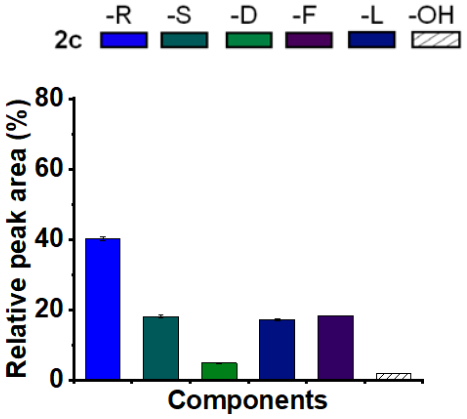

Supplementary Figure 72: Bar graphs showing peptide coupling between 10 mM **2c** and 50 mM amino acid mixture - I (D, S, L, R, F, each amino acid is 10 mM), in 0.6 M borate buffer, pH 9.1. The striped bar represents the hydrolysis product **2c-OH**. Error bars represent standard deviation from three independent experiments. Peptide coupling yields were measured after 60 minutes.

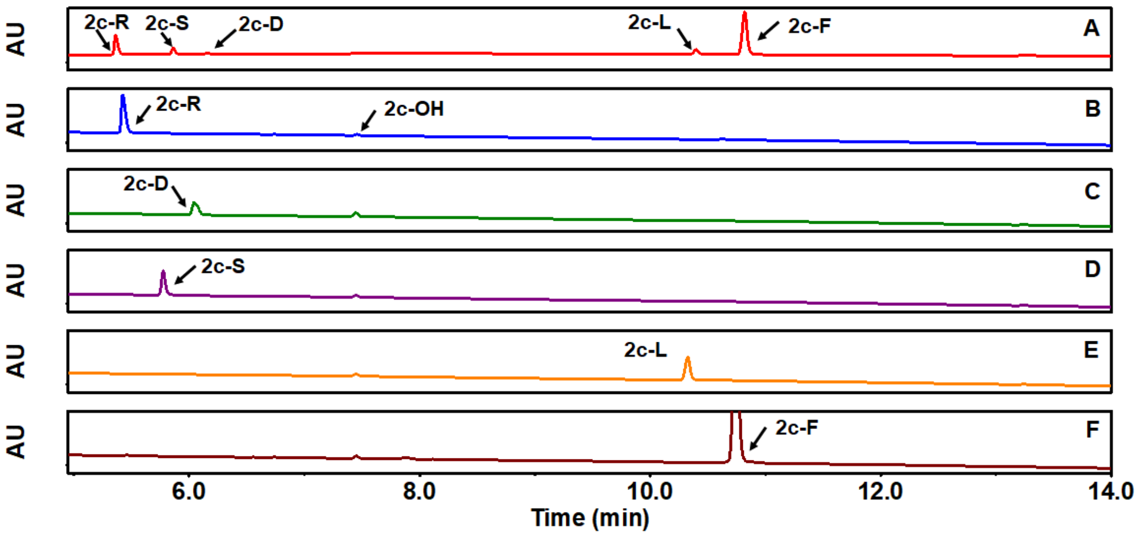

Supplementary Figure 73: UPLC chromatogram of reaction between 10 mM **2c** and A) 50 mM amino acid mixture I (D, S, L, R, and F, each at 10 mM), B) 10 mM R, C) 10 mM D, D) 10 mM S, E) 10 mM L and F) 10 mM F in 0.6 M borate buffer, pH 9.1. Measurements were taken after 60 minutes.

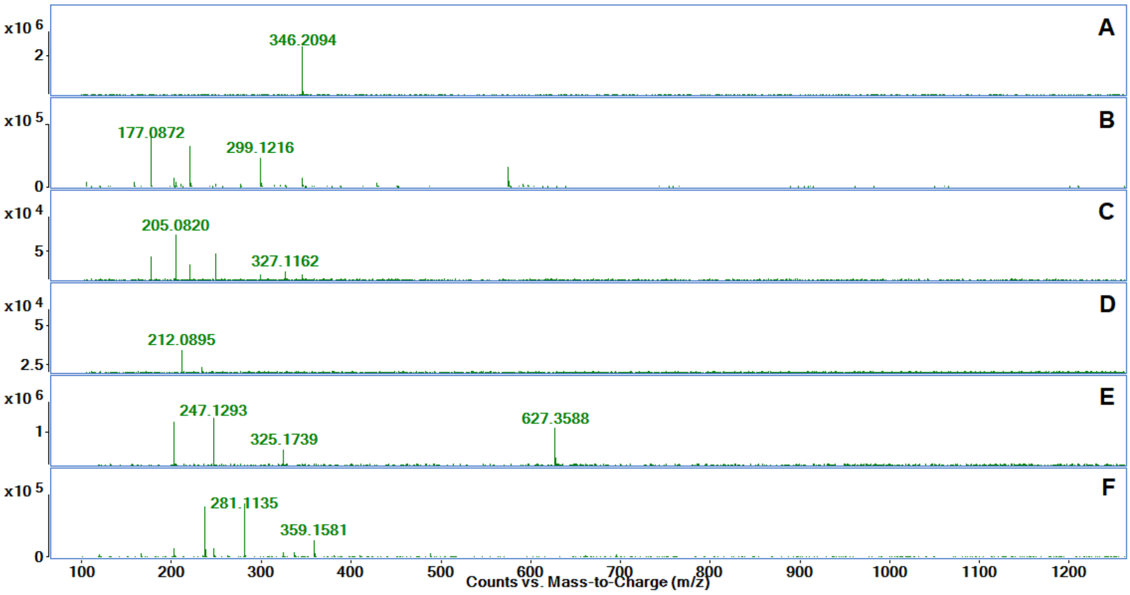

Supplementary Figure 74: Mass spectra of species formed between **2c** and amino acid mixture I, shown for: A) **2c-R** (retention time 5.30 min), B) **2c-S** (retention time 5.83 min), C) **2c-D** (retention time 6.11 min), D) **2c-OH** (retention time 7.50 min), E) **2c-L** (retention time 10.37 min), F) **2c-F** (retention time 10.79 min) in Supplementary Figure 73 A, obtained from the LC-MS analysis.

Calculated m/z $[M+H]^+$: **2c-R** 346.2085, **2c-S** 277.1394**, 2c-D** 327.1163, **2c-L** 303.1914, **2c-F** 337.1758, **2c-OH** 190.1074

Observed m/z $[M+H]^+$: **2c-R** 346.2094, **2c-D** 327.1162, $[M+Na]^+$: **2c-S** 299.1216, **2c-L** 325.1739, **2c-F** 359.158, **2c-OH** 212.0895.

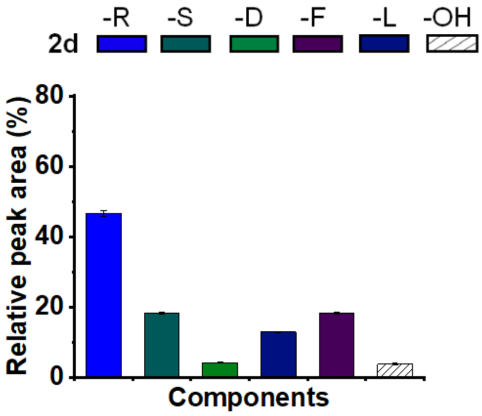

Supplementary Figure 75: Bar graphs showing peptide coupling between 10 mM **2d** and 50 mM amino acid mixture - I (D, S, L, R, F, each amino acid is 10 mM), in 0.6 M borate buffer, pH 9.1. The striped bar represents the hydrolysis product **2d-OH**. Error bars represent standard deviation from three independent experiments. Peptide coupling yields were measured after 12 hours.

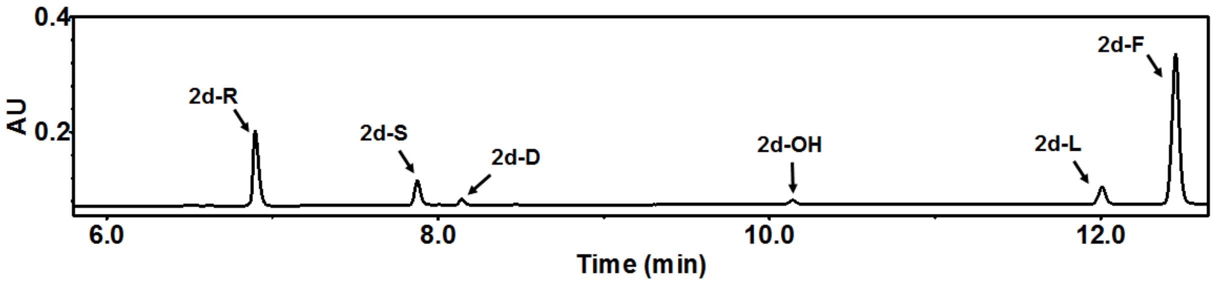

Supplementary Figure 76: UPLC chromatogram of reaction between 10 mM **2d** and 50 mM amino acid mixture I (D, S, L, R, and F, each at 10 mM), in 0.6 M borate buffer, pH 9.1. Measurements were taken after 12 hours.

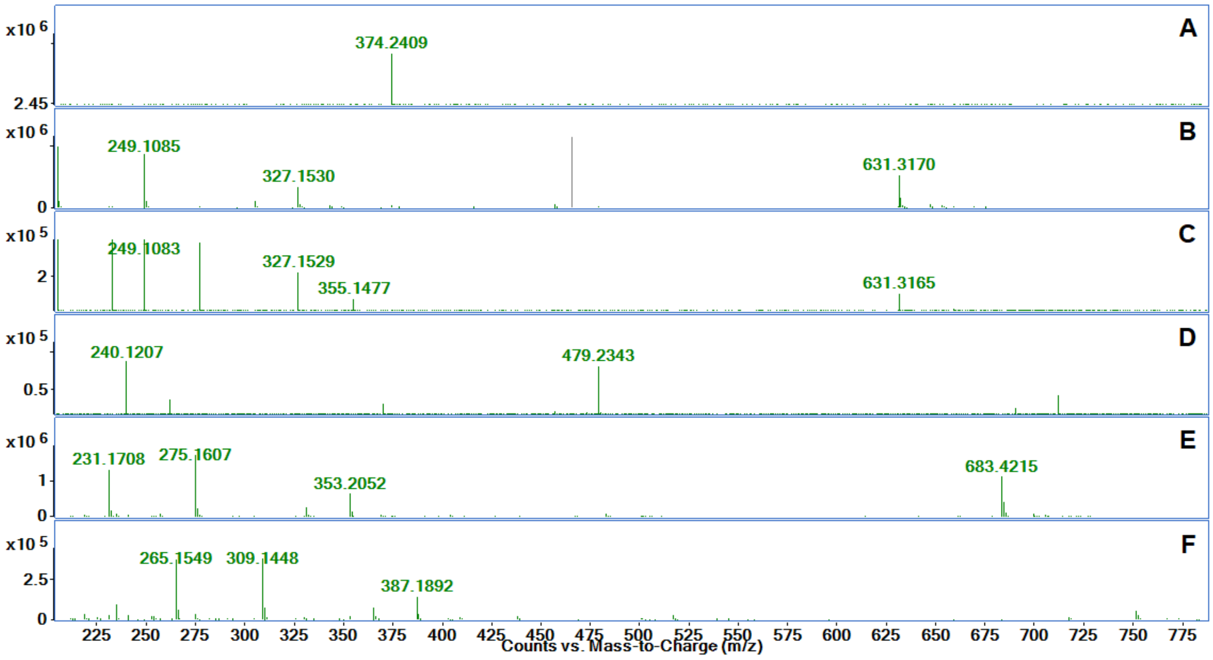

Supplementary Figure 77: Mass spectra of species formed between **2d** and amino acid mixture I, shown for: A) **2d-R** (retention time 6.85 min), B) **2d-S** (retention time 7.85 min), C) **2d-D** (retention time 8.11 min), D) **2d-OH** (retention time10.11 min), E) **2d-L** (retention time 11.98 min), F) **2d-F** (retention time 12.42 min) in Supplementary Figure 76, obtained from the LC-MS analysis.

Calculated m/z $[M+H]^+$: **2d-R** 374.2398, **2d-S** 305.1707**, 2d-D** 333.1656, **2d-OH** 218.1387, **2d-L** 331.2227, **2d-F** 356.2071

Observed m/z $[M+H]^+$: **2d-R** 374.2409, $[M+Na]^+$: **2d-S** 327.1530, **2d-D** 355.1477, **2d-OH** 240.1207, **2d-L** 353.2052, **2d-F** 387.1892.

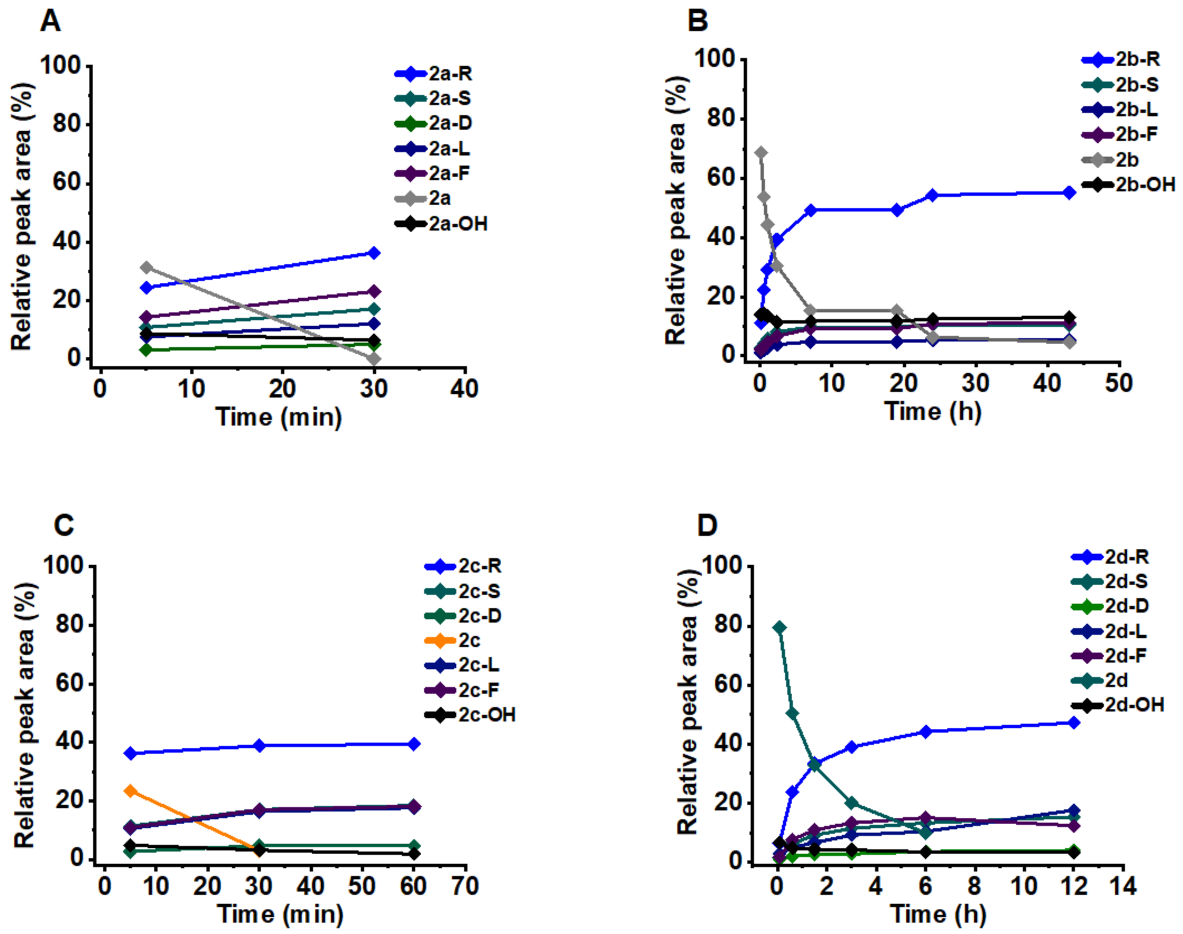

Supplementary Figure 78: Time-dependent peptide bond formation between A) **2a**, B) **2b**, C) **2c**, D) **2d** with amino acids mixture I (D, S, L, R, F) in 0.6 M borate buffer, pH 9.1. The concentration used for **2a**, **2b**, **2c** and **2d** was 10 mM, while the total concentration for the amino acids in the mixture was 50 mM (10 mM each).

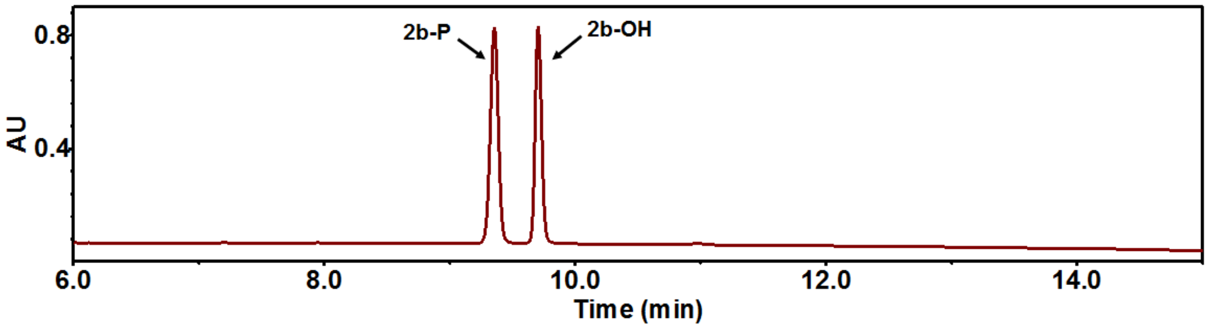

Supplementary Figure 79: UPLC chromatogram of reaction between 10 mM **2b** and 10 mM P in 0.6 M borate buffer, pH 9.1. Measurements were taken after 48 hours.

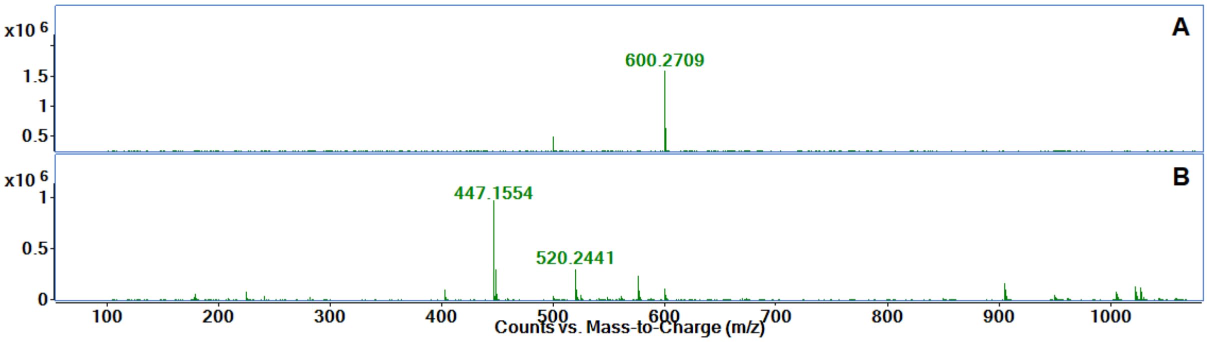

Supplementary Figure 80: Mass spectra of species formed between 10 mM **2b and** 10 mM P**,** shown for: A) **2b-P** (retention time 9.35 min), B) **2b-OH** (retention time 9.70 min) in Supplementary Figure 79, obtained from the LC-MS analysis.

Calculated m/z $[M+H]^+$: **2b-P** 600.2704, **2b-OH** 503.2177.

Observed m/z $[M+H]^+$: **2b-P** 600.2709, $[M+H_2O]$: **2b-OH** 520.2441.

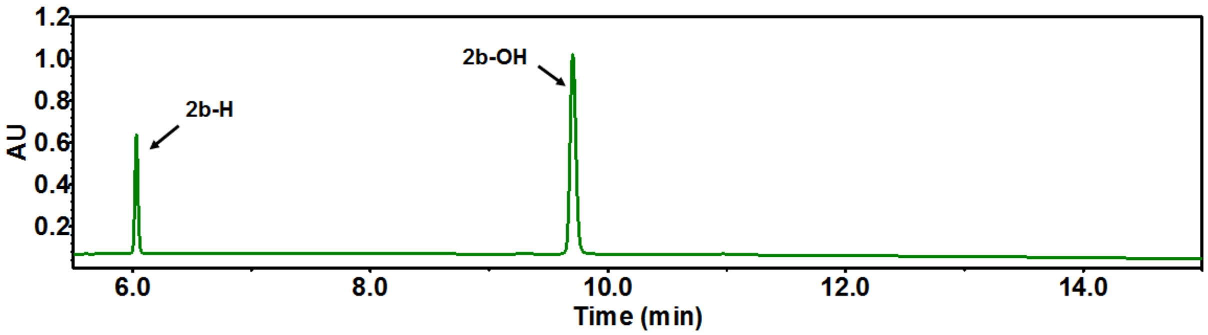

Supplementary Figure 81: UPLC chromatogram of reaction between 10 mM **2b** and 10 mM H in 0.6 M borate buffer, pH 9.1. Measurements were taken after 48 hours.

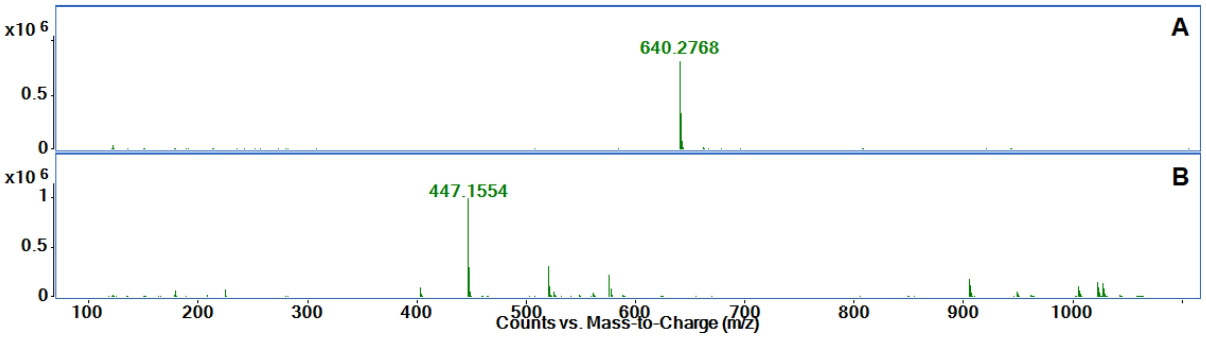

Supplementary Figure 82: Mass spectra of species formed between 10 mM **2b** and 10 mM H, shown for: A) **2b-H** (retention time 6.02 min), B) **2b-OH** (retention time 9.70 min) in Supplementary Figure 81, obtained from the LC-MS analysis.

Calculated m/z $[M+H]^+$: **2b-H** 640.2766**, 2b-OH** 503.2177.

Observed m/z $[M+H]^+$: **2b-H** 640.2768, $[M+H_2O]$: **2b-OH** 520.2441.

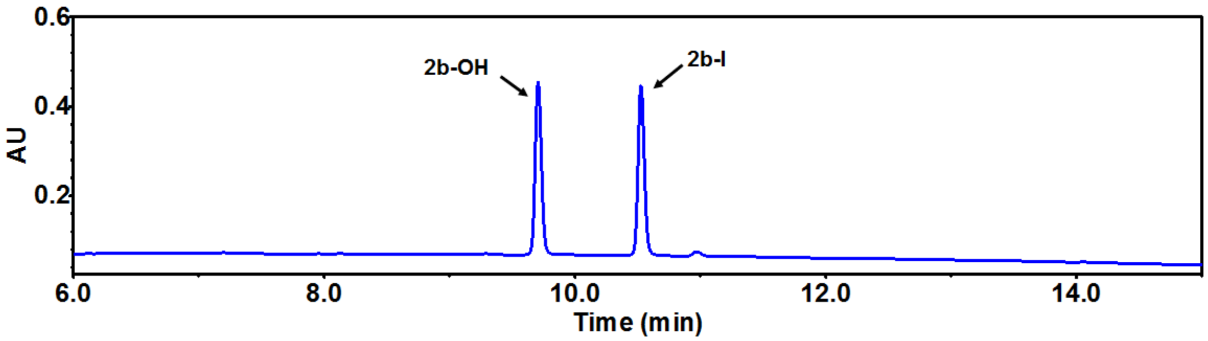

Supplementary Figure 83: UPLC chromatogram of reaction between 10 mM **2b** and 10 mM I in 0.6 M borate buffer, pH 9.1. Measurements were taken after 48 hours.

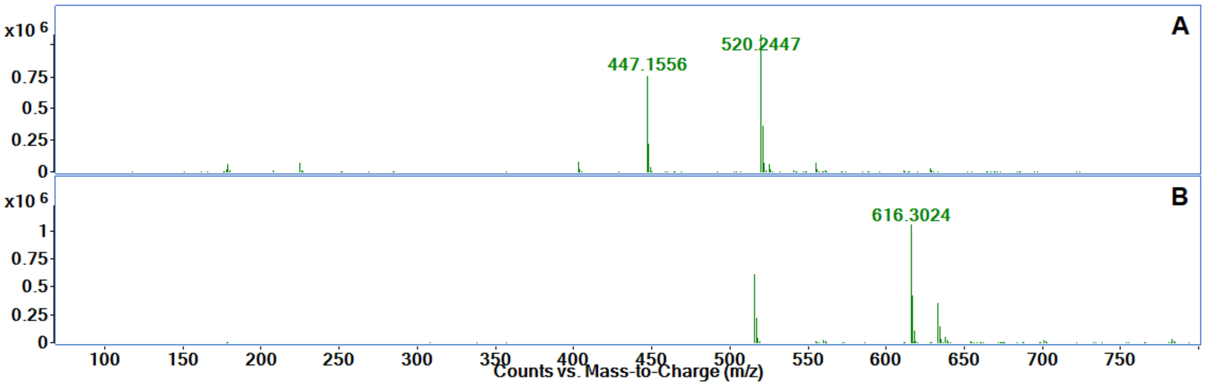

Supplementary Figure 84: Mass spectra of species formed between 10 mM **2b** and 10 mM I, shown for: A) **2b-OH** (retention time 9.70 min), B) **2b-I** (retention time 10.52 min), in Supplementary Figure 83 obtained from the LC-MS analysis.

Calculated m/z $[M+H]^+$: **2b-I** 616.3017**, 2b-OH** 503.2177.
Observed m/z $[M+H]^+$: **2b-I** 616.3024, $[M+H_2O]$: **2b-OH** 520.2447.

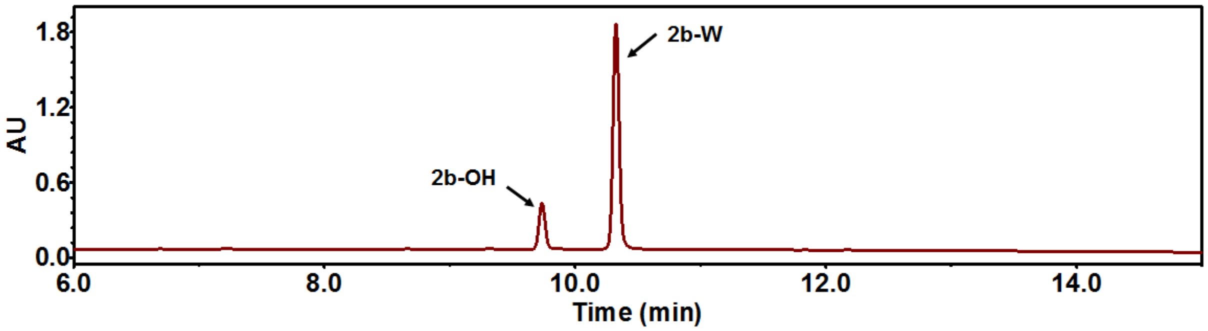

Supplementary Figure 85: UPLC chromatogram of reaction between 10 mM **2b** and 10 mM W in 0.6 M borate buffer, pH 9.1. Measurements were taken after 48 hours.

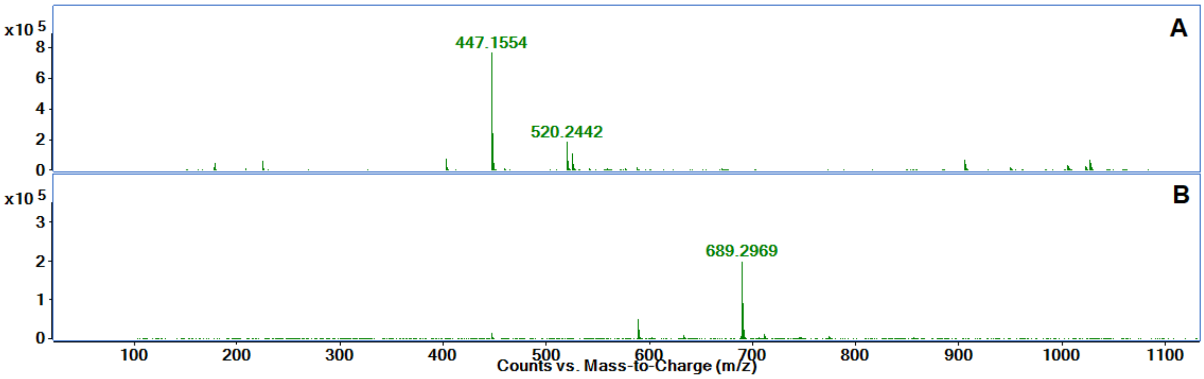

Supplementary Figure 86: Mass spectra of species formed between 10 mM **2b** and 10 mM W, shown for: A) **2b-OH** (retention time 9.70 min), B) **2b-W** (retention time 10.29 min), in Supplementary Figure 85, obtained from the LC-MS analysis.

Calculated m/z $[M+H]^+$: **2b-W** 689.2970**, 2b-OH** 503.2177.

Observed m/z $[M+H]^+$: **2b-W** 689.2969, $[M+H_2O]$: **2b-OH** 520.2442.

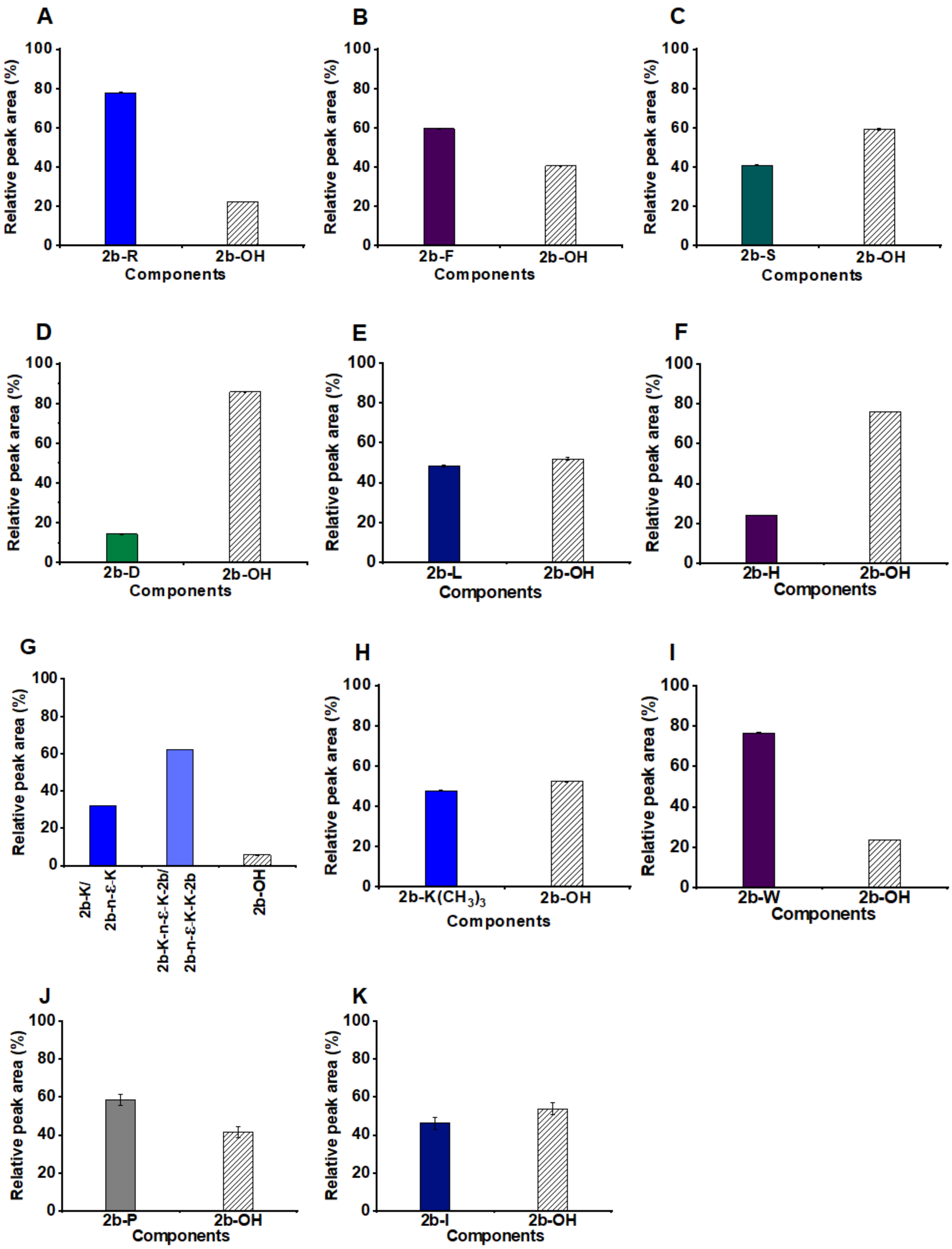

Supplementary Figure 87: Bar graphs showing peptide conversion between 10 mM **2b** and, A) 10 mM R, B) 10 mM F, C) 10 mM S, D) 10 mM D, E) 10 mM L, F) 10 mM H, G) 10 mM K, H) 10 mM $K(CH_3)_3$, I) 10 mM W, J) 10 mM P, K), 10 mM I in 0.6 M borate buffer, pH 9.1. In each bar graph striped bar represent the hydrolysis product **2b-OH**. Error bars represent standard deviation from three independent experiments. Peptide coupling yields were measured after 48 hours.

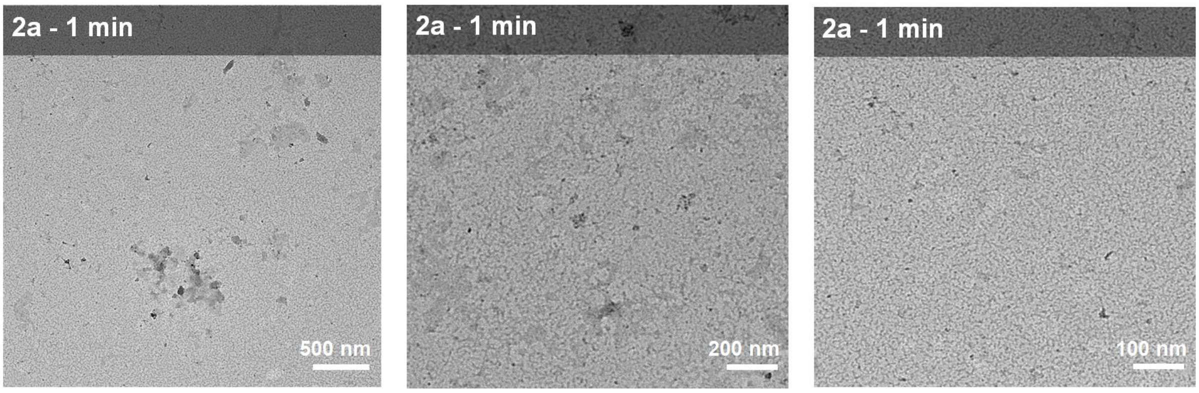

Supplementary Figure 88: TEM images of 10 mM **2a** measured at 1 minute after dissolving in 0.6 M borate buffer pH 9.1.

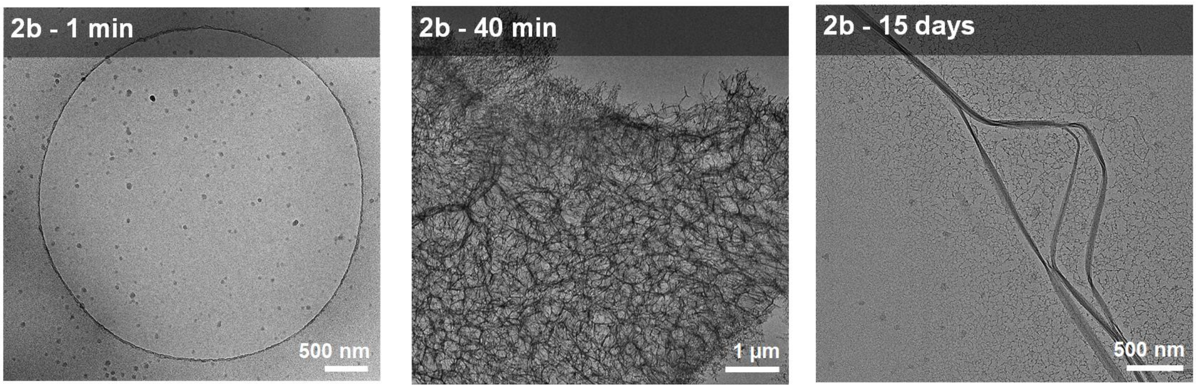

Supplementary Figure 89: Time-dependent cryo-TEM images of 10 mM **2b** in 0.6 M borate buffer pH 9.1.

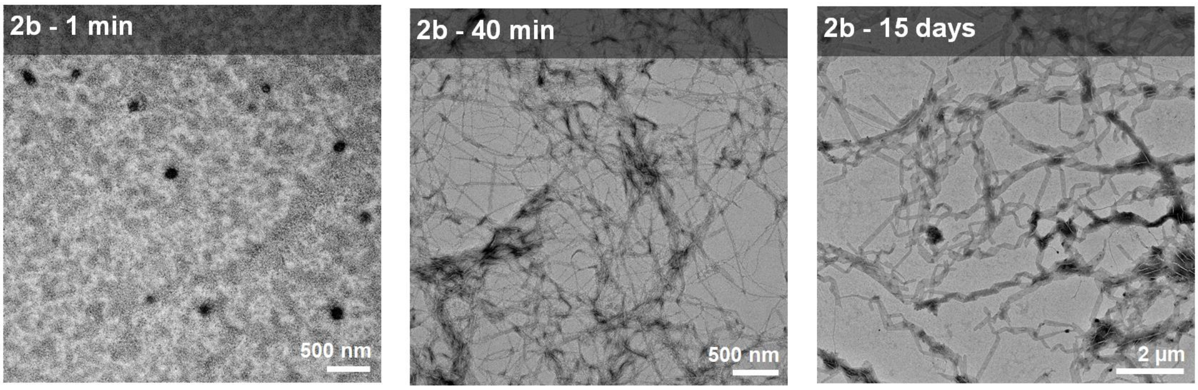

Supplementary Figure 90: Time-dependent TEM images of 10 mM **2b** in 0.6 M borate buffer pH 9.1.

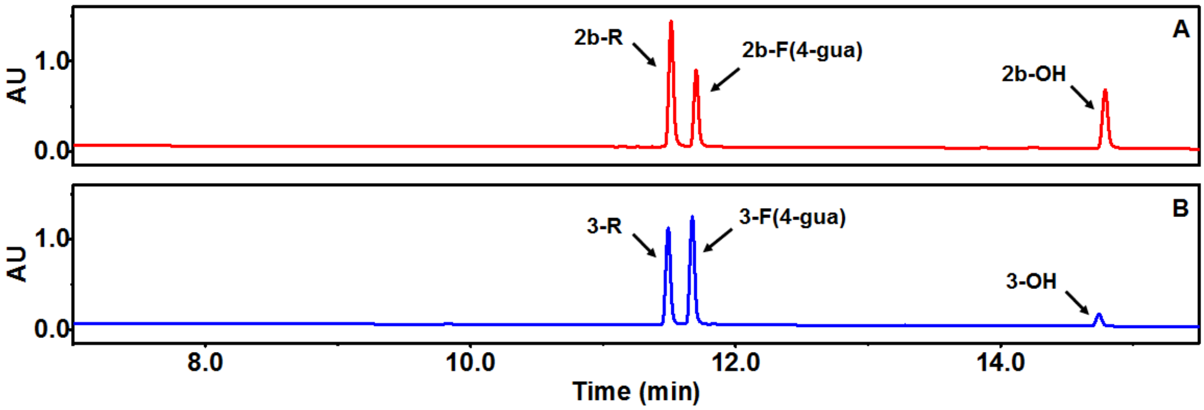

Supplementary Figure 91: UPLC chromatograms of reactions A) 10 mM **2b** and B) 10 mM **3** with 20 mM amino acid mixture (R, F(4-gua), each amino acid at 10 mM) in 0.6 M borate buffer, pH 9.1. Measurements were taken after 48 hours.

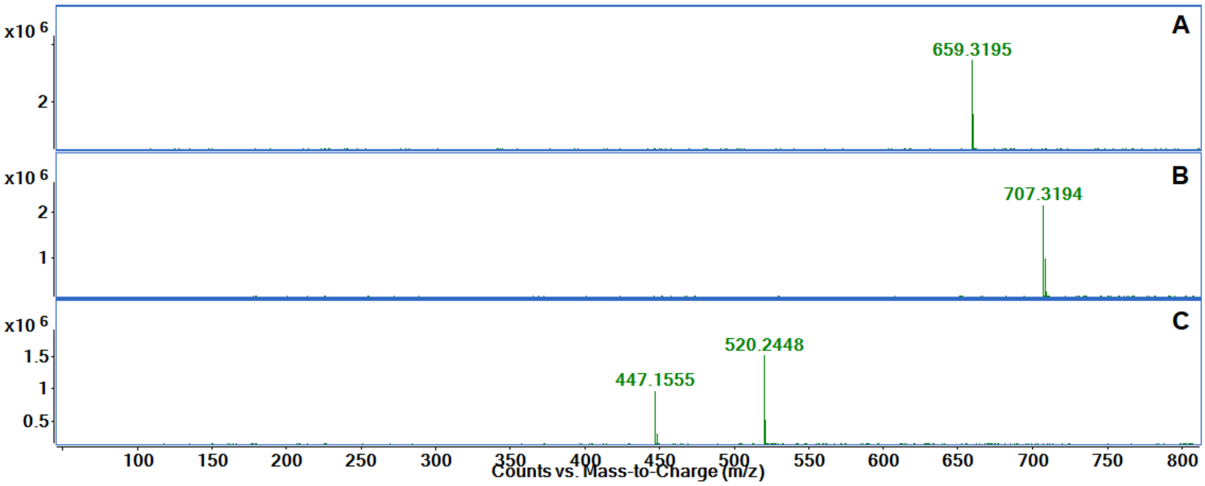

Supplementary Figure 92: Mass spectra of species formed between 10 mM **2b** and 20 mM amino acid mixture (R + F(4-gua)), shown for: A) **2b-R** (retention time 11.49 min), B) **2b- F(4-gua)** (retention time 11.67 min), C) **2b-OH** retention time 14.74 min), in Supplementary Figure 91 A, obtained from the LC-MS analysis.

Calculated m/z $[M+H]^+$: **2b-R** 659.3188, **2b-F(4-gua)** 707.3188, **2b-OH** 503.2177.
Observed m/z $[M+H]^+$: **2b-R** 659.3195, **2b-F(4-gua)** 707.3194, $[M+H_2O]$: **2b-OH** 520.2448.

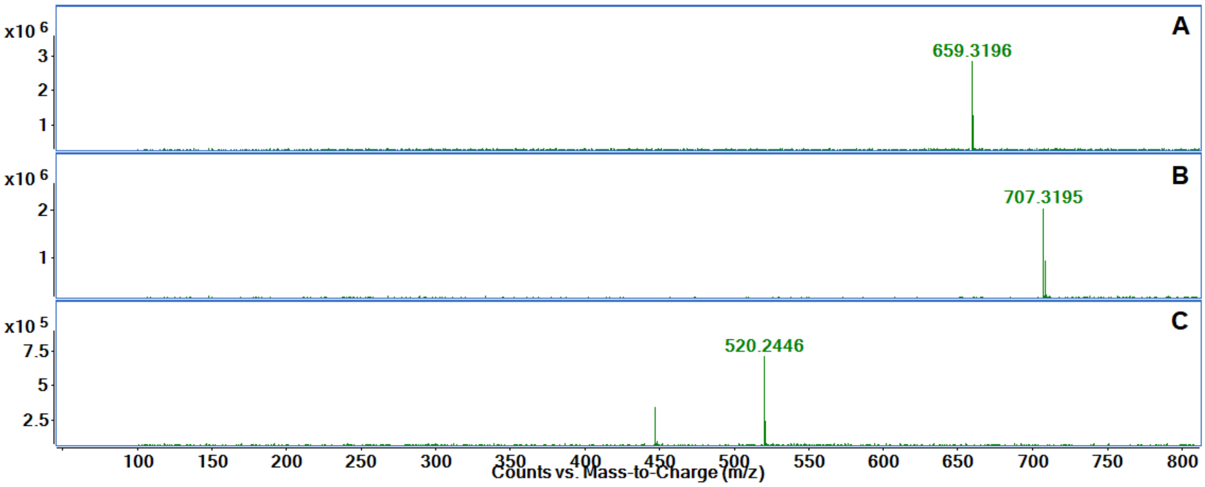

Supplementary Figure 93: Mass spectra of peptides formed between 10 mM **3** and 20 mM amino acid mixture (R + F(4-gua), shown for: A) **3-R** (retention time 11.49 min), B) **3-F(4-gua)** (retention time 11.67 min), C) **3-OH** retention time 14.74 min), in Supplementary Figure 91 B, obtained from the LC-MS analysis.

Calculated m/z $[M+H]^+$: **3-R** 659.3188, **3-F(4-gua)** 707.3188, **3-OH** 503.2177.
Observed m/z $[M+H]^+$: **3-R** 659.3196, **3-F(4-gua)** 707.3195, $[M+H_2O]$: **3-OH** 520.2446.

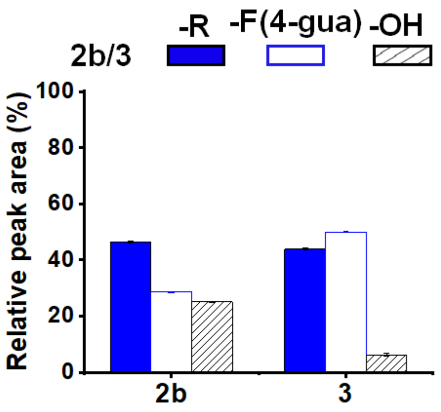

Supplementary Figure 94: Bar graph showing peptide coupling between 10 mM **2b** or 10 mM **3** with 20 mM amino acid mixture (R, F(4-gua)), each amino acid at 10 mM) in 0.6 M borate buffer, pH 9.1. Peptide coupling yields were measured after 48 hours.

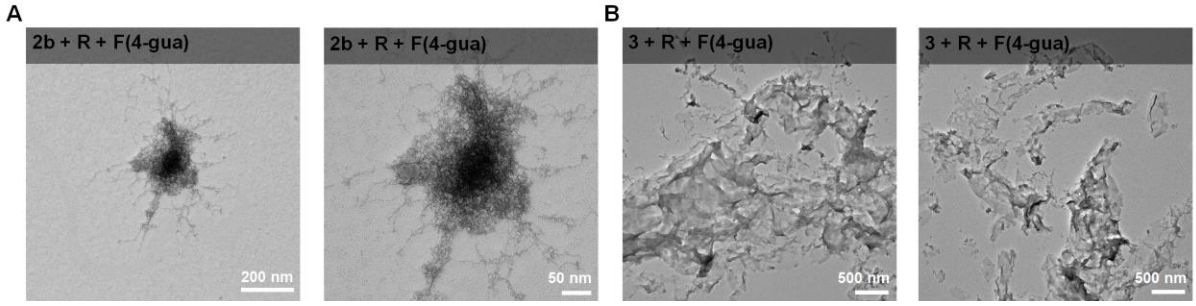

Supplementary Figure 95: TEM images of A) 10 mM **2b** and B) 10 mM **3** with 20 mM amino acid mixture (R, F(4-gua), each amino acid is 10 mM) in 0.6 M borate buffer, pH 9.1.

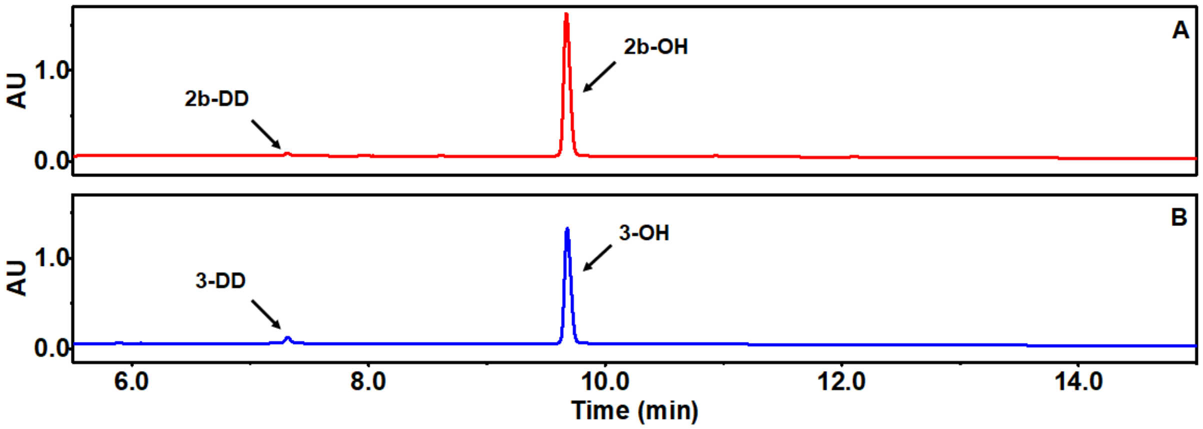

Supplementary Figure 96: UPLC chromatograms of A) 10 mM **2b** and B) 10 mM **3** with 10 mM DD in 0.6 M borate buffer, pH 9.1. Measurements were taken after 72 hours.

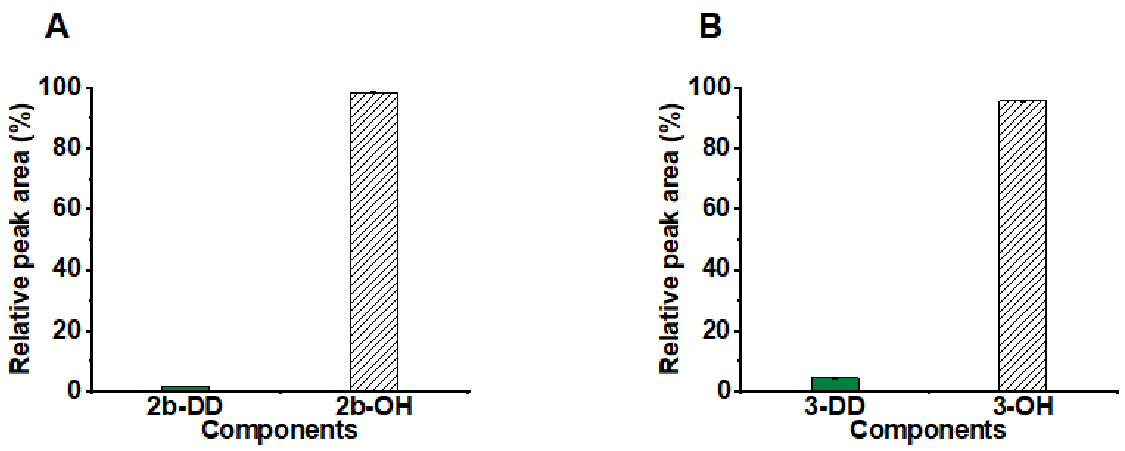

Supplementary Figure 97: Bar graphs showing peptide conversion of A) 10 mM **2b** and B) 10 mM **3** with 10 mM DD in 0.6 M borate buffer, pH 9.1. In each bar graph striped bar represent the hydrolysis product **2b-OH/3-OH**. Error bars represent standard deviation from three independent experiments. Peptide coupling yields were measured after 72 hours.

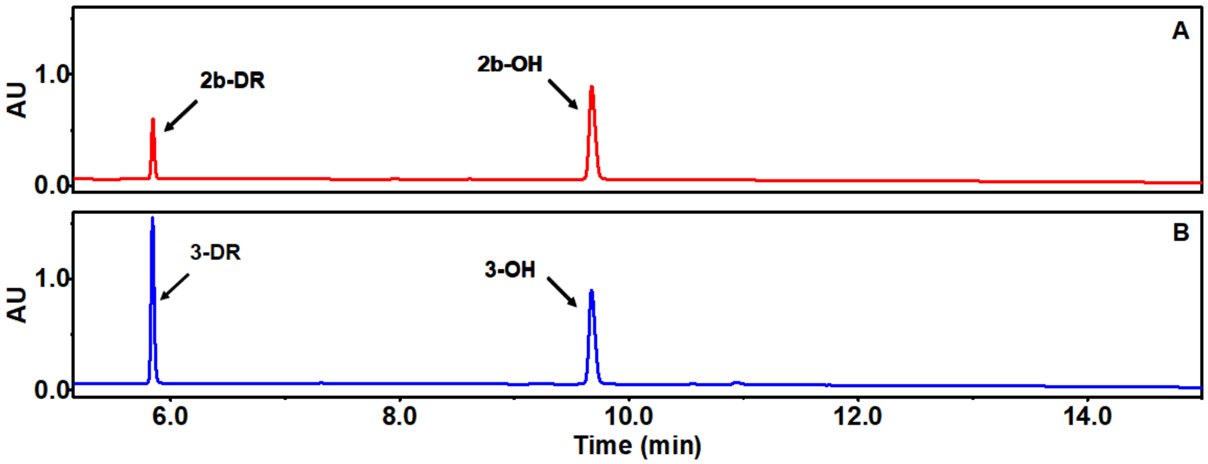

Supplementary Figure 98: UPLC chromatograms of A) 10 mM **2b** and B) 10 mM **3** with 10 mM DR in 0.6 M borate buffer, pH 9.1. Measurements were taken after 72 hours.

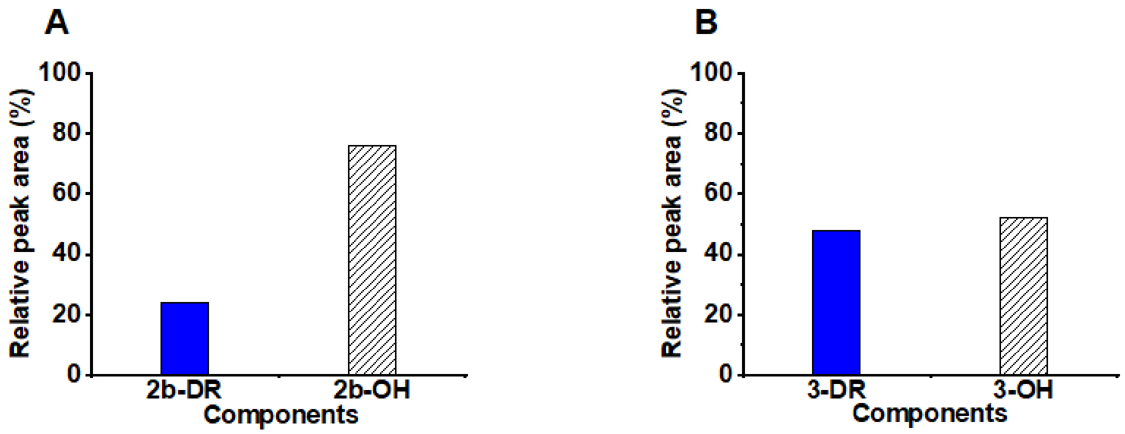

Supplementary Figure 99: Bar graphs showing peptide coupling of A) 10 mM **2b** and B) 10 mM **3** with 10 mM DR, in 0.6 M borate buffer, pH 9.1. In each bar graph striped bar represent the hydrolysis product **2b-OH/3-OH**. Peptide coupling yields were measured after 72 hours.

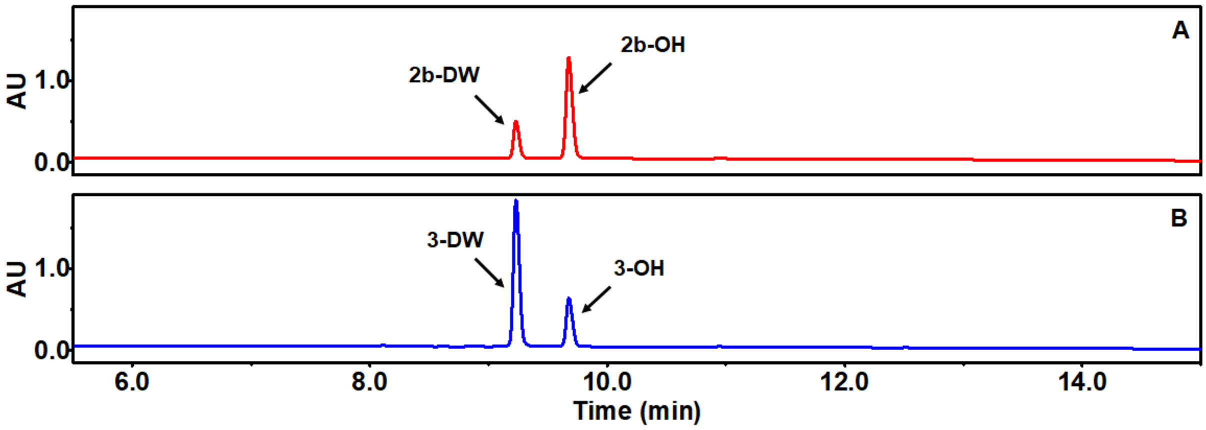

Supplementary Figure 100: UPLC chromatograms of A) 10 mM **2b** and B) 10 mM **3** with 10 mM DW in 0.6 M borate buffer, pH 9.1. Measurements were taken after 72 hours.

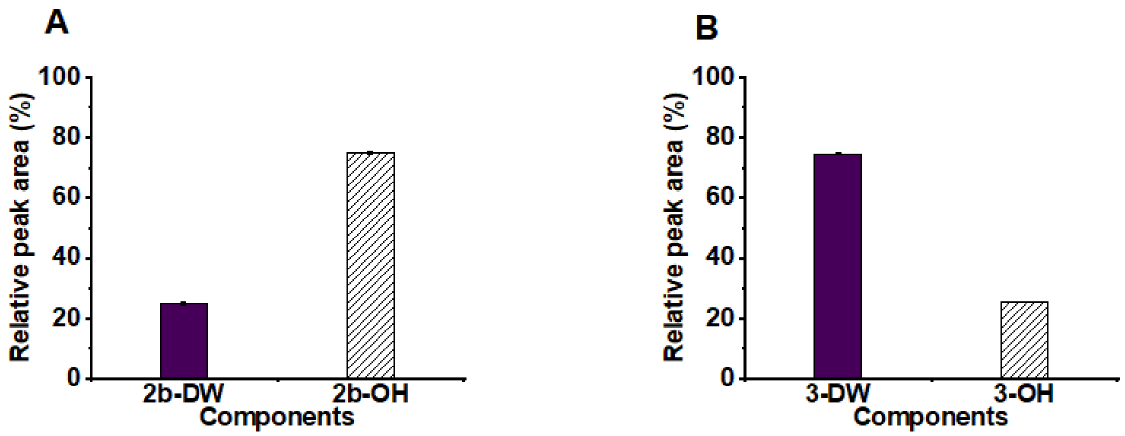

Supplementary Figure 101: Bar graphs showing peptide coupling of A) 10 mM **2b** and B) 10 mM **3** with 10 mM DW, in 0.6 M borate buffer, pH 9.1. In each bar graph striped bar represent the hydrolysis product **2b-OH/3-OH**. Error bars represent standard deviation from three independent experiments. Peptide coupling yields were measured after 72 hours.

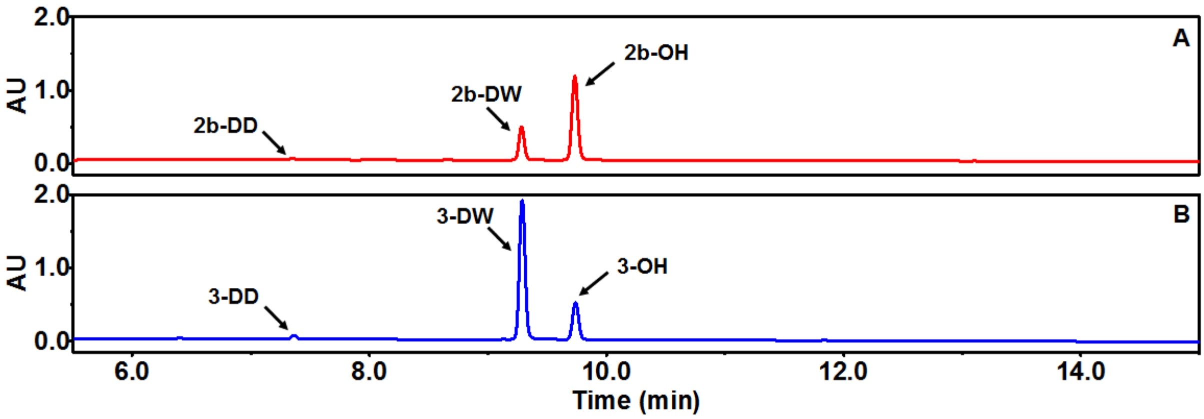

Supplementary Figure 102: UPLC chromatograms of A) 10 mM **2b** and B) 10 mM **3** with 20 mM DD + DW (each dipeptide is 10 mM), in 0.6 M borate buffer, pH 9.1. Measurements were taken after 72 hours.

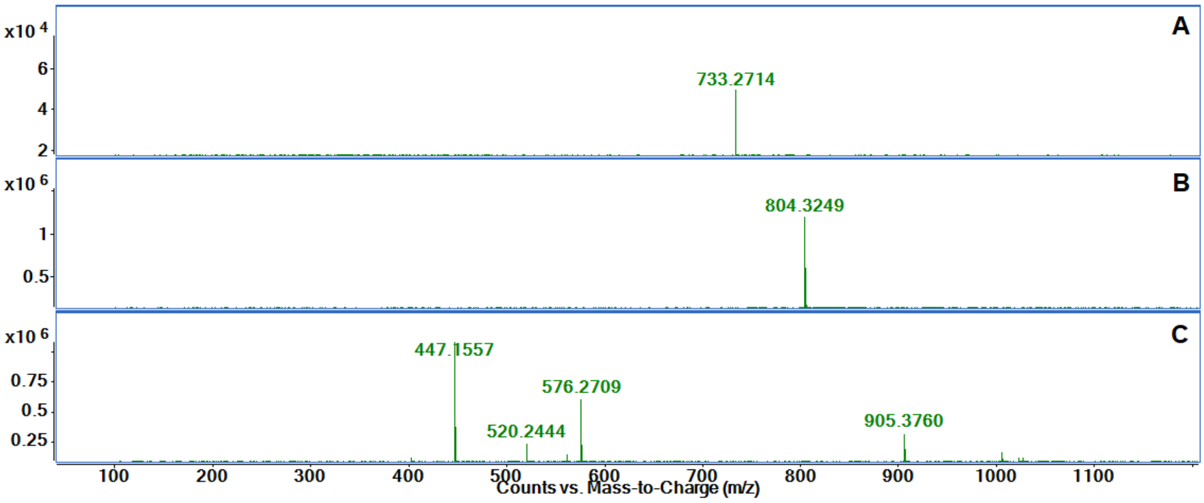

Supplementary Figure 103: Mass spectra of species formed between 10 mM **2b/3** and 20 mM DD + DW (each dipeptide is 10 mM), shown for: A) **2b/3-DD** (retention time 7.35 min), B) **2b/3-DW** (retention time 9.28 min), C) **2b/3-OH** (retention time 9.73 min), in Supplementary Figure 102 A and B, obtained from the LC-MS analysis.

Calculated m/z $[M+H]^+$: **2b/3-DD** 733.2715, **2b/3-DW** 804.3239, **2b/3-OH** 503.2177.
Observed m/z $[M+H]^+$: **2b/3-DD** 733.2714, **2b/3-DW** 804.3249, $[M+H_2O]$: **2b/3-OH** 520.2444.

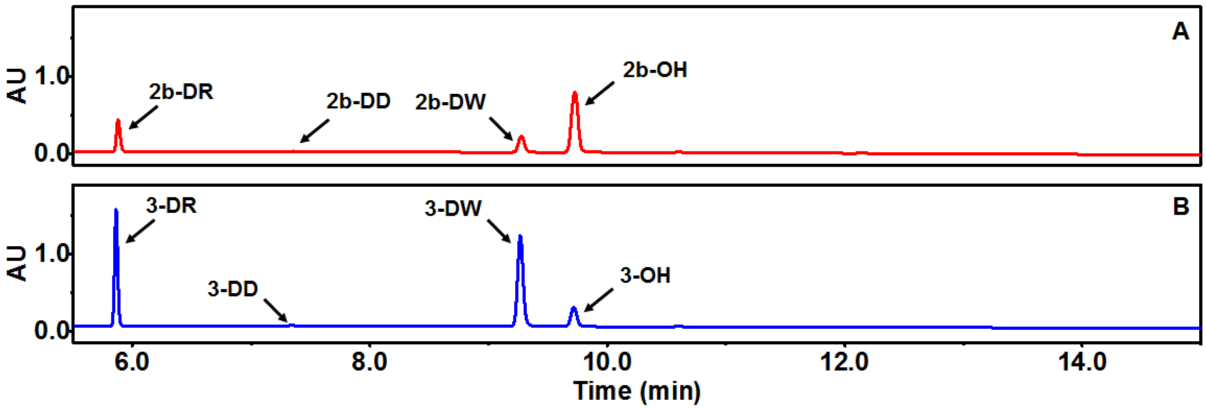

Supplementary Figure 104: UPLC chromatograms of A) 10 mM **2b** and A) 10 mM **3** with 30 mM dipeptide mixture (DR, DD, DW, each dipeptide is 10 mM), in 0.6 M borate buffer, pH 9.1. Measurements were taken after 72 hours.

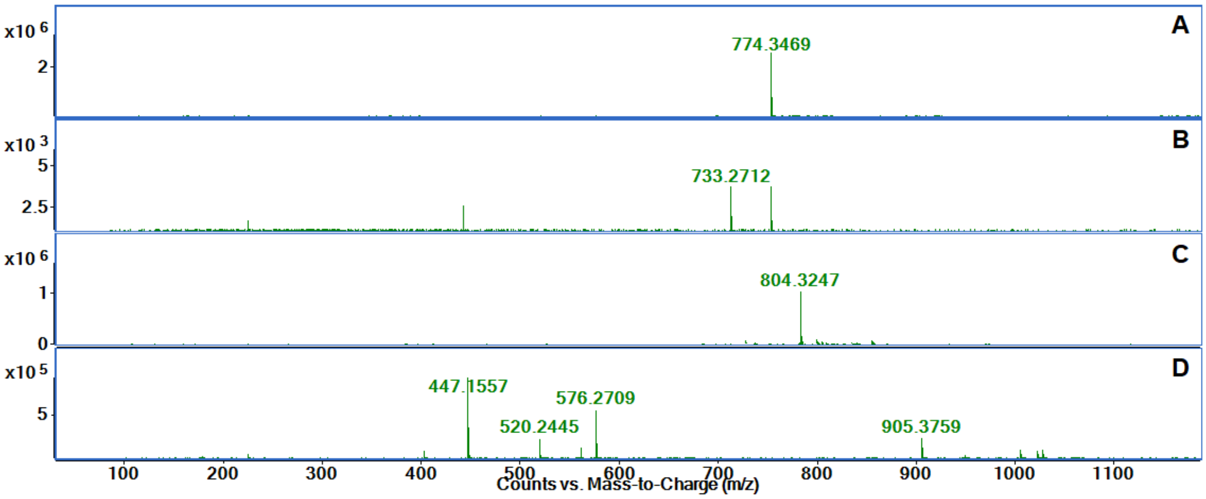

Supplementary Figure 105: Mass spectra of species formed between 10 mM **2b/3** and 30 mM dipeptide mixture (DR, DD, DW, each dipeptide is 10 mM), shown for: A) **2b/3-DR** (retention time 5.85 min), B) **2b/3-DD** (retention time 7.35 min), C) **2b/3-DW** (retention time 9.26 min), D) **2b/3-OH** (retention time 9.71 min), in Supplementary Figure 104 A and B, obtained from the LC-MS analysis.

Calculated m/z $[M+H]^+$: **2b/3-DR** 774.3457, **2b/3-DD** 733.2715, **2b/3-DW** 804.3239, **2b/3-OH** 503.2177.

Observed m/z $[M+H]^+$: **2b/3-DR** 774.3469, **2b/3-DD** 733.2712, **2b/3-DW** 804.3247, $[M+H_2O]$: **2b/3-OH** 520.2445.

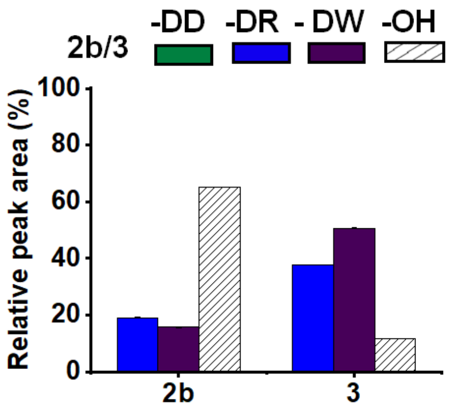

Supplementary Figure 106: Bar graph showing peptide coupling between 10 mM **2b** and 10 mM **3** with 30 mM dipeptide mixture (DR, DD and DW, each dipeptide is 10 mM), in 0.6 M borate buffer, pH 9.1. In each bar graph striped bar represent the hydrolysis product **2b-OH/3-OH**. Error bars represent standard deviation from three independent experiments. Peptide coupling yields were measured after 72 hours.

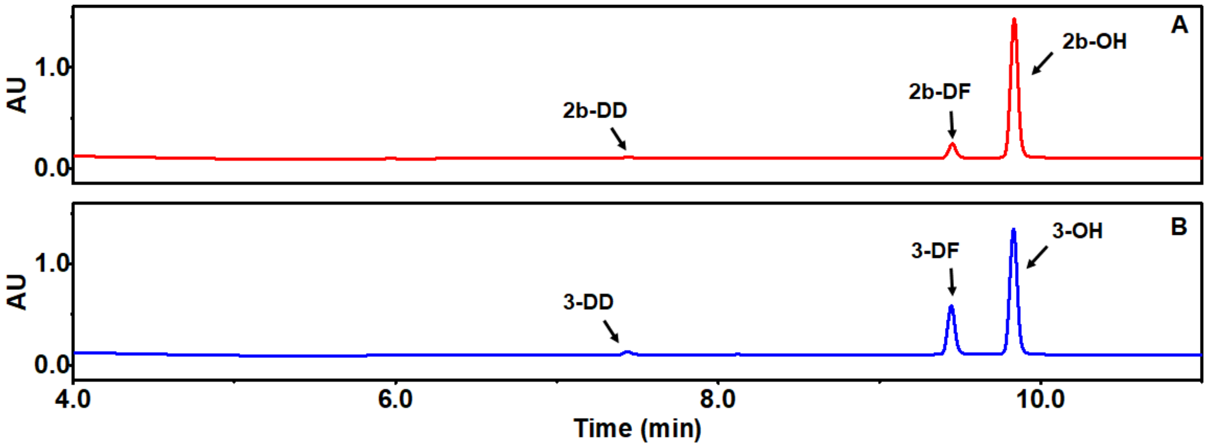

Supplementary Figure 107: UPLC chromatograms of A) 10 mM **2b** and B) 10 mM **3** with 20 mM dipeptide mixture (DD, DF, each dipeptide is 10 mM), in 0.6 M borate buffer, pH 9.1. Measurements were taken after 72 hours.

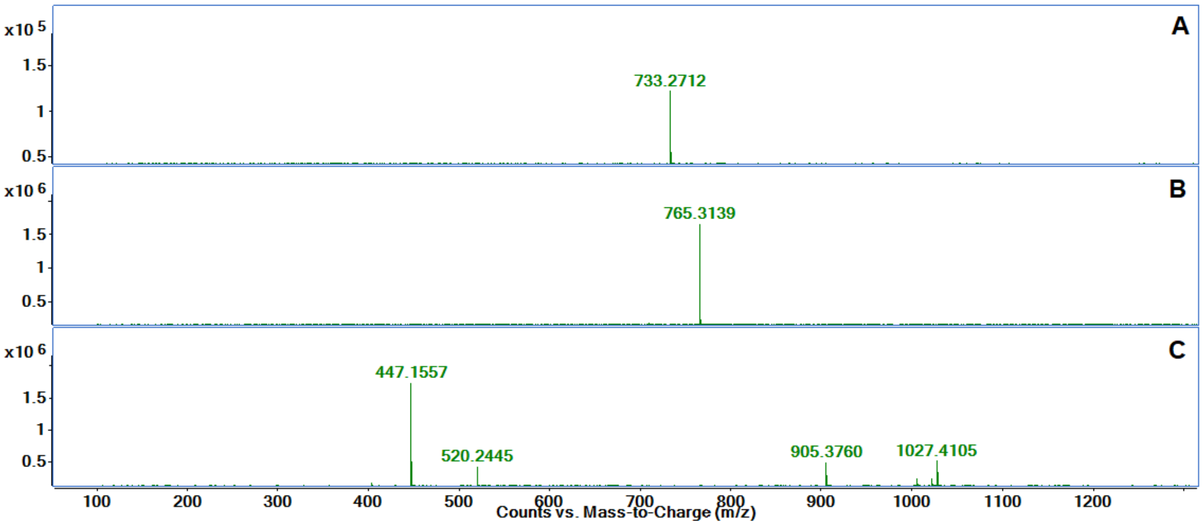

Supplementary Figure 108: Mass spectra of species formed between 10 mM **2b/3** and 20 mM dipeptide mixture (DD, DF, each dipeptide is 10 mM), shown for: A) **2b/3-DD** (retention time 7.35 min), B) **2b/3-DF** (retention time 9.28 min), C) **2b/3-OH** (retention time 9.73 min), in Supplementary Figure 107A and B, obtained from the LC-MS analysis.

Calculated m/z $[M+H]^+$: **2b/3-DD** 733.2715, **2b/3-DF** 765.3130, **2b/3-OH** 503.2177.
Observed m/z $[M+H]^+$: **2b/3-DD** 733.2714, **2b/3-DF** 765.3139, $[M+H_2O]$: **2b/3-OH** 520.2445.

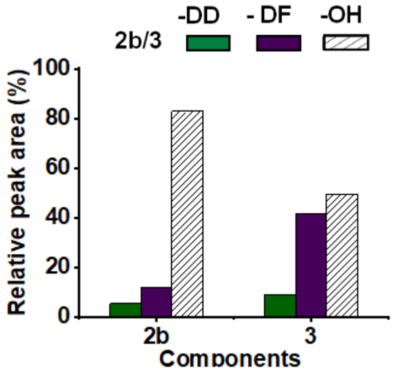

Supplementary Figure 109: Bar graphs showing peptide coupling between 10 mM **2b** or 10 mM **3** with 20 mM dipeptides mixture of DD and DF (10 mM each) in 0.6 M borate buffer, pH 9.1. Peptide coupling yields were measured after 72 hours.

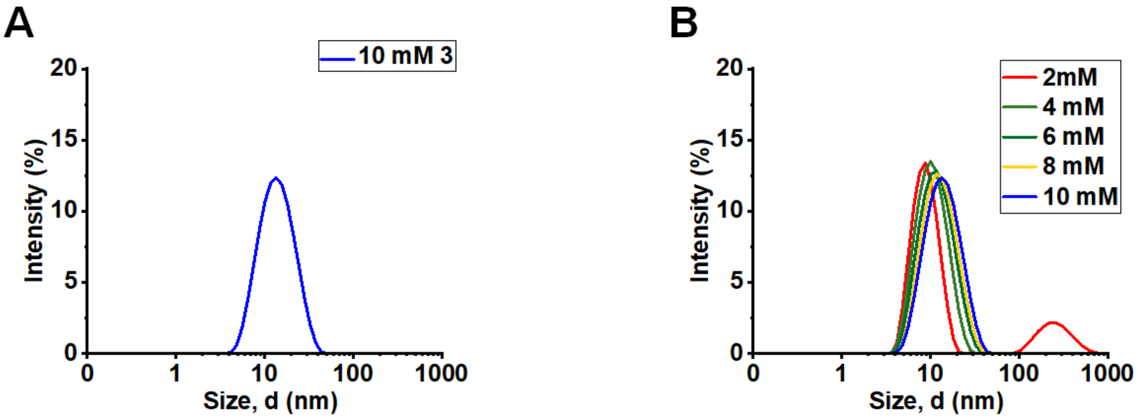

Supplementary Figure 110: A) Size distribution measured by Dynamic Light Scattering (DLS) of 10 mM **3,** B) Concentration-dependent size distribution of **3**, in 0.6 M borate buffer, pH 9.1. Samples of all concentrations were measured upon dissolving **3** in buffer (after 1 min). At lower concentrations *i.e.* at 2 mM, the hydrolysis of **3** into **3-OH** (free acid) resulted in the formation of larger in size aggregates.

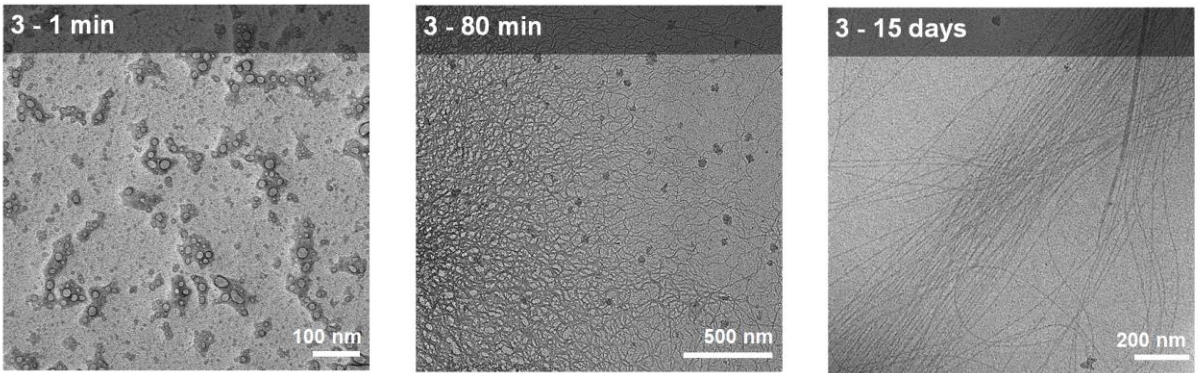

Supplementary Figure 111: Time-dependent cryo-TEM images of 10 mM **3** in 0.6 M borate buffer pH 9.1.

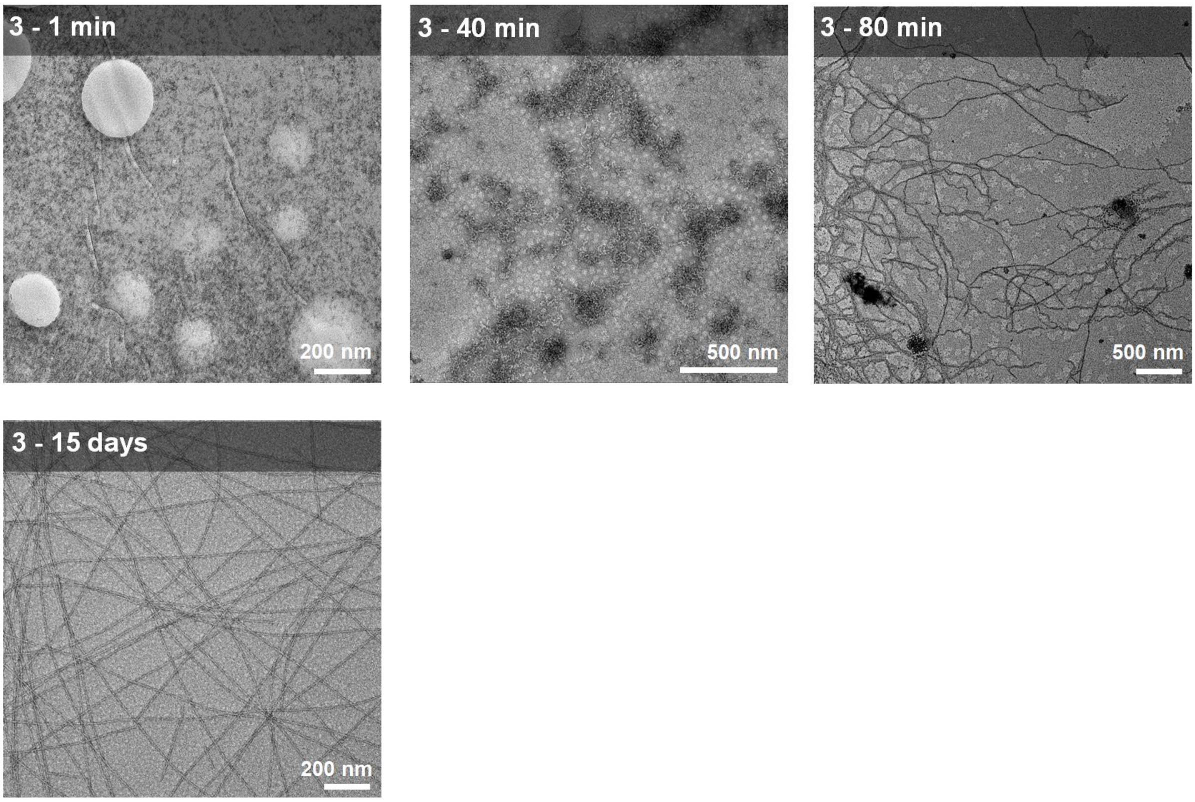

Supplementary Figure 112: Time-dependent TEM images of 10 mM **3** in 0.6 M borate buffer pH 9.1.

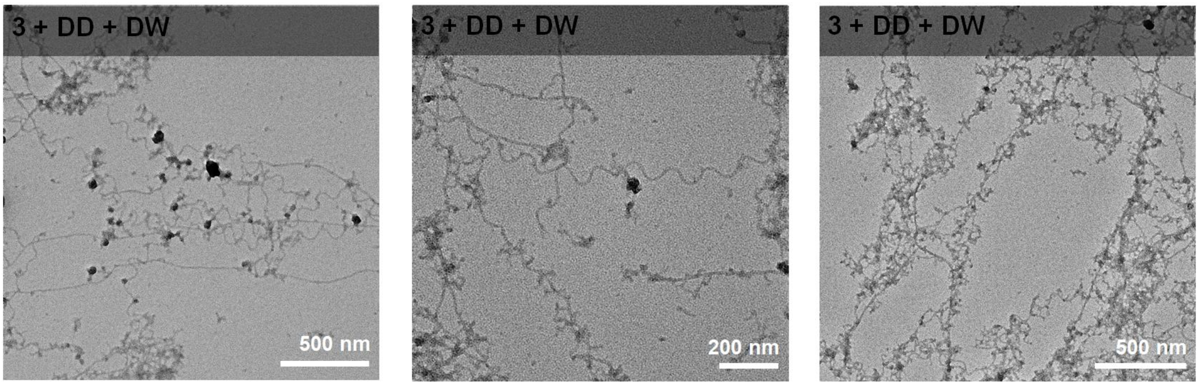

Supplementary Figure 113: TEM images of reaction between 10 mM **3** and mixture of 20 mM DD, DW (each dipeptide is 10 mM), in 0.6 M borate buffer, pH 9.1.

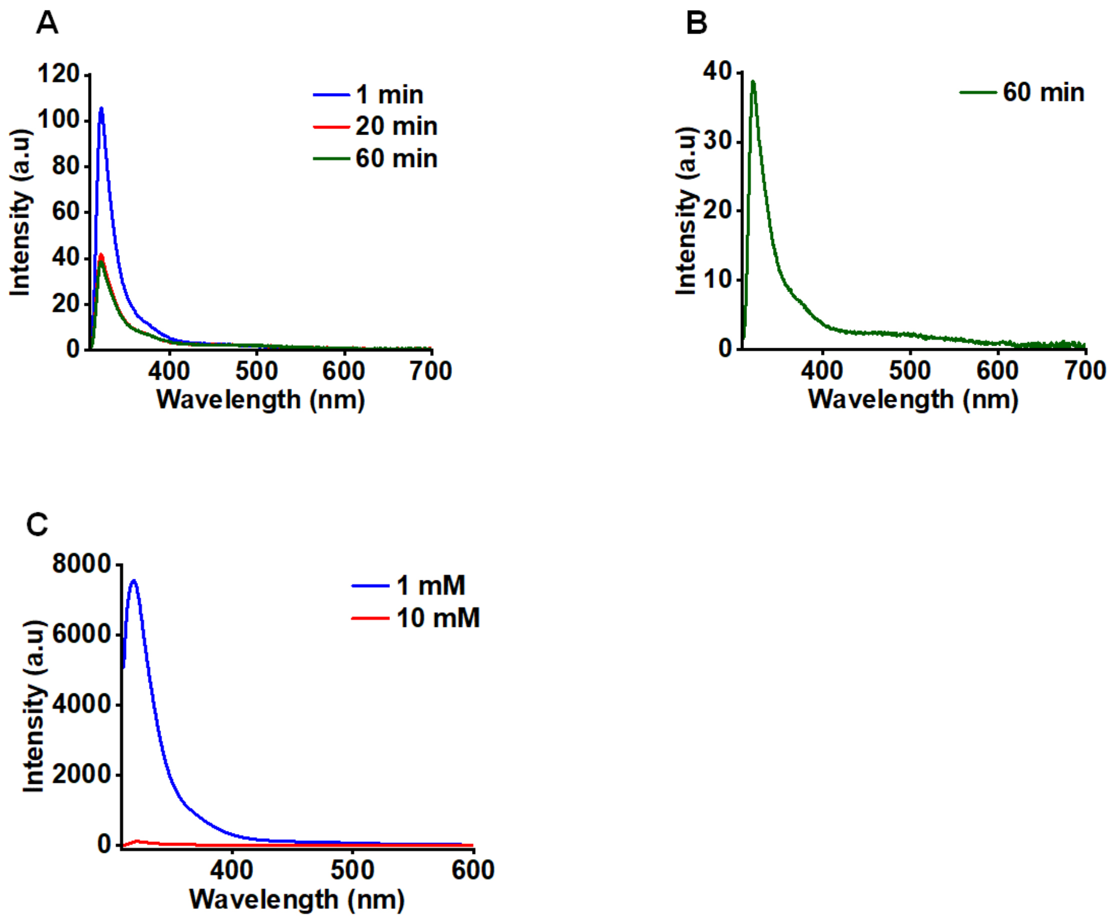

Supplementary Figure 114: Fluorescence emission spectra of A) 10 mM **3**. B) Zoomed in spectra of 10 mM **3** at 60 min. C) Concentration-dependent fluorescence emission spectra of **3**, in 0.6 M borate buffer, pH 9.1.

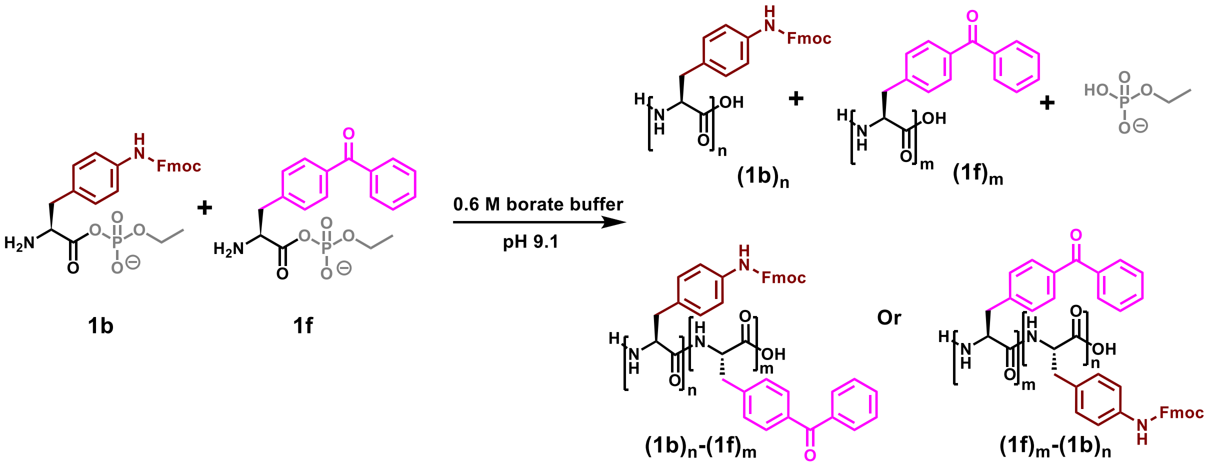

Supplementary Figure 115: Reaction scheme of 10 mM **1b** and 10 mM **1f**.

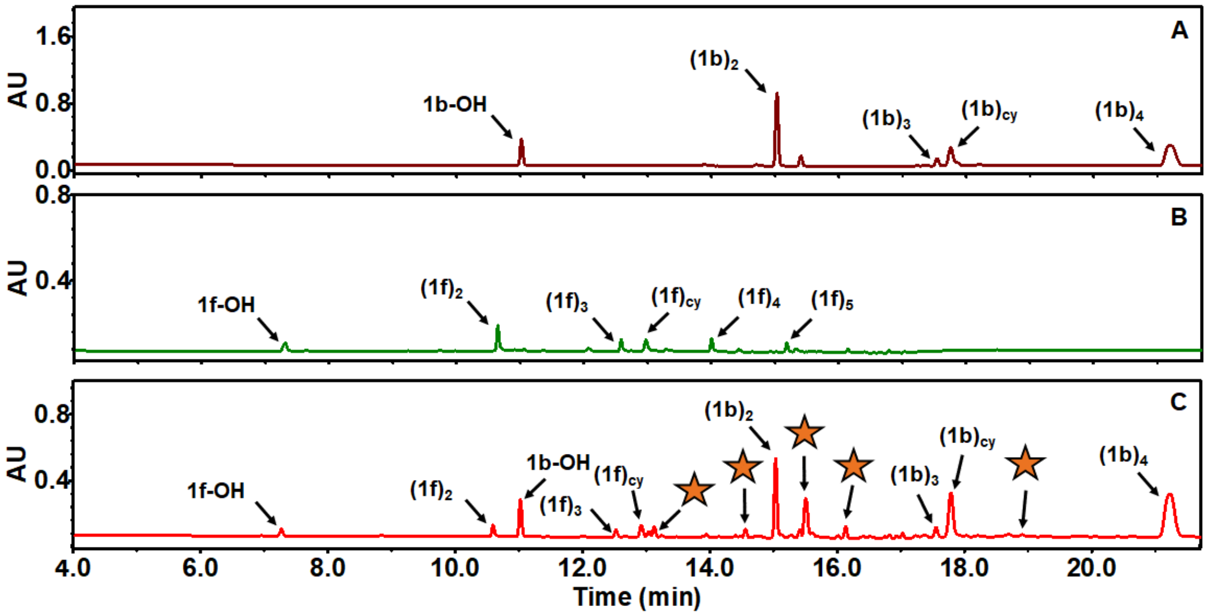

Supplementary Figure 116: UPLC chromatograms of A) 10 mM **1b** B) 10 mM **1f** and C) 10 mM **1b** + 10 mM **1f** (1:1), in 0.6 M borate buffer, pH 9.1. Measurements were taken after 24 hours.

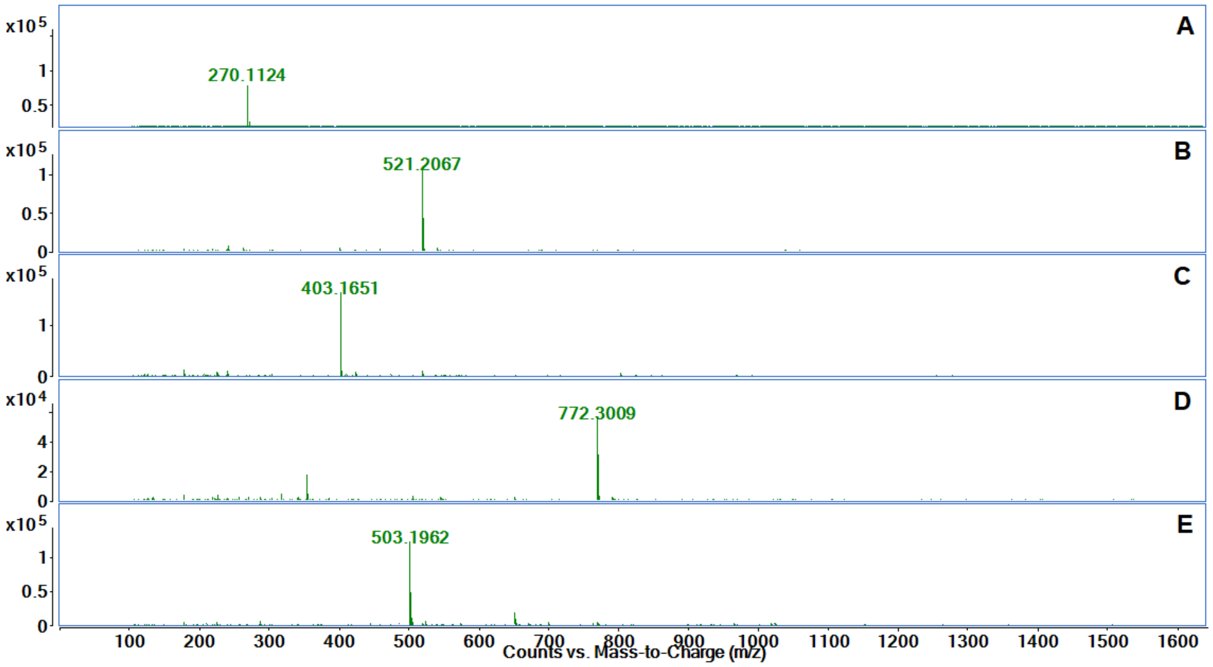

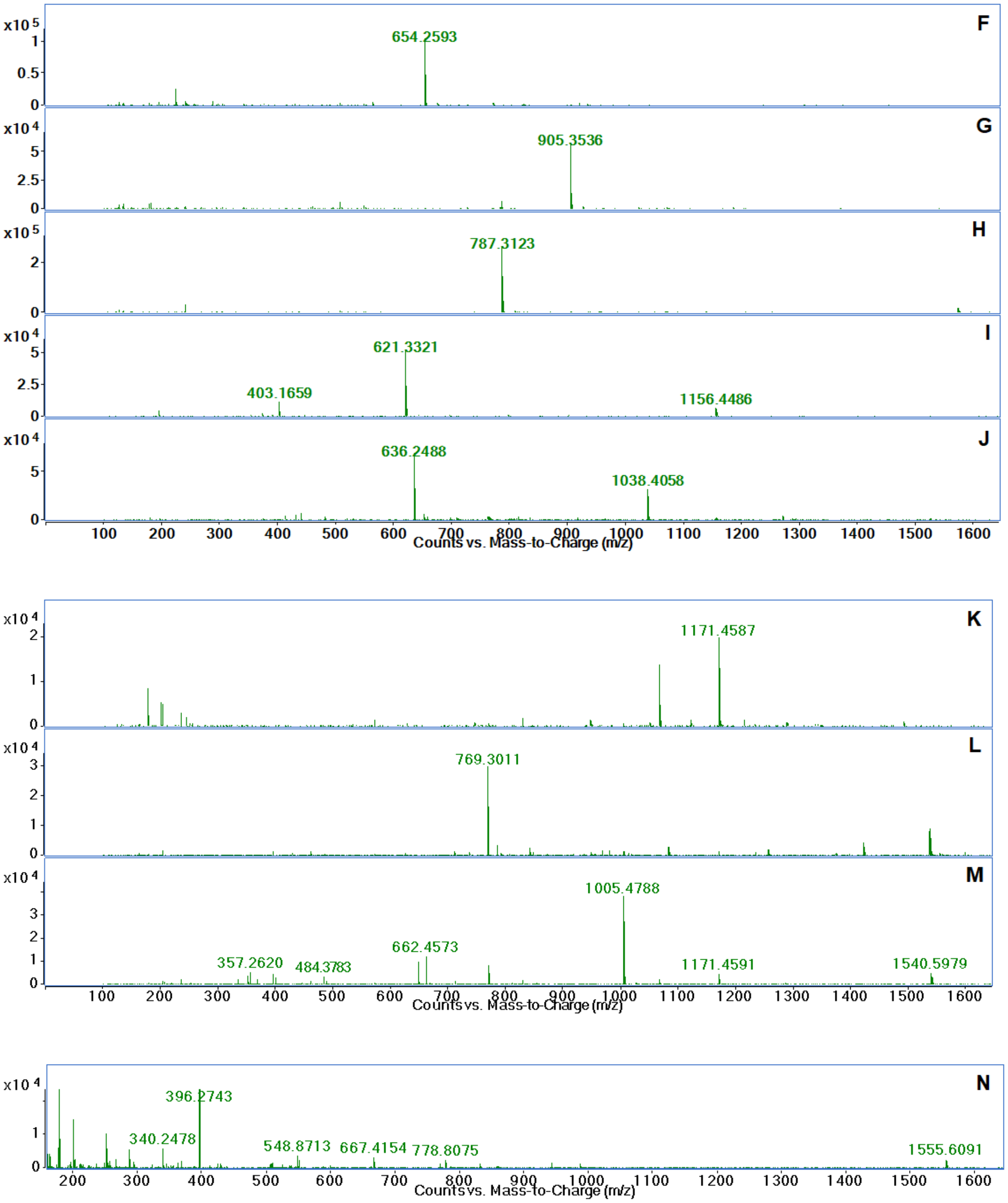

Supplementary Figure 117: Mass spectra of homo and hetero oligomers formed between 10 mM **1b** and 10 mM **1f** (1:1) from figure A-O found in Supplementary Figure 116C, obtained from the LC-MS analysis.

Supplementary Table 2: Mass of oligomers formed in a library of 10 mM **1b** and 10 mM **1f** found in Supplementary Figure 90 C.

Mass spectra Fig. No	Peptides formed	Distribution (%)	Reten-tion time (min)	Calculated m/z $[M+H]^+$	Observed m/z $[M+H]^+$
117 A	$(1f)_1$	4.39	7.26	270.1125	270.1124
117 B	$(1f)_2$	11.41	10.58	521.2071	521.2067
117 C	$(1b)_1$	4.73	11.01	403.1652	403.1651
117 D	$(1f)_3$	3.71	12.51	772.3017	772.3009
117 E	$(1f)_{cy}$#	5.60	12.91	503.1965	503.1962
117 F	Hetero dimer* ★	2.77	13.11	654.2599	654.2593
117 G	Hetero trimer-1* ★	2.26	14.551	905.3545	905.3536
117 H	$(1b)_2$	11.23	15.025	787.3126	787.3123
117 I	Hetero tetramer* ★	17.99	15.50	1156.4491	1156.4486
117 J	Hetero trimer-2*	3.20	16.12	1038.4073	1038.4058
117 K	$(1b)_3$	3.76	17.54	1171.4600	1171.4587
117 L	$(1b)_{cy}$	9.57	17.776	769.3021	769.3011
117 M	Hetero pentamer* ★	0.97	18.891	1540.5965	1540.5979
117 N	$(1b)_4$	18.35	21.217	1555.6074	1555.6091

Cyclic dipeptide (diketopiperazine) of **1b**.

*The formation of hetero dimer corresponds to the molecular weight of **1b-1f** which might also be its constitutional isomer, **1f-1b**.

Hetero trimer-1 = **1b-$(1f)_2$**

Hetero trimer-2 = **$(1b)_2$-1f**

Hetero tetramer = **1b-$(1f)_3$**

Hetero pentamer = **$(1b)_2$-$(1f)_3$**

The mass for the hereto-oligomers could also be assigned to their potential constitutional isomers.

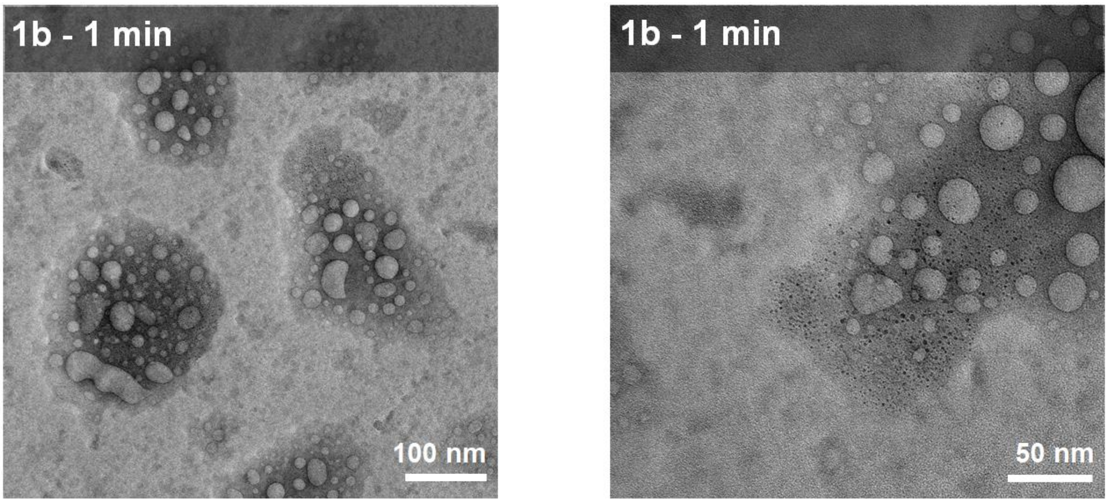

Supplementary Figure 118: Cryo-TEM images of 10 mM **1b** immediately after dissolving in 0.6 M borate buffer, pH 9.1.

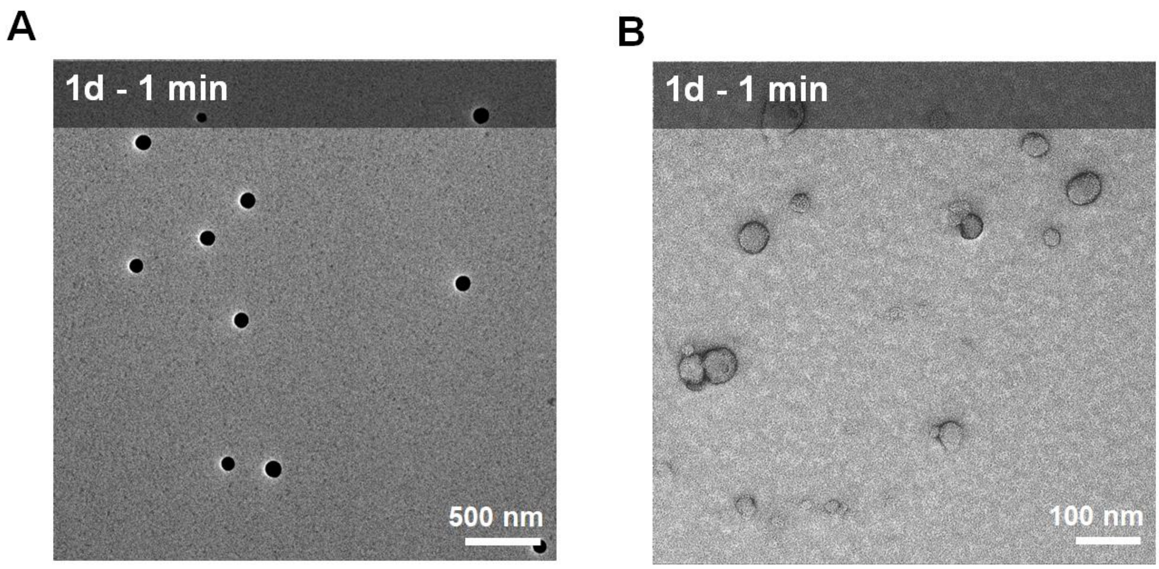

Supplementary Figure 119: (A) Cryo-TEM image and (B) Negative stain TEM of 10 mM **1f** immediately after dissolving in 0.6 M borate buffer, pH 9.1.

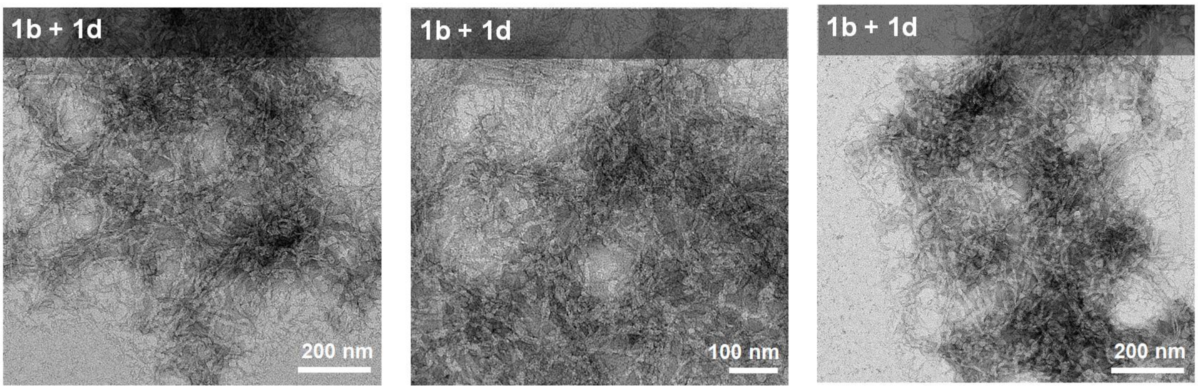

Supplementary Figure 120: TEM images of reaction between 10 mM **1b** and 10 mM **1f** in 0.6 M borate buffer, pH 9.1.

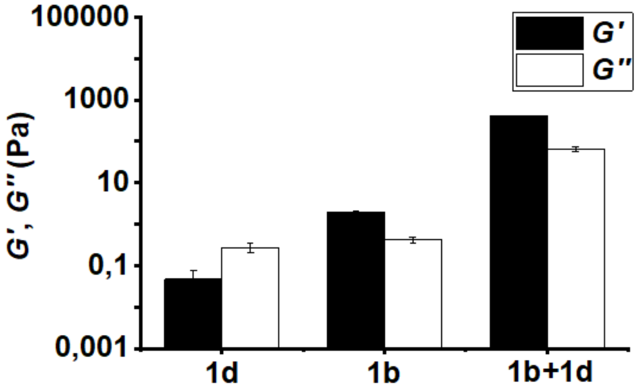

Supplementary Figure 121: Storage and loss modulus of the oligomers formed from 10 mM **1b**, 10 mM **1d** and from the mixture (10 mM each) after oligomerization. The solid squares represent the storage modulus (G’) while open squares represent the loss modulus (G”). In the bar graphs, error bars represent the standard deviation from three independent experiments.

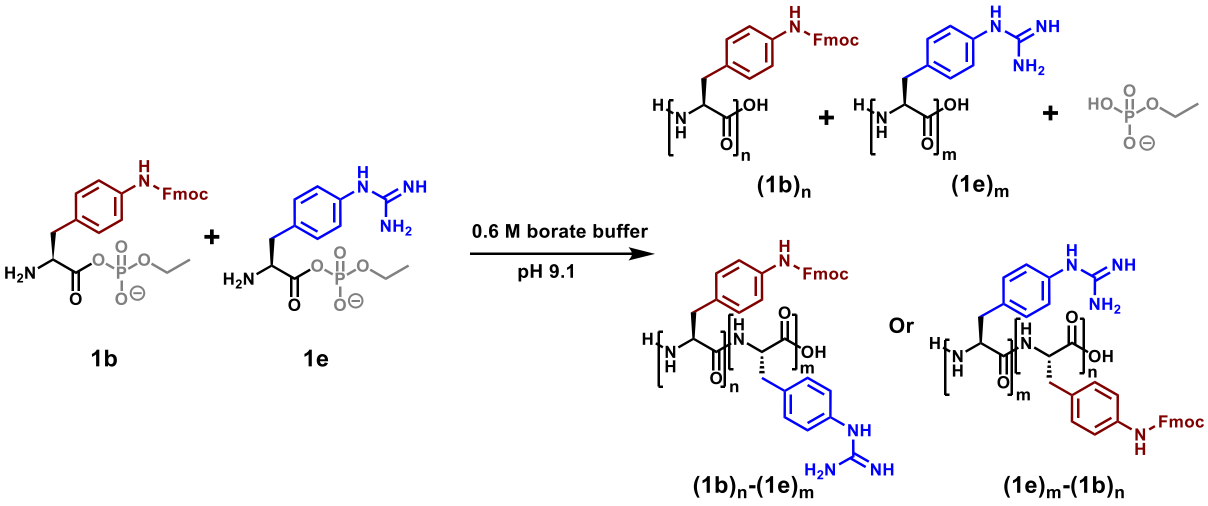

Supplementary Figure 122: Reaction scheme of 10 mM **1b** and 10 mM **1e**.

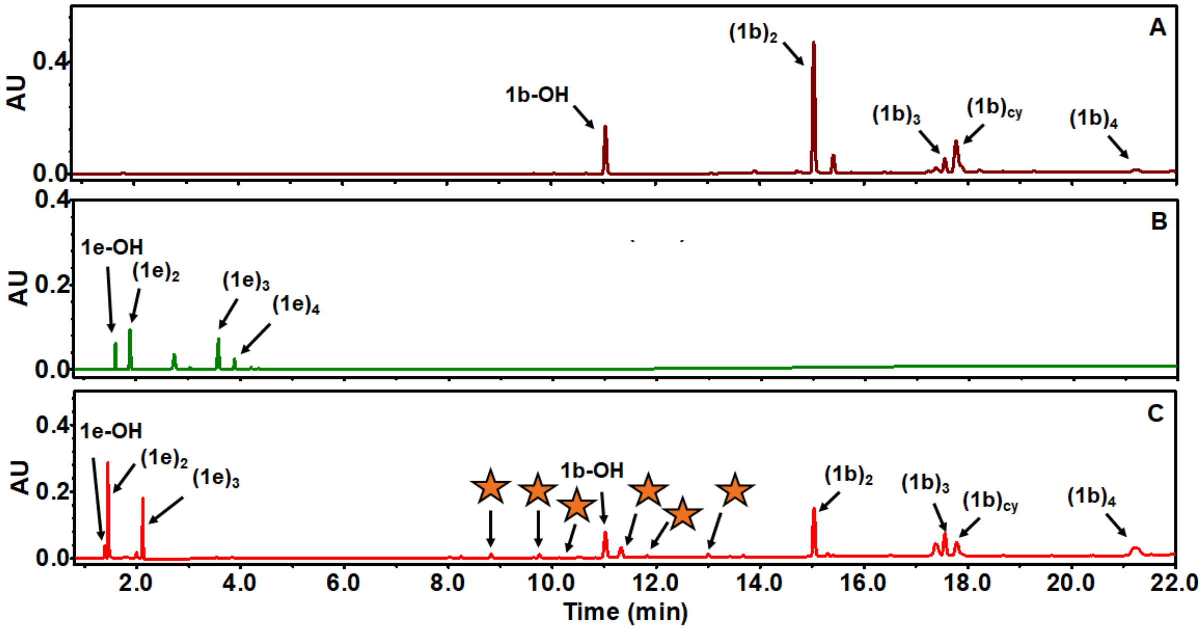

Supplementary Figure 123: UPLC chromatograms of A) 10 mM **1b** B) 10 mM **1e** and C) 10 mM **1b** + 10 mM **1e** (1:1), in 0.6 M borate buffer, pH 9.1. The peak shifts observed (before 4 minutes retention time) in Figure C are attributed to the use of a 30% THF:H_2O solution for sample preparation in UPLC analysis. Measurements were taken after 24 hours.

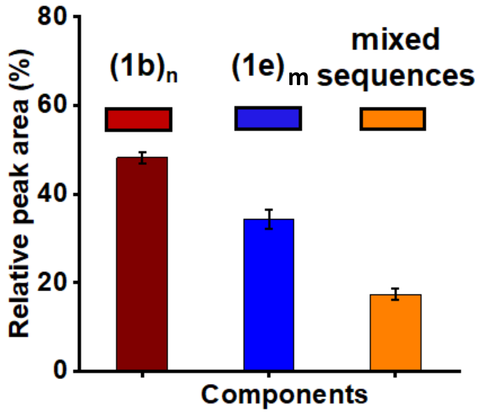

Supplementary Figure 124: Bar graph showing the sum of all library components (grouped as homo or hetero oligomers) formed between 10 mM **1b** and 10 mM **1e** in 0.6 M borate buffer, pH 9.1. Peptide coupling yields were measured after 24 hours. Error bars represent the standard deviation of three independent experiments.

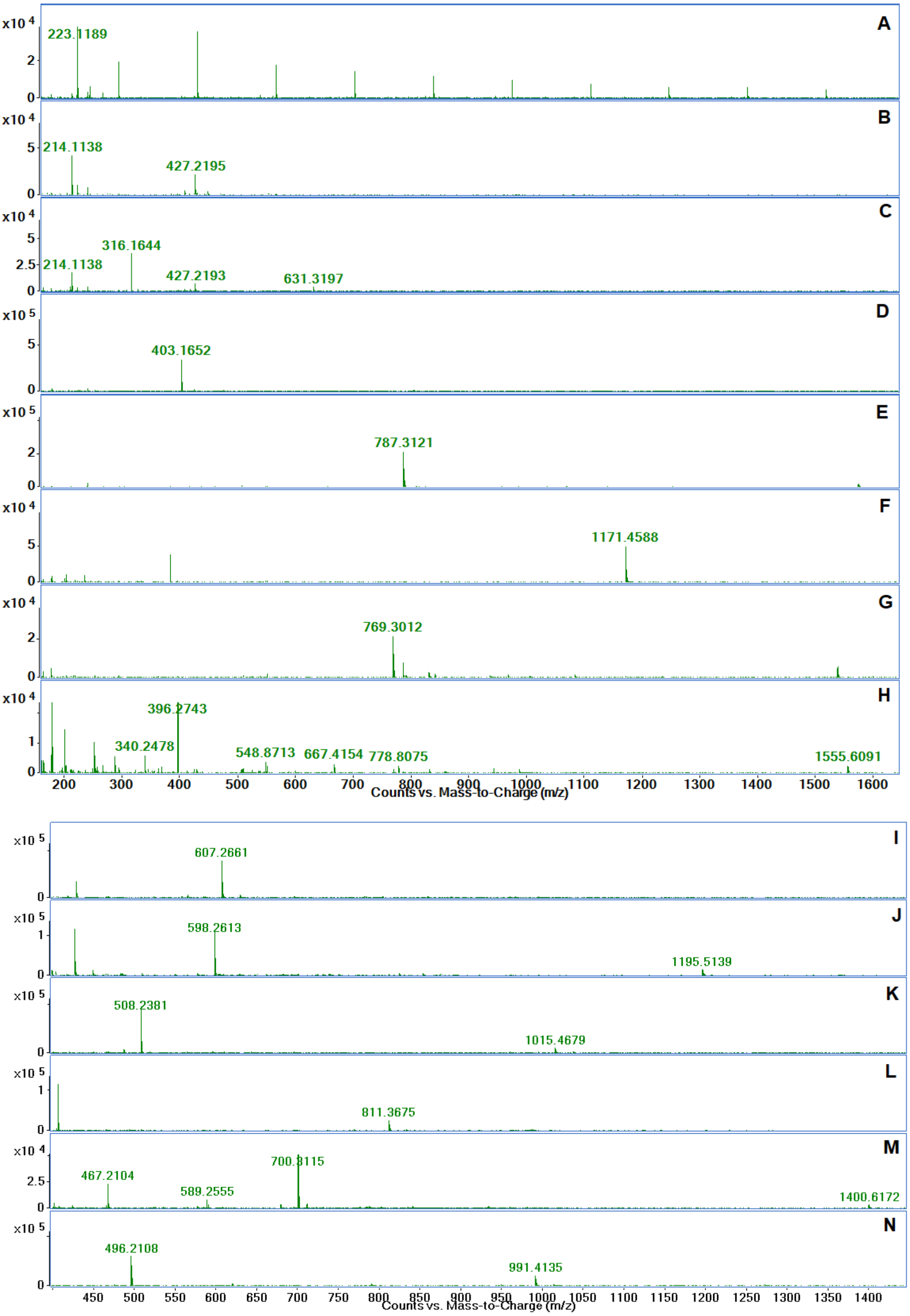

Supplementary Figure 125: Mass spectra of homo and hetero oligomers formed between 10 mM **1b** and 10 mM **1e** (1:1) from figure A-O found in Supplementary Figure 123 C, obtained from the LC-MS analysis.

Supplementary Table 3: Mass of oligomers formed in a library of 10 mM **1b** and 10 mM **1e** found in Supplementary Figure 123 C.

Mass spectra Fig. No	Peptides formed	Distribution (%)	Reten-tion time (min)	Calculated m/z $[M+H]^+$	Observed m/z $[M+H]^+$
125 A	(1e)$_1$	3.42	1.38	223.1190	223.1189
125 B	(1e)$_2$	19.39	1.44	427.2201	427.2195
125 C	(1e)$_3$	11.54	2.11	631.3212	631.3197
125 D	(1e)$_1$	8.11	11.02	403.1652	403.1654
125 E	(1b)$_2$	17.91	15.03	787.3126	787.3121
125 F	(1b)$_3$	4.34	17.55	1171.4600	1171.4588
125 G	(1b)$_{cy}$#	7.98	17.78	769.3021	769.3012
125 H	(1b)$_4$	9.87	21.21	1555.6074	1555.6091
125 I	hetero dimer* ★	2.32	9.75	607.2663	607.2661
125 J	hetero tetramer-1* ★	1.26	11.82	1195.5148	1195.5139
125 K	hetero tetramer-2* ★	2.12	8.82	1015.4686	1015.4679
125 L	hetero trimer-1* ★	1.03	9.75	811.3675	811.3675
125 M	hetero pentamer-1* ★	8.60	11.31	1399.6160	1400.6172
125 N	hetero trimer-2* ★	2.05	13.00	991.4137	991.4135

Cyclic dipeptide (diketopiperazine) of **1b**.
*The formation of hetero dimer corresponds to the molecular weight of **1b-1e** which might also be its constitutional isomer, **1e-1b**.

Hetero trimer-1 = **1b-(1e)$_2$**

Hetero trimer-2 = **(1b)$_2$-1e**

Hetero tetramer-1 = **(1b)$_2$-(1e)$_2$**

Hetero tetramer-2 = **1b-(1e)$_3$**

Hetero pentamer = **(1b)$_2$-(1e)$_3$**

The mass for the hereto-oligomers could also be assigned to their potential constitutional isomers.

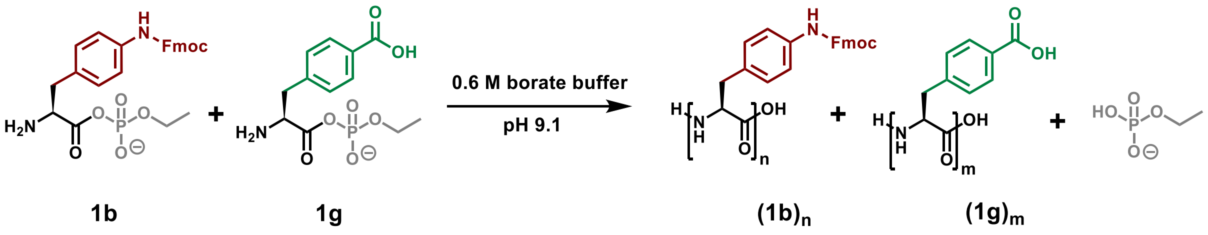

Supplementary Figure 126: Reaction scheme of 10 mM **1b** and 10 mM **1g**.

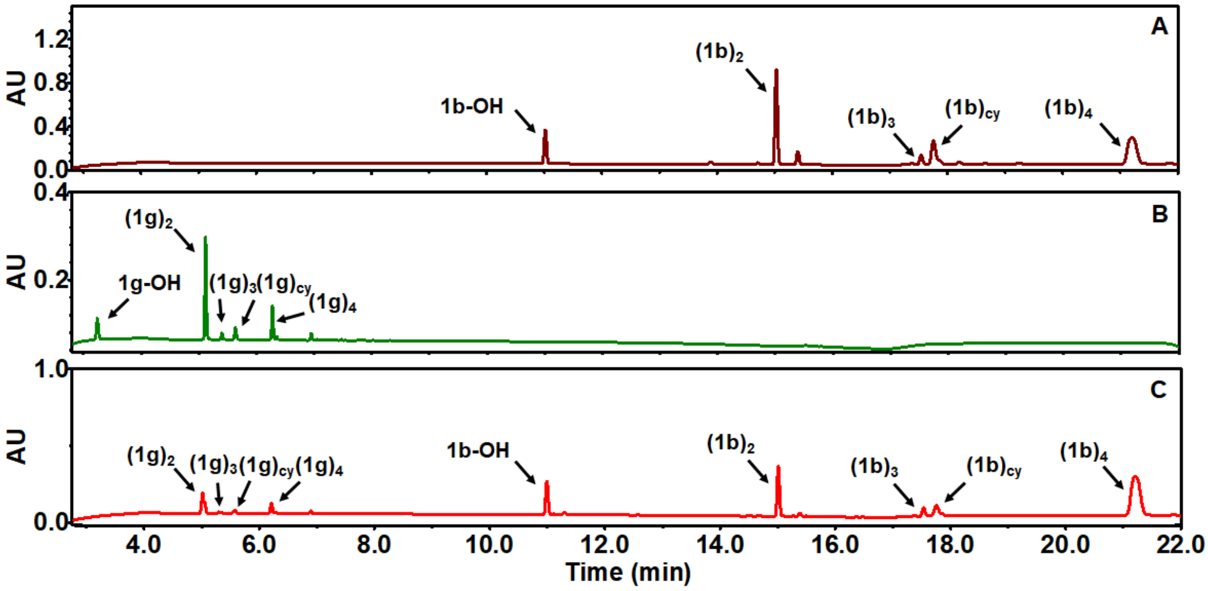

Supplementary Figure 127: UPLC chromatograms of A) 10 mM **1b** B) 10 mM **1g** and C) 10 mM **1b** + 10 mM **1g** (1:1), in 0.6 M borate buffer, pH 9.1. Measurements were taken after 24 hours.

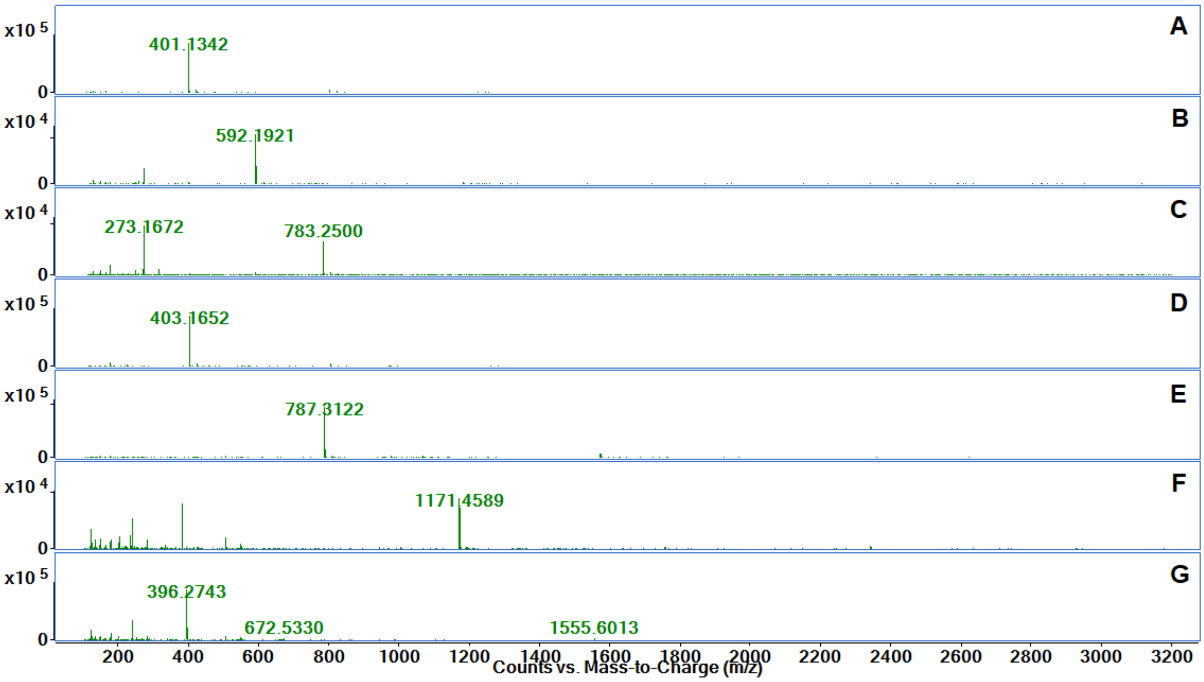

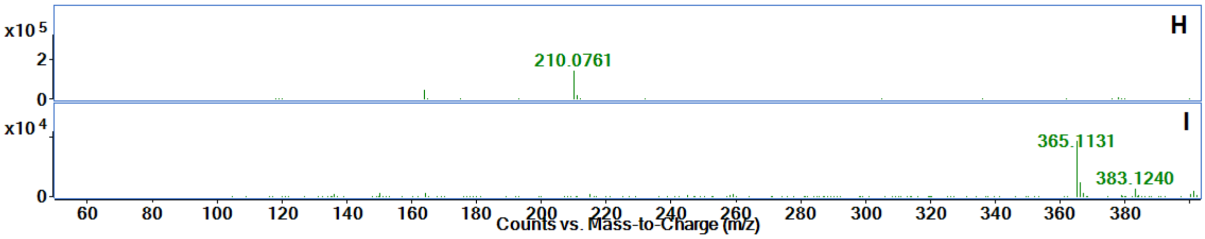

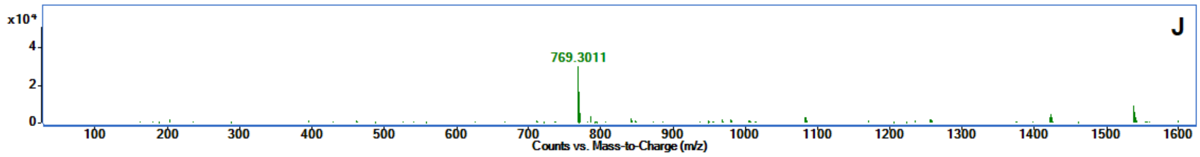

Supplementary Figure 128: Mass spectra of self-sorted homo oligomers formed between 10 mM **1b** and 10 mM **1g** (1:1) from figure A-J found in Supplementary Figure127 C, obtained from the LC-MS analysis.

Supplementary Table 4: Mass of oligomers formed in a library of 10 mM **1b** and 10 mM **1g** found in Supplementary Figure 127 C.

Mass spectra Fig. No	Peptides formed	Distribution (%)	Retention time (min)	Calculated m/z $[M+H]^+$	Observed m/z $[M+H]^+$
128 A	$(1g)_2$	25.19	5.06	401.1343	401.1342
128 B	$(1g)_3$	1.57	5.34	592.1926	592.1921
128 C	$(1g)_4$	11.22	6.25	783.2508	783.2500
128 D	$(1b)_1$	6.89	11.02	403.1652	403.1652
128 E	$(1b)_2$	9.89	15.02	787.3126	787.3122
128 F	$(1b)_3$	2.17	17.54	1171.4600	1171.4589
128 G	$(1b)_4$	26.81	21.21	1555.6074	1555.6013
128 H	$(1g)_1$	6.18	3.31	210.0761	210.0761
128 I	$(1g)_{cy}$[#]	3.73	5.62	383.1238	383.1240
128 J	$(1b)_{cy}$[#]	4.27	17.76	769.3021	769.3011

[#]Cyclic dipeptides (diketopiperazine) of **1g** and **1b**.

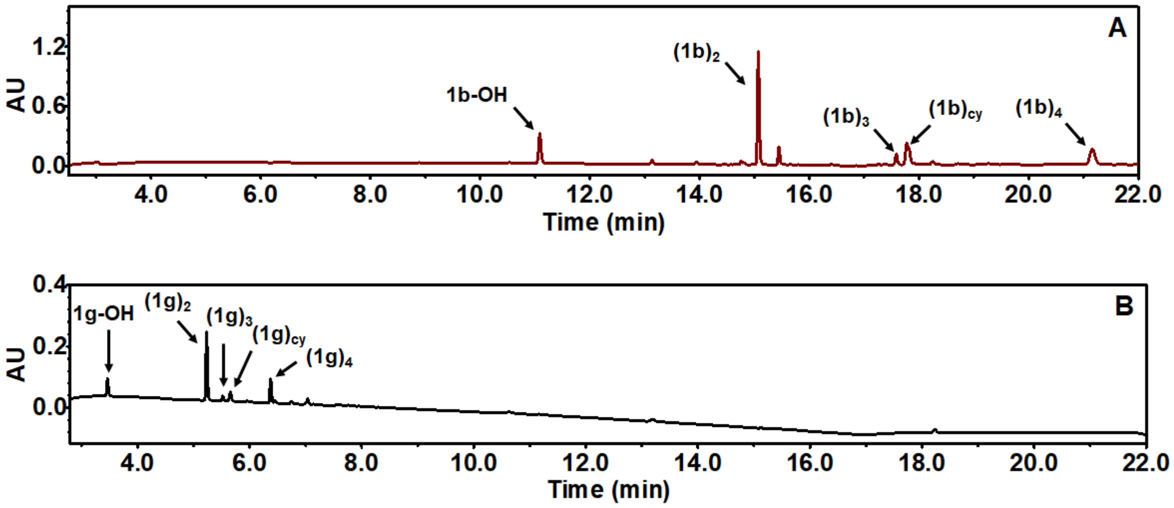

Supplementary Figure 129: UPLC chromatograms after centrifugation of the library between 10 mM **1b** and 10 mM **1g** A) Oligomers of **1b** in aggregated phase B) Oligomers of **1g** in solution phase. Measurements were taken after 24 hours.

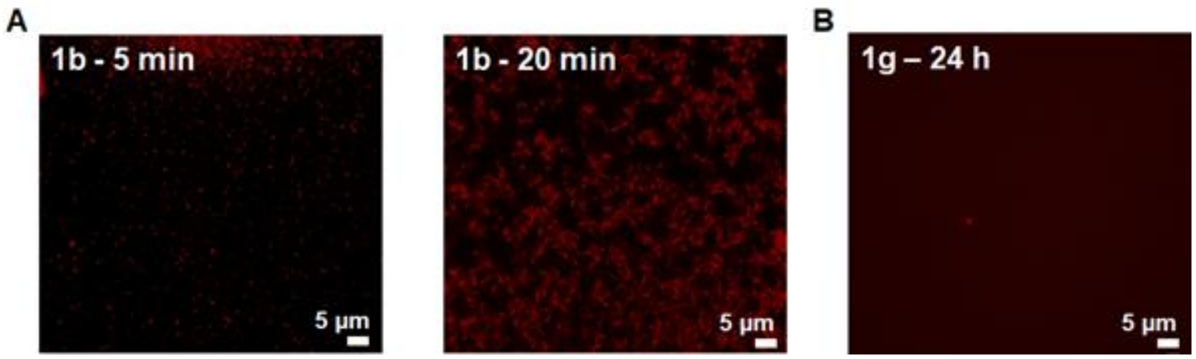

Supplementary Figure 130: Time-dependent confocal microscopy images of A) 10 mM **1b**. B) 10 mM **1g** in 0.6 M borate buffer, pH 9.1.

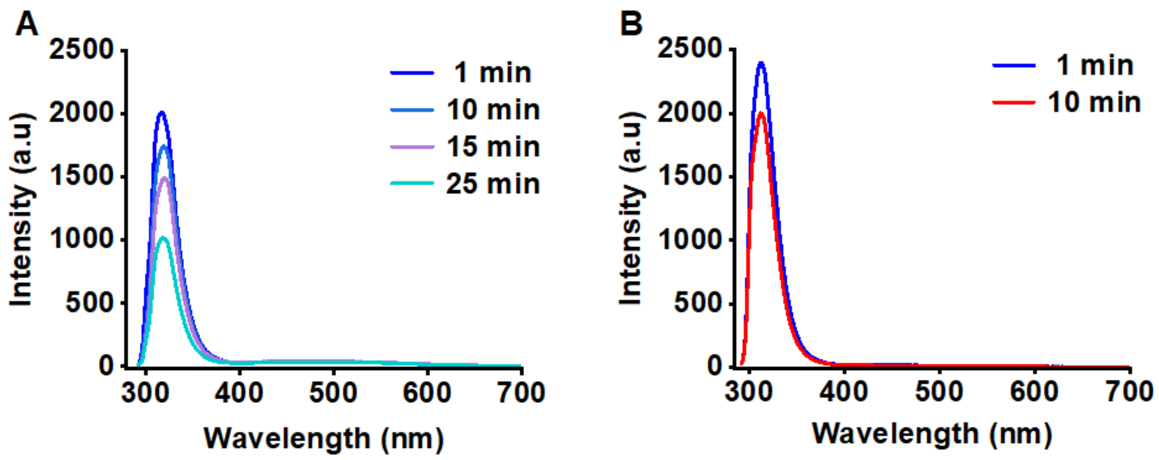

Supplementary Figure 131: Time-dependent Fluorescence emission spectra of A) 10 mM **1b** and B) Mixed library of 10 mM **1b** and10 mM **1g** in 0.6 M borate buffer, pH 9.1.

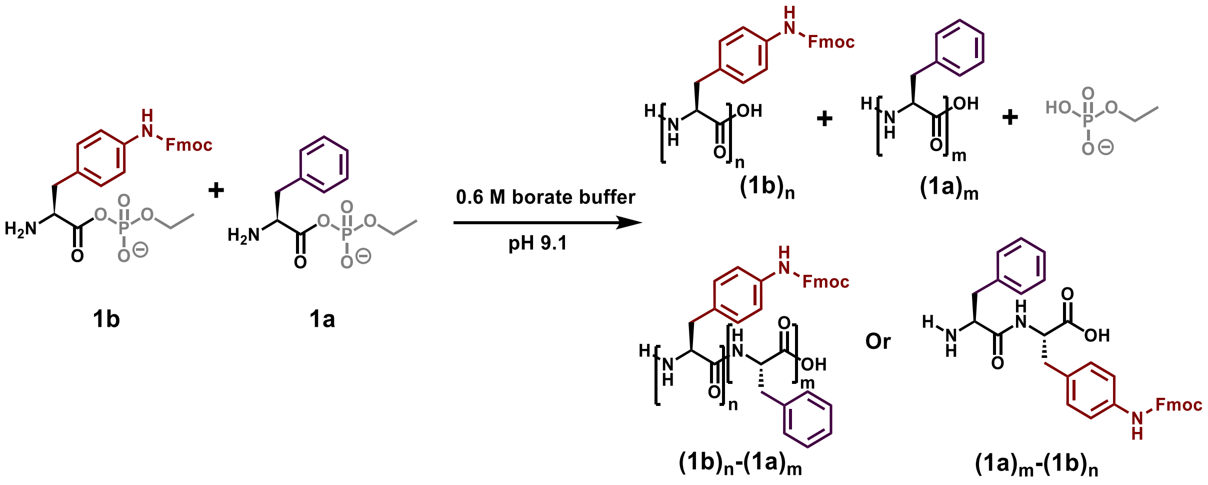

Supplementary Figure 132: Reaction scheme of 10 mM **1b** and 10 mM **1a**.

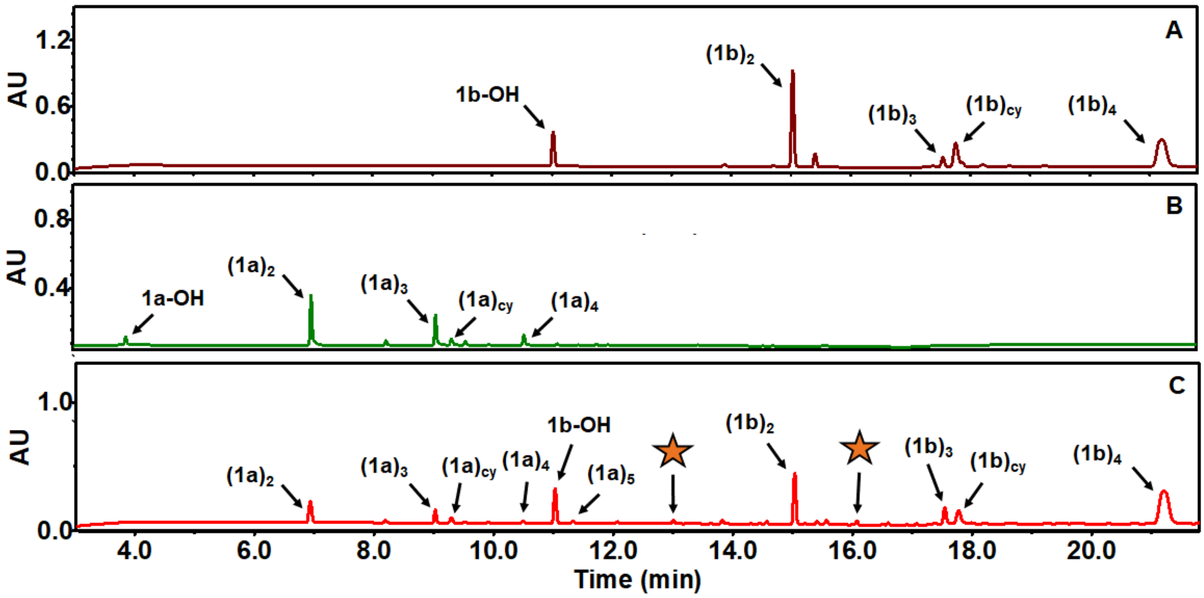

Supplementary Figure 133: UPLC chromatograms of A) 10 mM **1b**, B) 10 mM **1a** and C) 10 mM **1b** with10 mM **1a** (1:1), in 0.6 M borate buffer, pH 9.1. Measurements were taken after 24 hours.

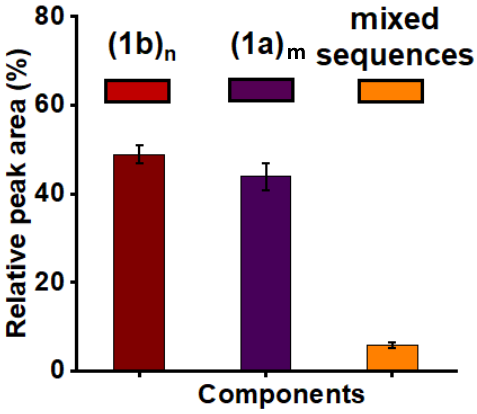

Supplementary Figure 134: Bar graph showing the sum of all library components (grouped as homo or hetero oligomers) formed between 10 mM **1b** and 10 mM **1a** in 0.6 M borate buffer, pH 9.1. Peptide coupling yields were measured after 24 hours. Error bars represent the standard deviation of three independent experiments.

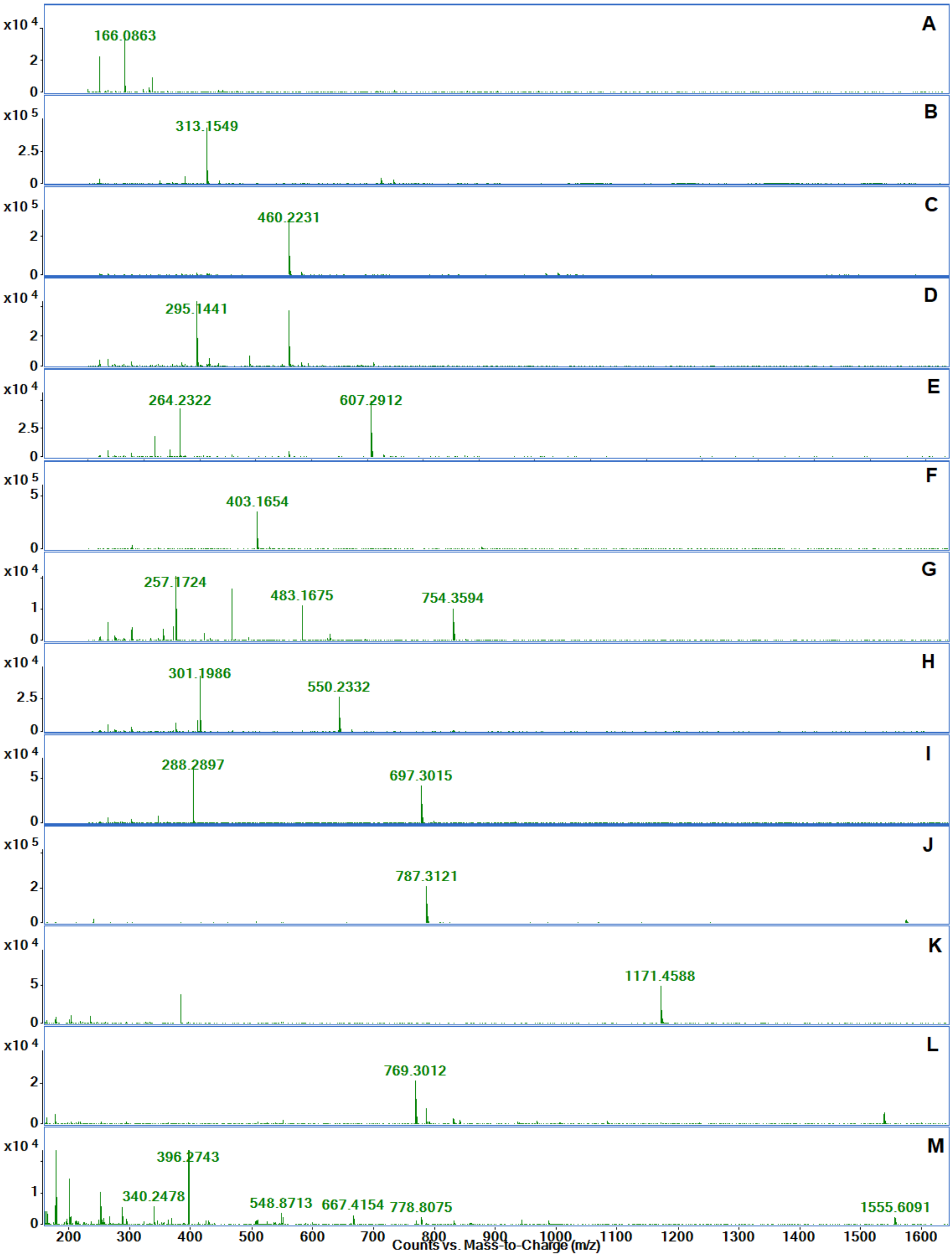

Supplementary Figure 135: Mass spectra of homo and hetero oligomers formed between 10 mM **1b** and 10 mM **1a** (1:1) from figure A-M found in Supplementary Figure 133C, obtained from the LC-MS analysis.

Supplementary Table 5: Mass of homo and hetero oligomers formed from 10 mM **1b** and 10 mM **1a** found in Supplementary Figure 133 C.

**Mass spectra Fig. No**	**Peptides formed**	**Distribution (%)**	**Retention time (min)**	**Calculated m/z $[M+H]^+$**	**Observed m/z $[M+H]^+$**
135 A	$(1a)_1$	7.71	3.86	166.0863	166.0863
135 B	$(1a)_2$	18.83	6.92	313.1547	313.1549
135 C	$(1a)_3$	8.62	9.011	460.2236	460.2231
135 D	$(1a)_{cy}$#	5.93	9.28	295.1441	295.1441
135 E	$(1a)_4$	2.24	10.48	607.2920	607.2912
135 F	$(1b)_1$	10.02	11.01	403.1652	403.1654
135 G	$(1a)_5$	2.39	11.32	754.3599	754.3594
135 H	hetero dimer* ★	3.58	12.99	550.2342	550.2332
135 I	hetero trimer* ★	2.26	16.06	697.3026	697.3015
135 J	$(1b)_2$	11.41	15.02	787.3126	787.3121
135 K	$(1b)_3$	3.83	17.53	1171.4600	1171.4588
135 L	$(1b)_{cy}$#	4.79	17.77	769.3021	769.3012
135 M	$(1b)_4$	18.33	21.21	1555.6074	1555.6091

*The formation of hetero-dimer corresponds to the molecular weight of **1b-1a** which might also be its constitutional isomer, **1a-1b**. Similarly, hetero-trimer which corresponds to the molecular weight of **1b-(1a)$_2$** could also exhibit constitutional isomers for example **(1a)$_2$-1b** and **1a-1b-1a**.

#Cyclic dipeptides (diketopiperazine) of **1a** and **1b**.

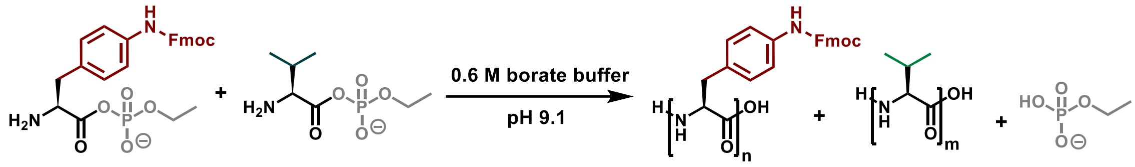

1b **1d** $(\mathbf{1b})_n$ $(\mathbf{1d})_m$

Supplementary Figure 136: Reaction scheme of 10 mM **1b** and 10 mM **1d**.

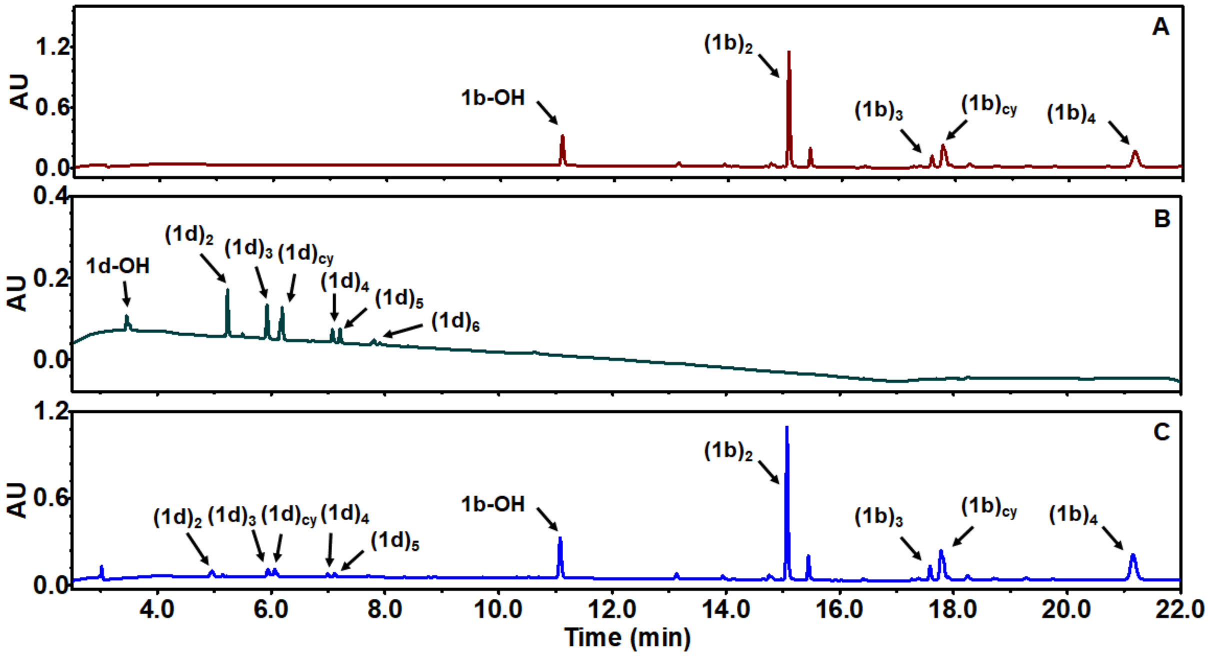

Supplementary Figure 137: UPLC chromatograms of A) 10 mM **1b** B), 10 mM **1d** and C) 10 mM **1b** with 10 mM **1d** (1:1), in 0.6 M borate buffer, pH 9.1. Measurements were taken after 24 hours.

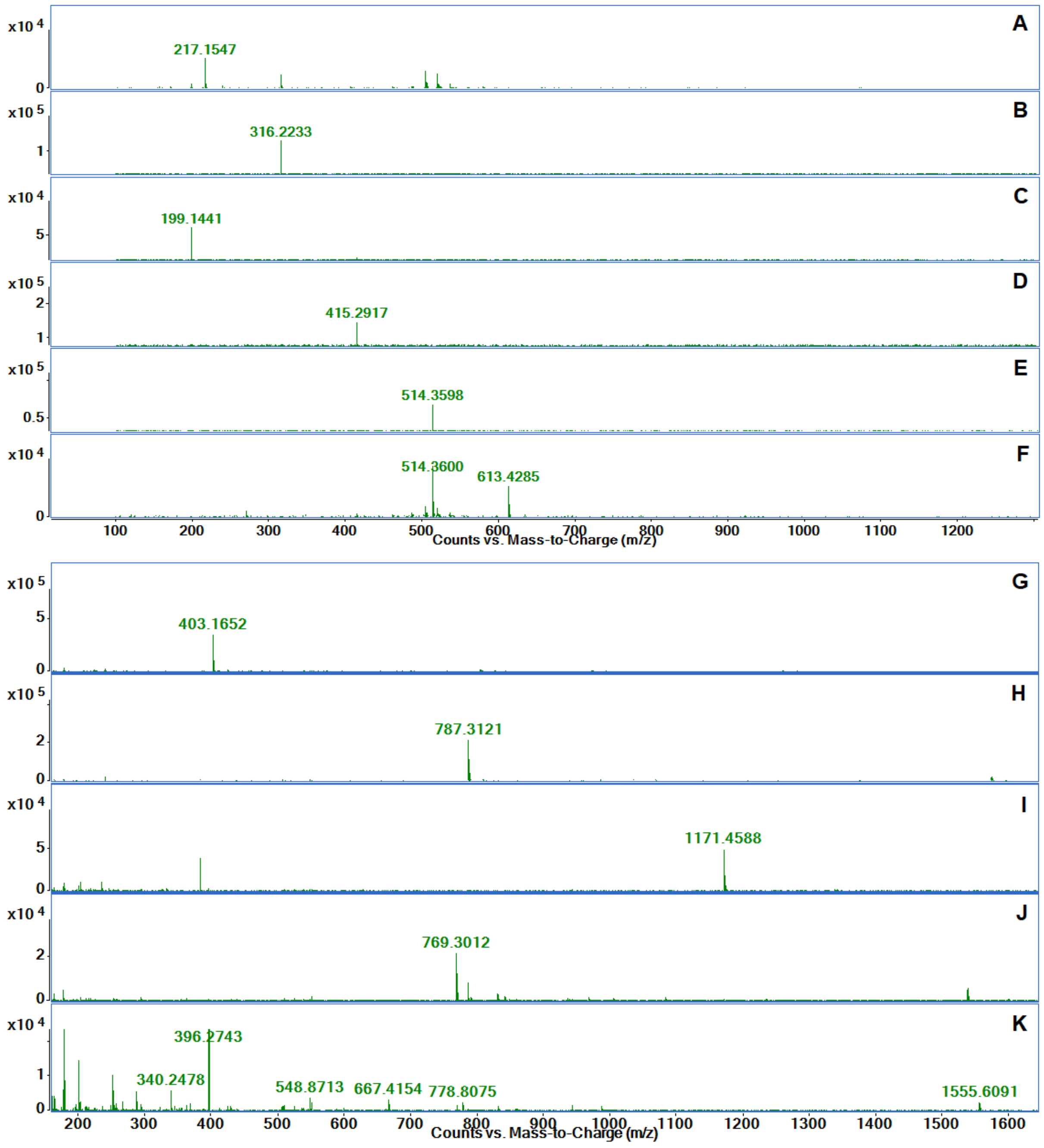

Supplementary Figure 138: Mass spectra of homo oligomers formed between 10 mM **1b** and 10 mM **1d** (1:1) from figure A-K found in Supplementary Figure 137C, obtained from the LC-MS analysis.

Supplementary Table 6: Mass of homo oligomers formed from 10 mM **1b** and 10 mM **1d** found in Supplementary Figure 137 C.

Mass spectra Fig. No	Peptides formed	Distribution (%)	Retention time (min)	Calculated m/z $[M+H]^+$	Observed m/z $[M+H]^+$
138 A	$(1d)_2$	13.79	4.96	217.1547	217.1547
138 B	$(1d)_3$	10.90	5.94	316.2231	316.2233
138 C	$(1d)_{cy}$#	10.72	6.05	199.1441	199.1441
138 D	$(1d)_4$	4.78	6.98	415.2915	415.2917
138 E	$(1d)_5$	6.02	7.11	514.3599	514.3598
138 F	$(1d)_6$	3.64	7.72	613.4283	613.4285
138 G	$(1b)_1$	7.57	11.08	403.1652	403.1652
138 H	$(1b)_2$	22.29	15.06	787.3126	787.3122
138 I	$(1b)_3$	2.39	17.58	1171.4600	1171.4588
138 J	$(1b)_{cy}$#	8.96	17.77	769.3021	769.3012
138 K	$(1b)_4$	8.89	21.15	1555.6074	1555.6091

#Cyclic dipeptides (diketopiperazine) of **1d** and **1b**.

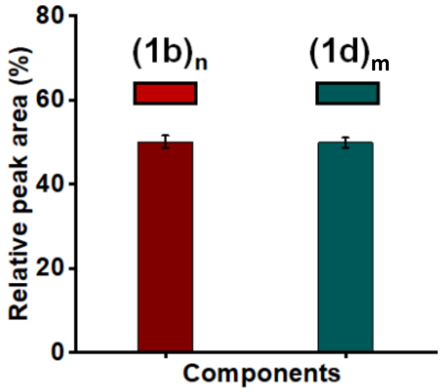

Supplementary Figure 139: Bar graph showing the sum of all library components formed from 10 mM **1b** and 10 mM **1d** in 0.6 M borate buffer, pH 9.1. Error bars represent the standard deviation of three independent experiments. Peptide coupling yields were measured after 24 hours.

4. References

1. Dai K, *et al.* Spontaneous and Selective Peptide Elongation in Water Driven by Aminoacyl Phosphate Esters and Phase Changes. *J. Am. Chem. Soc.* **145**, 26086-26094 (2023).

2. Reddy, P. Nagi, et al. "Positive and negative ion electrospray tandem mass spectrometry (ESI MS/MS) of boc-protected peptides containing repeats of L-Ala-γ 4 Caa/γ 4 Caa-L-Ala: Differentiation of some positional isomeric peptides. *J. Am. Soc. Mass. Spectrom.* **18**, 651-662 (2007).
